# Synthesis of Polyethyleneimines from the Manganese‐Catalysed Coupling of Ethylene Glycol and Ethylenediamine

**DOI:** 10.1002/anie.202306655

**Published:** 2023-06-14

**Authors:** Claire N. Brodie, Aniekan E. Owen, Julian S. Kolb, Michael Bühl, Amit Kumar

**Affiliations:** ^1^ Department of Chemistry University of St Andrews North Haugh KY16 9ST St Andrews UK

**Keywords:** Ethylene Diamine, Ethylene Glycol, Manganese, Pincer, Polyethyleneimine

## Abstract

Polyethyleneimines find many applications in products such as detergents, adhesives, cosmetics, and for processes such as tissue culture, gene therapy, and CO_2_ capture. The current state‐of‐the‐art technology for the production of the branched polyethyleneimines involves aziridine feedstock which is a highly toxic, volatile and mutagenic chemical and raises significant concern to human health and environment. We report here a novel method for the synthesis of branched polyethyleneimine derivative from ethylene glycol and ethylenediamine which are much safer, environmentally benign, commercially available and potentially renewable feedstock. The polymerisation reaction is catalysed by a complex of an earth‐abundant metal, manganese and liberates H_2_O as the only by‐product. Our mechanistic studies using a combination of DFT computation and experiment suggest that the reaction proceeds by the formation and subsequent hydrogenation of imine intermediates.

## Graphical abstract

A new method for the synthesis of polyethyleneimines is reported based on manganese catalysed coupling of ethylene glycol and ethylene diamine. This avoids the use of the highly toxic feedstock, namely aziridine, that is currently used for the production of this polymer.

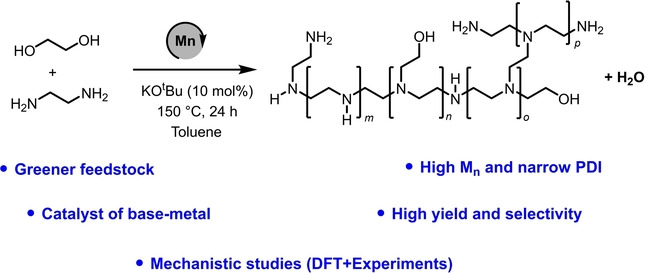

## Introduction

**Figure 1:**
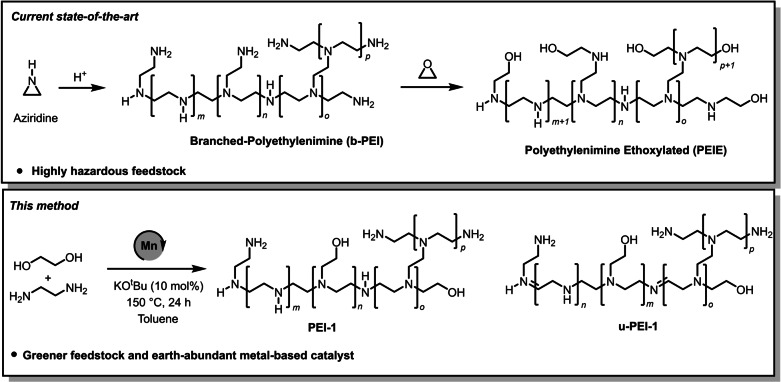
Preparation of branched and ethoxylated polyethyleneimine using the current state‐of‐the‐art process and the method reported herein along with the structure of unsaturated polyethyleneimine (u‐PEI‐1).

Polyethyleneimines with annual global market of around £400 million are found in linear, branched, and ethoxylated form and have a number of applications such as in detergents, adhesives, cosmetics, and water treatment agents. Recently, they have also been employed for a number of biomedical applications such as tissue culture, drug delivery, gene delivery[^1^, ^2^, ^3^, ^4^] as well as for CO_2_ capture,^[5]^ and optoelectronic devices.^[6]^ The branched polyethyleneimines are produced from the acid catalysed ring opening polymerisation of ethyleneimine or aziridine (Figure 1). Polyethyleneimine ethoxylated (PEIE) can be formed from the reaction of polyethyleneimine with ethylene oxide (Figure 1).^[7]^ The main drawback of the current synthetic technology is that the feedstock—aziridine—is a highly reactive, toxic, corrosive, mutagenic and volatile chemical.^[8]^ Furthermore, the polymerisation process is exothermic and releases a considerable amount of heat that is hazardous and therefore it is difficult to store aziridine or ethylenimine due to the associated risk of auto‐polymerisation. Additionally, due to the associated hazard, it can become challenging to take permission from the government regulatory authorities to use this feedstock at the commercial scale.^[9]^ Currently, polyethyleneimines are mainly produced by the BASF and Nippon Shokubai. Thus, the development of an alternative method to make branched polyethylenimines from safer and environmentally benign feedstock will benefit human health, environment and economy.

Catalytic dehydrogenation is a green and atom‐economic approach for the synthesis of organic compounds.[^10^, ^11^, ^12^] The synthesis of imines has been reported from the dehydrogenative coupling of alcohols and amines.[^13^, ^14^] N‐alkylation of amines using alcohols has also been reported using a dehydrogenative strategy.[^15^, ^16^, ^17^, ^18^, ^19^, ^20^, ^21^, ^22^, ^23^] The dehydrogenative coupling approach has also been utilized for the synthesis of polymers. For example, Robertson has reported the synthesis of polyesters from the dehydrogenative coupling of diols using a ruthenium‐pincer catalyst.^[24]^ Guan^[25]^ and Milstein^[26]^ have independently reported the synthesis of polyamides from the dehydrogenative coupling of diols and diamines using a ruthenium‐pincer catalyst. We,[^27^, ^28^] Robertson,^[29]^ and Liu^[30]^ independently reported the synthesis of polyureas from the dehydrogenative coupling of diamines and methanol/diformamides using Macho‐type pincer complexes. To the best of our knowledge, the synthesis of branched polyethyleneimine derivatives from alcohols, and amines feedstock has not been reported in peer‐reviewed literature. The only precedence can be found in patents published by BASF where the preparation of polyethylenimines has been claimed from diols and diamines or amino alcohols in the presence of precious metals‐based catalysts such as ruthenium or iridium and H_2_ gas.[^31^, ^32^] Another patent claims the synthesis of branched polyethyleneimines through the formation of linear polyethyleneimines followed by their subsequent alkylation using β‐chlorethylene or β‐aminoethylsulphate.^[33]^ The preparation of branched polyethyleneimines directly from 2‐chloroethylamine in a one‐pot, two‐stage process has also been reported.^[34]^ In this approach, 2‐chloroethylamine is first dehydrochlorinated to form aziridine which is then polymerised to form branched polyethyleneimine. Here, we report a direct synthesis of branched polyethylenimine derivatives from the manganese catalysed coupling of ethylene glycol and ethylene diamine (Figure 1). Both ethylene glycol and ethylene diamine are environmentally benign, commercially available and can be sourced from biomass[^35^, ^36^] making the reported method a greener and sustainable alternative to the current state‐of‐the‐art process.

## Results and Discussion

We started our investigation by studying a variety of transition metal catalysts, supported by pincer‐motif, from groups 7, 8 and 9 (complexes **1–5**) that are known for their activity towards catalytic (de)hydrogenation reactions.^[37]^ These unoptimized reactions were performed in the presence of a base (e.g. K_2_CO_3_) at 150 °C for 24 h in THF solvent (Table 1) in a sealed 250 mL Young's flask. Of the precatalysts screened, Mn(PN^H^P‐iPr)(CO)_2_Br (**1**, Mn‐MACHO‐iPr)^[38]^ and Ru(PN^H^P^Ph^)(CO)ClH (**4**, Ru‐MACHO) successfully mediated the formation of polymer, generating a mixture of unsaturated‐poly(ethyleneimine) [**u‐PEI‐1**, unsaturation is due to the presence of C=N bond], poly(ethyleneimine) [**PEI‐1**] and poly(ethyleneamide) [**PA**, (C_2_H_3_NO)_*n*_] products (Figure 1, Table 1). IR and ^13^C{^1^H} NMR spectra suggested the formation of branched over linear polyethyleneimine.^[39]^ NMR spectra (^1^H NMR, δ_H_: 3.5–4 ppm, ^13^C{^1^H} NMR, δ_C_: ≈60–70 ppm), IR spectra (br m≈3200–3300 cm^−1^), and ESI‐MS (Figure S84–87) analysis confirmed the presence of ethoxy groups in the polymer chain (see Supporting Information). Some possible evidence of ether functionality (^13^C{^1^H} NMR, δ_C_: ≈71 ppm, and IR stretching frequency in the range of 1150‐1000 cm ^‐1^) is also observed. Of note, when the *tert*‐butyl derivative (**2**, Mn‐MACHO‐tBu)^[40]^ instead of **1** was used, no coupling products were observed and the reaction returned unreacted starting materials. In all cases and under the conditions shown in Table 1, the conversion to polymeric products was low, as indicated by low isolated yields, and the requirement to remove residual ethylene glycol (identified by ^1^H and ^13^C{^1^H} NMR spectroscopies)^[41]^ from the product mixture by distillation. As higher conversion was obtained using complex **1**, this earth‐abundant Mn‐precatalyst was taken forward for optimization studies.

**Table 1:**
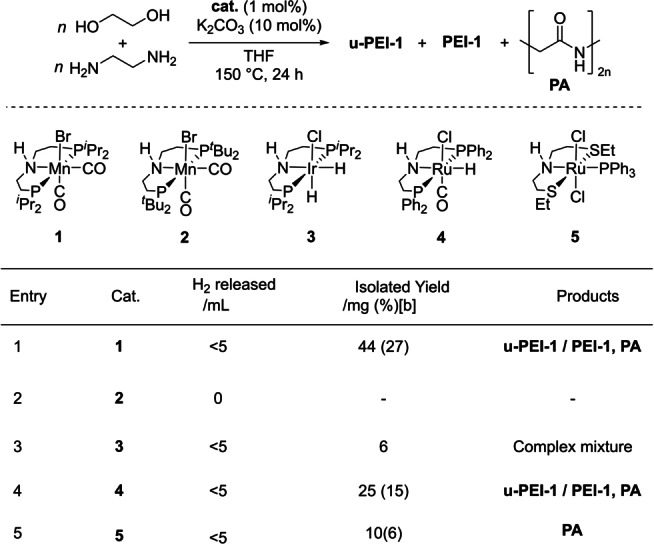
Optimisation of precatalyst choice for the coupling of ethylenediamine and ethylene glycol.^[a]^

[a] Experimental conditions: **1** (1 mol %), K_2_CO_3_ (10 mol %), ethylene glycol (2 mmol), ethylene diamine (2 mmol), 150 °C, 24 h, sealed 250 mL system; [b] theoretical yield based on exclusive formation of major product, u‐PEI‐1.

Decreasing the reaction vessel size to 100 mL and increasing the temperature to 170 °C resulted in a similar yield (26 %) and selectivity of the reaction, producing a mixture of **u‐PEI‐1**, **PEI‐1** and polyamide, and releasing 40 mL of gas (Table 2, Entry 1). A ^13^C{^1^H} NMR spectrum (D_2_O) of the reaction products shows signals at δ_C_ 179.8 and 155.4 ppm, attributed to amide (PA) and imine functionalities, respectively. Infrared analysis of the product mixture showed the presence of bands at ν1634 cm^−1^ and ν1577 cm^−1^, attributed to C=N/C=O for **u‐PEI‐1** and **PA** and N−H for **PEI‐1** and **PA**, respectively. Doubling the reaction time to 48 h did not improve the isolated yields obtained, however the volume of H_2_ evolved did halve in this time, which could indicate further conversion to polyethyleneimine (**PEI‐1**) from unsaturated polyethyleneimine (**u‐PEI‐1**) intermediate—although both were still present in the resulting mixture (Table 2; Entry 2). Increasing the loading of K_2_CO_3_ from 10 mol % to 50 mol % (Table 2; Entry 3) resulted in a similar volume of H_2_ being evolved, however, the presence of residual K_2_CO_3_ obfuscated spectroscopic analysis.

**Table 2:** Optimisation of reaction conditions for the dehydrogenative coupling of ethylene glycol and ethylene diamine with complex **1**.^[a]^

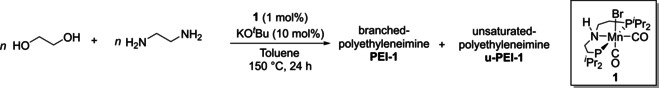											
Entry	1 [mol %]	Base [mol %]	Solvent	H_2_ released [mL]	Product(s)	Yield/mg [%]^[b]^	*M*_n_^[c]^ [g mol^−1^]	*Đ* ^[c]^	*T*_g_ [°C]	*T*_m_ [°C]	*T*_d_^[d]^ [°C]
1^[e]^	1	K_2_CO_3_ (10)	THF	40	u‐PEI‐1, PEI, PA	42 (26)	–	–	–	139.6	245
2^[f]^	1	K_2_CO_3_ (10)	THF	20	**u‐PEI‐1**, PEI‐1, PA	22 (13)	38 500	1.4	–	182.8, 187.9	260
3^[e]^	1	K_2_CO_3_ (50)	THF	15	N/A	110^[g]^	–	–	–	139.4	261
4	1	K_2_CO_3_ (10)	THF	<5	u‐ PEI‐1, PEI‐1	50 (29)	58 600	1.2	–	169.5, 180.8	229
5^[e]^	2	K_2_CO_3_ (10)	THF	15	**u‐PEI‐1**, PEI‐1	75 (44)	58 600	1.2	–	180.9, 191.8	259
6^[e]^	1	KO^*t*^Bu (10)	THF	<5	u‐PEI‐1, **PEI‐1**	49 (28)	48 400	1.2	−32.9	–	269
7	1	KO^*t*^Bu (10)	THF	<1	**PEI‐1**	32 (18)	22 600	1.4	–	–	250
8^[e,h]^	1	KO^*t*^Bu (10)	THF	<5	u‐PEI‐1, **PEI‐1**	55 (32)	24 100	1.3	–	176.1, 181.8	254
9^[e,i]^	1	KO^*t*^Bu (10)	THF	<5	u‐PEI‐1, **PEI‐1**	44 (26)	27 000	1.3	−36.5	179.6, 191.6	240
10	1	KO^*t*^Bu (10)	Toluene	<1	**PEI‐1**	160 (92)	59 000	1.1	−31.3	189.8	235
11^[e]^	1	K_2_CO_3_ (10)	Toluene	<5	u‐PEI‐1, **PEI‐1**	164 (95)	26 900	1.9	–	166.5	238
12^[e,j]^	1	KO^*t*^Bu (10)	–	<5	**PEI‐1**, i^[k]^	113 (65)	61 600	1.1	−30.7	151.4	237
13^[e,l]^	1	KO^*t*^Bu (10)	Toluene	<5	**PEI‐1**	116 (67)	63 700	1.1	−32.2	176.0	238
14^[e,m]^	1	KO^*t*^Bu (10)	Toluene	<5	**PEI‐1**	99 (57)	62 300	1.1	–	–	250

[a] Experimental conditions: 170 °C, 24 h, 2 mmol C_2_H_6_O_2_ [0.5 M in THF or toluene], sealed 100 cm^3^ system; [b] Based on major product (indicated in bold), all yields are isolated yields; [c] Determined by GPC—see text for limitations; [d] Defined as 5 % mass loss after solvent loss; [e] 170 °C; [f] 48 h; [g] contains residual K_2_CO_3_; [h] 4 : 1 [C_2_H_8_N_2_]:[C_2_H_6_O_2_]; [i] 250 mL sealed system; [j] no solvent; [k] unidentified impurity; [l] 1.0 M [C_2_H_6_O_2_]; [m] H_2_O (2 equivalents to C_2_H_6_O_2_).

Reduction of the temperature to 150 °C (Entry 4) in a 100 mL sealed system improved selectivity of the reaction to a mixture of water‐soluble **PEI‐1** and **u‐PEI‐1** (i.e. no polyamide observed). A ^13^C{^1^H} NMR (D_2_O) spectrum obtained of this product mixture showed a signal at δ_C_ 164.6 ppm, attributed to the imine functionality of **u‐PEI‐1**. Carrying out the reaction at 120 °C showed little to no‐conversion, with unreacted ethylene glycol the major (>95 %) species observed post work‐up (see Supporting Information). Using 2 mol % of complex **1** and 10 mol % of K_2_CO_3_ for 24 h at 170 °C resulted in a mixture of **u‐PEI‐1** and **PEI‐1** with moderate yield (44 %), releasing 15 mL of H_2_ gas (Table 2; Entry 5). Unsaturation under these conditions is spectroscopically indicated by a strong absorbance at ν1655 cm^−1^ and weak signal at δ_C_ 164.6 ppm indicative of an imine functionality.

A further increase in selectivity to saturated **PEI‐1** is gained through application of KO^*t*^Bu, rather than K_2_CO_3_, as a base (Table 2; Entry 6), indicated through a reduction in the shoulder corresponding to ν_C=N_ in the resulting infrared spectrum (see Supporting Information, Figure S12F). However, isolated yields remain relatively unchanged (*c.f*. 26 % and 28 % for K_2_CO_3_ and KO^*t*^Bu, respectively). These data suggest the significance of base in a hydrogen borrowing process for the synthesis of **PEI‐1** via formation and hydrogenation of imines; a phenomenon that has been commented upon before.[^15^, ^42^, ^43^] The role of potassium *tert*butoxide in lowering the barrier for aldehyde hydrogenation through aiding alcohol release from the metal center has also been recently reported.^[44]^ Indeed, performing the reaction at 150 °C with KO^*t*^Bu (10 mol %) now almost exclusively forms **PEI‐1** (Table 2; Entry 7), albeit with very poor conversion (isolated yield 18 %).

Increasing the vessel size from 100 mL to 250 mL or changing the ratio of ethylene glycol: ethylene diamine from 1 : 1 to 1 : 4 made little difference to the yield obtained and did not change the observed selectivity significantly (Table 2; Entries 8 and 9, respectively). Remarkably, a significant increase to the reaction yield was obtained through the use of toluene as a solvent with KO^*t*^Bu base, allowing selective formation of branched‐polyethyleneimine (**PEI‐1**) exclusively (ν_N−H_ 1577 cm^−1^) with isolated yield of 92 % (Table 2; Entry 10). Use of toluene with K_2_CO_3_ base retains a high yield (95 %), but some degree of unsaturation remains (ν_C=N_ 1649 cm^−1^ and δ_C_ 164.6 ppm), as shown in Table 2; Entry 11.

As both substrates for this transformation are liquids, we also attempted the reaction in the absence of solvent. However, alongside the generation of **PEI‐1**, performing the reaction neat produces some unidentified side‐product (Table 2; Entry 12). This may be due to poor mixing of these small scale (2 mmol) reactions. As such, the reaction was also conducted at higher concentration (1 M vs 0.5 M [substrate]), which did retain selectivity, but a drop in isolated yield to 67 % (*c.f*. 92 %) at this higher concentration was noted (Table 2; Entry 13). Addition of water to the reaction (2 equivalents to diol) at the onset results in the formation of **PEI‐1** in moderate yield (Entry 14, 57 %). Additionally, no conversion to **PEI‐1** or **u‐PEI‐1** was observed when the reaction was carried out under open conditions (see Supporting Information), or in the absence of any of: catalyst, base, or ethylene glycol, with these control reactions all returning unreacted starting materials. Therefore, the optimized conditions for the coupling of ethylene glycol and ethylene diamine to **PEI‐1** are as follows: **1** (1 mol %), KO^*t*^Bu (10 mol %), 24 h, toluene [0.5 M ethylene glycol/ethylene diamine], 150 °C.

Thermal gravimetric analysis of the **PEI‐1** samples obtained often displayed the presence of entrained water/solvent in several samples through a small (<10 %) mass loss around 100–120 °C, despite drying the samples under reduced pressure at 120 °C for several hours post work‐up. After this initial mass loss, the samples were relatively stable up to ≈230 °C, after which decomposition began. Decomposition temperature, *T*_d_ (defined as 5 % mass loss, after solvent loss) of the isolated polymers were found to be in the range of 235–269 °C. Differential scanning calorimetry performed on the prepared samples (−50–200 °C) revealed low temperature glass transition around −32 °C (when observed) and *T*_m_ ranging between 167–192 °C. Where mixtures of **PEI‐1** and **u‐PEI‐1** were produced, two exothermic events, which could be attributed to localized melting regions, were observed. Where present, when **PA** is produced alongside the desired products, this led to a reduction in the observed melting temperature. These observed thermal characteristics are in line with those previously reported samples of polyethyleneimines.[^6^, ^7^, ^39^]

The molecular weight and dispersity of each sample of polyethyleneimine was investigated through gel permeation chromatography (relative to PEG/PEO standards) using H_2_O eluent. In all cases, high molecular weight material (M_*n*_>24 000 g mol^−1^) with narrow polydispersity (*Ð* 1.1–1.4 typically) was produced. Importantly, it should be noted that, under our GPC measurement conditions, a commercial sample of branched PEI (expected molecular weight of 10 000 g mol^−1^) returned *M*_n_≈3× higher than reported, and our calibration is only valid between 200–50 000 g mol^−1^ due to the size exclusion limitations of our column. Therefore, caution must be applied when considering the molecular weights presented here. Crucially, what can be inferred from the GPC data obtained is that the products obtained are: polymeric in nature; have narrow dispersity and likely have high molecular weight (*M*_n_>10 000 g mol^−1^, *c.f*. commercial b‐PEI, see Supporting Information).

**Figure 2:**
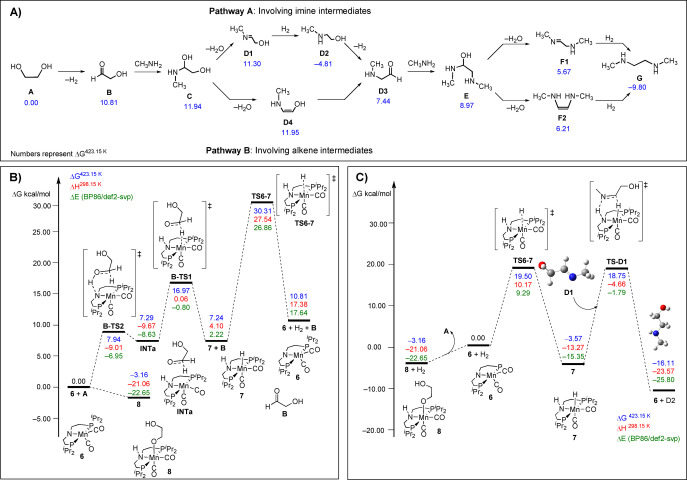
a) Pathways and thermodynamics driving forces for the formation of dimethyl ethylenediamine (G) from ethylene glycol; b) Free energy profiles for the proposed pathways for dehydrogenation of ethylene glycol (**A**) to give glycol aldehyde (**B**); c) Free energy profiles of the proposed pathways for hydrogenation of **D1** (*trans*‐methyl imine ethanol) to give methylamine ethanol, **D2**.

With reaction condition optimization and product characterization in hand, we turned to DFT computation to probe the mechanism of the catalytic coupling of ethylene glycol and ethylene diamine. Using the same methodology as in our work on polyurea,^[28]^ we first studied thermodynamic driving forces for the reaction of ethylene glycol with methyl amine to form dimethyl ethylenediamine (**G**) through various pathways at the PBE0‐D3[pcm,THF]/def2‐TZVP//RI‐BP86[pcm,THF]/def2‐SVP level of theory. Lowest‐lying pathways are shown in Figure 2A (full details in the Supporting Information, Section 2.1, Scheme S1). The reaction starts with the dehydrogenation of ethylene glycol to form the cis conformer of glycol‐aldehyde (**B**) with Δ*G*=10.81 kcal mol^−1^. Methylamine is then added to hydroxyl ethanal to afford the anti‐conformer of *N*‐methyl ethylene glycol (**C**) which dehydrates to form either an imine (*trans*‐methyl imine ethanol [**D1**], pathway I) or an alkene (*cis*‐methylamine ethenol [**D4**], pathway II) both leading to form an aldehyde, *cis*‐methylamine aldehyde (**D3**). The reaction of **D3** with methyl amine followed by dehydration again leads to the formation of an imine (**F1**) or an alkene (**F2**) of similar energy which upon hydrogenation can lead to the formation of **G**. Our studies showed that the energetics for the formation of the branched oligomer is similar or slightly more favourable than those of the linear oligomers as seen in Schemes S4a and S4b (Supporting Information). We then studied the mechanistic pathway involving manganese complexes as described below.

### Dehydrogenation of ethylene glycol

**Scheme 1:**
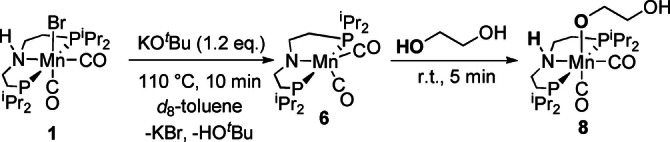
Reactivity of **6** towards ethylene glycol.

Based on well documented literature of pincer chemistry,[^45^, ^46^] it is likely that the first step is the generation of amido complex **6** from the reaction of precatalyst **1** with base (e.g. KO^t^Bu) that will immediately react with ethylene glycol via metal‐ligand cooperation^[47]^ to form an alkoxide complex **8** as also observed by us experimentally (see below, Scheme 1). At our level of theory, dehydrogenation of ethylene glycol to give glycol aldehyde (**B**) using complex **6** is similar to that of MeOH as recently reported by us, i.e. the reaction proceeds in a stepwise transfer of hydrogen via a zwitterionic intermediate, **INTa** (Figure 2B). The highest barrier of the two steps is found for **B‐TS1** corresponding to the transfer of hydride at Δ*G* ^≠^=16.97 kcal mol^−1^ to afford the separated product, **B** and the hydrogenated catalyst **7**. The regeneration of the active catalyst **6** from **7** (H_2_ loss) is the turnover limiting step with an overall barrier of Δ*G* ^≠^=33.47 kcal mol^−1^ at **TS6‐7** relative to the off‐cycle intermediate **8** (see below). We also found that the overall barrier for catalyst regeneration via **TS6‐7** is further reduced by 2.7–5.4 kcal mol^−1^ through participation of protic solvents (e.g. water).^[17]^ While this would probably result in a barrier slightly lower than expected from the required temperature used in experiment, the relation between both is difficult to quantify, and overall the barrier appears to be broadly compatible with the experimental conditions.

### (De)hydration steps

Many of the elementary steps in Figure 2A involve hydration or dehydration steps interconverting alcohols and corresponding unsaturated intermediates. We first computed the barrier for the uncatalysed elimination of water (see Supporting Information, Scheme S3). Established in literature,^[48]^ the strain in such four‐membered transition states in such processes can be alleviated by the involvement of protic substrates acting as proton relays. Interestingly, the involvement of a single water molecule reduces the barriers by ≈20–30 kcal mol^−1^, with Δ*G* ^≠^ between 37.36 and 45.58 kcal mol^−1^, relative to **C** (see Supporting Information, Scheme S3). This is consistent with previous reports by Poater, where the energy barrier to dehydration decreases from 54.2 kcal mol^−1^ to 39.5 or 34.1 kcal mol^−1^ when assisted by two water or benzyl alcohol molecules, respectively.^[49]^ Realising the high barrier observed in the dehydration step, we explored the possible involvement of Mn catalysts such as **6** in these processes. In view of the known ability of complex **6** to heterolytically split the OH bond in water,^[50]^ it is reasonable to assume that a corresponding OH activated intermediate can be involved, viz. the [Mn(OH)‐N(H)] hydrated complex, **9**. Formation of this complex is slightly exergonic by Δ*G*=−0.59 kcal mol^−1^ (see Supporting Information, section 2.6). Our computational studies showed that the barrier for the formation of an imine product (e.g. **D1**, Δ*G* ^≠^=26.35 kcal mol^−1^ or **F1**, Δ*G* ^≠^=25.1 kcal mol^−1^) is much lower than that of an alkene product e.g. (**D4**, Δ*G* ^≠^=41.78 kcal mol^−1^ or **F2**, Δ*G* ^≠^=36.8 kcal mol^−1^). Full details on the pathways and barriers for the manganese catalysed (de)hydration steps can be found in the Supporting Information (section 2.6).

### Hydrogenation of imine and olefin intermediates

Finally, we turn to steps involving hydrogenation of unsaturated intermediates. Hydrogenation of imines (C=N) have been reported to be efficiently catalysed by a triazine core‐based (PN_5_P) Mn catalyst through an outer sphere mechanism to afford amines.[^51^, ^52^] In contrast, hydrogenation of alkenes (C=C) have been reported to be challenging using such complexes, although Kirchner and co‐workers have shown that alkyl Mn^I^ complexes can be used under base‐free conditions to hydrogenate mono‐ and di‐substituted alkenes via alkyl migration.^[53]^ We have shown computationally that the dehydrogenation of amides affording ketenes (with a C=N moiety) can be efficiently catalysed by **6**.^[28]^ The same is thus to be expected for the reverse reaction, hydrogenation of imines. We have now corroborated this by explicit study of the formation of methylamine ethanol (**D2**) by the hydrogenation of **D1**. This process is initiated by the well‐studied H_2_ activation by complex **6**, followed by H‐transfer to **D1** through the transition state **TS‐ D1** (Figure 2C). The hydrogen transfer process occurs in a concerted manner and the overall barrier for the general process is Δ*G* ^≠^=22.33 kcal mol^−1^ as shown in Figure 2C. Similar results are obtained for the catalytic hydrogenation of the C=N double bond in **F1** affording **G**. Hydrogen transfer to **F1** occurs in a rather concerted fashion via **F1‐TSb** (Scheme S12) with a barrier of Δ*G* ^≠^=20.40 kcal mol^−1^. In contrast, hydrogenation of non‐polar C=C double bond by complex **7** is indicated to be much less favourable. As an illustrative example, that of **F2** to form **G** (the same final product as obtained from reduction of the imine moiety in **F1**, c.f. Scheme S12) is illustrated in Scheme S13. As with hydration steps of such olefins, a large barrier is computed for hydrogenation by **7** (Δ*G* ^≠^=33.85 kcal mol^−1^).

To supplement the DFT computation of the mechanism of the catalytic coupling of ethylene glycol and ethylene diamine, we have also probed the mechanism through experiment. Our calculations found the turnover limiting step to be dihydrogen loss from complex **7** to regenerate the catalytically active species, **6**. To demonstrate dihydrogen release during the reaction (i.e. proceeding via borrowing hydrogen^[54]^ route) during the reaction, the polymerisation was performed using our optimized conditions (Table 2; Entry 10) in one side of a sealed H‐shaped flask (see Supporting Information section 1.5). H_2_ release from dehydrogenation of ethylene glycol was then demonstrated through the successful hydrogenation of benzaldehyde (2 mol % Pd/C, 8 % conversion after 24 hours as measured by GC‐MS) in the other side of the same sealed H‐flask.

Dehydrohalogenation of complex **1** (δ_P_ 81.4 ppm) to generate the previously reported coordinatively unsaturated species **6** (δ_P_ 113.0 ppm) is demonstrated through reaction of **1** with 1.2 equivalents of KO^t^Bu in *d*_8_‐toluene after heating to 110 °C for 10 minutes.^[55]^ Addition of excess ethylene glycol to complex **6** at room temperature generates the new, O−H activated complex, **8**, quantitatively on time of mixing (Scheme 1). Complex **8** has been characterized by ^1^H, ^13^C{^1^H} and ^31^P{^1^H} NMR spectroscopies. A single resonance is observed in the ^31^P{^1^H} NMR spectrum at δ_P_ 81.3 ppm, and Mn‐coordinated O−H activated ethylene glycol aliphatic proton resonances are observed in the ^1^H NMR with δ_H_ 3.86 and 3.63 ppm, slightly downfield compared to free residual ethylene glycol (observed at δ_H_ 3.30). The spectral details are in agreement with the previously reported alkoxide complexes of manganese containing pincer ligands.[^55^, ^56^, ^57^] This facile addition of ethylene glycol to **6** is calculated computationally to be exergonic by −3.16 kcal mol^−1^ (Figure 2). Therefore, complex **8** is likely an off cycle resting state.

Sampling of the reaction mixture of the catalytic coupling of ethylene glycol and ethylene diamine after 2 hours allowed the in situ speciation present within the catalytic mixture to be probed. Indeed, the off‐cycle complex **8** was observed in the resulting ^31^P{^1^H} NMR spectrum (Figure S99). Also observed in the ^31^P{^1^H} NMR spectrum of the catalytic mixture is a broad resonance with δ_P_ 85.2 ppm, which may correspond to O−H activated oligomers, with general formula Mn(PN^H^P‐^*i*^Pr)(CO)_2_(OR) (where R=oligomer). Evidence for these proposed Mn‐oligomeric species comes from several Mn‐containing oligomeric fragments being observed within the ESI mass spectra obtained of polymeric samples (Figures S87–S90).

## Conclusion

**Scheme 2:**
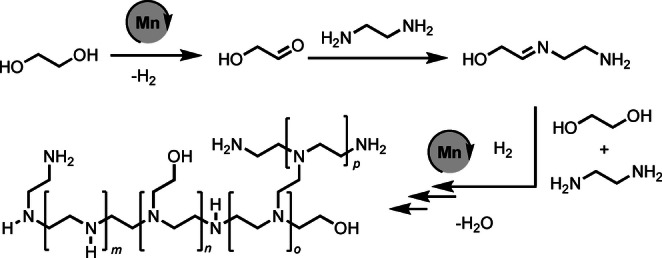
Proposed pathway for the synthesis of branched polyethyleneimines from ethylene glycol and ethylenediamine.

We present herein a new method for the synthesis of polyethyleneimines based on manganese catalysed coupling of ethylene glycol and ethylene diamine. The methodology is significantly greener than the current state‐of‐the‐art as it substitutes a highly toxic feedstock, aziridine with much safer and potentially renewable feedstock (ethylene glycol and ethylene diamine). The use of a catalyst based on earth‐abundant metal is an added advantage of the process. The characterisation studies of the polymer shows that the formed polymer is a branched polyethyleneimine derivative (**PEI‐1**) with high molecular weights (*M*_n_>10 000 g mol^−1^, and narrow PDI (e.g. 1.1–1.4). Based on the DFT computation, experimental and prior studies, we suggest a hydrogen borrowing pathway where the reaction proceeds via the dehydrogenative condensation of ethylene glycol with ethylene diamine to form imine intermediates followed by their subsequent hydrogenation to form polyethyleneimine as depicted in Scheme 2.

## Conflict of interest

The authors declare no conflict of interest.

1

## Supporting information

As a service to our authors and readers, this journal provides supporting information supplied by the authors. Such materials are peer reviewed and may be re‐organized for online delivery, but are not copy‐edited or typeset. Technical support issues arising from supporting information (other than missing files) should be addressed to the authors.

Supporting Information

## Data Availability

The data that support the findings of this study are available in the Supporting Information of this article.
