# Supplementary material for: Synthesis of Polyethyleneimines from the Manganese‐Catalysed Coupling of Ethylene Glycol and Ethylenediamine
Source: Angew Chem Int Ed Engl. 2023 Jun 14;62(29):e202306655. doi: 10.1002/anie.202306655 (PMC11497229; doi:10.1002/anie.202306655)
Supplement: Supplementary file 1 — Supporting Information [file ANIE-62-e202306655-s001.pdf]

Angewandte Chemie
Eine Zeitschrift der Gesellschaft Deutscher Chemiker

Supporting Information

Synthesis of Polyethyleneimines from the Manganese-Catalysed Coupling of Ethylene Glycol and Ethylenediamine

C. N. Brodie, A. E. Owen, J. S. Kolb, M. Bühl, A. Kumar**

Electronic Supporting Information for Manuscript:

Synthesis of Polyethylenimines from the Manganese Catalyzed Coupling of Ethylene Glycol and Ethylenediamine

Claire N. Brodie,[a] Aniekan E. Owen,[a] Julian S. Kolb,[a] Michael Bühl*[a] and Amit Kumar*[a]

[a] *Department of Chemistry, University of St Andrews, St Andrews, Scotland KY16 9ST*

Email:

Amit Kumar: ak366@st-andrews.ac.uk

Michael Bühl: mb105@st-andrews.ac.uk

Table of Contents

1. Experimental Details S3

1.1 General Considerations S3

1.2 Procedure for the coupling of diamines and diols S4

1.3. Pre-catalyst Optimisation S5

1.3.1 Infrared Spectra S7

1.3.2 NMR Spectra S8

1.4. Condition Optimisation/Control Experiments S13

1.4.1 Infrared Spectra S18

1.4.2 TGA S19

1.4.3 DSC S24

1.4.4 NMR Spectra S32

1.4.5 GPC Traces S48

1.4.6 EI-MS spectra S53

1.5 Mechanistic Studies S55

1.5.1 Reaction of **2** with ethylene glycol S55

1.5.3 Evidence for the release of H_2 gas S56

1.5.4 NMR Spectra S57

1.5.6 GC-MS spectra S61

2. Computational Details S62

2.1 Thermodynamic driving forces for the various steps for the formation of G S63

2.2 Thermodynamics for the formation of a cyclic product and dehydration step in the absence of a catalyst S65

2.3 Metal-free Transition States for the formation of D4 and D1 .... S66

2.4 Thermodynamic driving forces for Branched oligomer and Linear oligomer S68

2.5 Dehydrogenation of ethylene glycol S70

2.6 Steps involving dehydration S72

2.7 Hydrogenation of imine and olefin intermediates S77

2.8 Adduct formation of complex 6 .... S79

3. References S80

4. DFT Coordinates S82

1. Experimental Details

1.1 General Considerations

All manipulations, unless otherwise stated, were performed under an argon atmosphere using standard Schlenk line and glove-box techniques. Glassware was oven-dried at 130 °C overnight and flamed under vacuum prior to use. THF and toluene were dried using a Grubbs-type solvent purification system (Innovative Technologies SPS) equipped with a degasser. Pre-catalysts **1**[1] and **2**[2] were prepared in accordance with the literature procedure. KO^{t}Bu (anhydrous; sublimed) and K_2CO_3 (anhydrous) were stored at 80 °C and dried before use. Precatalysts **3-5**, NMR solvents and iPr-PN^{H}P (10 wt% solution in THF) were purchased from Strem Chemicals and used as received. Branched polyethyleneimine (b-PEI, MW 10,000 Da) was obtained from Thermo Scientific.

NMR spectra were recorded on a Bruker AVIII-HD 500 MHz NMR spectrometer at 298 K unless otherwise specified. Residual protio solvent was used as reference for ^{1}H spectra in deuterated solvent samples. All chemical shifts (δ) are quoted in ppm and coupling constants (*J*) in Hz.

Gel permeation chromatography (GPC) was performed on an Agilent 1260 InfinityLab II GPC fitted with a refractive index (RI) detector (35 °C). The single (plus guard column) Agilent PolarGel column setup was contained within an oven (35 °C). H_2O was used as the eluent at a flow rate of 1.0 mL min^{-1}. Samples were dissolved in the eluent (2.0 mg mL^{-1}), filtered (0.2 μm pore size) and run immediately. The calibration was conducted using a series of monodisperse poly(ethylene glycol) (M_n = 194–20,000 g mol^{-1}) and poly(ethylene oxide) (M_n = 30,000–50,000 g mol^{-1}) standards obtained from Agilent Technologies.

Infrared spectra (ATR-FTIR) were collected using a Shimadzu IRAffinity-1. TGA was performed using an Stanton Redcroft STA-780 Series Thermal Analyser between 30–600°C at a heating rate of 10 °C/min under a flow of nitrogen gas (25 mL/min). DSC analyses were performed using a Netzsch DSC204 between –50–200°C at a heating rate of 10 °C/min under a flow of nitrogen gas (20 mL/min) after an initial heat/cool cycle (25–120 °C at 10 °C/min with a 20 minute isothermal at 120 °C) to remove the thermal history of the sample.

GC-MS spectra were collected as solutions in HPLC grade DCM using an Agilent 8860 GC system coupled to an Agilent 5977B EI instrument. EI spectra were collected as solutions in acetonitrile using a Micromass LCT spectrometer.

1.2 Procedure for the coupling of diamines and diols

General method for the coupling of ethylene glycol and ethylenediamine under closed conditions:

A 100 mL or 250 mL ampoule equipped with a J-Young's valve is charged with pre-catalyst (*e.g.* **1**; 10 mg, 0.02 mmol, 1 mol%) and base (*e.g.* KO^{t}Bu, 22 mg, 0.20 mmol, 10 mol%). THF or toluene (4 mL), ethylene glycol (0.11 mL, 2.0 mmol) and ethylenediamine (0.14 mL, 2.0 mmol) are added and the flask sealed under an argon atmosphere before heating to the desired temperature (*e.g.* 170 or 150 °C) for the desired length of time (*e.g.* 24 hours) with stirring. After this period, the reaction vessel is allowed to cool to room temperature and any gas evolved (presumably H_2) during the reaction measured by syringe. The product is extracted into distilled water (5 mL) and any volatile components are removed under reduced pressure at 110 °C.

General method for the coupling of ethylene glycol and ethylenediamine under open conditions:

A 100 mL round bottomed flask equipped with a condenser is charged with pre-catalyst (*e.g.* **1**; 10 mg, 0.02 mmol, 1 mol%) and base (*e.g.* K_2CO_3. 28 mg, 0.20 mmol, 10 mol%). THF or toluene (4 mL), ethylene glycol (0.11 mL, 2.0 mmol) and ethylenediamine (0.14 mL, 2.0 mmol) are added and the reaction heated to reflux under a continuous flow of argon for the desired length of time (*e.g.* 24 hours) with stirring. After this period, the reaction is allowed to cool to room temperature. Any volatile components from the resulting mixture are removed under reduced pressure to isolate the product that is analysed by NMR and IR spectroscopies as well as by TGA and DSC.

1.3. Pre-catalyst Optimisation

Table S1. Catalyst optimisation for the coupling of ethylene glycol and ethylene diamine.[a]

Entry	Catalyst	H_2 released /mL	Isolated Yield /mg (%)[b]	Product(s)
1	**1**	<5	44 (27)	u-PEI-1 / PEI-1, PA
2	**2**	0	-	-
3	**3**	<5	6	Complex mix
4	**4**	<5	25 (15)	u-PEI-1 / PEI-1, PA
5	**5**	<5	10 (6)	Oligo-PA

[a] Experimental conditions: 1 mol% cat., 150 °C, 24 h, 2 mmol ethylene glycol, 2 mmol ethylene diamine, THF (4 mL), 10 mol% K_2CO_3, sealed 250 mL system; [b] theoretical yield based on exclusive formation of major product, u-PEI-1.

Table S1; Entry 1:

^{1}H NMR (D_2O, 500 MHz): δ_H 8.42 (CONH), 8.03 (CONH), 6.59 (CH=N), 6.14 (CH=N), 4.06 (s), 3.90 (s), 3.68 (s), 2.82 (br s).

^{13}C{^{1}H} NMR (D_2O, 126 MHz): δ_c 181.3 (C=O), 179.9 (C=O), 167.7 (C=N), 69.6, 61.4, 58.5 43.6 (broad), 36.3.

IR (ATR-FTIR, cm^{-1}): ν 3227m (N-H, O-H), 2918w (C-H), 1670m (C=O/C=N), 1576s (N-H), 1383m (C-H).

Table S1; Entry 2:

^{1}H NMR (D_2O, 500 MHz) and ^{13}C{^{1}H} NMR (D_2O, 126 MHz) spectra of the reaction product revealed the presence of unreacted starting ethylene glycol (>99%).

Table S1; Entry 3:

^{1}H NMR (D_2O, 500 MHz): δ_H 8.48 (CONH), 8.07 (CONH or HCO end group), 7.55 (CONH), 6.59 (CH=N).

^{13}C{^{1}H} NMR (D_2O, 126 MHz): δ_c 181.3 (C=O), 179.8 (C=O), 172.6 (C=O), 164.6 (C=N), 161.8 (C=N).

Note, due to the small quantity of product isolated from the reaction promoted by complex **3**, no infrared spectroscopic data was obtained.

Table S1; Entry 4:

^{1}H NMR (D_2O, 500 MHz): δ_H 8.40 (CONH), 8.00 (CONH or HCO end group), 7.55 (CONH) 6.62 (CH=N), 3.90 (s), 3.63 (s), 3.38 (s).

^{13}C{^{1}H} NMR (D_2O, 126 MHz): δ_c 179.8 (C=O), 172.6 (C=O), 161.1 (C=N).

IR (ATR-FTIR, cm^{-1}): ν 3219m (N-H/O-H), 2196w (C-H), 1664m (C=O/C=N), 1577s (N-H), 1389m (C-H).

Table S1; Entry 5:

^{1}H NMR (D_2O, 500 MHz): δ_H 7.39 (br s, CONH), 4.03 (s), 3.91 (s), 3.63 (s), 3.39 (s).

^{13}C{^{1}H} NMR (D_2O, 126 MHz): δ_c 179.9 (C=O, amide), 175.3 (C=O, amide), 62.2 (C-OH), 62.1 (C-OH), 52.6 (C-COH), 45.0 (C-COH), 43.9 (C-C=O) and 33.2 (NHC-H).

IR (ATR-FTIR, cm^{-1}): ν 3229m (N-H/OH), 2895w (C-H), 1631m (C=O, amide) 1577vs (N-H), 1394m (C-H).

1.3.1 Infrared Spectra

Note, due to the small quantity of product isolated from the reaction promoted by complex **3**, no infrared data was obtained.

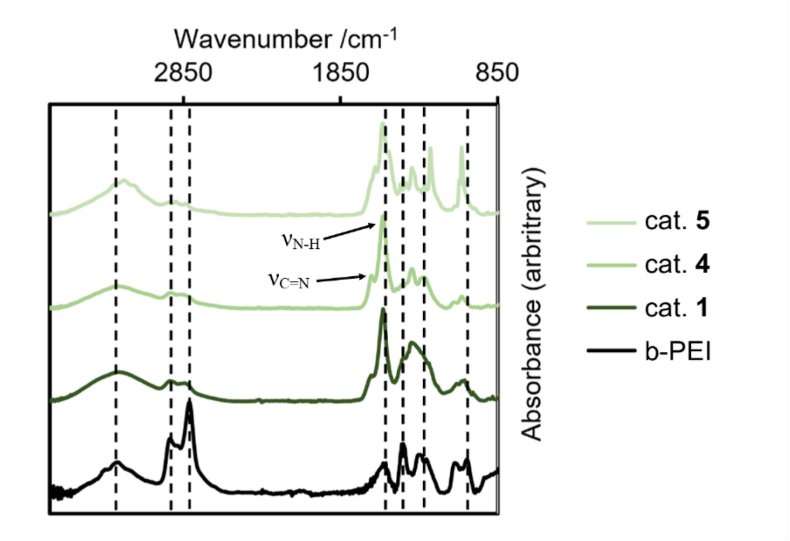

Figure S1 Infrared spectra of product mixtures with various pre-catalysts, compared to a commercial branched-PEI sample.

1.3.2 NMR Spectra

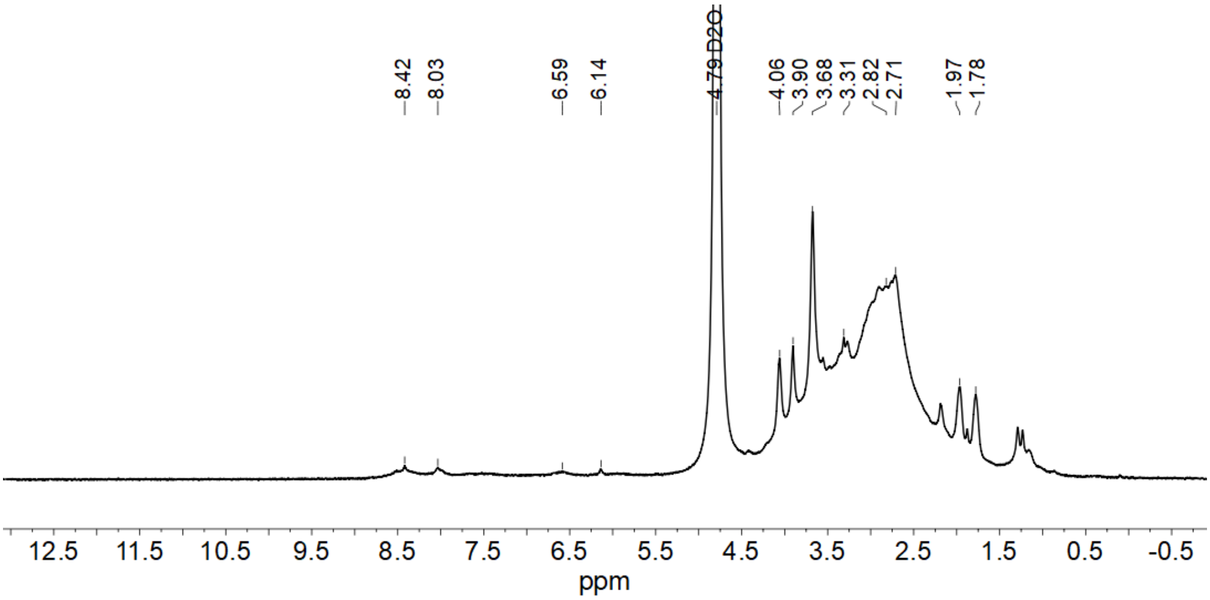

Figure S2 ^{1}H NMR (500 MHz, D_2O) spectrum of resulting mixture from reaction corresponding to Table S1; Entry 1.

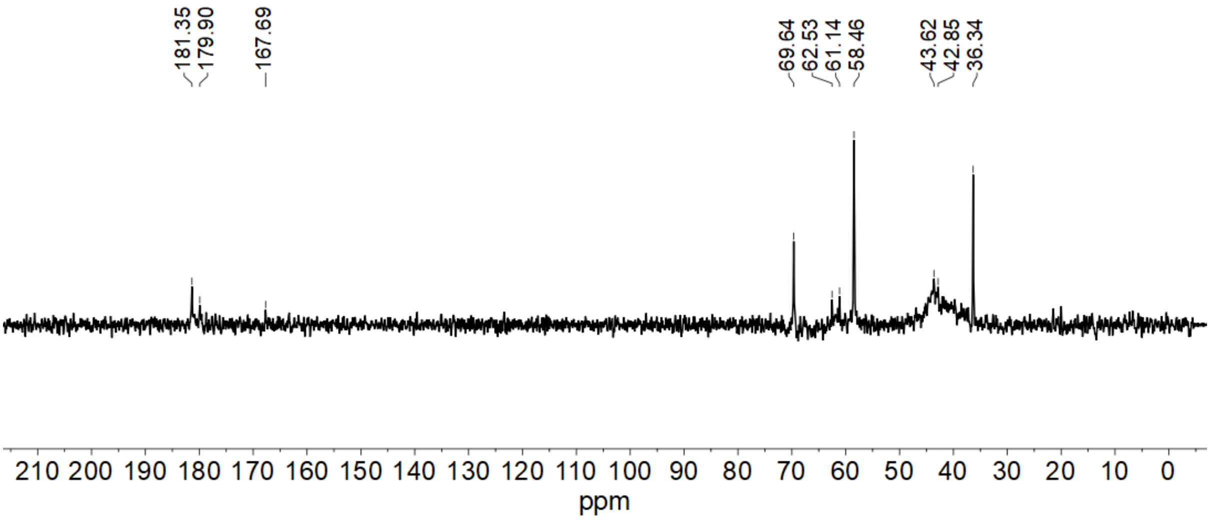

Figure S3 $^{13}C\{^{1}H\}$ NMR (126 MHz, D_2O) spectrum of the resulting mixture corresponding to Table S1; Entry 1.

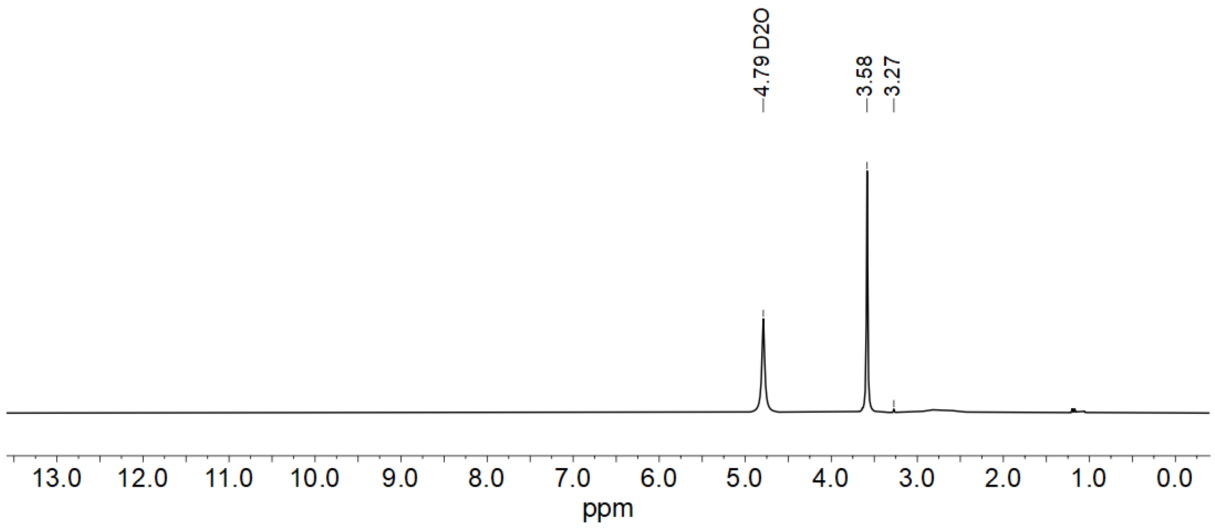

Figure S4 ^{1}H NMR (500 MHz, D_2O) spectrum of the resulting mixture corresponding to Table S1; Entry 2: showing unreacted ethylene glycol.

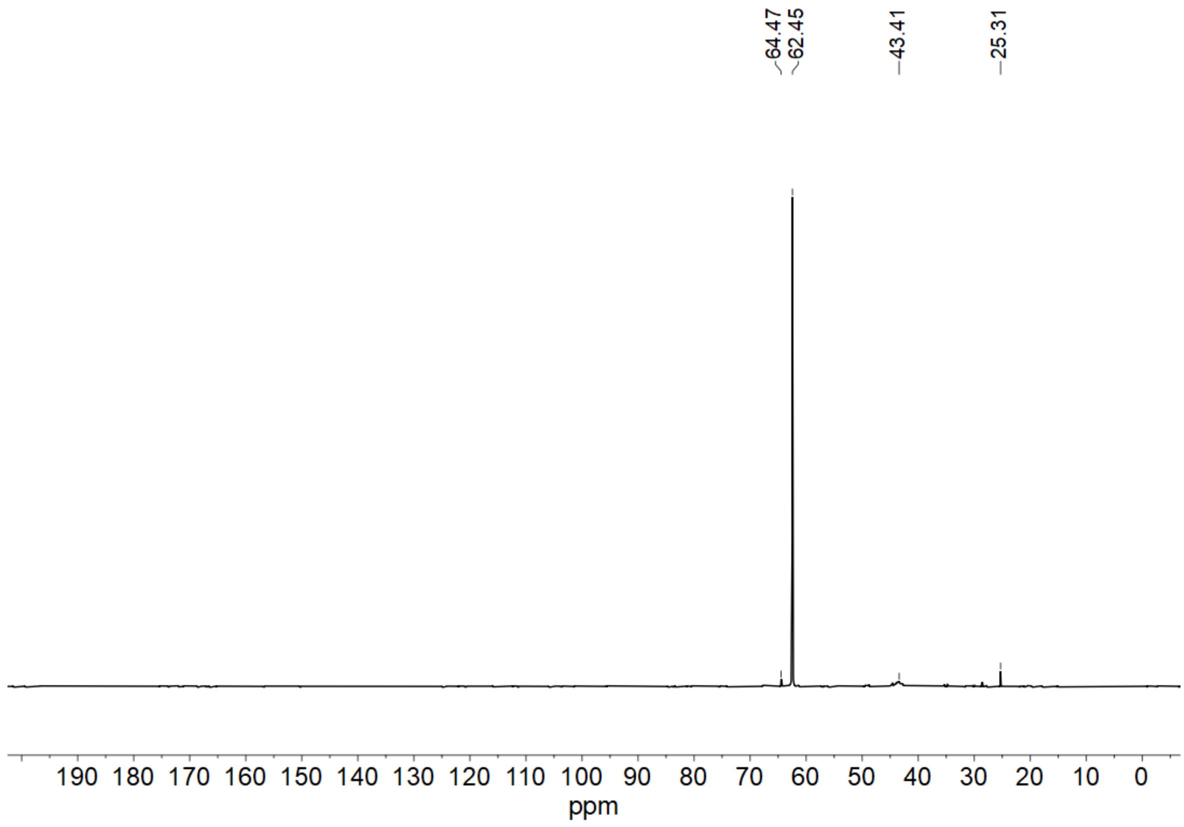

Figure S5 $^{13}C\{^{1}H\}$ NMR (126 MHz, D_2O) spectrum of the resulting mixture corresponding to Table S1; Entry 2: showing unreacted ethylene glycol.

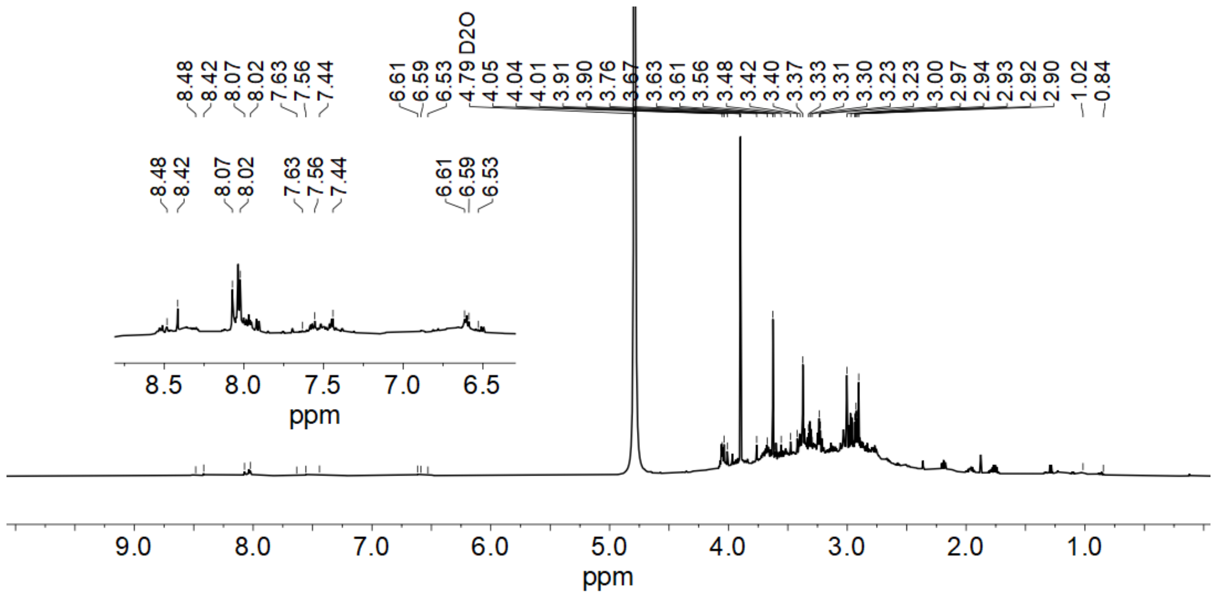

Figure S6 ^{1}H NMR (500 MHz, D_2O) spectrum obtained of mixture of products corresponding to Table S1; Entry 3.

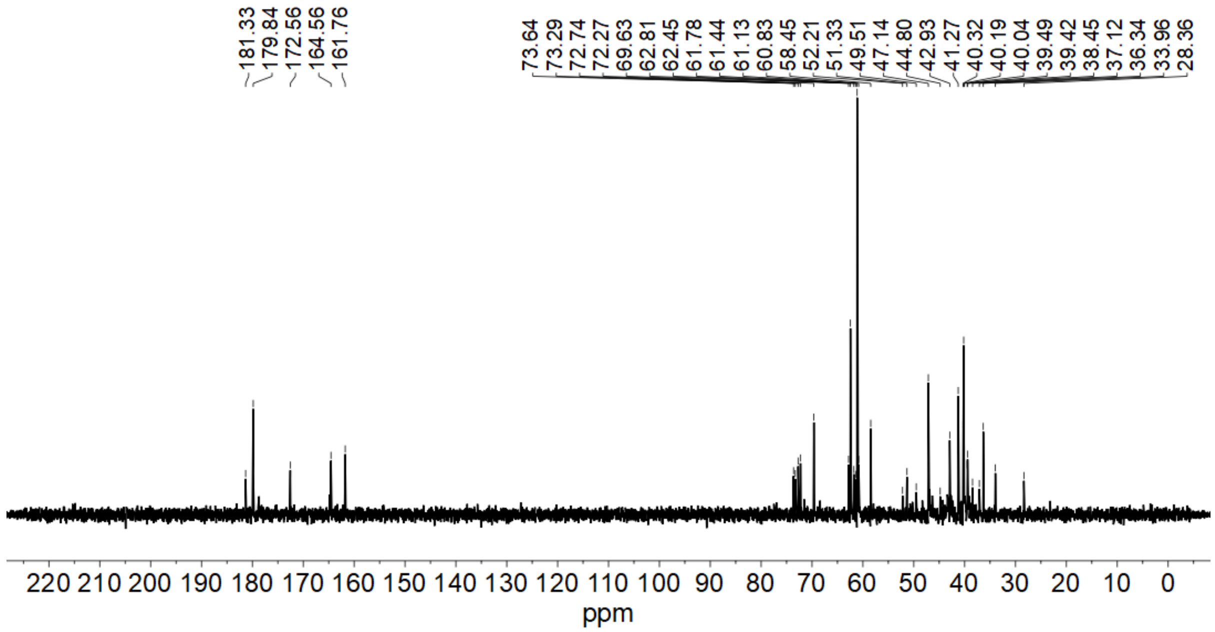

Figure S7 $^{13}C\{^{1}H\}$ NMR (126 MHz, D_2O) spectrum of product mixture corresponding to Table S1; Entry 3.

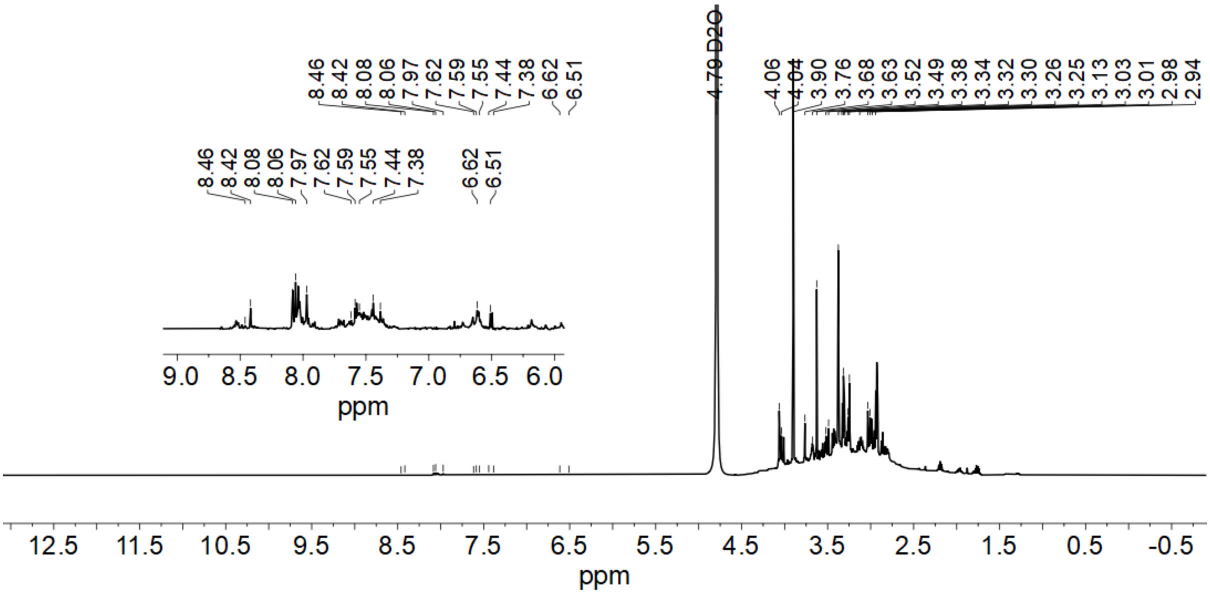

Figure S8 ^{1}H NMR (500 MHz, D_2O) spectrum obtained of mixture of products corresponding to Table S1; Entry 4.

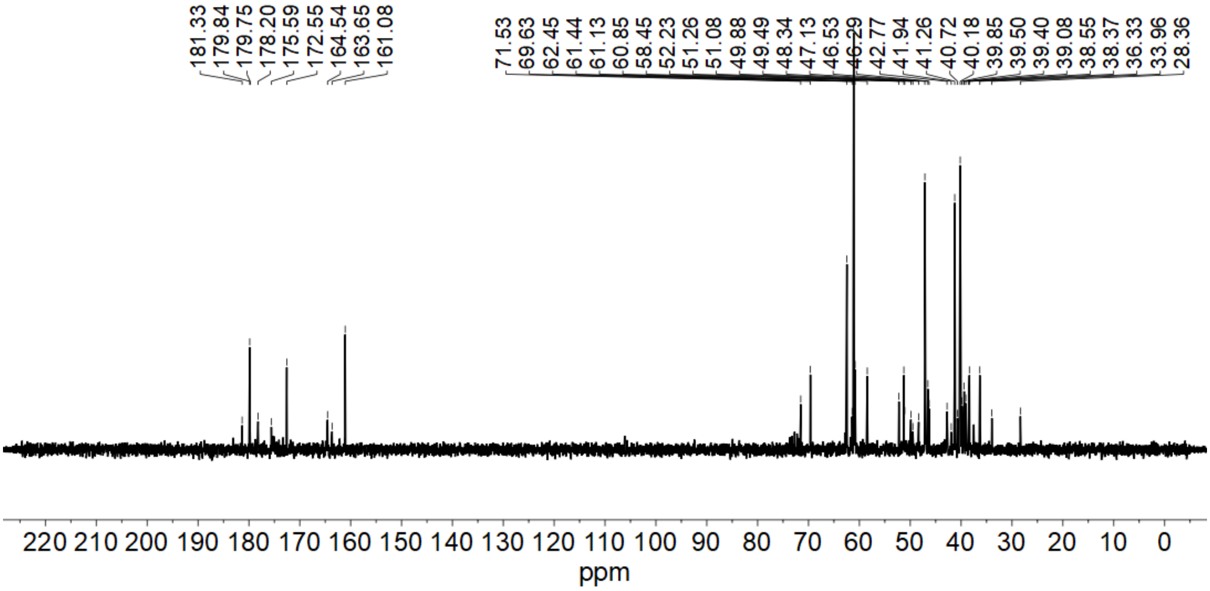

Figure S9 $^{13}C\{^{1}H\}$ NMR (126 MHz, D_2O) spectrum of product mixture corresponding to Table S1; Entry 4.

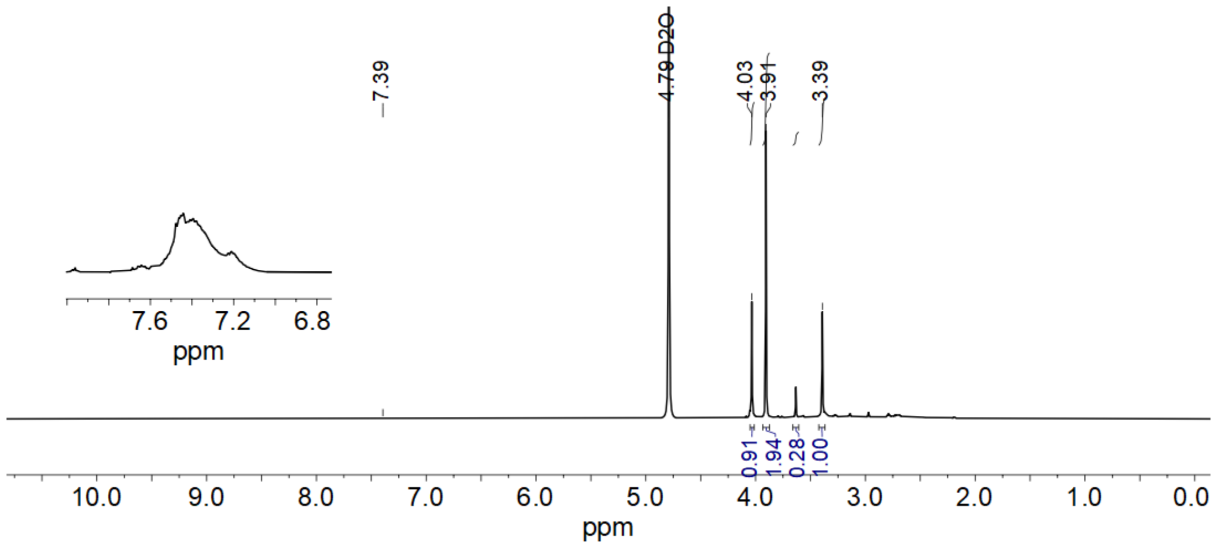

Figure S10 ^{1}H NMR (500 MHz, D_2O) spectrum obtained of mixture of products corresponding to Table S1; Entry 5.

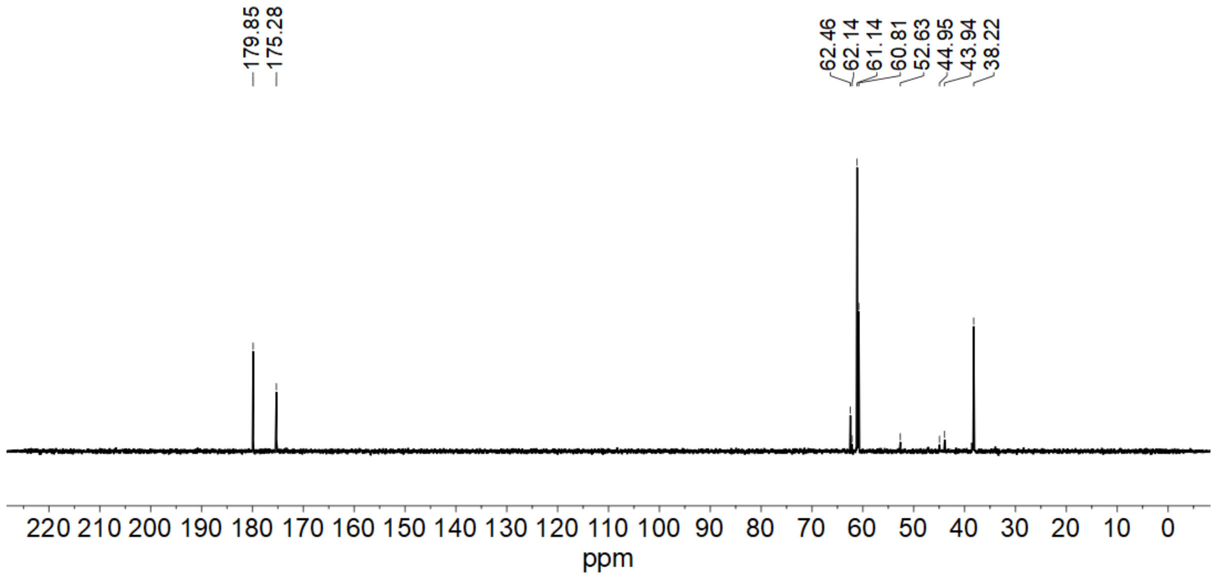

Figure S11 $^{13}C\{^{1}H\}$ NMR (126 MHz, D_2O) spectrum of product mixture corresponding to Table S1; Entry 5.

1.4. Condition Optimisation/Control Experiments

Table S2. Reaction optimisation for the dehydrogenative coupling of ethylene glycol and ethylene diamine with **1**.[a]

Entry	**1** /mol%	Base	Solvent	H_2 /mL	Products(s)	Yield /mg (%)[b]	Additional variable
1	1	K_2CO_3	THF	40	u-PEI-1, PEI-1, PA	42 (26)	-
2	1	K_2CO_3	THF	<5	u-PEI-1, PEI-1	50[e]	150 °C
3	1	K_2CO_3	THF	20	u-PEI-1, PEI-1, PA	22 (13)	48 h
4	1	KO^{t}Bu	THF	<5	u-PEI-1, PEI-1	49 (28)	-
5	2	K_2CO_3	THF	15	u-PEI-1, PEI-1	75 (44)	-
6	1	K_2CO_3	THF	15	u-PEI-1, PEI-1	110 (16)[f]	50 mol% base
7	1	KO^{t}Bu	THF	<5	u-PEI-1, PEI-1	55 (32)	4:1 [$C_2H_8N_2$]:[$C_2H_6O_2$]
8	1	KO^{t}Bu	THF	<5	PEI-1,	44 (26)	250 mL system
9	1	KO^{t}Bu	THF	<1	PEI-1	32 (18)	150 °C
10	1	K_2CO_3	Tol	<5	u-PEI-1, PEI-1	164 (95)	-
11	1	KO^{t}Bu	Tol	<1	PEI-1	160 (92)	150 °C
12	1	KO^{t}Bu	Tol	<5	PEI-1	116 (67)	1.0 M [$C_2H_6O_2$]
13	1	KO^{t}Bu	Tol	<5	PEI-1, i	113 (65)[e]	No solvent
14	1	KO^{t}Bu	Tol	<5	PEI-1, i	99 (57)	2 eq. H_2O added
15[c]	1	KO^{t}Bu	Tol	0	-	-	No diamine
16[d]	1	KO^{t}Bu	Tol	0	-	-	No diol
17	-	K_2CO_3	THF	0	-	-	-
18	1	-	THF	0	-	-	-
19	1	K_2CO_3	THF	0	-	-	Open conditions
20	1	KO^{t}Bu	THF	<1	-	<5%	120 °C

[a] Experimental conditions: 10 mol% base, 170 °C, 24 h, 2 mmol $C_2H_6O_2$ [0.5 M in THF or toluene], sealed 100 cm^3 system. [b] theoretical yield based on conversion to major product; [c] 4 mmol $C_2H_6O_2$; [d] 4 mmol $C_2H_8N_2$; [e] sample contains small quantities of residual ethylene glycol (determined by NMR spectrsocopies); [f] spectroscopic analysis shows sample contains significant residual K_2CO_3, yield quoted is corrected to omit mass from K_2CO_3.

Experimental data corresponding to samples using various conditions as outlined in Table S2 are reported below. In many cases, likely due to the mixture of products obtained, with high degrees of branching, the NMR spectra obtained were broad and poorly resolved: in such cases, only the key spectroscopic shifts are reported, although full spectra are shown in Section 1.4.2. All samples obtained were soluble in water.

Table S2; Entry 1:

^{1}H NMR (D_2O, 500 MHz): δ_H 8.42 (CONH), 8.03 (CONH or CH=N), 3.90 (s), 3.63 (br), 1.80 (s).

^{13}C{^{1}H} NMR (D_2O, 126 MHz, l.b. 10 Hz): δ_c 179.8 (C=O), 176.1 (C=O), 155.5 (<u>C</u>=N), 64.3, 61.1.

IR (ATR-FTIR, cm^{-1}): ν 3300brs (O-H/N-H stretch), 2934w (C-H), 1634m (C=N), 1577s (N-H).

TGA: T_d 245 °C

DSC: T_m 139.6 °C

<u>Table S2; Entry 2:</u>

From the NMR spectra obtained, there is likely residual ethylene glycol in the sample.

^{1}H NMR (D_2O, 500 MHz): δ_H 8.04 (C<u>H</u>=N), 5.46 (s), 3.81 (s), 3.71 (s), 3.25 (s), 2.90 (br m), 2.74 (br s).

^{13}C{^{1}H} NMR (D_2O, 126 MHz): δ_c 164.6 (C=N), 74.0, 67.8, 64.4, 44.8 44.3 24.9.

IR (ATR-FTIR, cm^{-1}): ν 3271m (N-H stretch), 2851m (C-H), 1655w (C=N), 1499s (C-H).

TGA: T_d 229 °C

DSC: T_m 169.5, 180.8 °C

<u>Table S2; Entry 3:</u>

^{1}H NMR (D_2O, 500 MHz): δ_H 8.42 (CON<u>H</u>), 8.04 (C<u>H</u>=N), 4.03, 3.90, 3.36, 2.74, [4.03 – 2.74 (br)], 1.88 (s).

^{13}C{^{1}H} NMR (D_2O, 126 MHz): δ_c 179.1 (<u>C</u>=O), 160.6 (<u>C</u>=N), 58.5, 47.1, 45.2 (br), 41.2 (br), 39.6 (br).

IR (ATR-FTIR, cm^{-1}): ν 3234brm (N-H/O-H), 2911w (C-H), 1647s (C=N), 1570s (N-H)

TGA: T_d 260 °C

DSC: T_m 182.8, 187.9 °C

<u>Table S2; Entry 4:</u>

^{1}H NMR (D_2O, 500 MHz): δ_H 6.55 (br s), 3.89, 3.67, 3.27, 2.75, [3.89 – 2.20 (br)].

Note: No signals were observed in the ^{13}C{^{1}H} NMR spectrum obtained for this sample despite reaching the solubility limit for the sample in D_2O and running for >1600 scans.

IR (ATR-FTIR, cm^{-1}): ν 3273m (N-H/O-H), 2922w (C-H), 1649m (C=N), 1577s (N-H)

TGA: T_d 269 °C

DSC: T_m Not observed below 200 °C

<u>Table S2; Entry 5:</u>

^{1}H NMR (D_2O, 500 MHz): δ_H 8.02 (br s, C<u>H</u>=N), 3.90, 3.58, 3.29, 3.13, 2.78 [3.90 – 2.20 (br)].

$^{13}C\{^1H\}$ NMR (D_2O, 126 MHz): δ_c 164.6 (<u>C</u>=N), 46.4 – 33.9 (br).

IR (ATR-FTIR, cm^{-1}): ν 3252brs (N-H/O-H), 2932m (C-H), 1655m (C=N), 1578s (N-H)

TGA: T_d 259 °C

DSC: T_m 180.9, 191.8 °C

<u>Table S2; Entry 6:</u>

^1H NMR (D_2O, 500 MHz): δ_H 8.03 (C<u>H</u>=N), 4.03, 3.89, 3.61, 3.34, 3.02, 2.65 (4.03 – 2.65 (br)].

$^{13}C\{^1H\}$ NMR (D_2O, 126 MHz, l.b. 10 Hz): δ_c very broad, poorly resolved signal between 50 – 30 ppm.

IR (ATR-FTIR, cm^{-1}): ν 3138w (N-H), 2924w (C-H), 1483 (C-H).

TGA: T_d 261 °C

DSC: T_m 139.4 °C

The spectroscopic data obtained show the presence of significant residual K_2CO_3. As such, GPC data for this sample was not collected.

<u>Table S2; Entry 7:</u>

^1H NMR (D_2O, 500 MHz): δ_H 8.03 (C<u>H</u>=N), 4.04, 3.67, 3.27, 2.92, 2.73, 2.18, [4.04 – 2.18 (br)].

$^{13}C\{^1H\}$ NMR (D_2O, 126 MHz, l.b. 10 Hz): δ_c very broad, poorly resolved signal between 50 – 30 ppm.

IR (ATR-FTIR, cm^{-1}): ν 3254m (N-H/O-H), 2922w (C-H), 1572s (N-H), 1394m (C-H)

TGA: T_d 254 °C

DSC: T_m 176.1, 181.8 °C

<u>Table S2; Entry 8:</u>

^1H NMR (D_2O, 500 MHz): δ_H 4.06 (s), 3.90 (s), 3.68 (s), 3.31, 2.71, 1.97 (s), 1.78 (s), 4.06 – 1.97 (br)].

$^{13}C\{^1H\}$ NMR (D_2O, 126 MHz): δ_c 69.6, 62.5, 61.1, 58.5, 43.6 (br), 36.3.

IR (ATR-FTIR, cm^{-1}): ν 3221m (N-H), 2922w (C-H), 1578s (N-H), 1396m (C-H).

TGA: T_d 240 °C

DSC: T_m 179.6, 191.6 °C

<u>Table S2; Entry 9:</u>

^1H NMR (D_2O, 500 MHz): δ_H 4.06 (s), 3.91 (s), 3.68 (s), 3.32 (s), 2.77 (br s), 2.19 (s) [4.06 – 2.19 (br)].

$^{13}C\{^1H\}$ **NMR** (D_2O, 126 MHz): δ_c 69.6, 61.4, 58.5, 50.1, 48.8, 36.4, 30.2.

IR (ATR-FTIR, cm^{-1}): ν 3196w (N-H), 2852w (C-H), 1570s (N-H), 1396m (C-H).

TGA: T_d 250 °C

Due to the small yield of sample obtained for this sample, independent DSC was not carried out, and DTA was collected alongside TGA.

DTA: T_m Not observed.

Table S2; Entry 10:

^{1}**H NMR** (D_2O, 500 MHz): δ_H 8.04 (C$\underline{H}$=N), 3.62, 3.48, 3.37, 3.02, 2.75, 2.68 , 2.33 [3.62 – 2.33 (br)].

$^{13}C\{^1H\}$ **NMR** (D_2O, 126 MHz): δ_c 164.6 ($\underline{C}$=N), 77.01, 62.4, 57.0, 49.5, 44.3, 42.8.

IR (ATR-FTIR, cm^{-1}): ν 3251m (N-H/O-H), 2933m (C-H), 1649m (C=N), 1564s (N-H), 1443m (C-H).

TGA: T_d 238 °C

DSC: T_m 166.5 °C

Table S2; Entry 11:

^{1}**H NMR** (D_2O, 500 MHz): δ_H 4.07 (br), 3.90 (br), 3.69 (br), 3.31 (s), 3.31 (br) 2.71 (br s). [4.07 – 2.13 (br)]

$^{13}C\{^1H\}$ **NMR** (D_2O, 126 MHz): δ_c 71.5, 69.6, 62.4, 58.5, 48.7, 42.2, 40.6, 36.3.

IR (ATR-FTIR, cm^{-1}): ν 3259m (N-H), 2910m (C-H), 1577s (N-H), 1458m (C-H), 1313m (C-H).

TGA: T_d 235 °C

DSC: T_m 189.8 °C

Table S2; Entry 12:

^{1}**H NMR** (D_2O, 500 MHz): δ_H 4.06, 3.91, 3.68, 3.32, 2.75, 2.16, [4.06 – 2.16 (br)].

$^{13}C\{^1H\}$ **NMR** (D_2O, 126 MHz): δ_c 71.5, 62.5, 58.5, 45.0, 41.4, 39.7 (br), 25.1, 21.6, 14.7.

IR (ATR-FTIR, cm^{-1}): ν 3269m (N-H), 2926m (C-H), 1571s (N-H), 1429m (C-H), 1311m (C-H).

TGA: T_d 238 °C

DSC: T_m 176.0 °C

Table S2; Entry 13:

From the NMR spectra obtained, there is likely residual ethylene glycol in the sample.

^{1}H NMR (D_2O, 500 MHz): δ_H 3.36, 3.14, 2.83, 2.77, 2.55, 2.33, [3.90 – 2.33 (br)].

$^{13}C\{^1H\}$ NMR (D_2O, 126 MHz): δ_c 58.1, 42.6 (br), 25.1, 14.7.

IR (ATR-FTIR, cm^{-1}): ν 3265m (N-H), 2926m (C-H), 1654m (C=N), 1577s (N-H), 1436m (C-H), 1325m (C-H).

TGA: T_d 237 °C

DSC: T_m 151.4 °C

Table S2; Entry 14:

^{1}H NMR (D_2O, 500 MHz): δ_H 3.90, 3.57, 3.32, 3.27, 3.20, 3.13, 2.75, 2.73, [3.90 – 2.64 (br, overlapping m)].

$^{13}C\{^1H\}$ NMR (D_2O, 126 MHz): δ_c 61.1, 42.4, 39.7 (br), 37.7 (br), 34.2 (br).

IR (ATR-FTIR, cm^{-1}): ν 3230m (N-H), 2918m (C-H), 1654m (C=N), 1577s (N-H), 1436m (C-H), 1325m (C-H).

TGA: T_d 237 °C

DSC: T_m Not observed below 200 °C

Table S2; Entry 20:

When the reaction is carried out at 120 °C, <5% conversion is observed, with the major species observed by ^{1}H and $^{13}C\{^1H\}$ NMR being ethylene glycol (ethylene diamine is removed under reduced pressure during work up).

1.4.1 Infrared Spectra

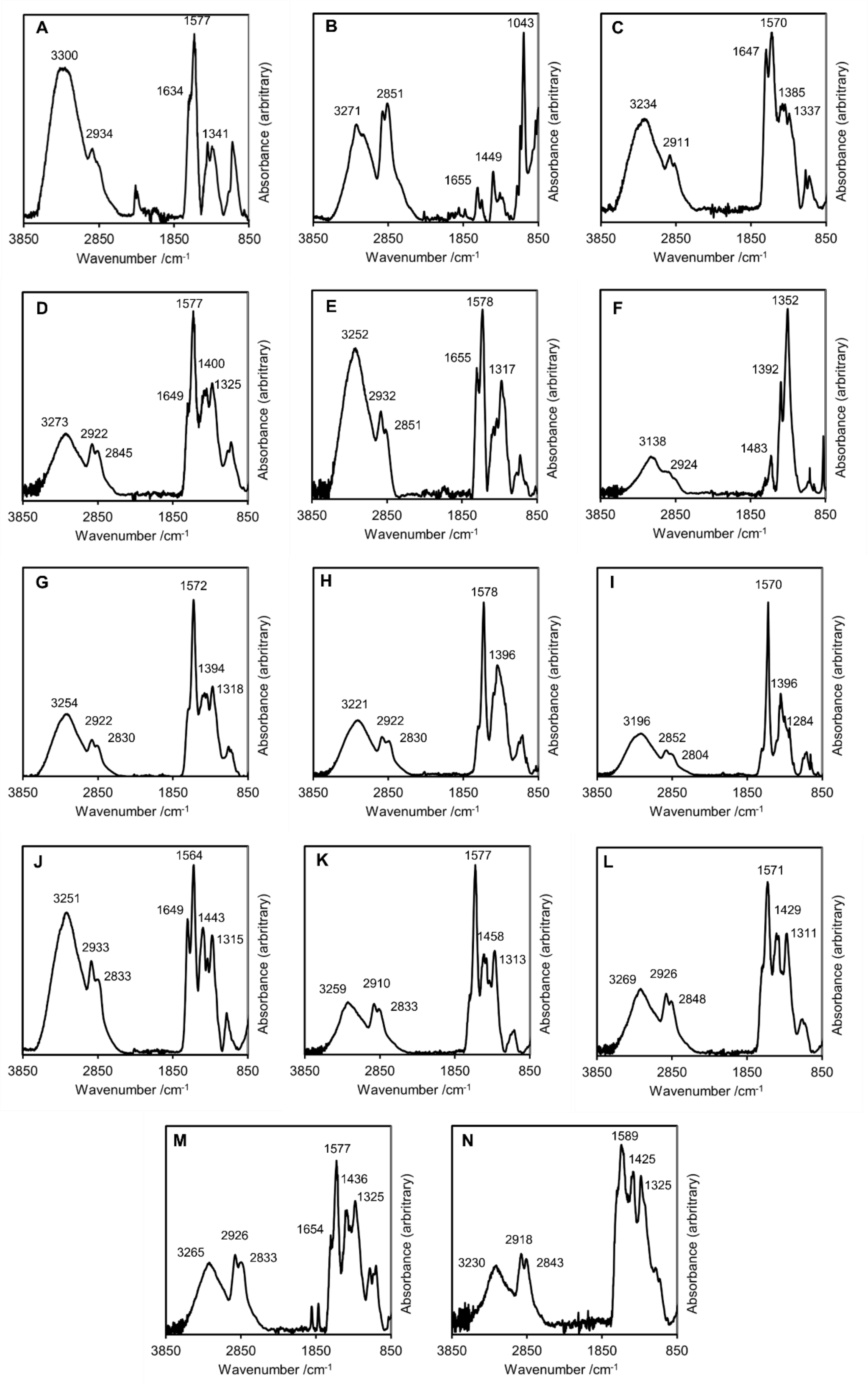

Figure S12 Infrared spectra (ATR-FTIR) for products of the dehydrogenative coupling of ethylene glycol and ethylene diamine. Where **A**) Corresponds to Table S2: Entry 1; **B**) corresponds to Table S2: Entry 2, **C**) corresponds to Table S2: Entry 3, *etc*.

1.4.2 TGA

Thermal degradation plots are shown below. The decomposition temperature (T_d) is taken as 5% mass loss after solvent loss. Derivative traces (smoothed with 20 pt. moving average) are also included.

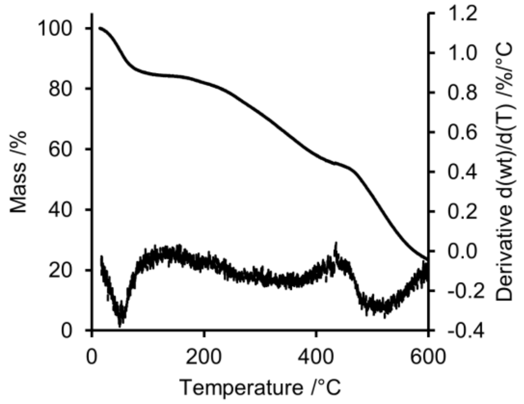

Figure S13 Mass loss as a function of temperature for sample corresponding to Table S2; Entry 1.

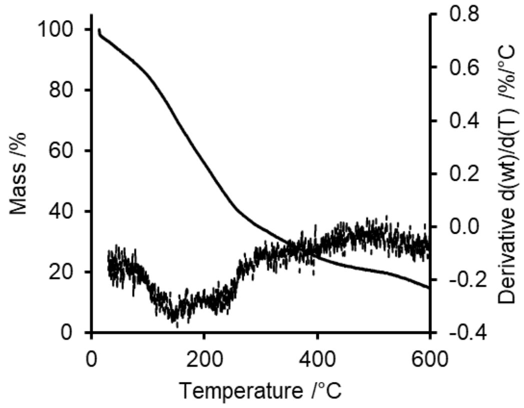

Figure S14 Mass loss as a function of temperature for sample corresponding to Table S2; Entry 2.

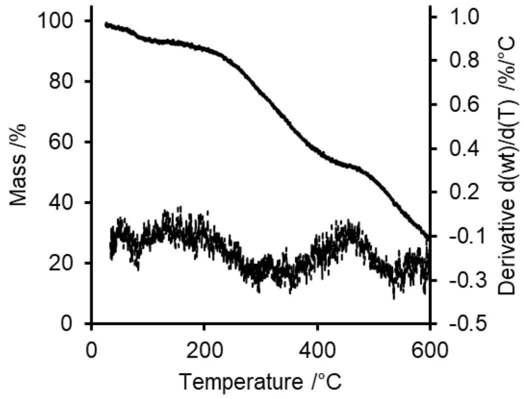

Figure 15 Mass loss as a function of temperature for sample corresponding to Table S2; Entry 3.

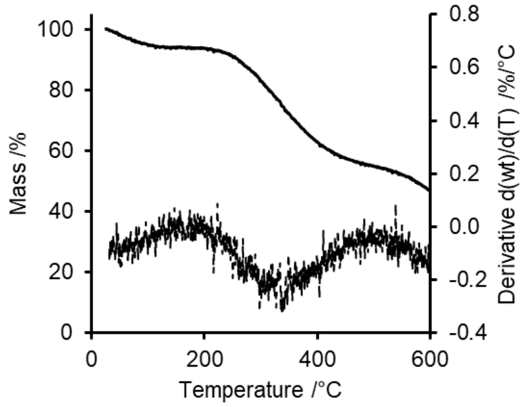

Figure S16 Mass loss as a function of temperature for sample corresponding to Table S2; Entry 4.

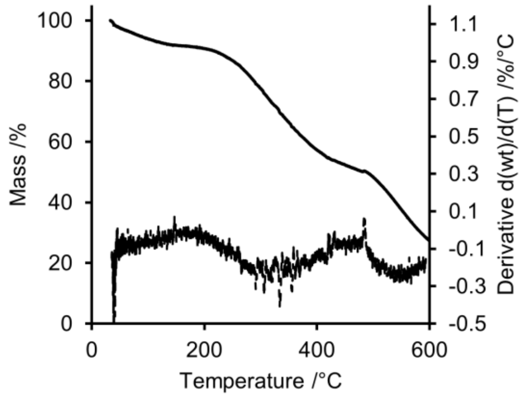

Figure S17 Mass loss as a function of temperature for sample corresponding to Table S2; Entry 5.

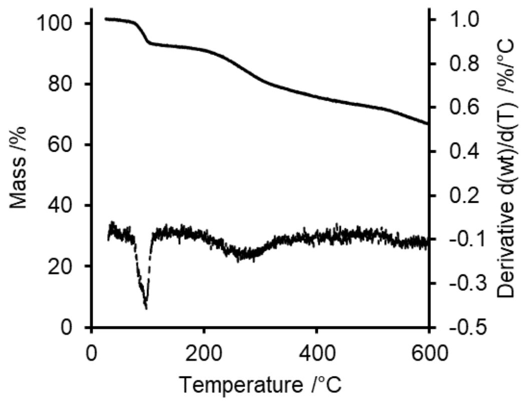

Figure S18 Mass loss as a function of temperature for sample corresponding to Table S2; Entry 6.

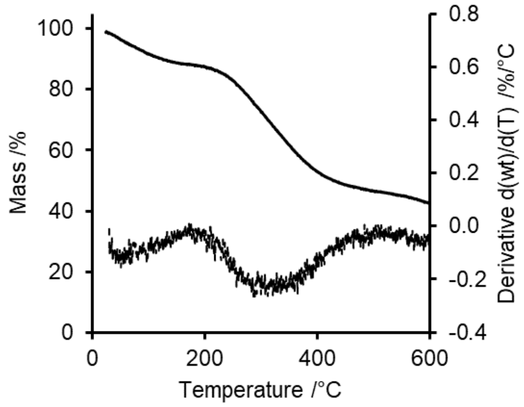

Figure S19 Mass loss as a function of temperature for sample corresponding to Table S2; Entry 7.

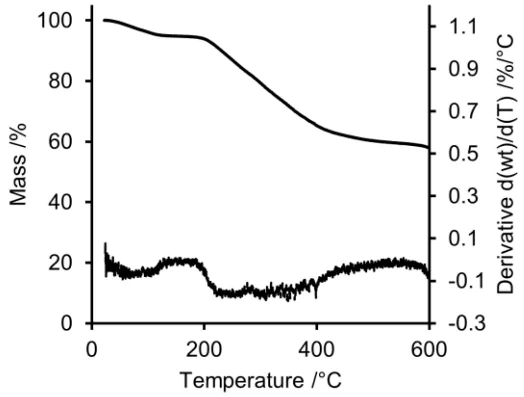

Figure S20 Mass loss as a function of temperature for sample corresponding to Table S2; Entry 8.

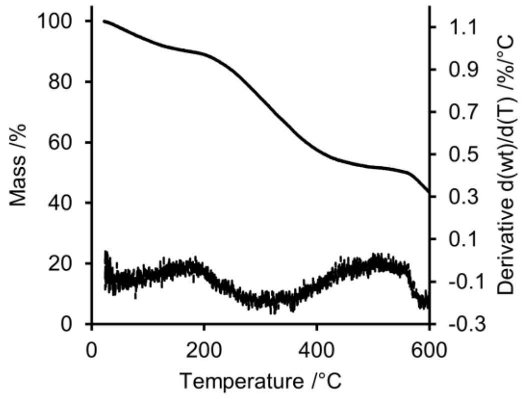

Figure S21 Mass loss as a function of temperature for sample corresponding to Table S2; Entry 9.

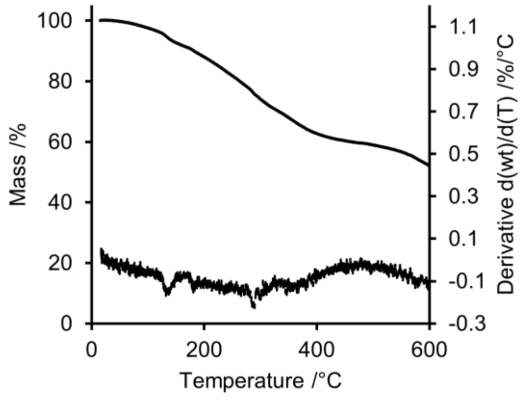

Figure S22 Mass loss as a function of temperature for sample corresponding to Table S2; Entry 10.

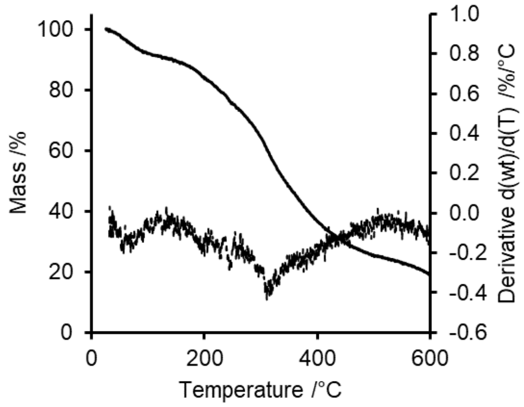

Figure S23 Mass loss as a function of temperature for sample corresponding to Table S2; Entry 11.

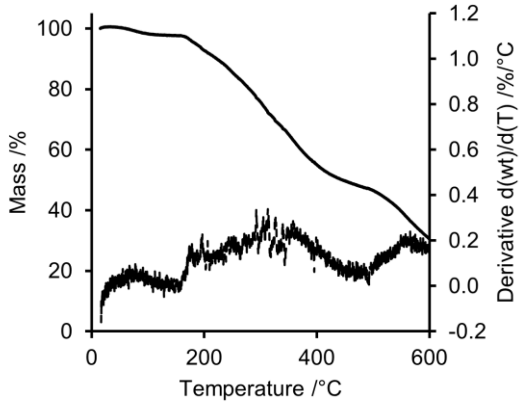

Figure S24 Mass loss as a function of temperature for sample corresponding to Table S2; Entry 12.

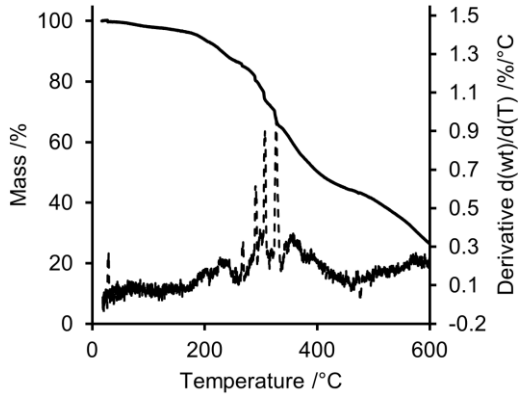

Figure 25 Mass loss as a function of temperature for sample corresponding to Table S2; Entry 13.

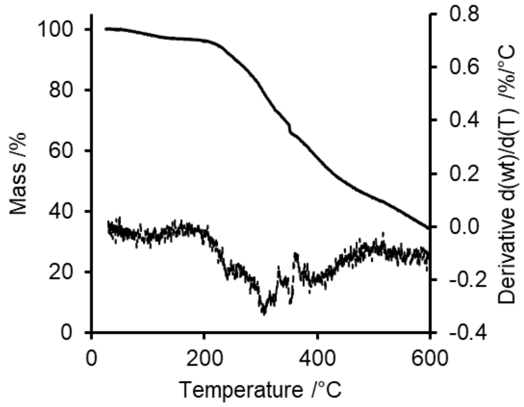

Figure S26 Mass loss as a function of temperature for sample corresponding to Table S2; Entry 14.

1.4.3 DSC

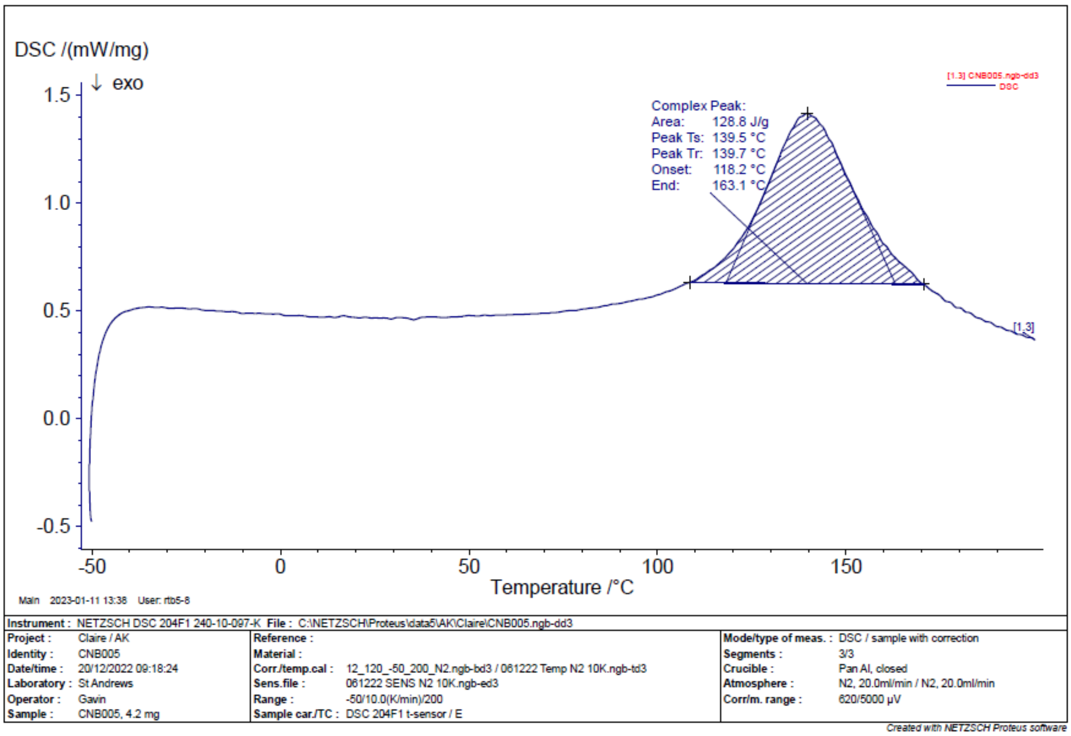

Figure S27 DSC trace corresponding to sample Table S2; Entry 1.

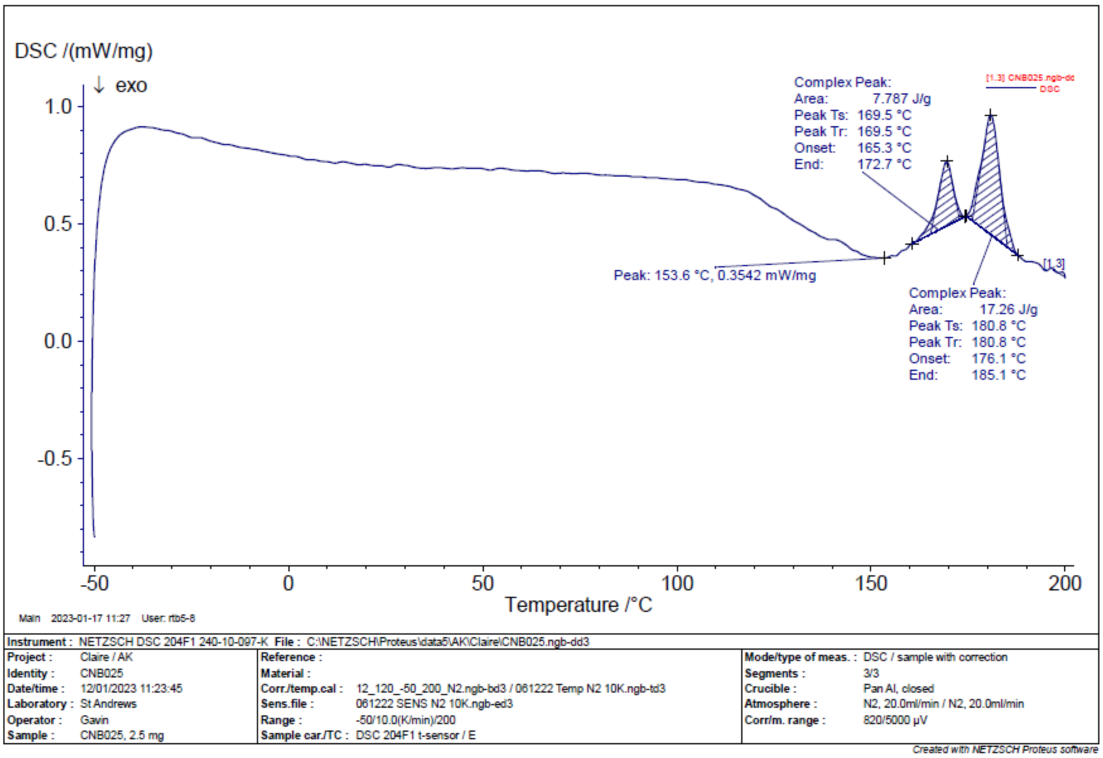

Figure S28 DSC trace corresponding to sample Table S2; Entry 2. Note, apparent endothermic event with peak 153.6 °C is likely the result of residual K_2CO_3 present within the product mixture – vide infra.

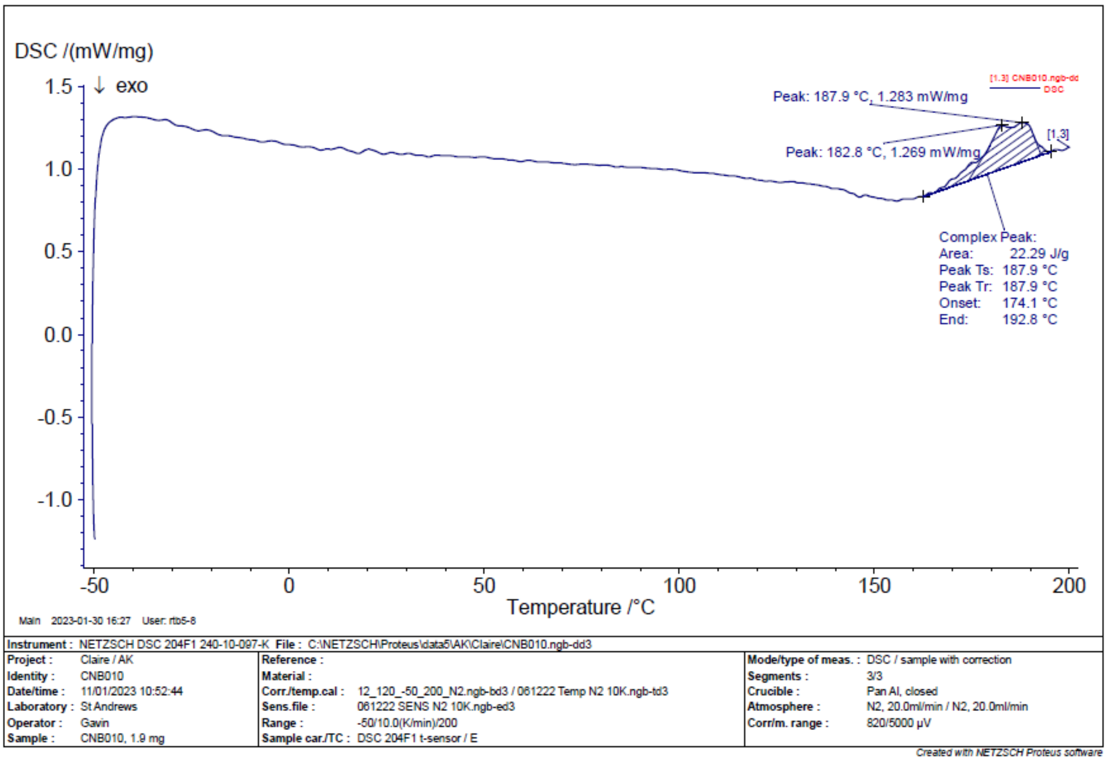

Figure S29 DSC trace corresponding to Table S2; Entry 3. Note, apparent endothermic event with peak 155.8°C is likely the result of residual K_2CO_3 present within the product mixture – vide infra.

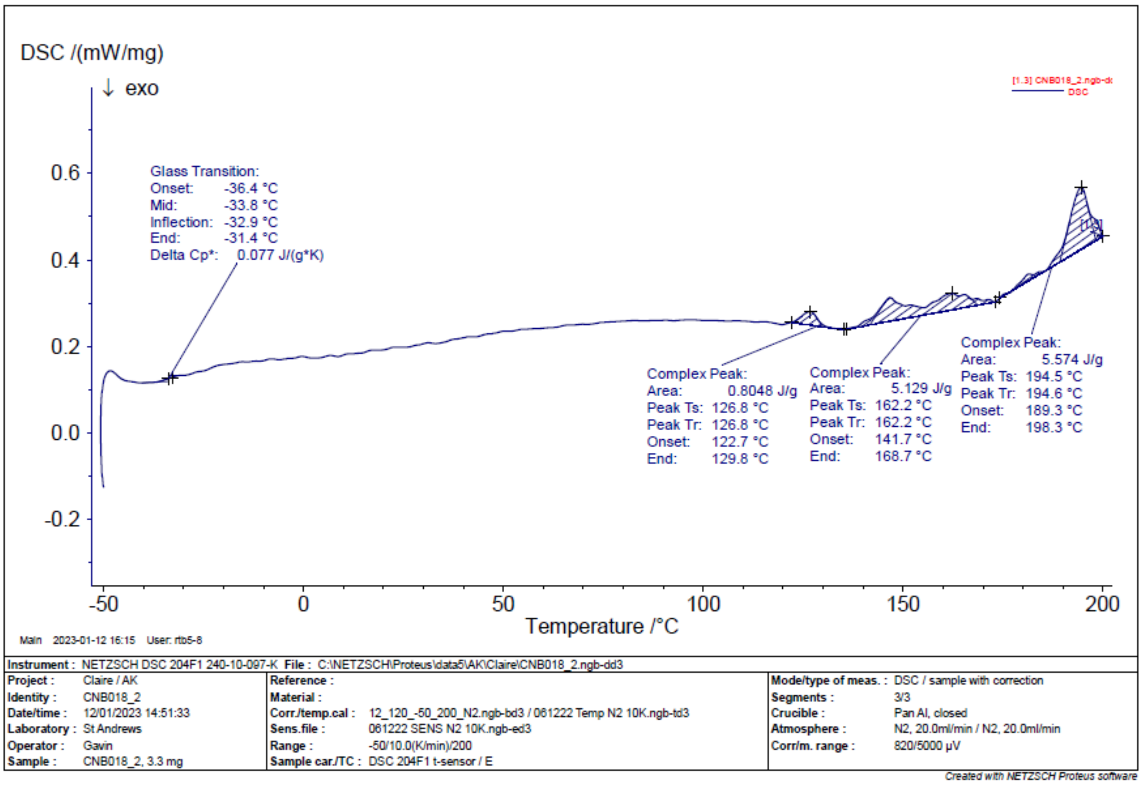

Figure S30 DSC trace corresponding to Table S2; Entry 4.

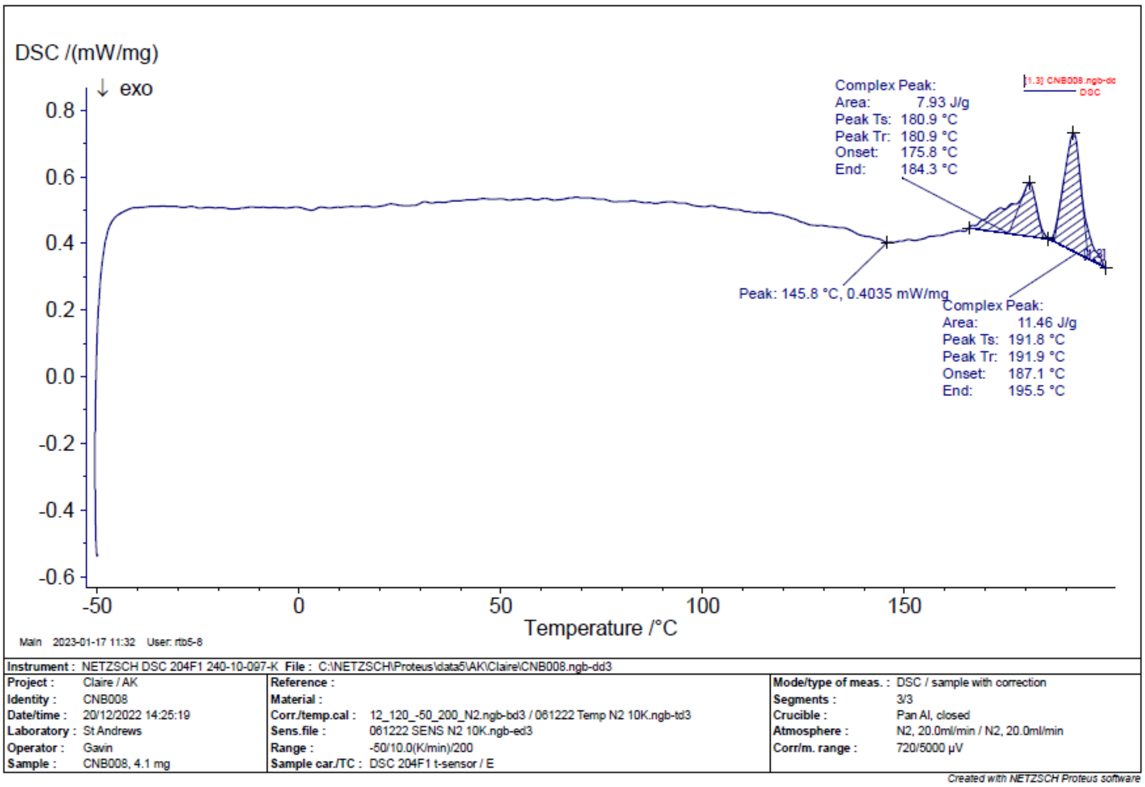

Figure S31 DSC trace corresponding to Table S2; Entry 5. Note, apparent endothermic event with peak ~150 °C is likely the result of residual K_2CO_3 present within the product mixture – vide infra.

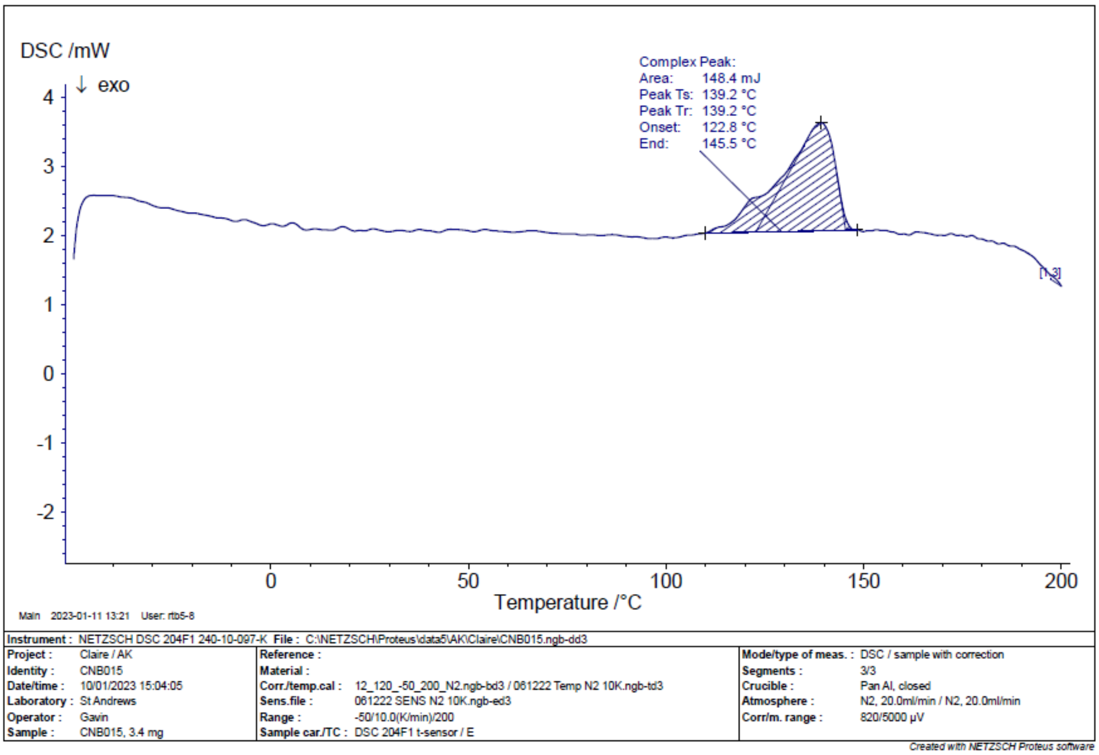

Figure S32 DSC trace corresponding to Table S2; Entry 6.

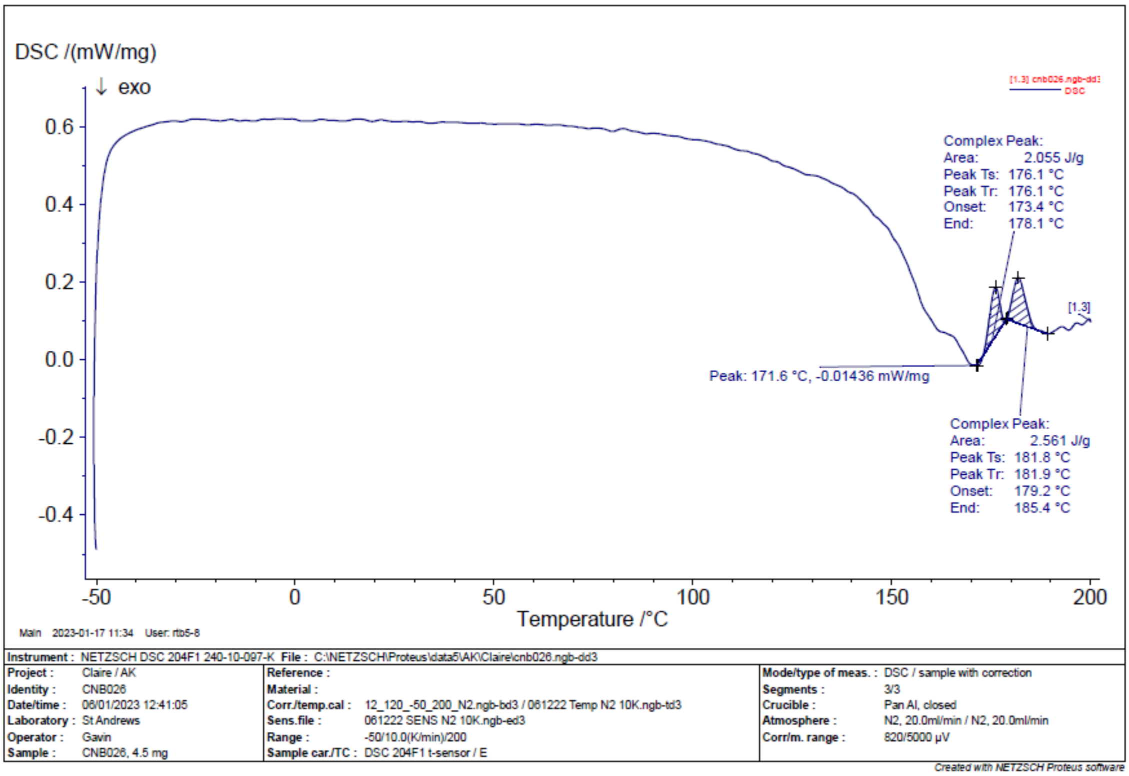

Figure S33 DSC trace corresponding to Table S2; Entry 7. Note, apparent endothermic event with peak 171.6°C is likely the result of residual KO^{t}Bu present within the product mixture – vide infra.

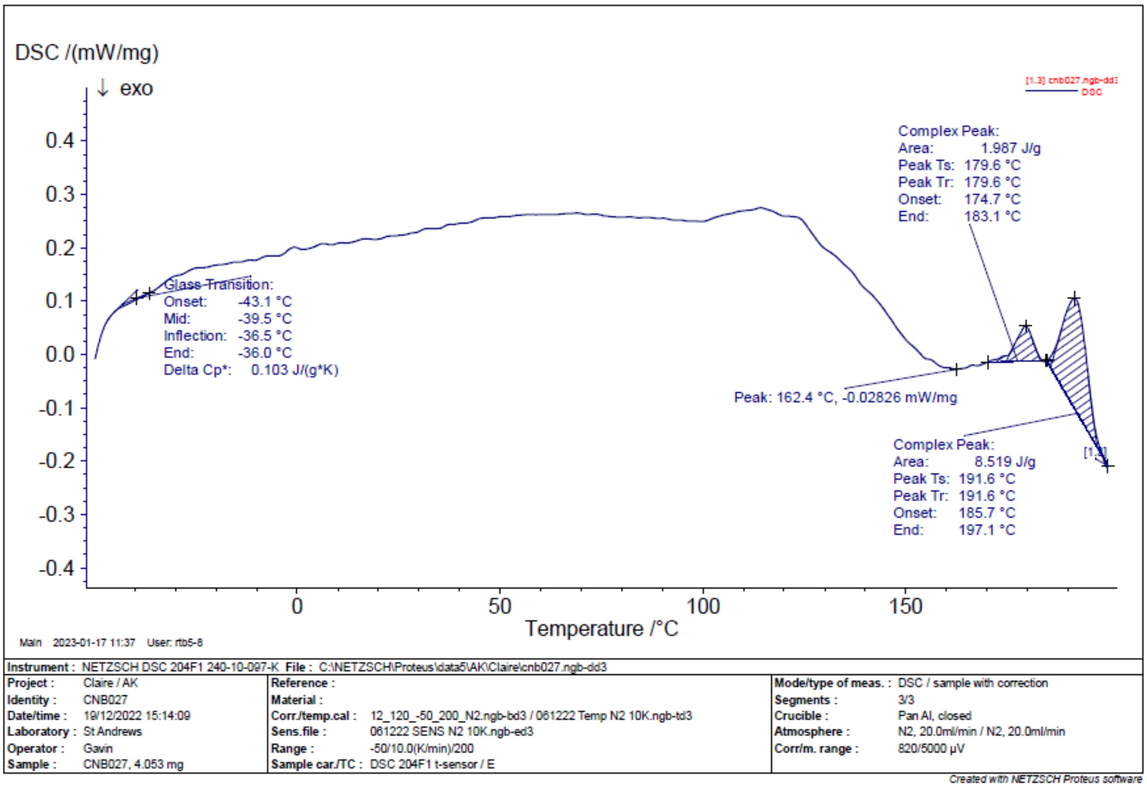

Figure S34 DSC trace corresponding to Table S2; Entry 8.

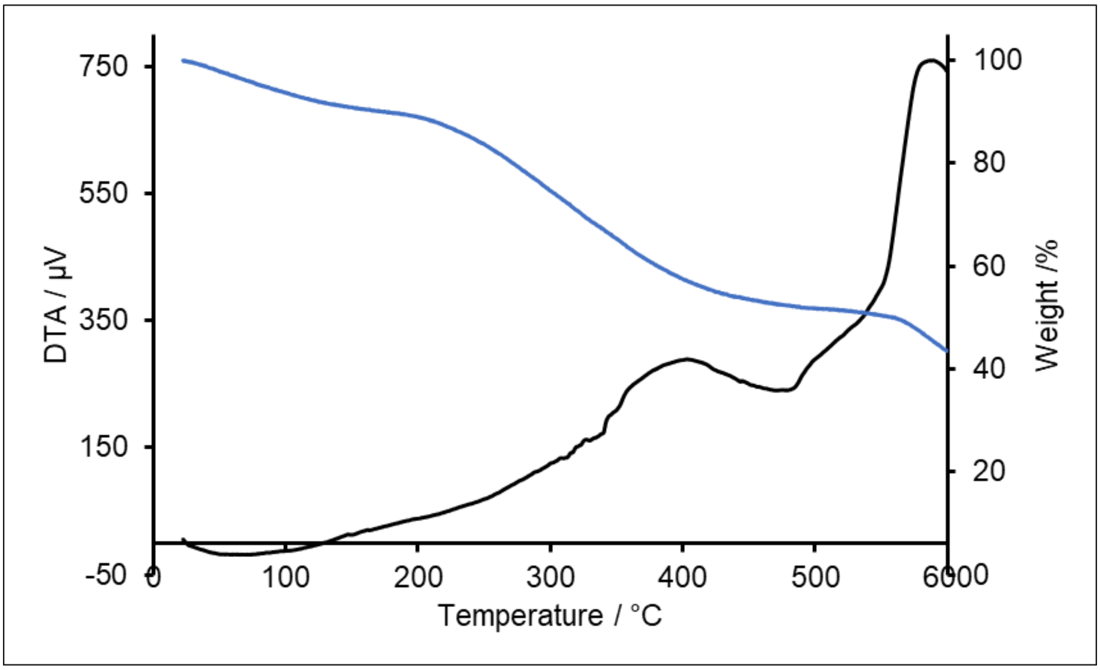

Figure S35 DTA/TGA trace corresponding to Table S2; Entry 9. Due to the small yield of sample obtained for this sample, independent DSC could not be carried out, and DTA was collected alongside TGA.

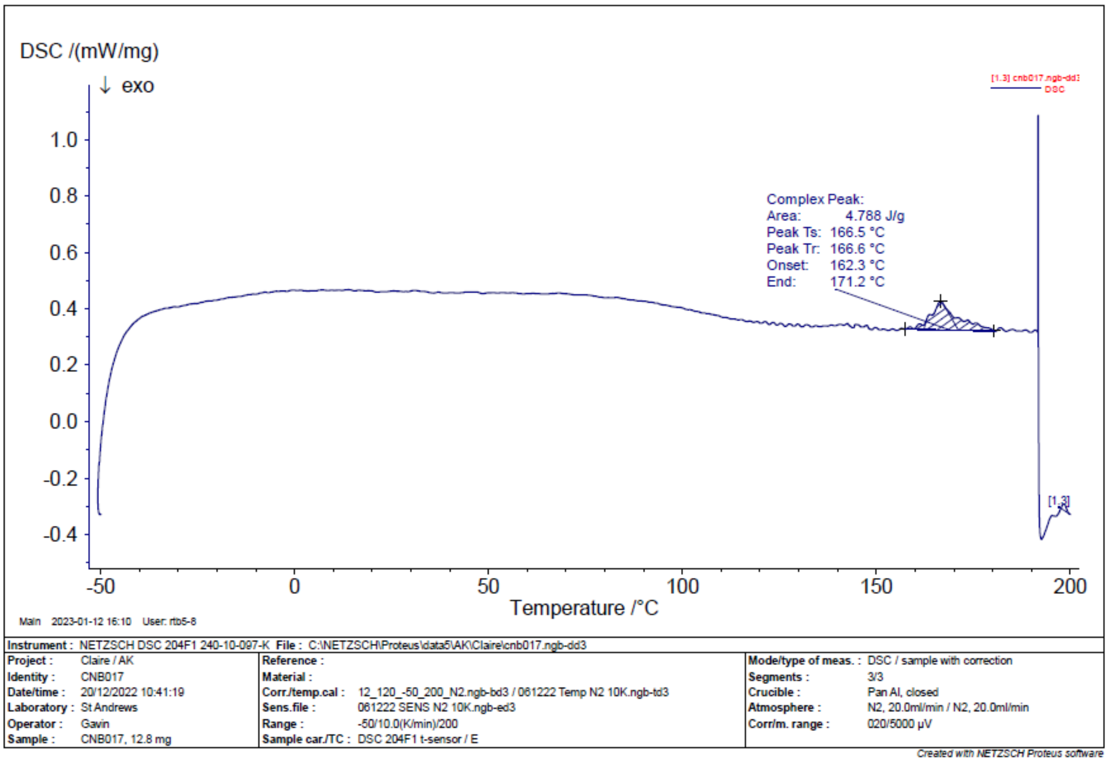

Figure S36 DSC trace corresponding to Table S2; Entry 10.

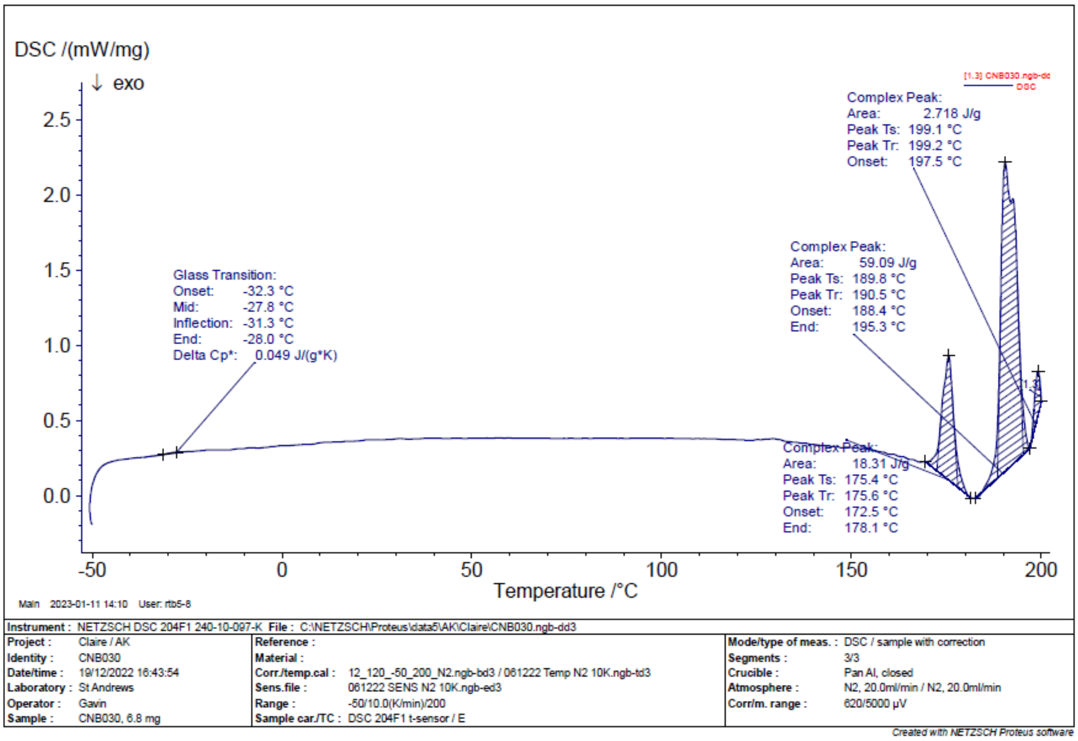

Figure S37 DSC trace corresponding to Table S2; Entry 11. Note, exotherm with peak 199.1 is likely the result of residual ethylene glycol (b.p. 197 °C)[3] present in the sample. Apparent endothermic event with peak 175.4°C is likely the result of residual KO^{t}Bu present within the product mixture – vide infra.

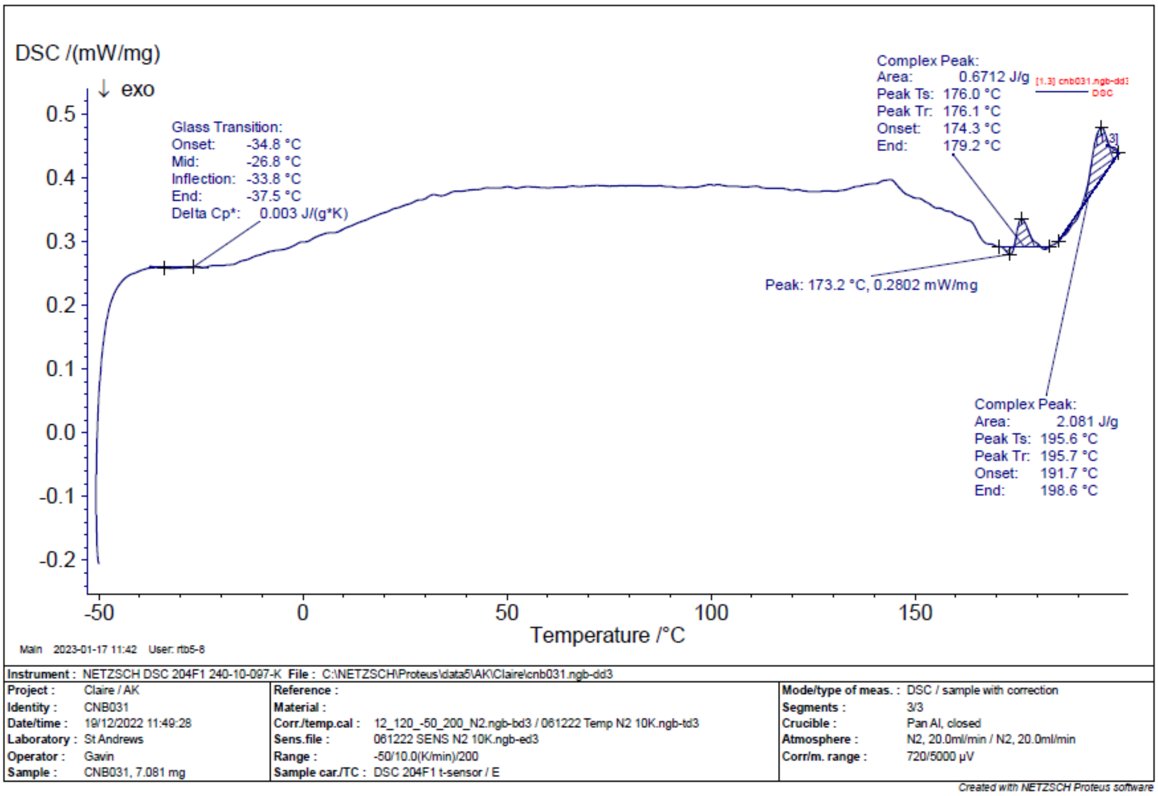

Figure S38 DSC trace corresponding to Table S2; Entry 12. Note, apparent endothermic event at 173.2°C is likely the result of residual KO^{t}Bu present within the product mixture – vide infra.

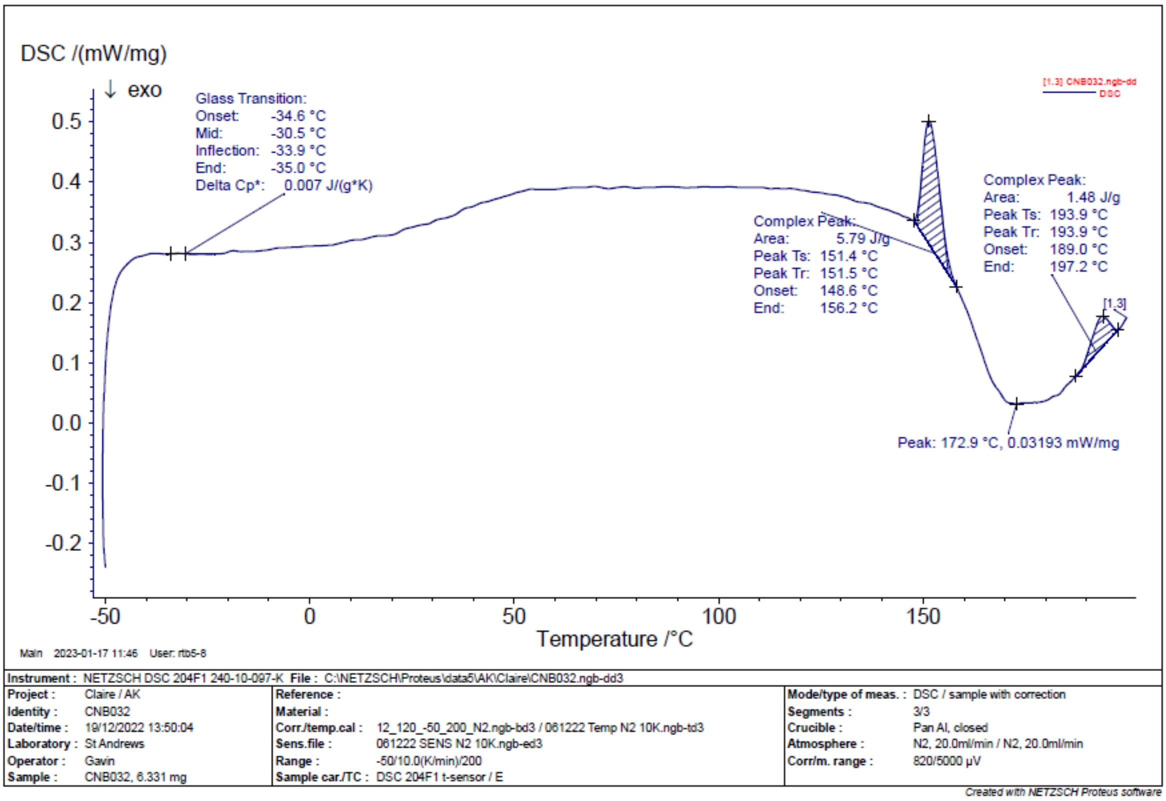

Figure S39 DSC trace corresponding to Table S2; Entry 13. Note, apparent endothermic event with peak at 172.9 °C is likely the result of residual KO^{t}Bu present within the product mixture – vide infra.

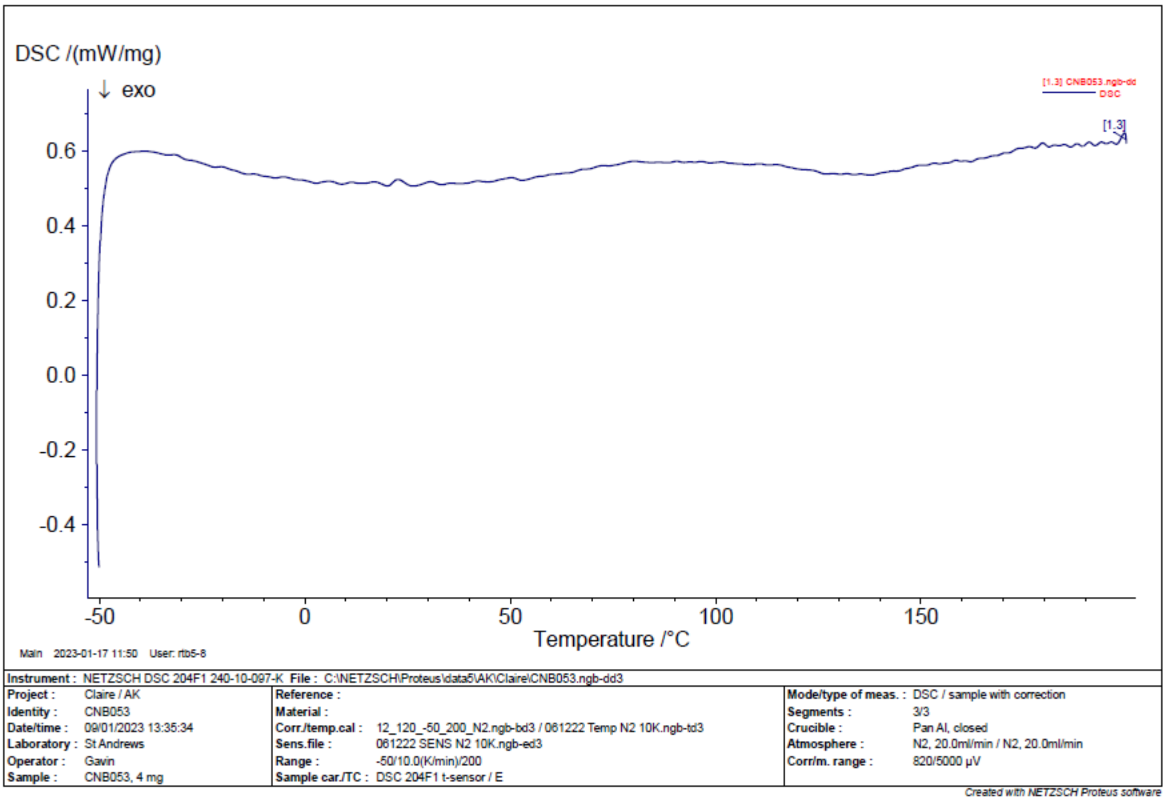

Figure S40 DSC trace corresponding to Table S2; Entry 14.

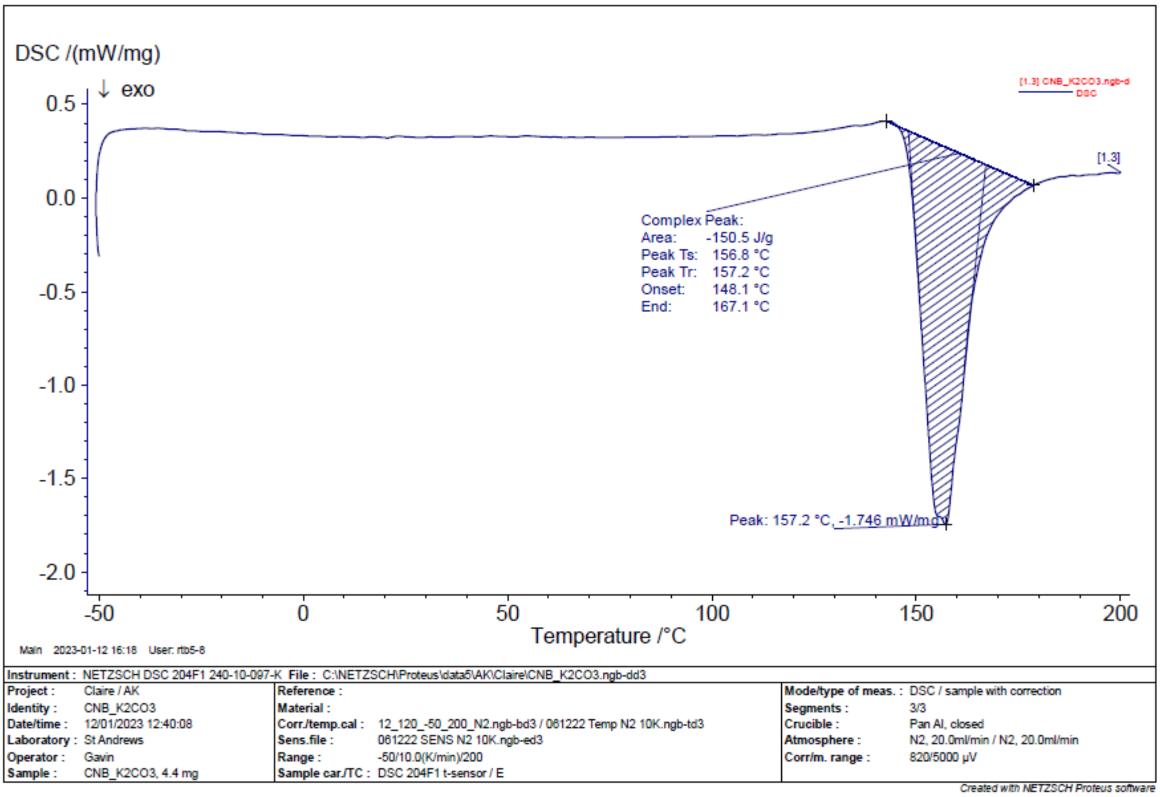

Figure S41 DSC trace corresponding to K_2CO_3 under our conditions.

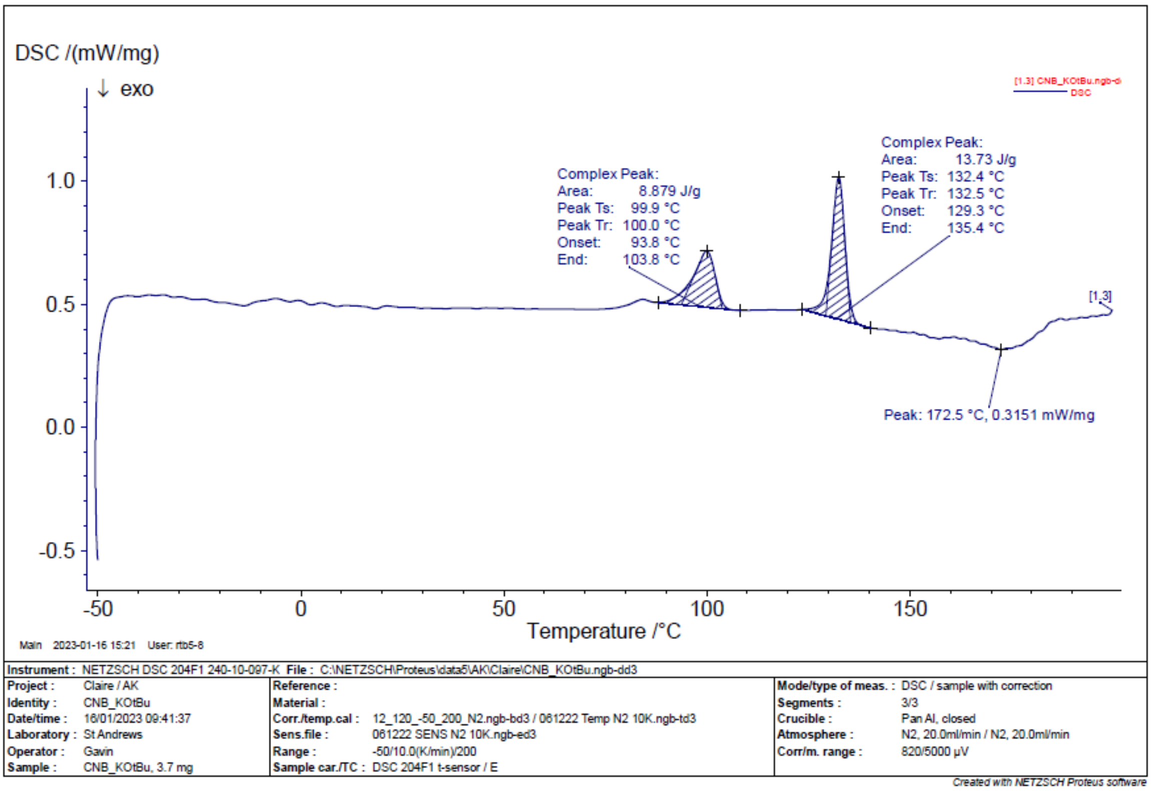

Figure S42 DSC trace corresponding to KO^{t}Bu under our conditions.

1.4.4 NMR Spectra

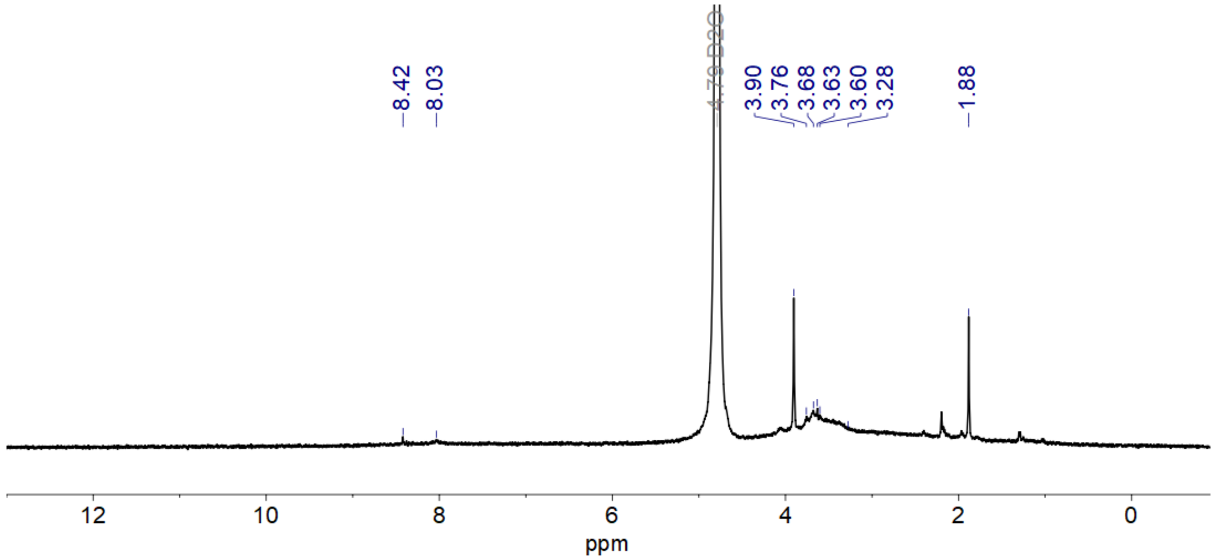

Figure S43 ^{1}H NMR (500 MHz, D_2O) spectrum corresponding to Table S2; Entry 1.

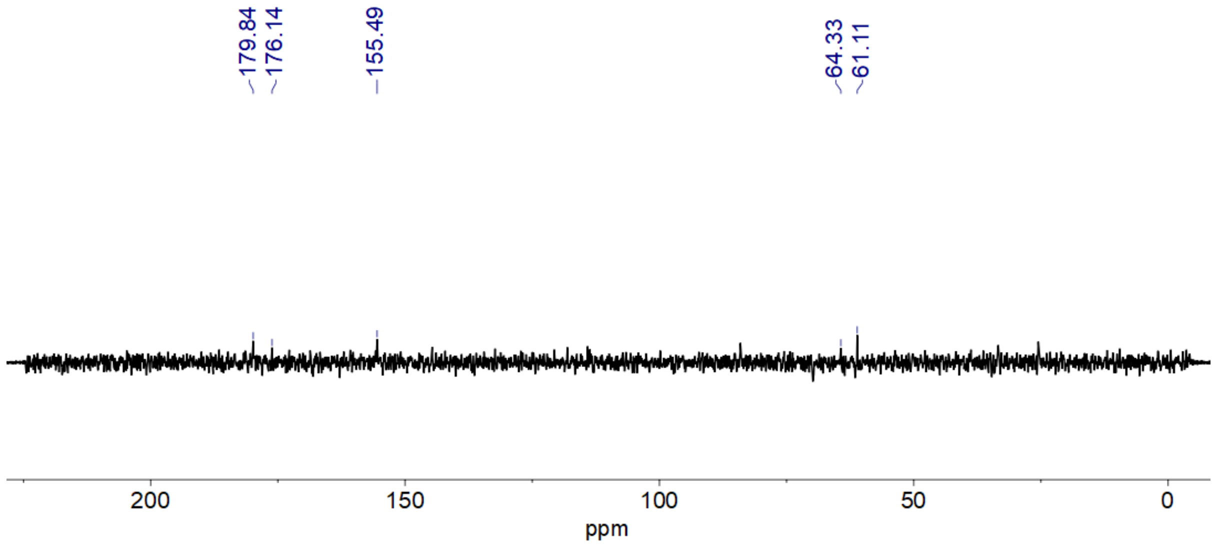

Figure S44 $^{13}C\{^{1}H\}$ NMR (126 MHz, D_2O, l.b. 10 Hz) spectrum corresponding to Table S2; Entry 1.

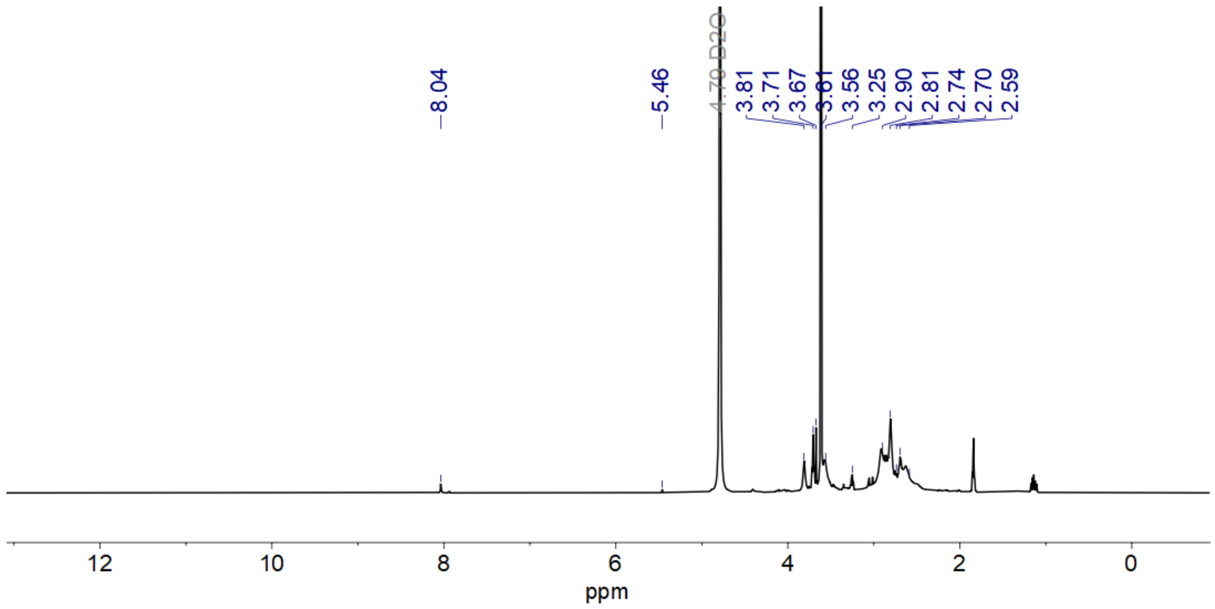

Figure S45 ^{1}H NMR (500 MHz, D_2O) spectrum corresponding to Table S2; Entry 2.

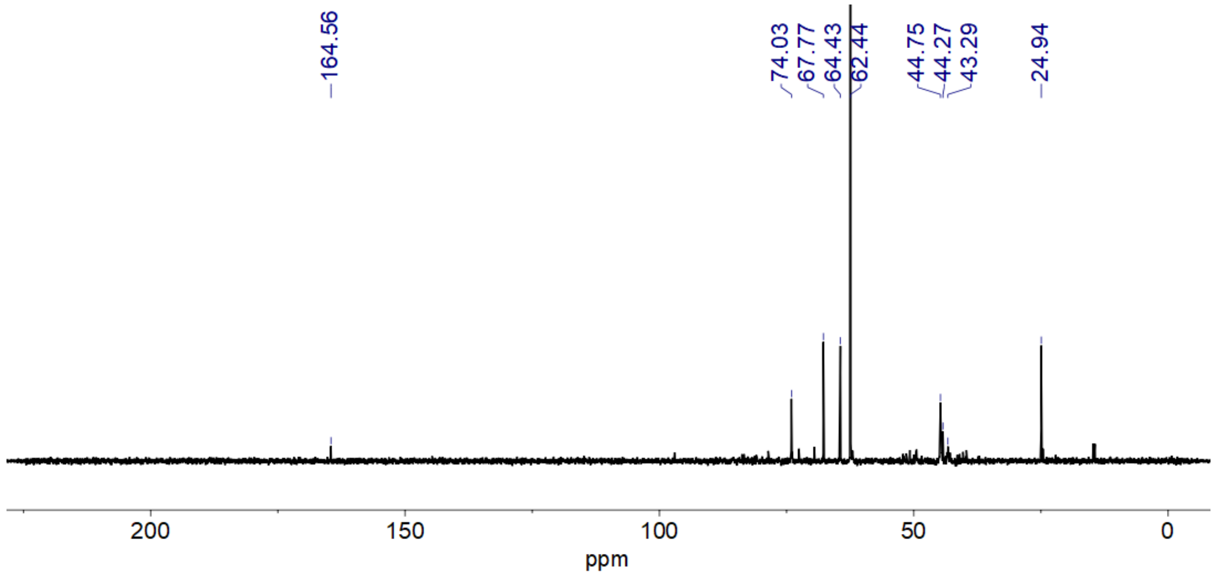

Figure S46 $^{13}C\{^{1}H\}$ NMR (126 MHz, D_2O) spectrum corresponding to Table S2; Entry 2.

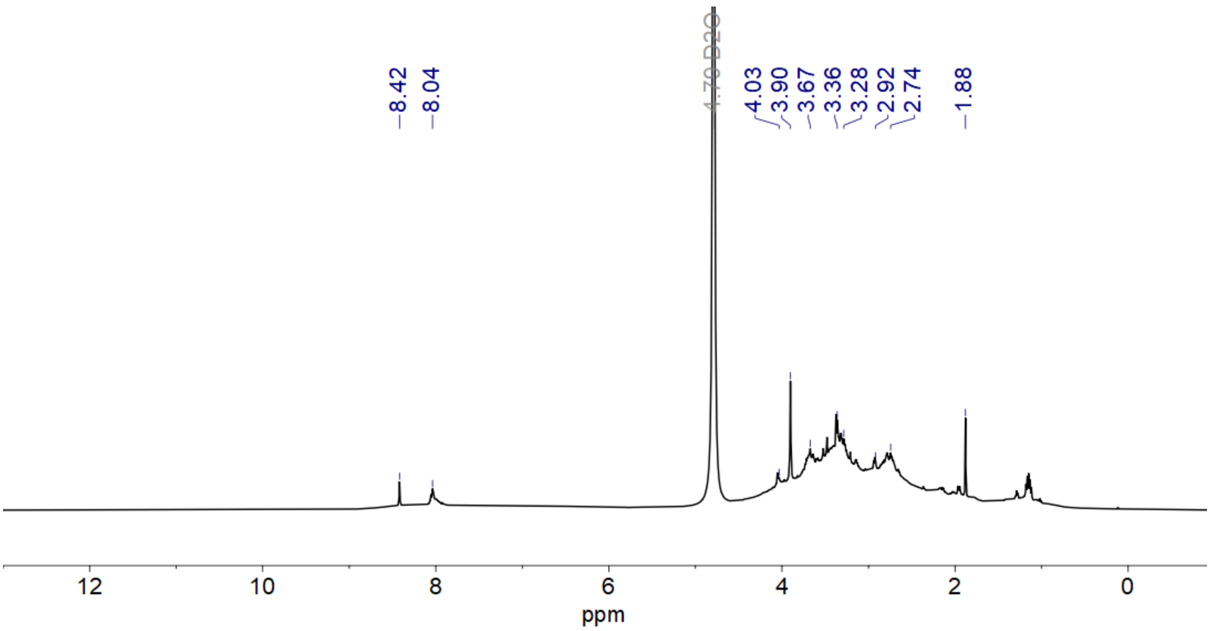

Figure S47 ^{1}H NMR (500 MHz, D_2O) spectrum corresponding to Table S2; Entry 3.

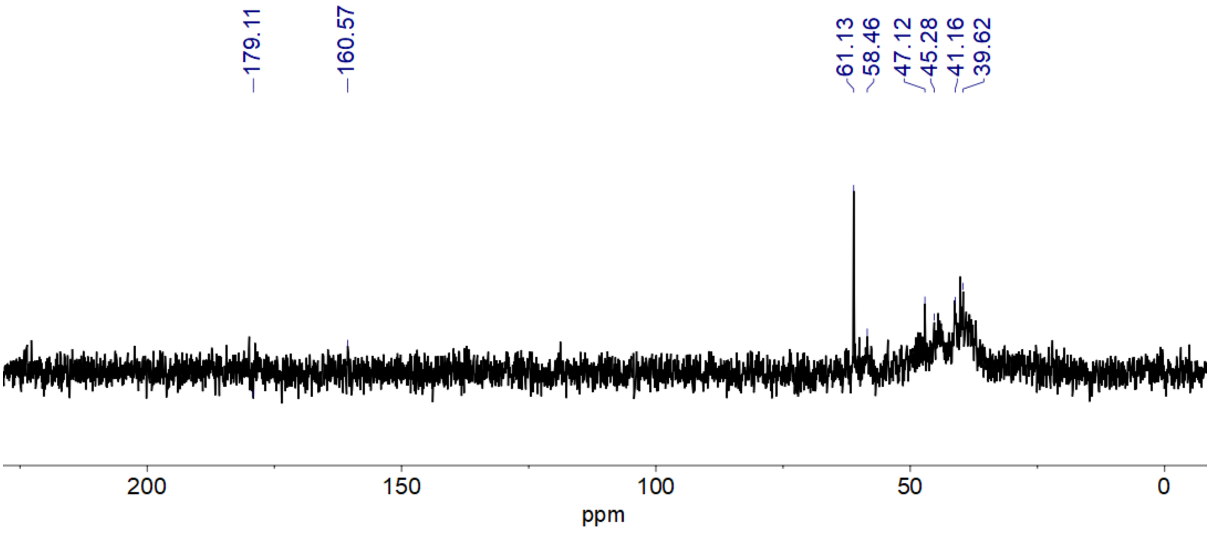

Figure S48 $^{13}C\{^{1}H\}$ NMR (126 MHz, D_2O, l.b. 5 Hz) spectrum corresponding to Table S2; Entry 3.

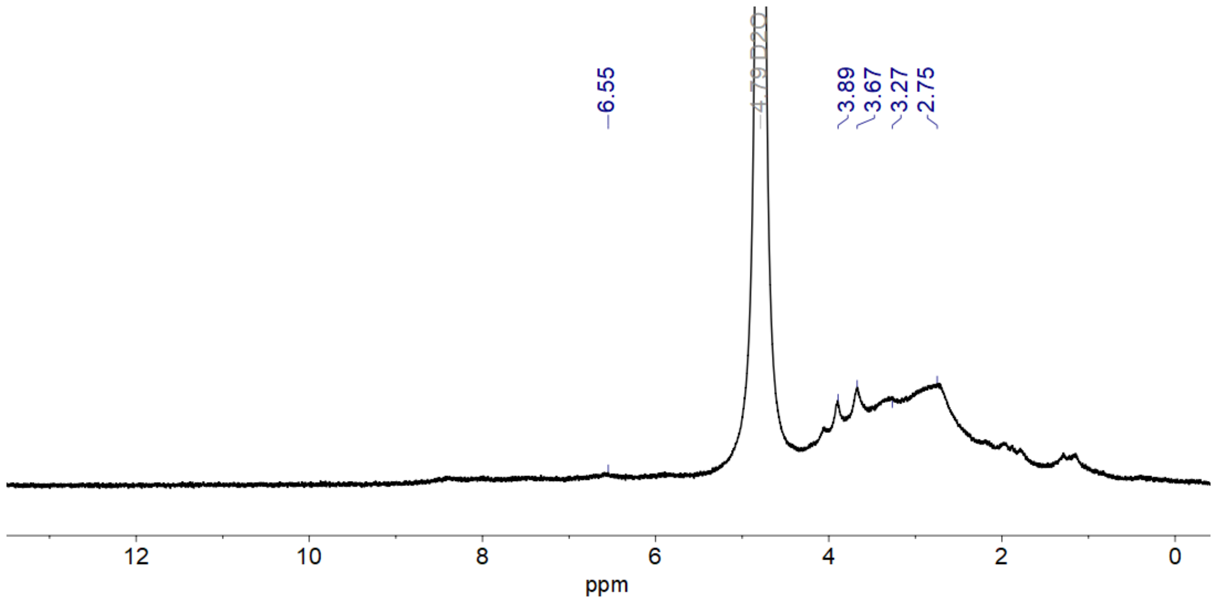

Figure S49 ^{1}H NMR (500 MHz, D_2O) spectrum corresponding to Table S2; Entry 4.

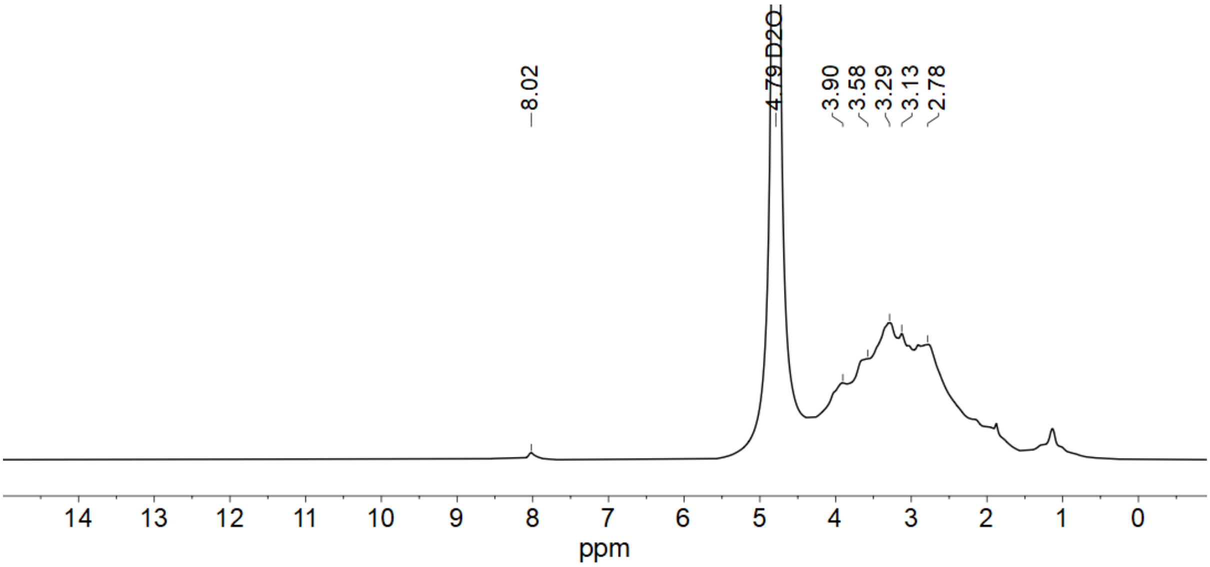

Figure S50 ^{1}H NMR (500 MHz, D_2O) spectrum corresponding to Table S2; Entry 5.

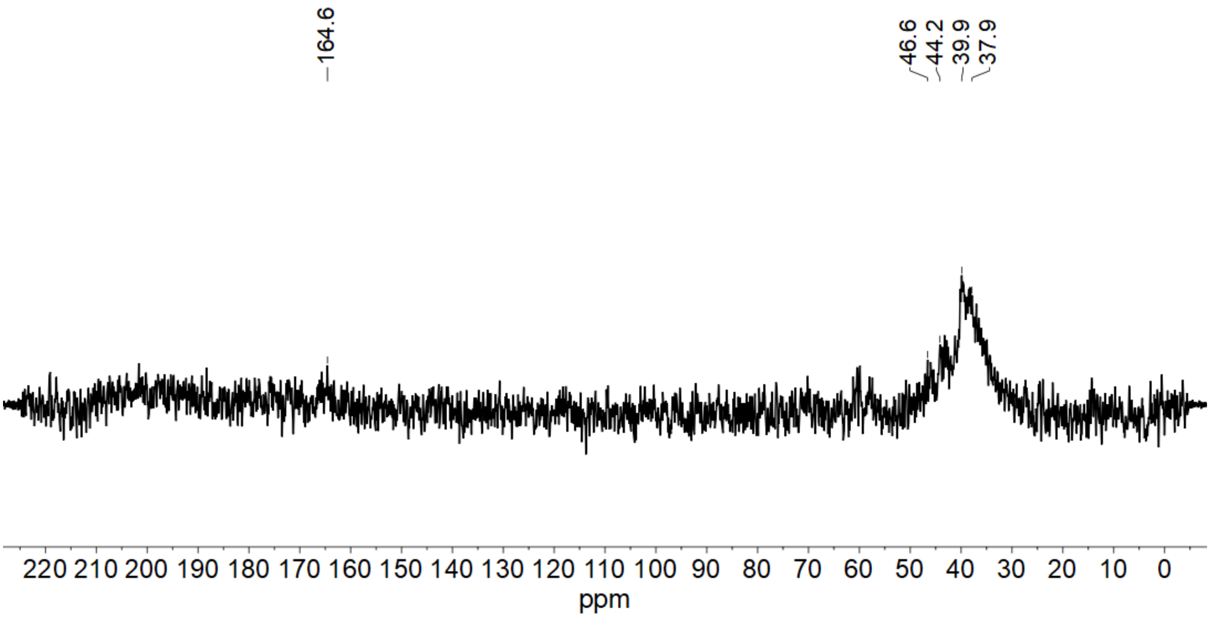

Figure S51 $^{13}C\{^{1}H\}$ NMR (126 MHz, D_2O) spectrum corresponding to Table S2; Entry 5.

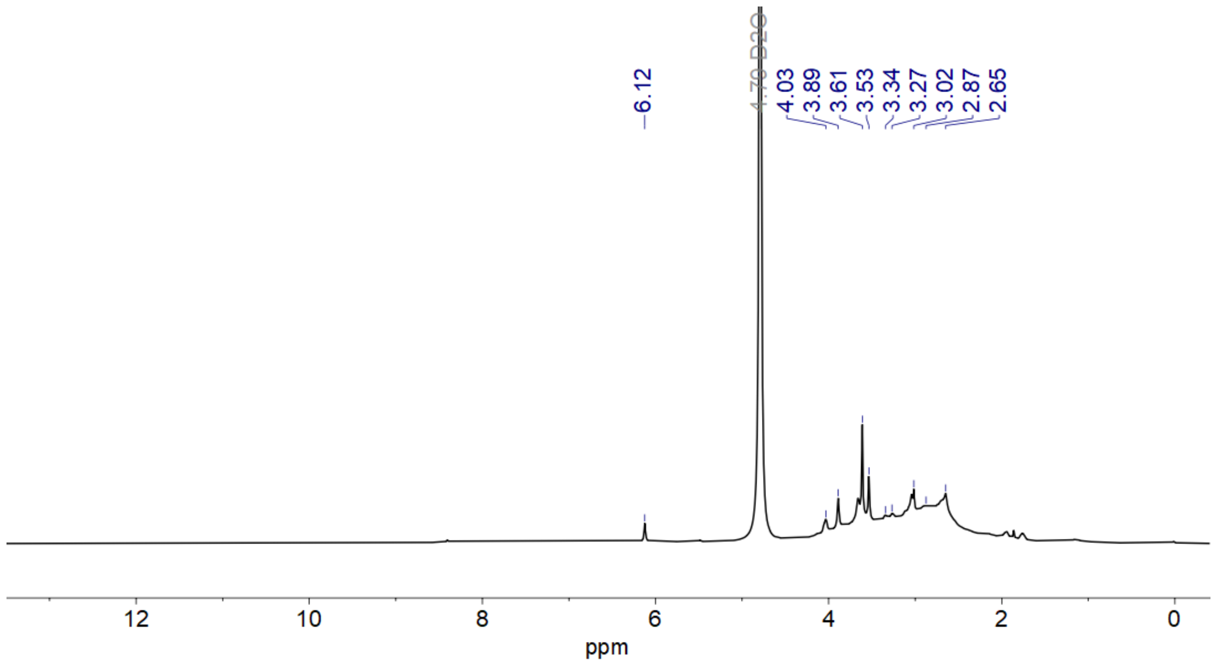

Figure S52 ^{1}H NMR (500 MHz, D_2O) spectrum corresponding to Table S2; Entry 6.

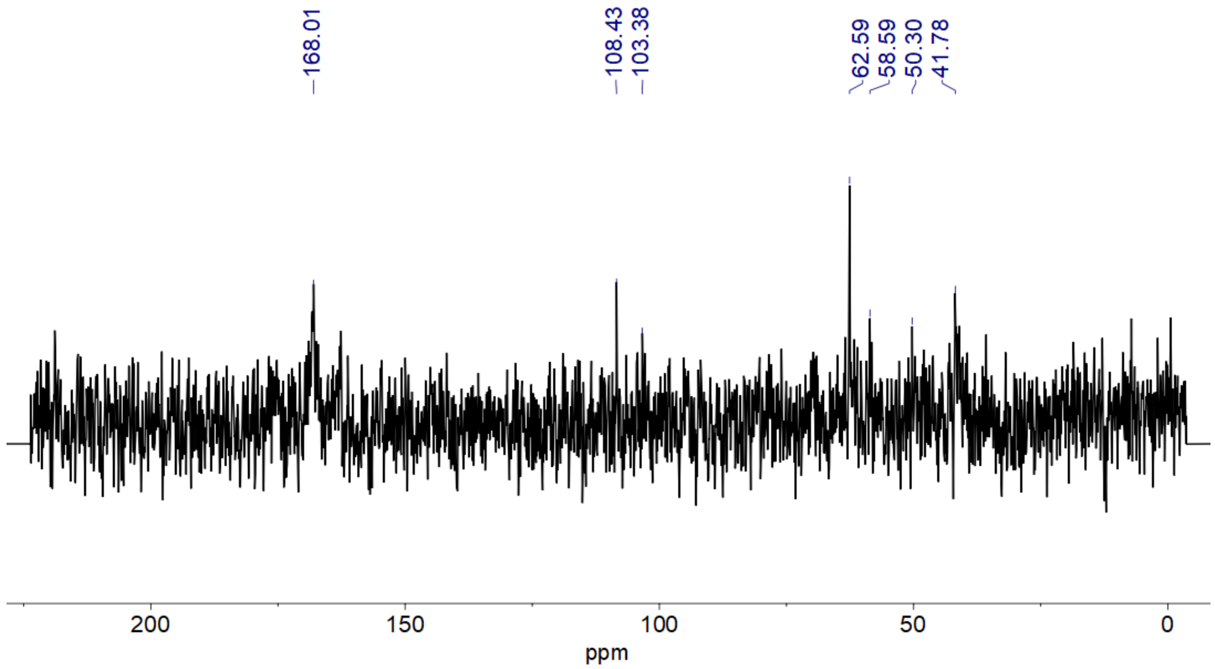

Figure S53 $^{13}C\{^{1}H\}$ NMR (500 MHz, D_2O, l.b. 10 Hz) spectrum corresponding to Table S2; Entry 6.

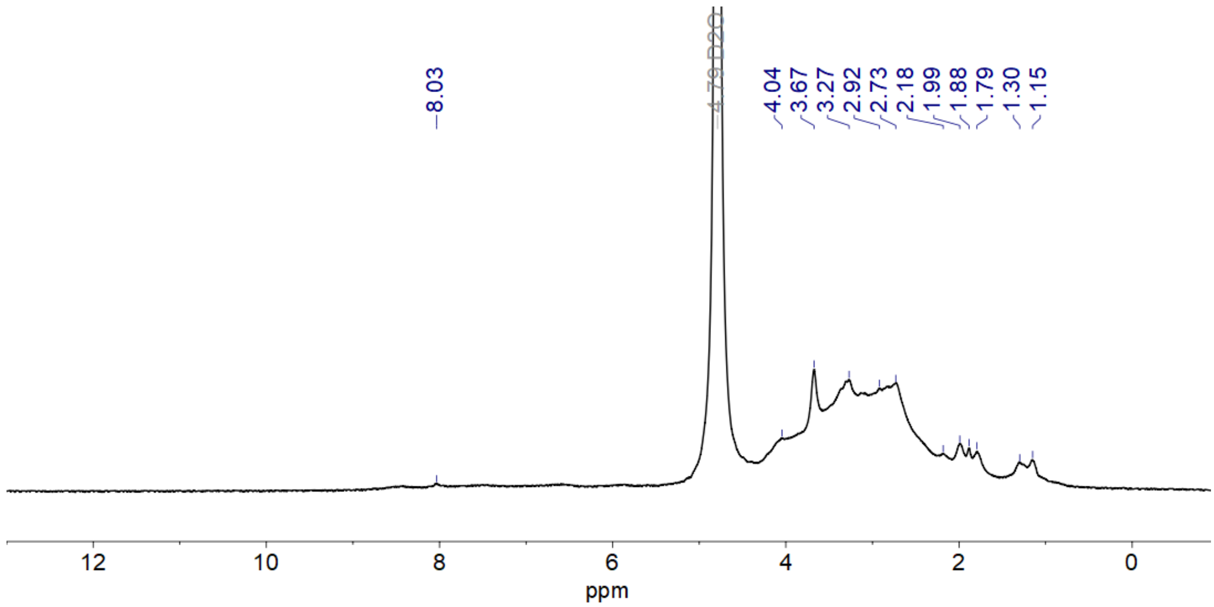

Figure S54 ^{1}H NMR (500 MHz, D_2O) spectrum corresponding to Table S2; Entry 7.

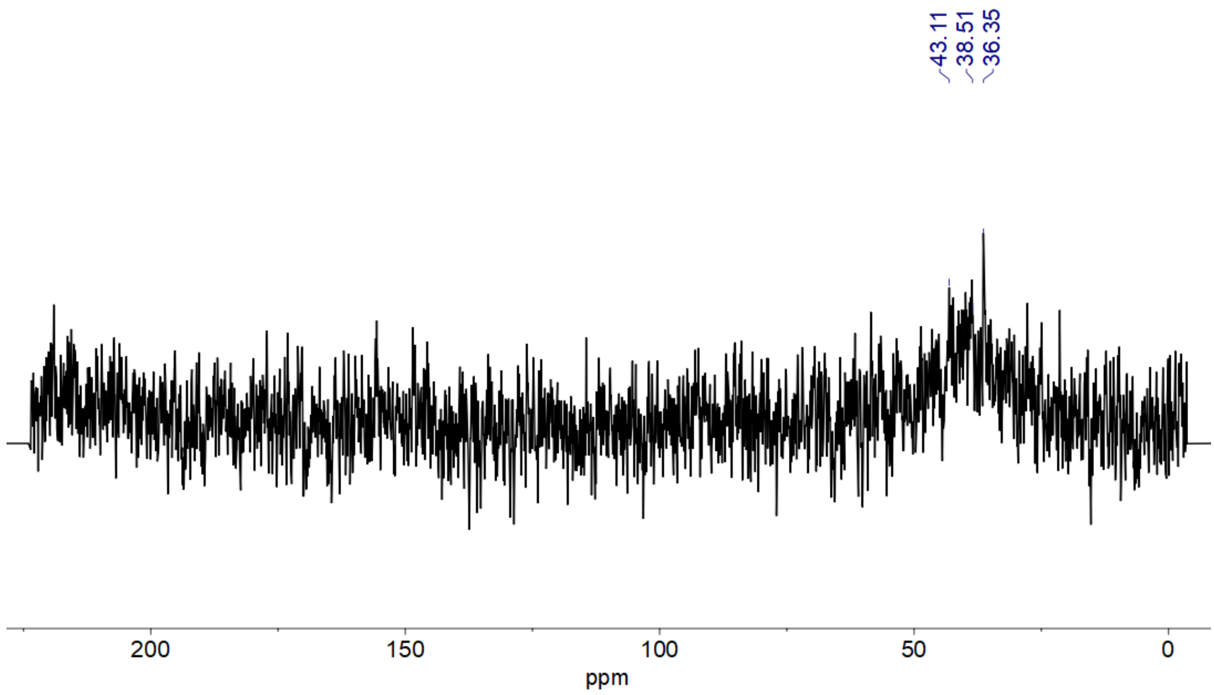

Figure S55 $^{13}C\{^1H\}$ NMR (126 MHz, D_2O, l.b. 10 Hz) spectrum corresponding to Table S2; Entry 7.

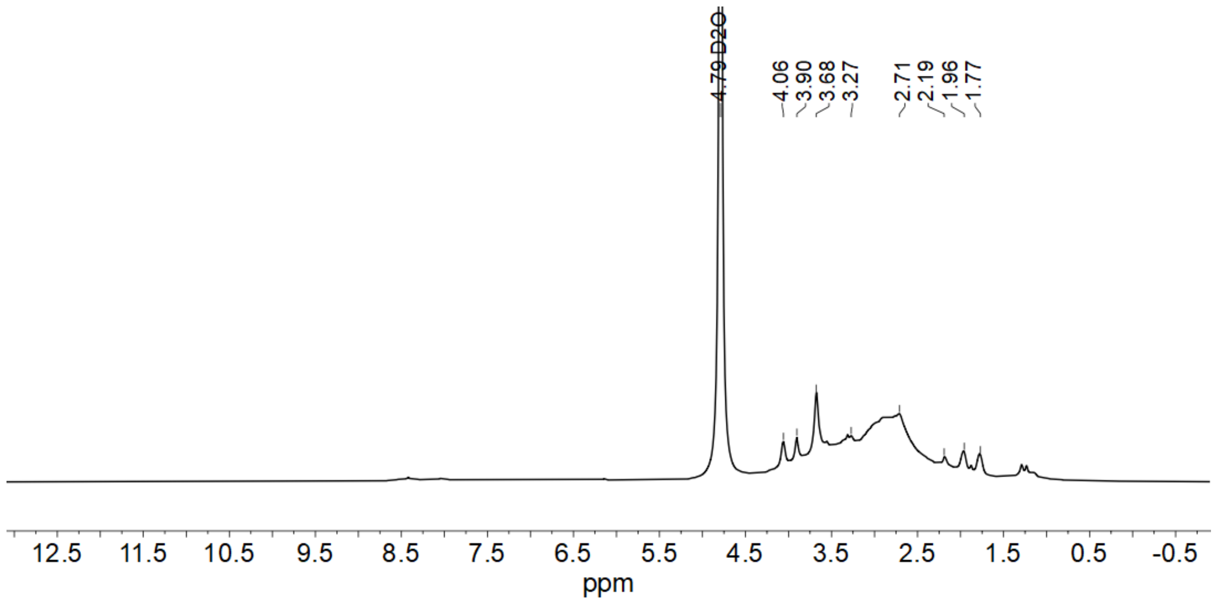

Figure S56 ^{1}H NMR (500 MHz, D_2O) spectrum corresponding to Table S2; Entry 8.

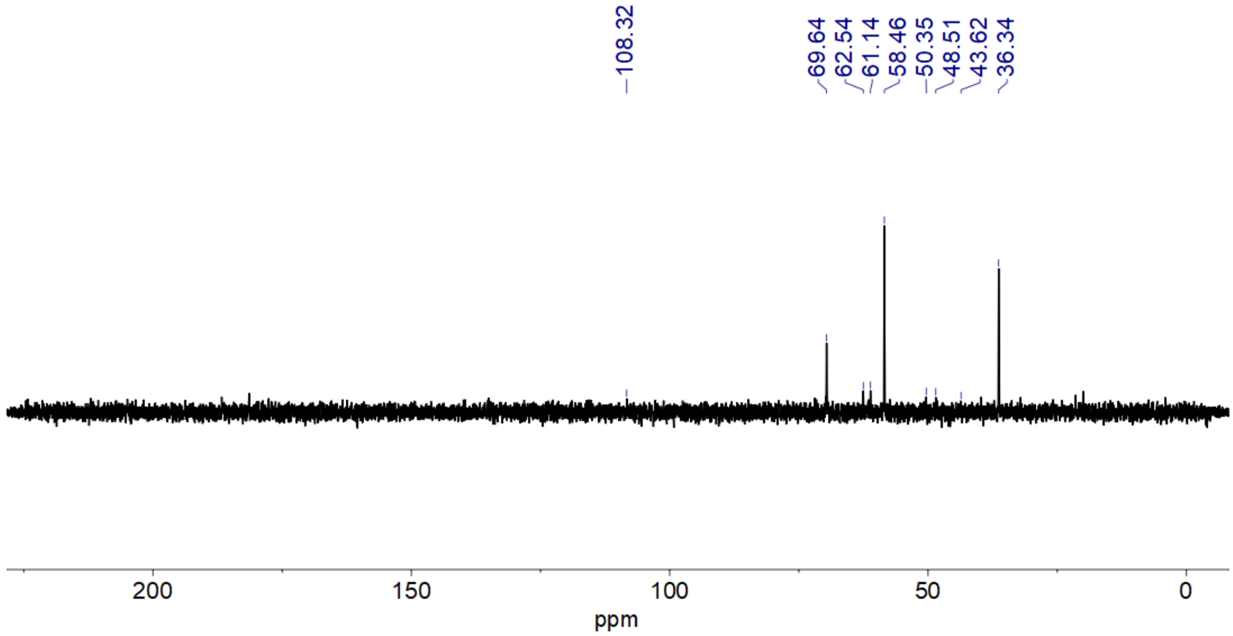

Figure S57 $^{13}C\{^{1}H\}$ NMR (126 MHz, D_2O) corresponding to Table S2; Entry 8.

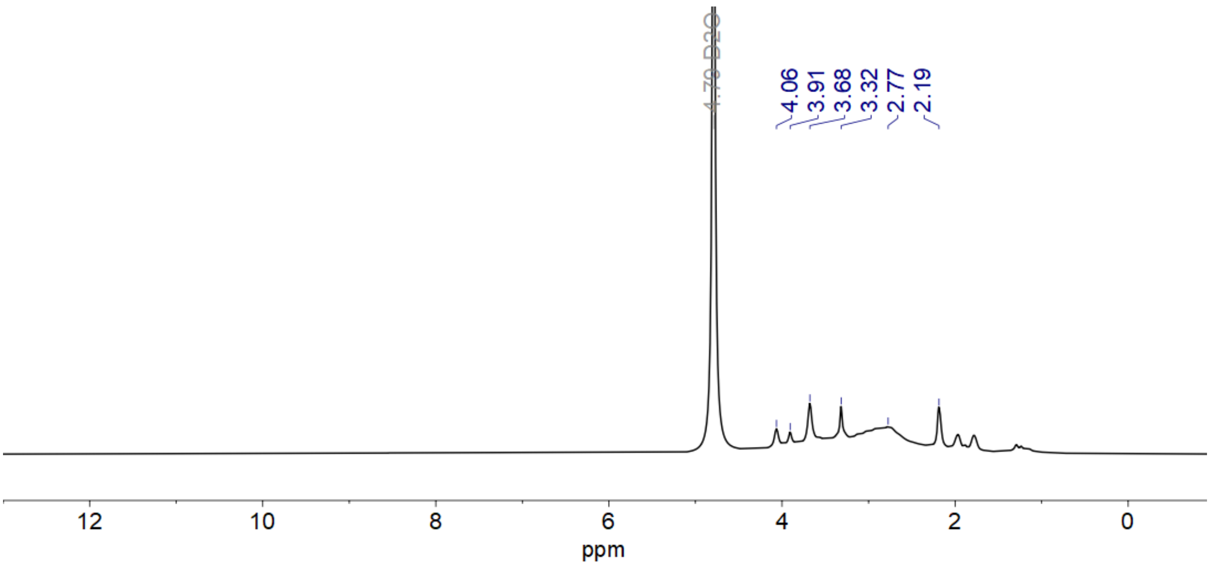

Figure S58 ^{1}H NMR (500 MHz, D_2O) spectrum corresponding to Table S2; Entry 9.

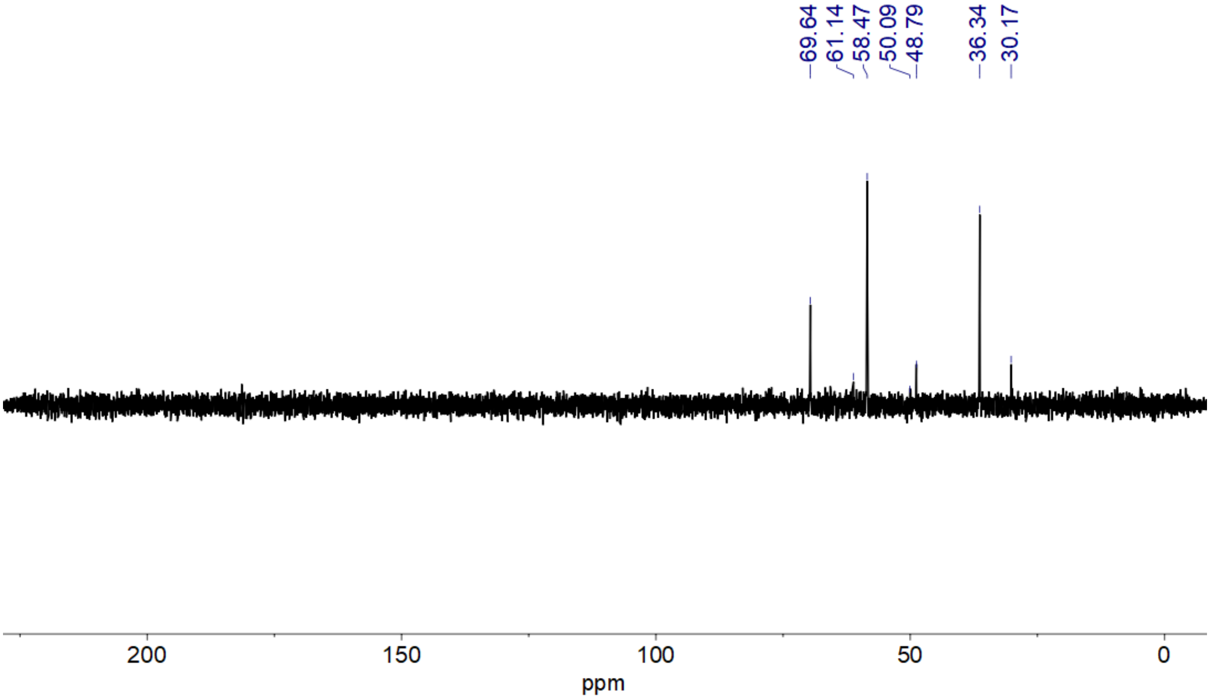

Figure S59 $^{13}C\{^{1}H\}$ NMR (126 MHz, D_2O) spectrum corresponding to Table S2; Entry 9.

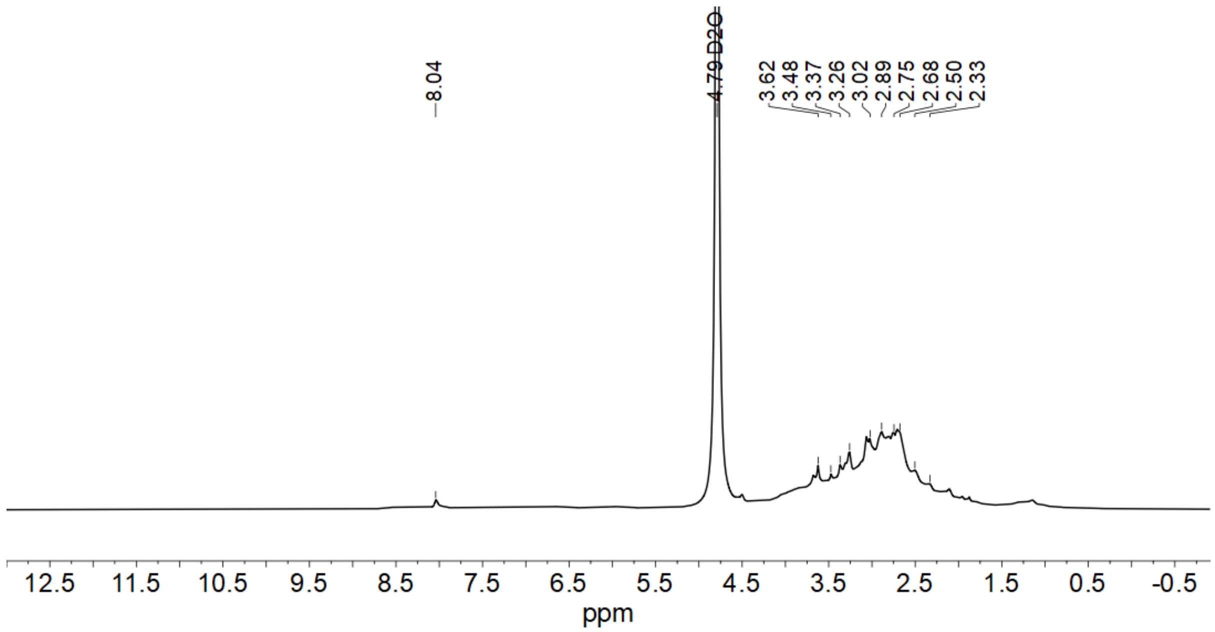

Figure S60 ^{1}H NMR (500 MHz, D_2O) corresponding to Table S2; Entry 10.

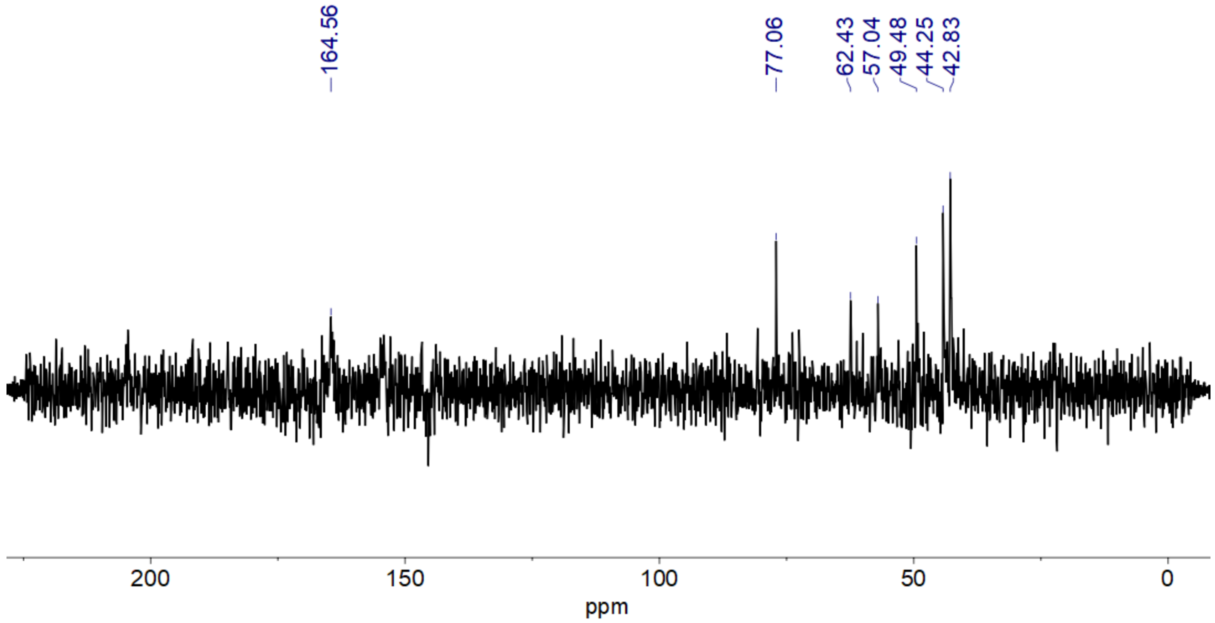

Figure S61 $^{13}C\{^{1}H\}$ NMR (126 MHz, D_2O, l.b. 10 Hz) spectrum corresponding to Table S2; Entry 10.

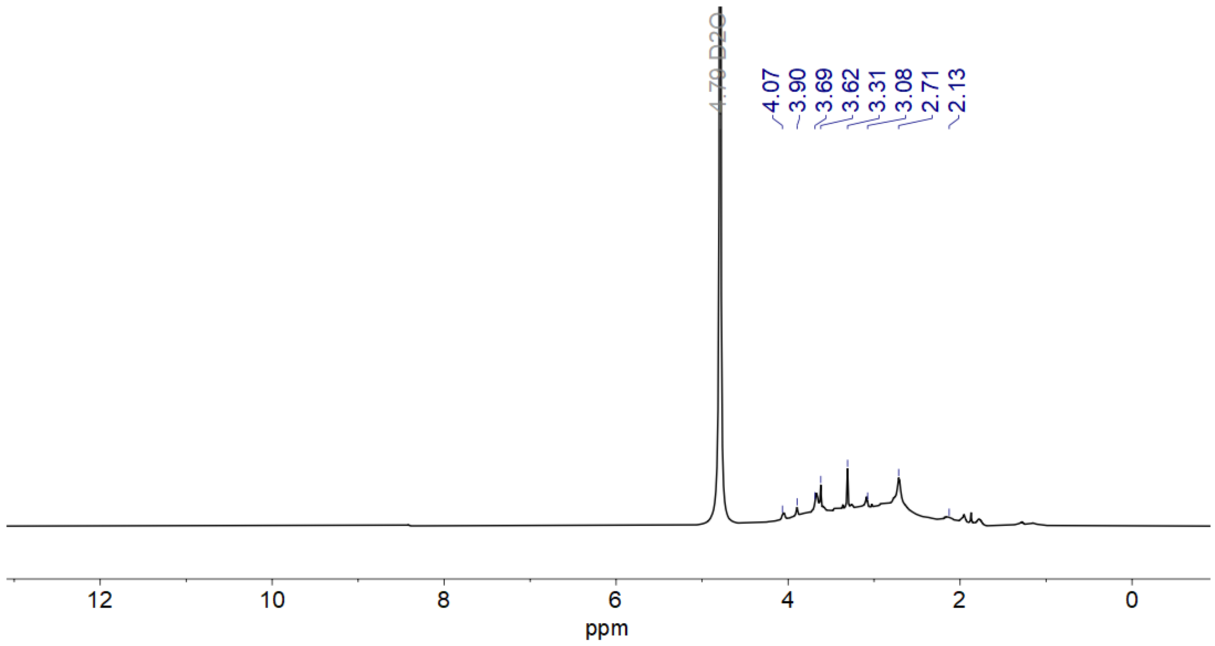

Figure S62 ^{1}H NMR (500 MHz, D_2O) spectrum corresponding to Table S2; Entry 11.

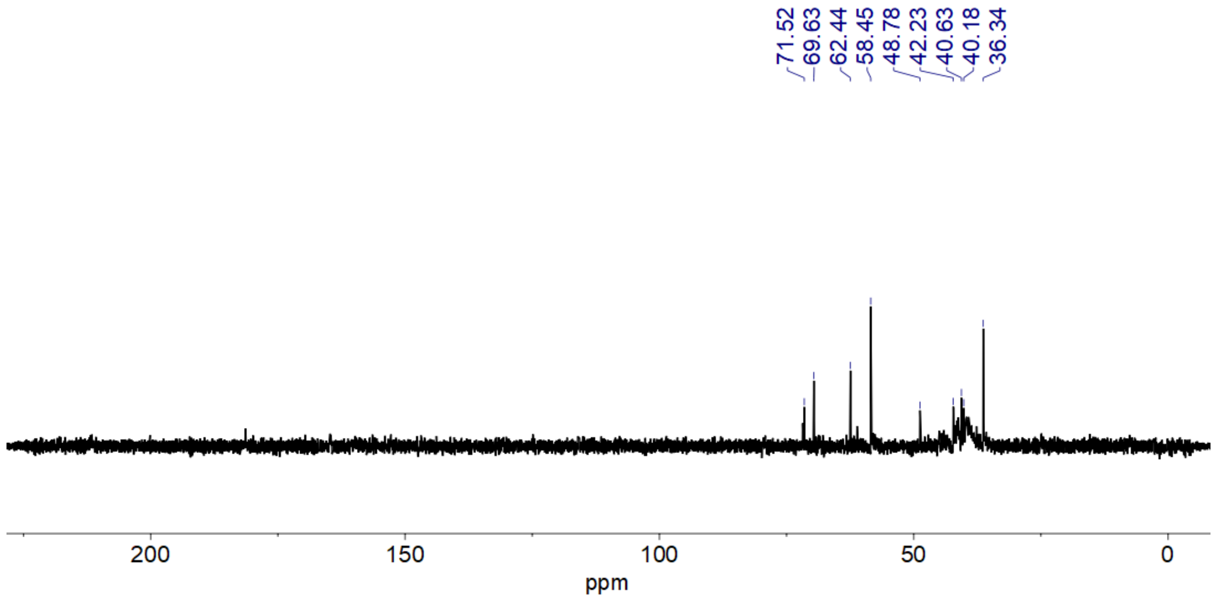

Figure S63 $^{13}C\{^{1}H\}$ NMR (126 MHz, D_2O) spectrum corresponding to Table S2; Entry 11.

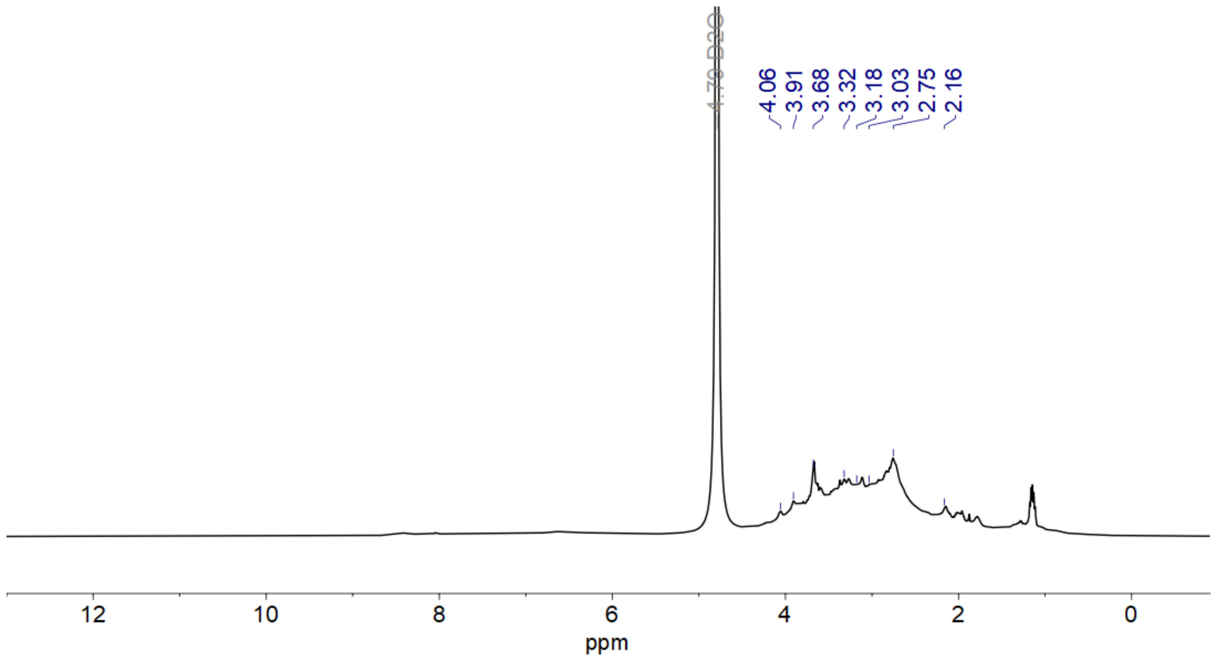

Figure S64 ^{1}H NMR (500 MHz, D_2O) spectrum corresponding to Table S2; Entry 12.

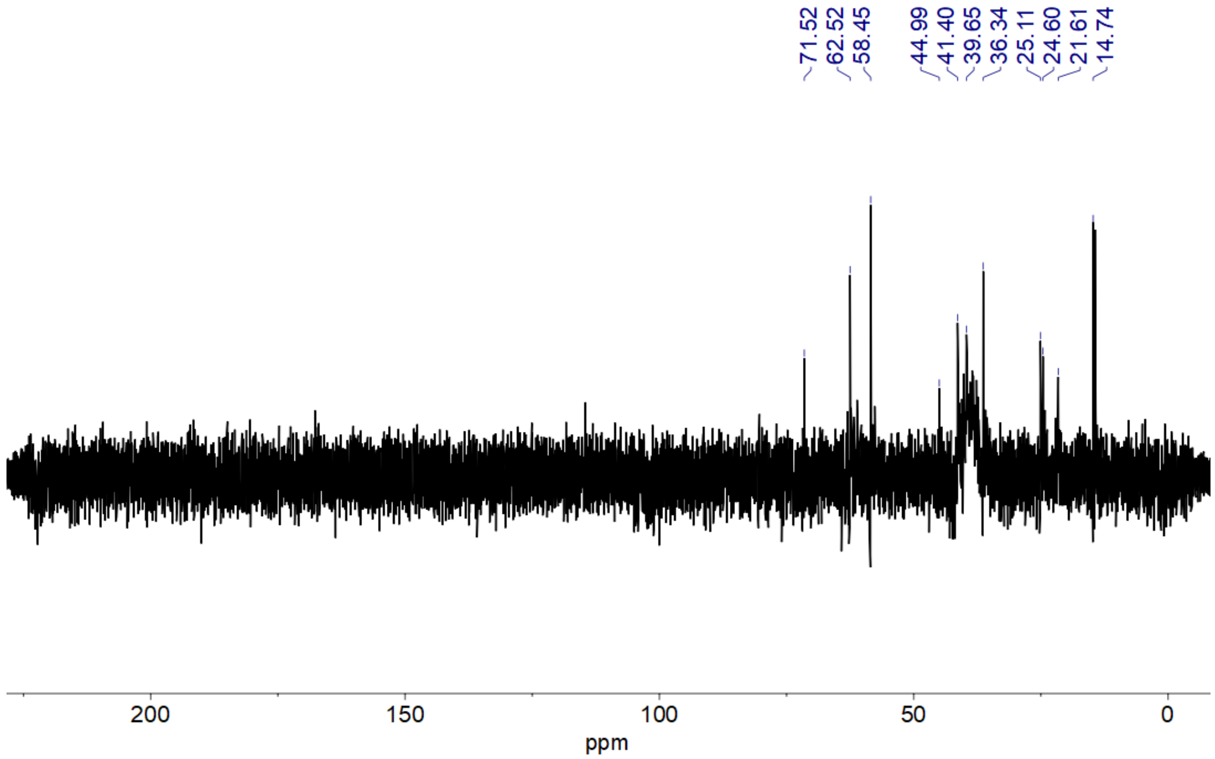

Figure S65 $^{13}C\{^{1}H\}$ NMR (126 MHz, D_2O) spectrum corresponding to Table S2; Entry 12.

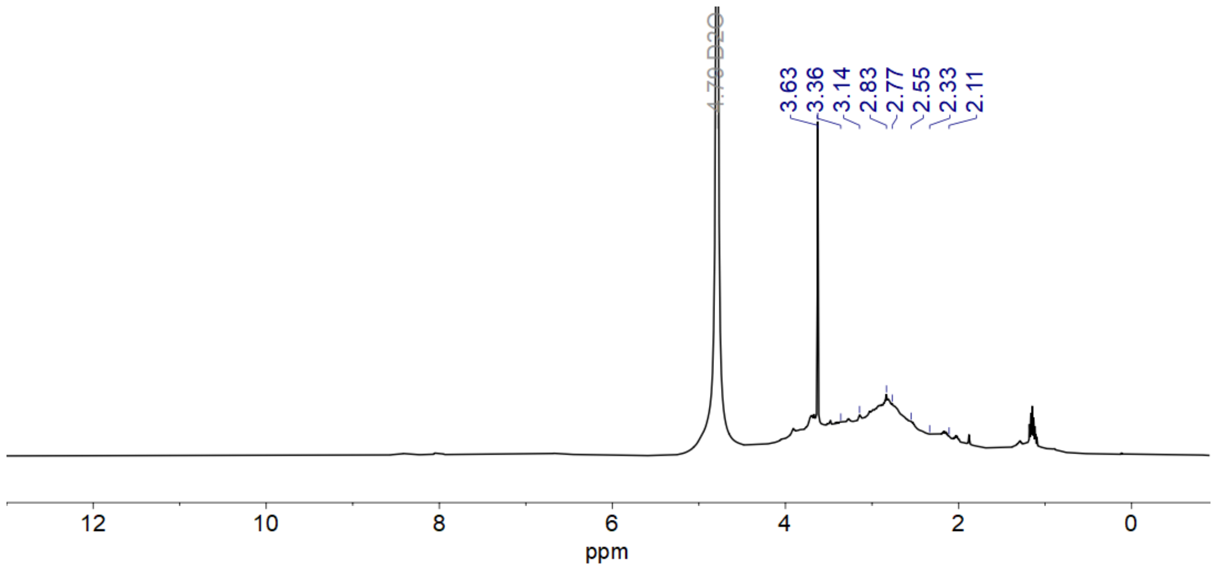

Figure S66 ^{1}H NMR (500 MHz, D_2O) spectrum corresponding to Table S2; Entry 13.

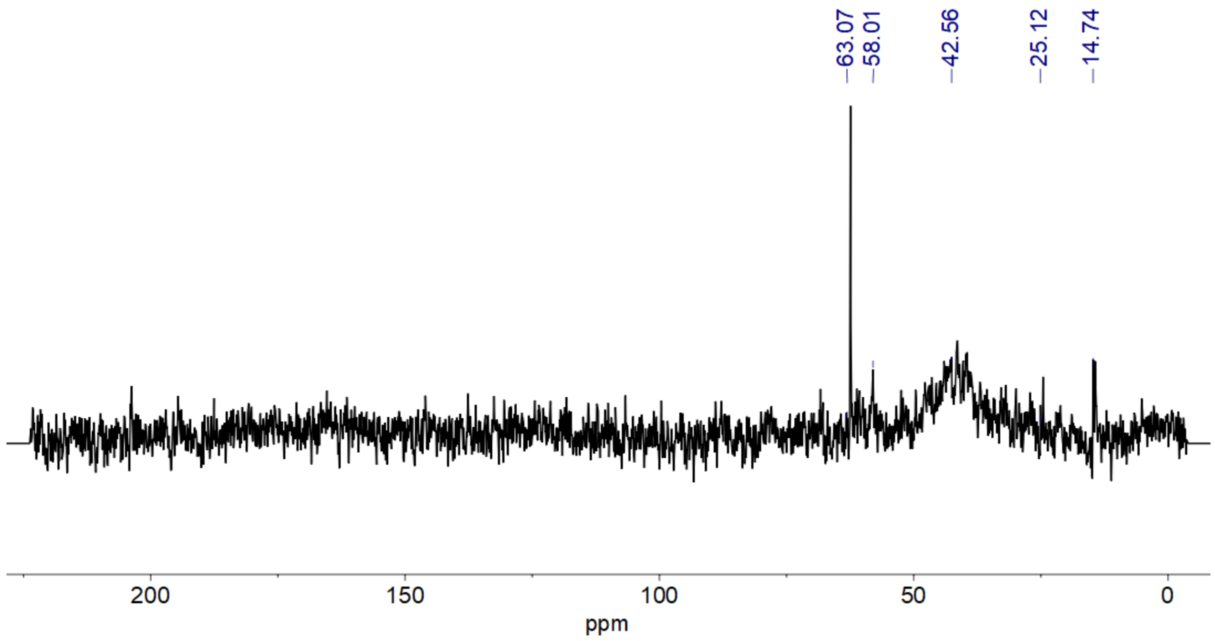

Figure S67 $^{13}C\{^{1}H\}$ NMR (126 MHz, D_2O, l.b. 10 Hz) spectrum corresponding to Table S2; Entry 13.

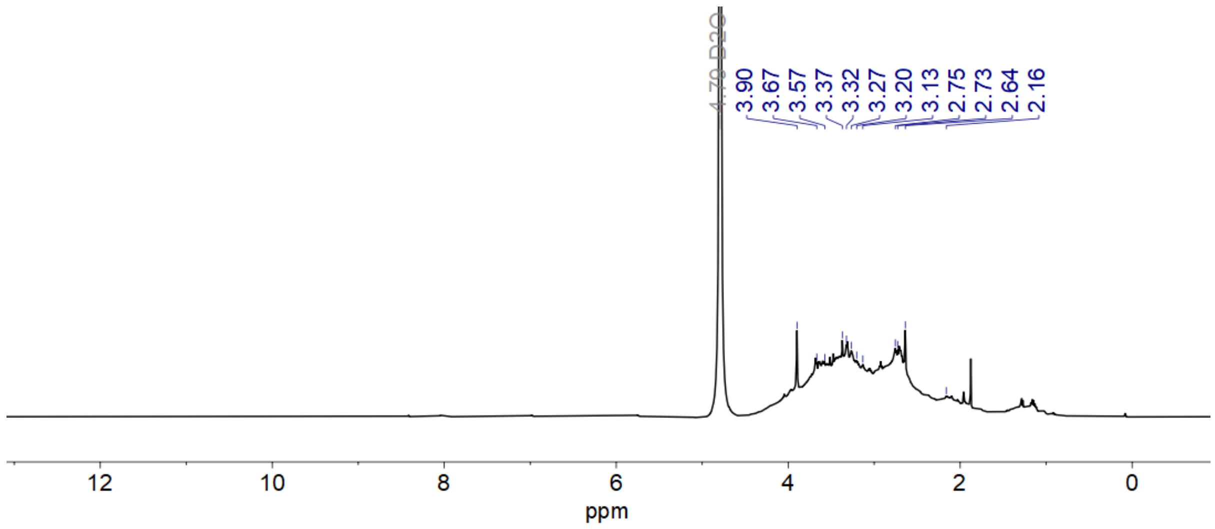

Figure S68 ^{1}H NMR (500 MHz, D_2O) spectrum corresponding to Table S2; Entry 14.

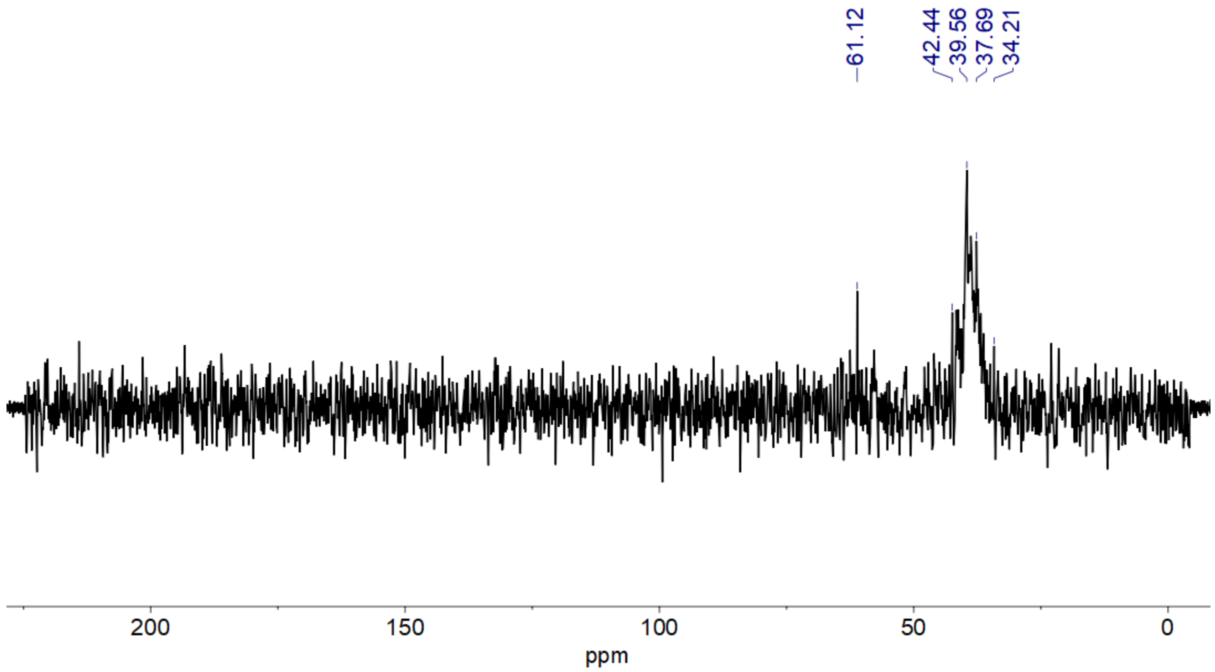

Figure S69 $^{13}C\{^{1}H\}$ NMR (126 MHz, D_2O, l.b. 10 Hz) spectrum corresponding to Table S2; Entry 14.

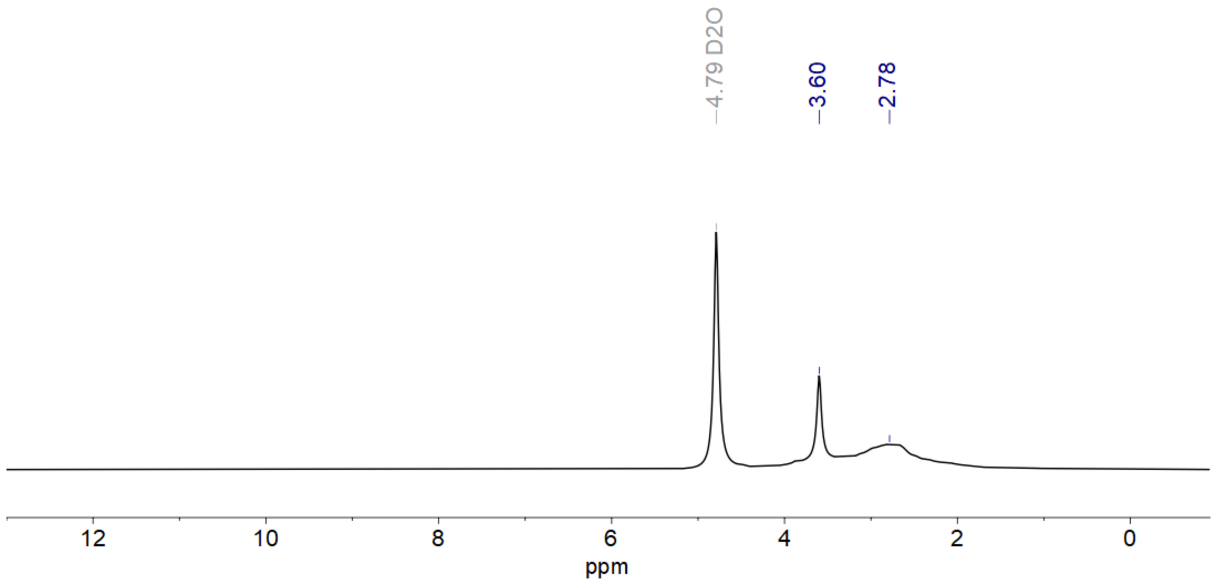

Figure S70 ^{1}H NMR (500 MHz, D_2O) spectrum corresponding to Table S2; Entry 20. Signal at δ_C 3.60 corresponds to ethylene glycol.

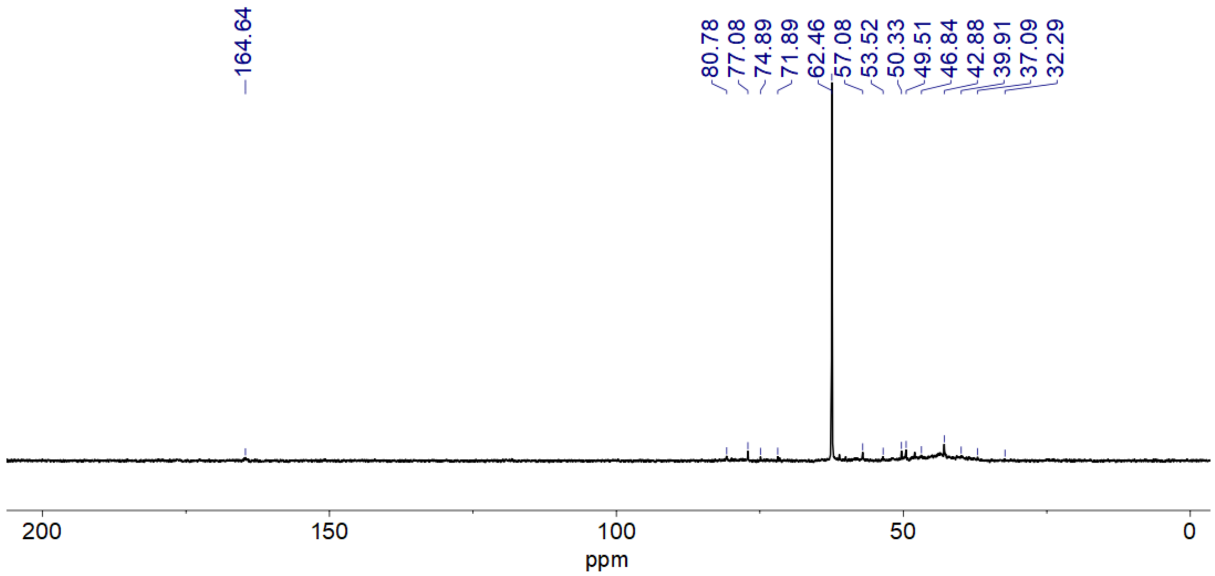

Figure S71 $^{13}C\{^1H\}$ NMR (126 MHz, D_2O, l.b. 10 Hz) spectrum corresponding to Table S2; Entry 14. Signal at δ_C 62.5 corresponds to ethylene glycol.

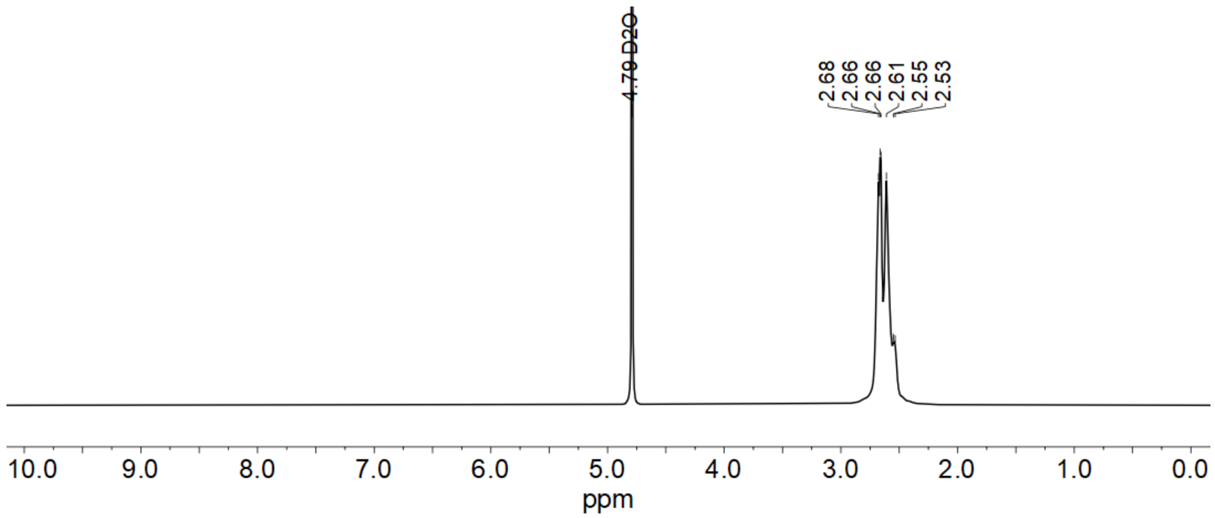

Figure S72 ^{1}H NMR (500 MHz, D_2O) spectrum of commercial sample of branched-PEI, with MW 10,000 Da and $-NH_2$ end groups.

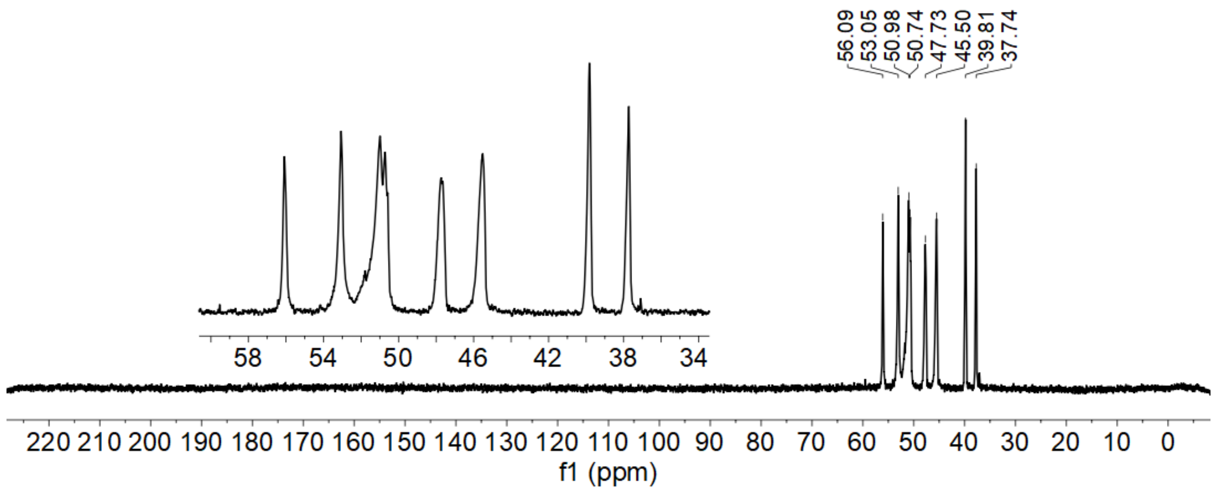

Figure S73 $^{13}C\{^{1}H\}$ NMR (126 MHz, D_2O) spectrum of commercial sample of branched-PEI, with MW 10,000 Da, and $-NH_2$ end-groups.

1.4.5 GPC Traces

Note: The spectroscopic data for the sample corresponding to Table S2; Entry 6 show the presence of significant residual K_2CO_3. As such, GPC data for this sample was not collected.

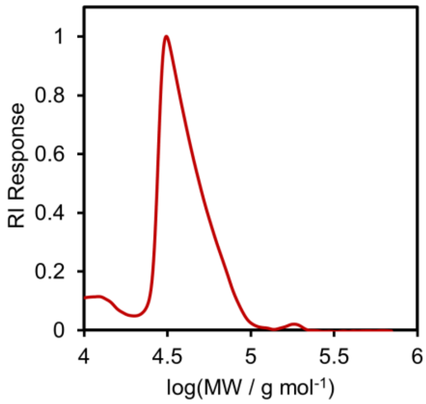

Figure S74 GPC chromatograph corresponding to a commercial sample of branched PEI with reported MW 10,000 g mol^{-1}.

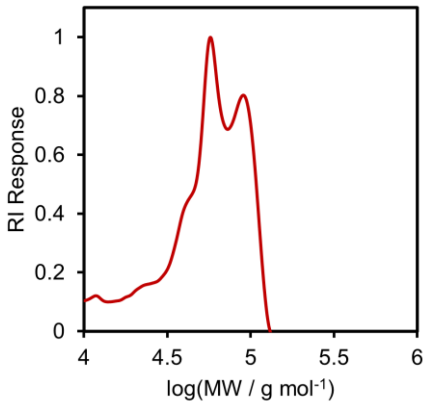

Figure S75 GPC chromatograph corresponding to Table S2; Entry 2.

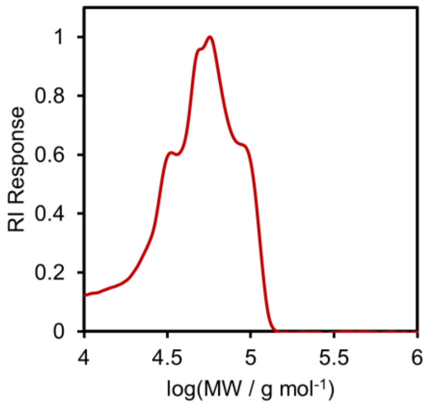

Figure S76 GPC chromatograph corresponding to Table S2; Entry 3.

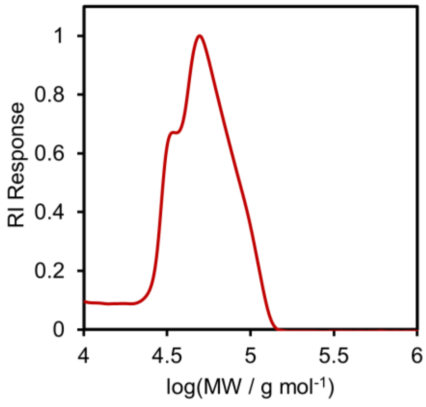

Figure S77 GPC chromatograph corresponding to Table S2; Entry 4.

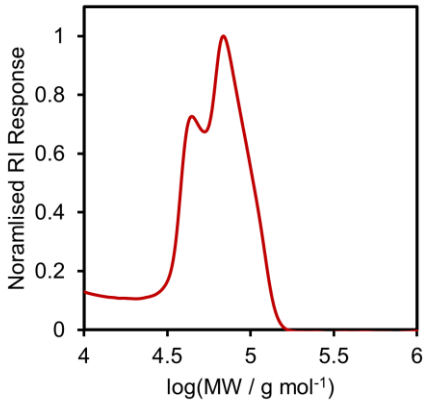

Figure S78 GPC chromatograph corresponding to Table S2; Entry 5.

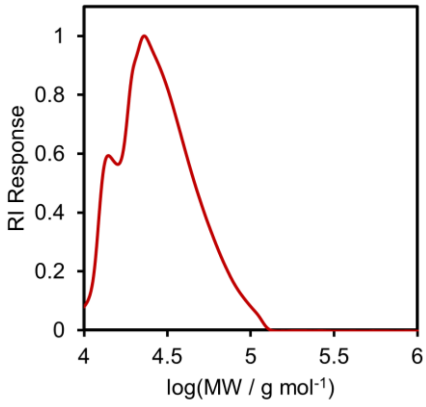

Figure S79 GPC chromatograph corresponding to Table S2; Entry 7.

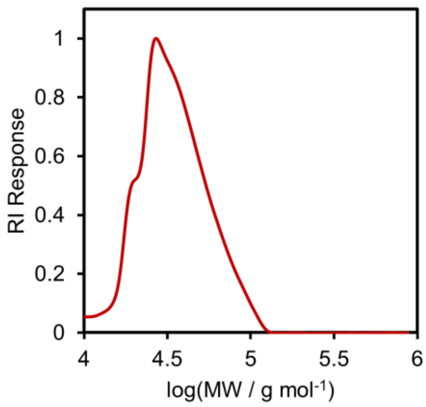

Figure S80 GPC chromatograph corresponding to Table S2; Entry 8.

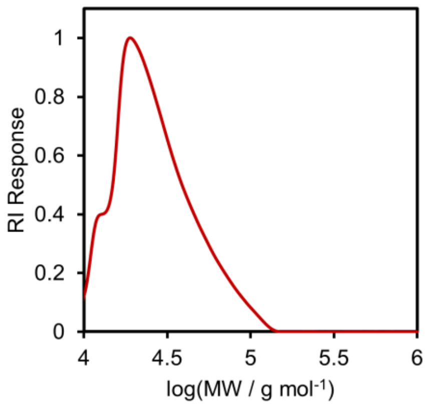

Figure S81 GPC chromatograph corresponding to Table S2; Entry 9.

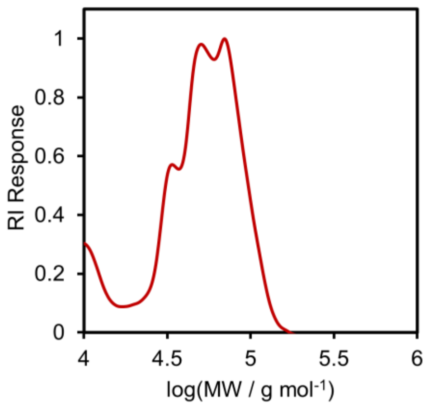

Figure S82 GPC chromatograph corresponding to Table S2; Entry 10.

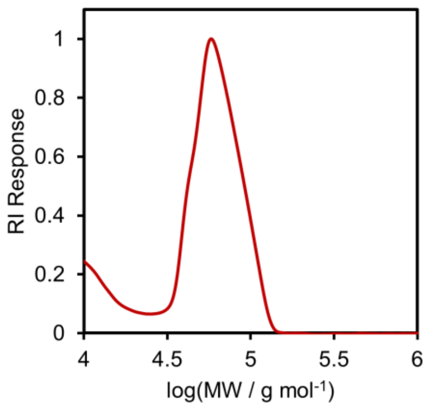

Figure S83 GPC chromatograph corresponding to Table S2; Entry 11.

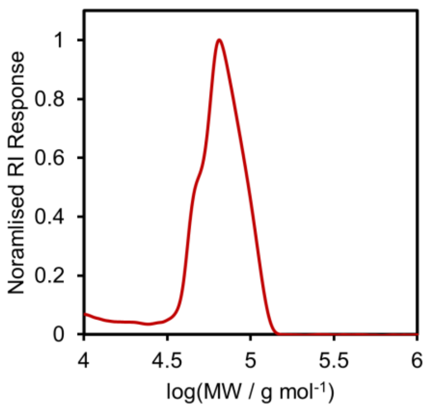

Figure S84 GPC chromatograph corresponding to Table S2; Entry 12.

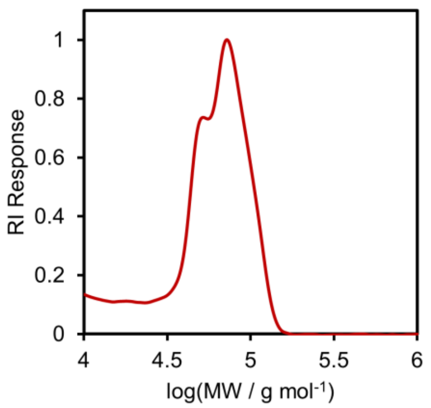

Figure S85 GPC chromatograph corresponding to Table S2; Entry 13.

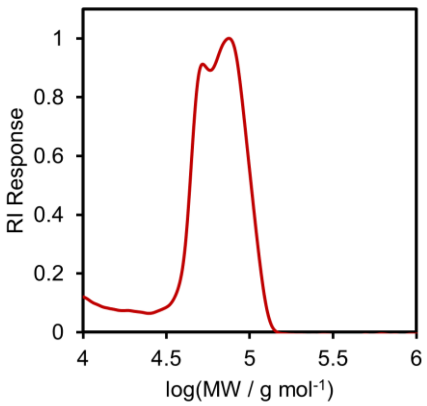

Figure S86 GPC chromatograph corresponding to Table S2; Entry 14.

1.4.6 EI-MS spectra

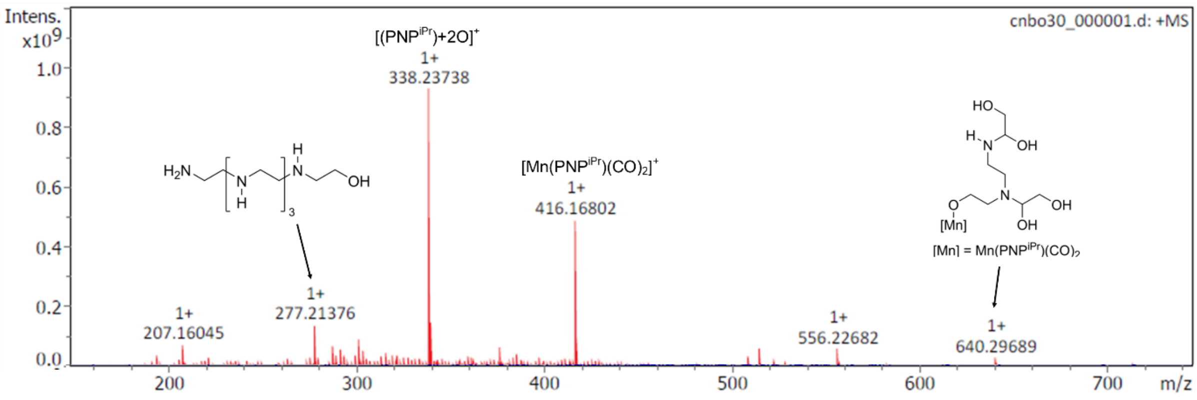

Figure S87 EI-MS spectrum corresponding to Table S2; Entry 11.

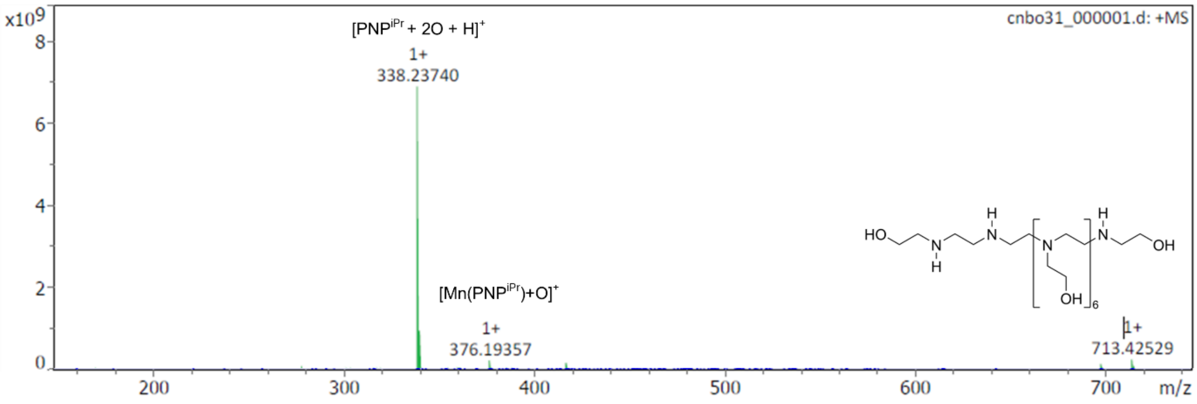

Figure S88 EI-MS spectrum corresponding to Table S2; Entry 12.

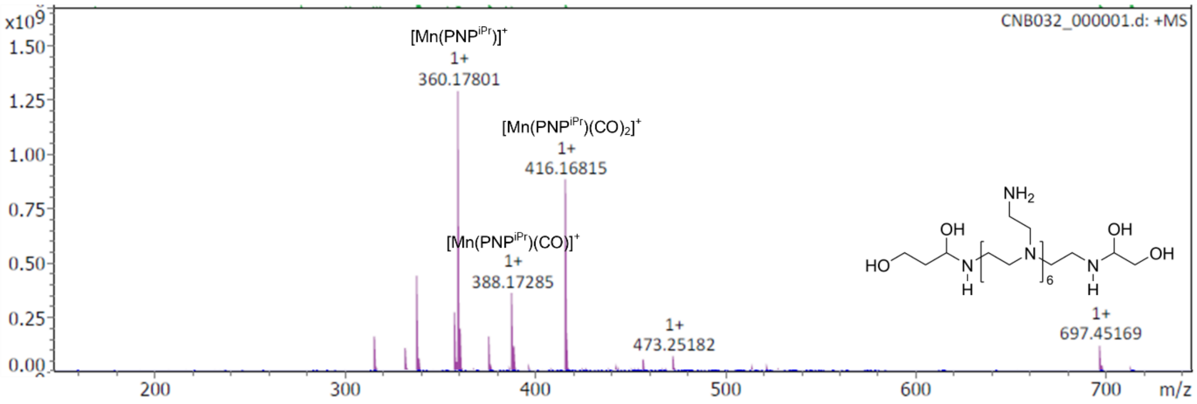

Figure S89 EI-MS spectrum corresponding to Table S2; Entry 13.

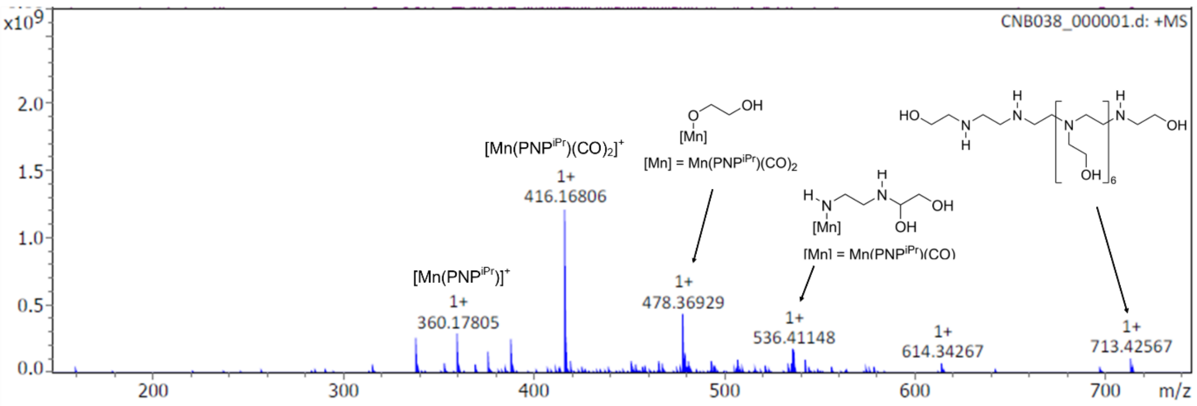

Figure S90 EI-MS spectrum corresponding to Table S2; Entry 14.

1.5 Mechanistic Studies

1.5.1 Reaction of **2** with ethylene glycol

To an NMR tube equipped with a J-Young's NMR valve was added **1** (7.0 mg, 14 μmol), KOtBu (1.3 mg, 14 μmol) and toluene-*d*8 (0.5 mL). The tube was heated to 110 °C for 10 minutes to generate **6** quantitatively. After cooling to room temperature, a drop of ethylene glycol was added and the NMR tube shaken vigorously at room temperature. The reaction was probed *in situ* by NMR spectroscopies (^{1}H, $^{31}P\{^{1}H\}$, $^{13}C\{^{1}H\}$) and complete conversion of **6** to **8** was observed.

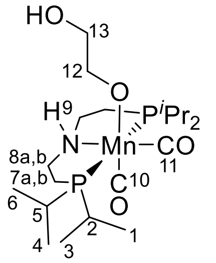

^{1}H NMR (500 MHz, toluene-*d*8): δ_H 3.86 (br s, 2H, H-12), 3.63 (br s, 2H, H-13), 3.30 (s, residual ethylene glycol), 3.24-3.16 (m, 2H, H-2), 2.81-2.76 (m, 1H, H-9), 2.58-2.46 (m, 2H, H-8a/b), 2.26-2.17 (m, 2H, H-5), 1.98-1.90 (m, 2H, H-8a/b), 1.73-1.65 (m, 2H, H-7a/b), 1.49-1.45 (*apparent* q, 6H, *J* = 7.4 Hz, H-6), 1.32-1.29 (*apparent* q, 6H, *J* = 7.0 Hz, H-4), 1.26-1.23 (m, 2H, H-7a/b), 1.22-1.18 (*apparent* q, *J* = 7.3 Hz, 6H, H-3), 1.09-1.06 (m, 6H, H-1), 1.05 (s, tBuOH).

In addition to the discrete complex signals observed in the ^{1}H NMR there is also observed some very broad resonances with δ_H 6.01 (FWHM = 225 Hz) and δ_H 4.14 (FWHM = 200 Hz), likely the result of additional molecules of ethylene glycol H-bonding to **8**.

$^{31}P\{^{1}H\}$ NMR (203 MHz, toluene-*d*8): δ_P 81.3

$^{13}C\{^{1}H\}$ NMR (126 MHz, toluene-*d*8): δ_C 72.6 (C-13), 68.6 (tBuOH), 65.0 (br, C-12), 64.2 (residual ethylene glycol), 53.1 (t, *J* = 5.4 Hz, C-8), 31.6 (tBuOH), 27.6 (t, *J* = 6.2 Hz, C-7), 26.5 (t, *J* = 9.3 Hz, C-2), 24.8 (t, *J* = 9.3 Hz, C-5), 19.2 (C-3), 18.7 (C-1).

Note: $^{13}C\{^{1}H\}$ chemical shifts corresponding to C4 and C6 were obscured by residual solvent signal, as determined by $^{1}H,^{13}C$-HSQC NMR spectroscopy.

1.5.3 Evidence for the release of H_2 gas

To determine whether the reaction proceeds via a (de)hydrogenative pathway, the polymerisation of ethylene glycol and ethylene diamine was carried out in a 200 cm^3 dual-vessel J-young's H-flask (maximum H_2 pressure obtainable (1 eq., 2 mmol at 150 °C) is 0.35 bar overpressure) with sinter separating the vessels such that the gaseous atmosphere present is shared by both vessels (Figure S91).

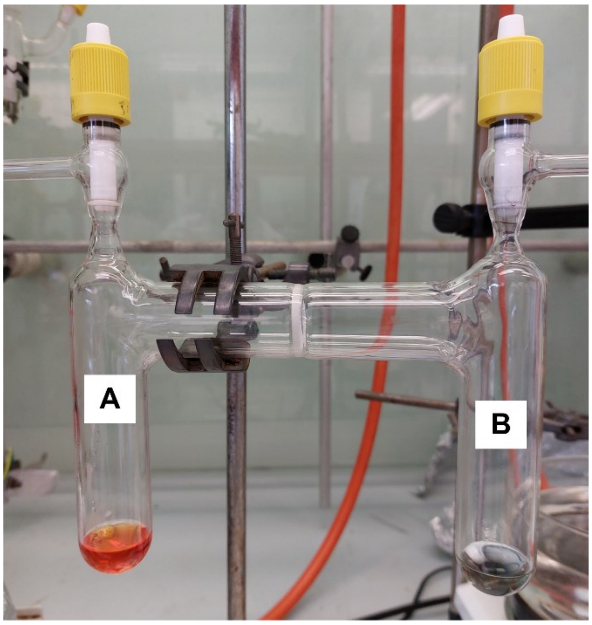

Figure S91 Reaction set up to probe the production of H_2 gas.

To vessel A was added ethylene glycol (0.14 mL, 2 mmol), ethylene diamine (0.11 mL, 2 mmol), KOtBu (22.4 mg, 0.2 mmol), **1** (1 mol%, 9.9 mg, 0.02 mmol) and toluene (4 mL). The reaction in vessel A was heated to 150 °C for 24 hours. To vessel B was added benzaldehyde (100 mg, 0.94 mmol), Pd/C (10% Pd, type 487: 2 mol%, 20 mg, 19 µmol) and toluene (2 mL). The reaction in vessel B was heated to 60 °C for 24 hours. The apparatus is allowed to cooled to room temperature. The product within vessel A is extracted into distilled water (5 mL) and volatile components removed under reduced pressure at 110 °C to yield polymer (35 mg) product as a mixture of polyamine, polyimine and polyamide. The solution within vessel B was filtered and the products diluted into DCM and investigated by GC-MS and 8% conversion of benzaldehyde to benzyl alcohol was observed.

Reaction A:

^{1}H NMR (500 MHz, D_2O): δ_H 8.41 (CONH), 8.01 (CH=N), 4.04 (s), 3.61 (residual ethylene glycol), 3.28 (br s), 3.02 (br s), 2.80 (br s), 2.65 (br s).

^{13}C{^{1}H} NMR (D_2O, 126 MHz, l.b. 5 Hz): 182.0 (C=O), 164.4 (C=N), 73.9, 71.9, 69.7, 62.4 (residual ethylene glycol), 57.9, 500.8, 49.7, 42.9, 40.8, 36.4.

Reaction B:

GC-MS (DCM): *m/z* C_7H_8O, calc.108.06; found 108.04.

1.5.4 NMR Spectra

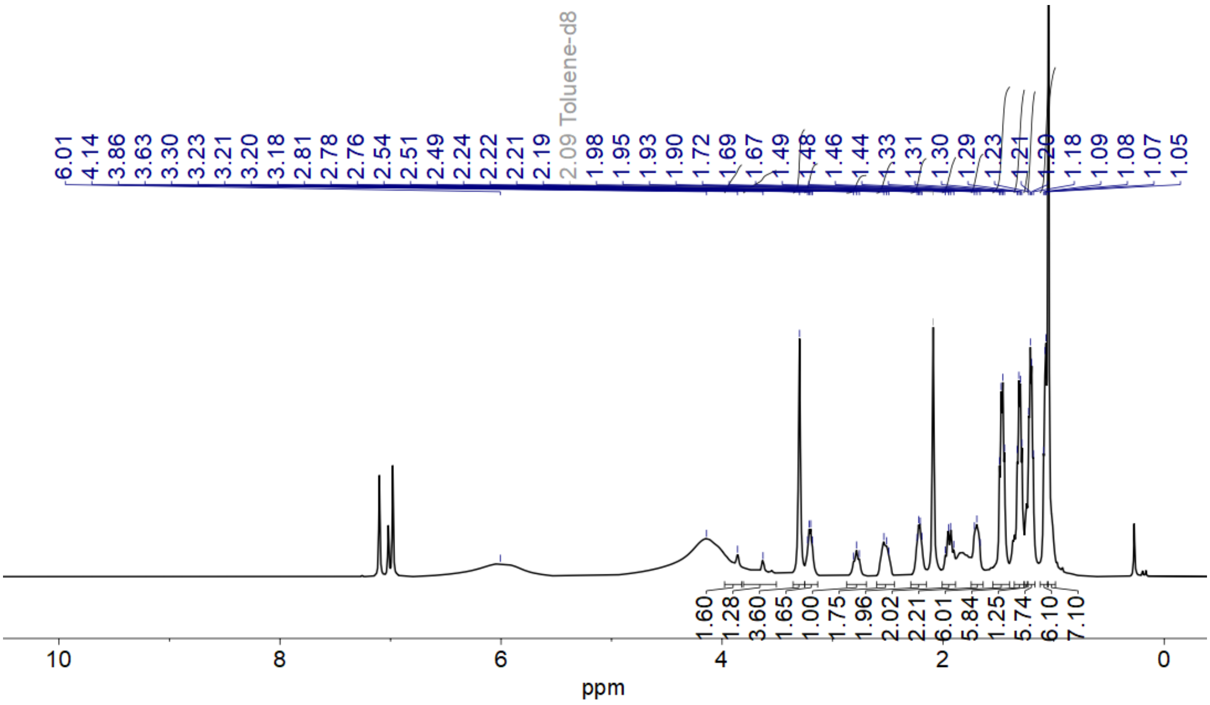

Figure S92 ^{1}H NMR (500 MHz, toluene-d_8) spectrum of **8**.

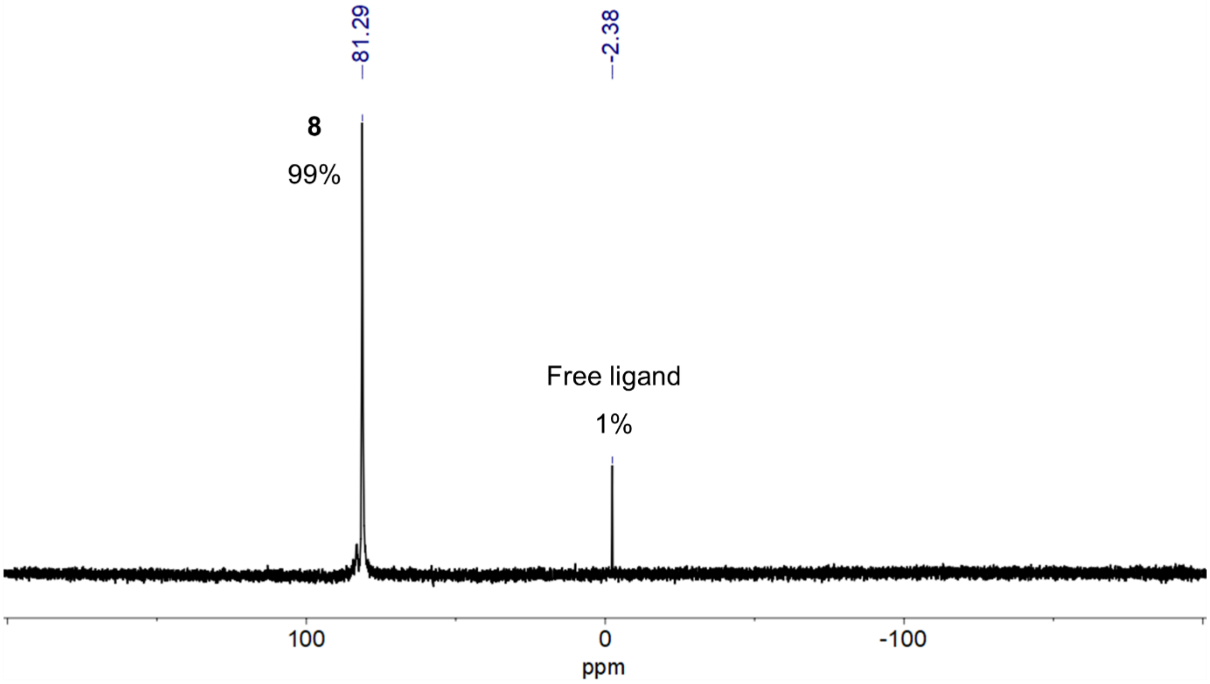

Figure S93 $^{31}P\{^{1}H\}$ NMR (203 MHz, toluene-d_8) spectrum of **8**.

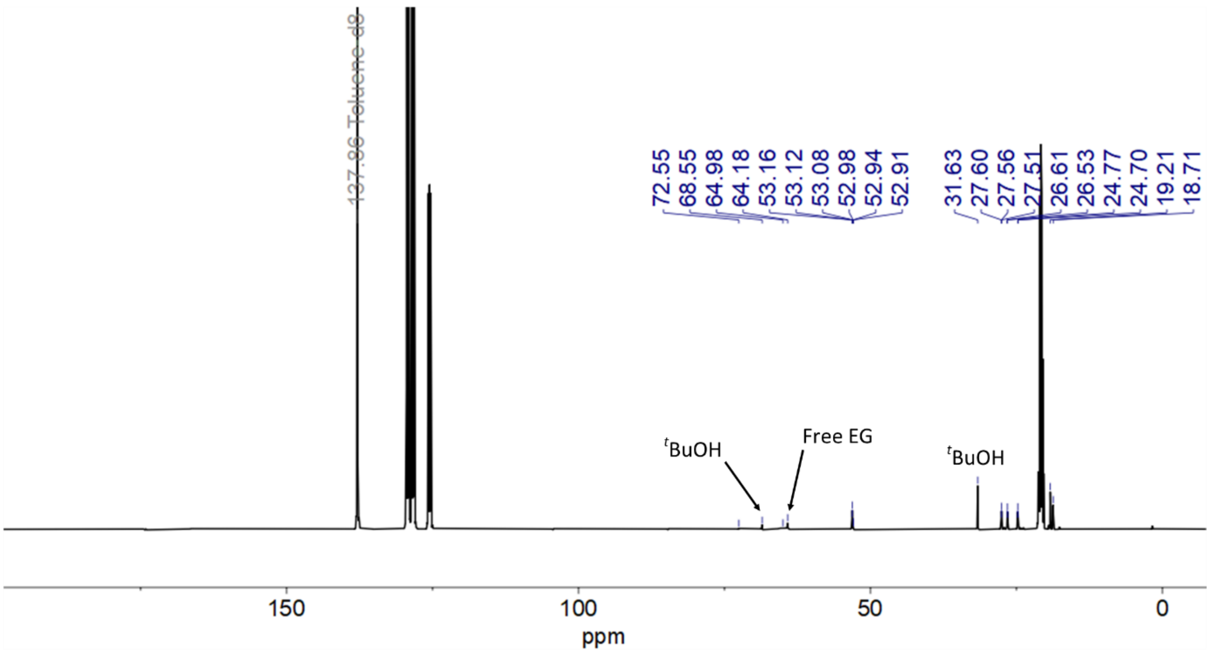

Figure S94 $^{13}C\{^{1}H\}$ NMR (126 MHz, toluene-d_8) spectrum of **8**. EG denotes ethylene glycol.

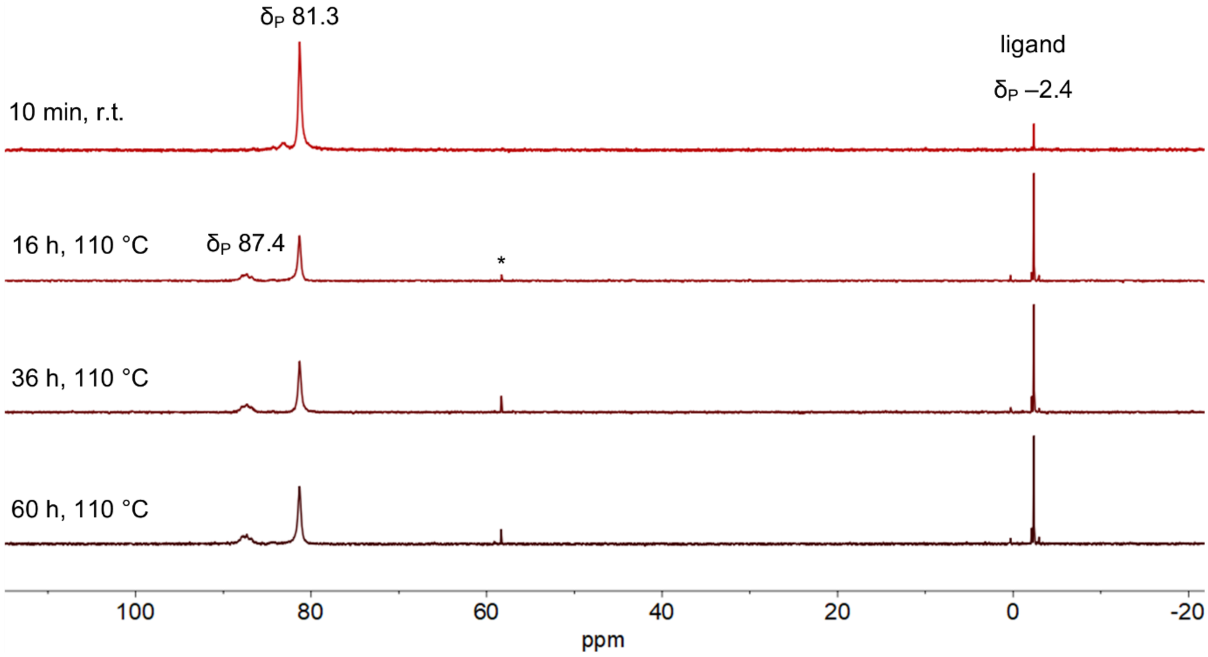

Figure S95 $^{31}P\{^{1}H\}$ NMR (203 MHz, toluene-d_8) spectrum of mixture obtained after heating complex **8**.

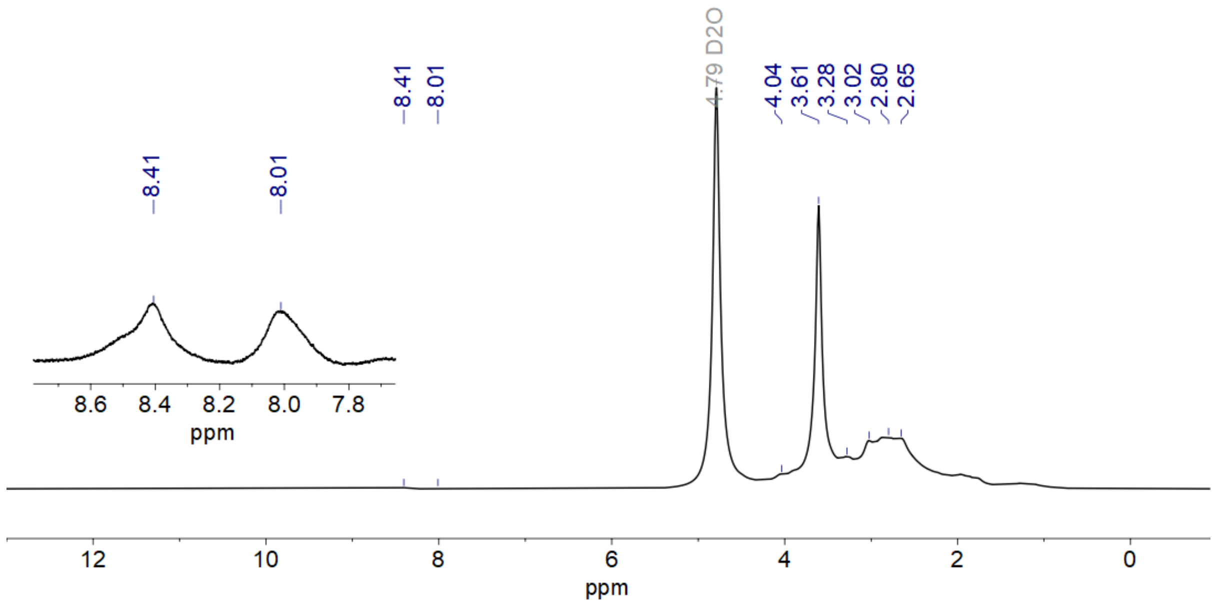

Figure S96 ^{1}H NMR (500 MHz, D_2O) spectrum of polymeric product obtained from reaction of ethylene glycol and ethylene diamine in the presence of **1** and KOtBu in a H-flask to probe the release of H_2 gas. Signal at δ_H 3.61 is residual ethylene glycol.

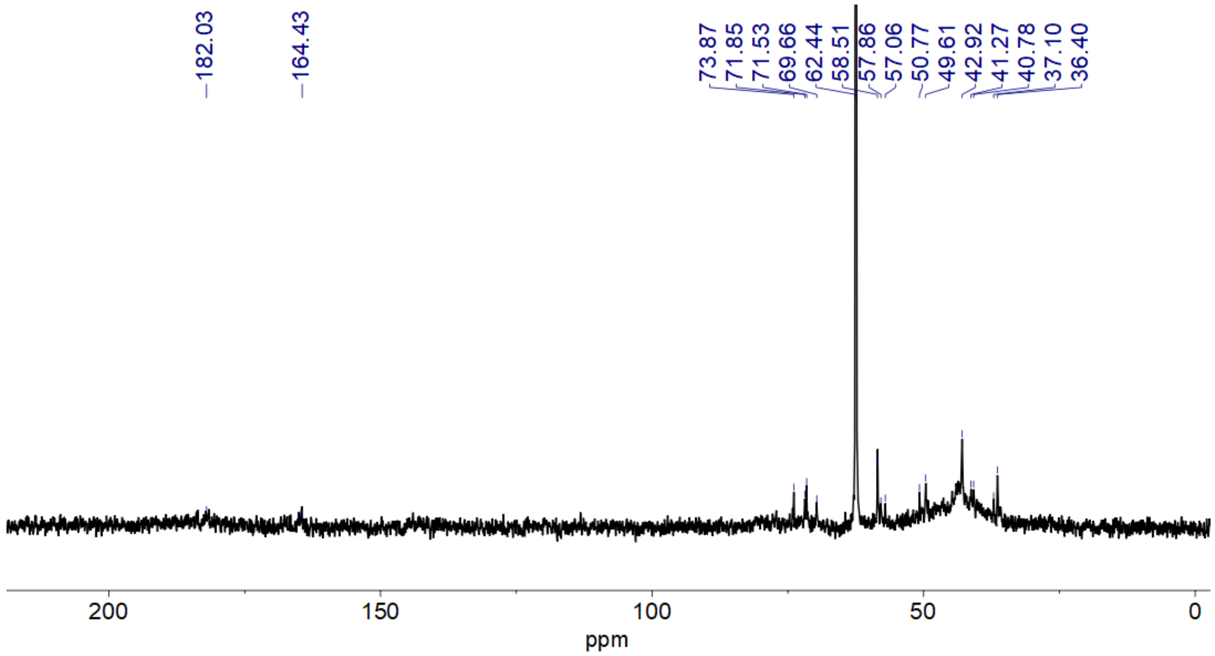

Figure S97 $^{13}C\{^1H\}$ NMR (126 MHz, D_2O) spectrum of polymeric product obtained from reaction of ethylene glycol and ethylene diamine in the presence of **1** and KOtBu in a H-flask to probe the release of H_2 gas. Signal at δ_H 62.4 is residual ethylene glycol.

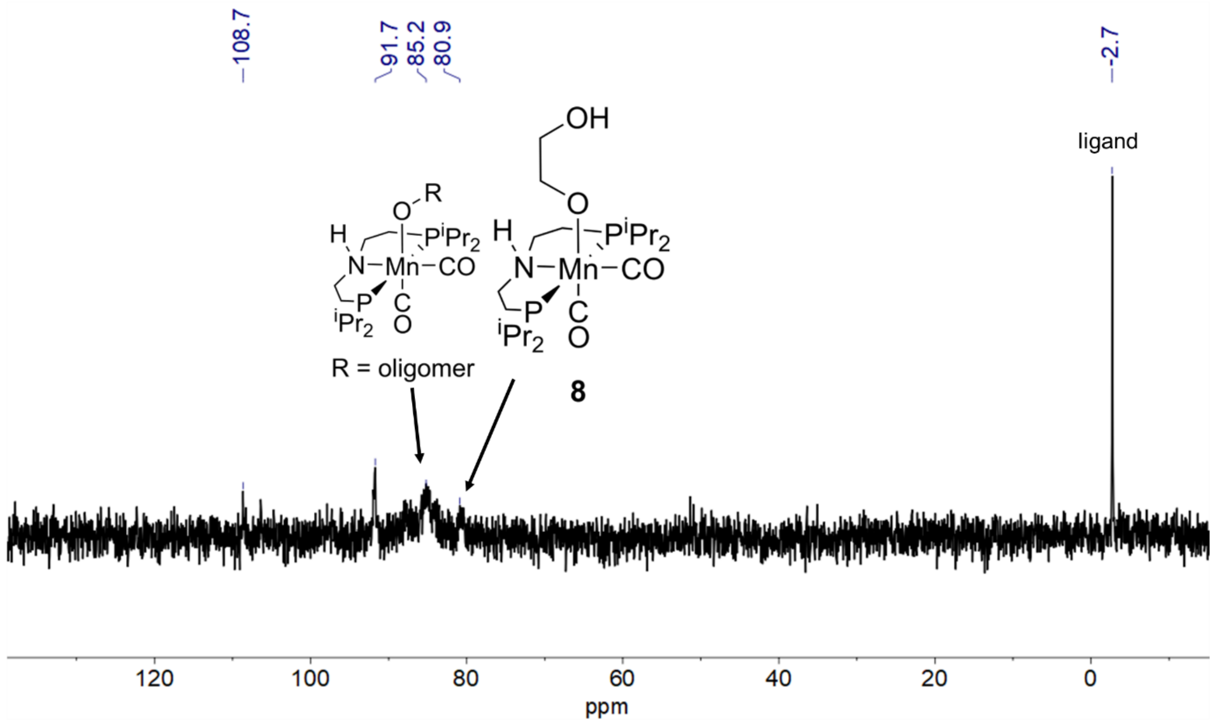

Figure S98 $^{31}P\{^1H\}$ NMR (203 MHz, toluene-H$_8$) spectrum of the reaction mixture of the coupling of ethylene diamine and ethylene glycol mediated by precatalyst, 1, after 1.5 hours at 150 °C.

1.5.6 GC-MS spectra

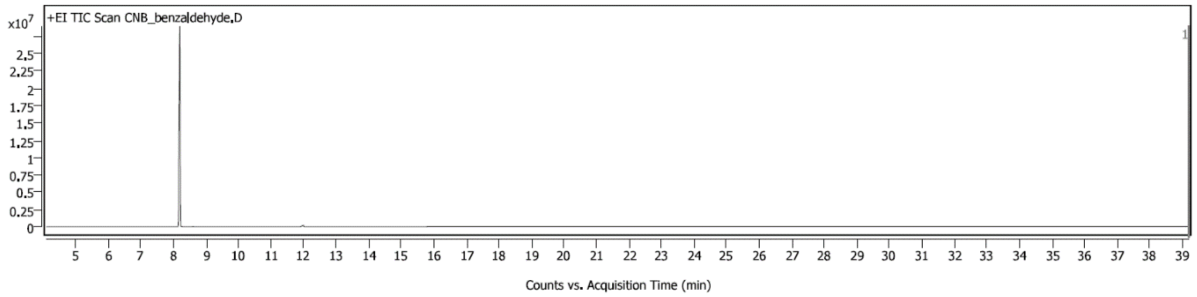

Figure S99 GC spectrum of benzaldehyde.

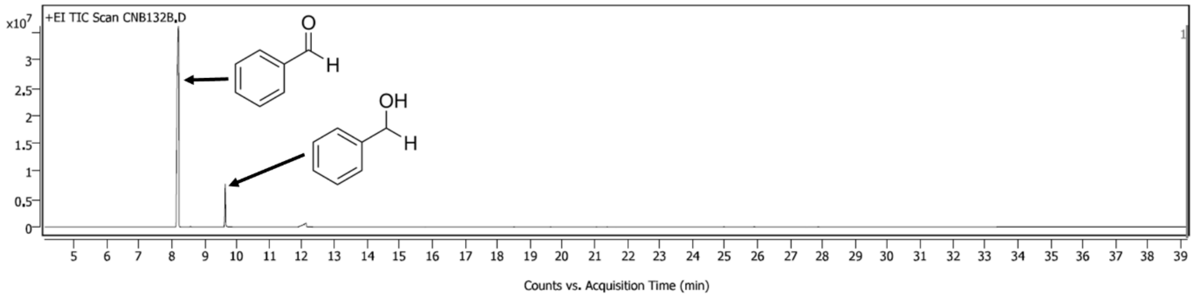

Figure S100 GC spectrum of product of reaction with benzaldehyde and Pd/C in the presence of *in situ* generated hydrogen gas from the reaction of ethylene glycol and ethylene diamine catalysed by **1**.

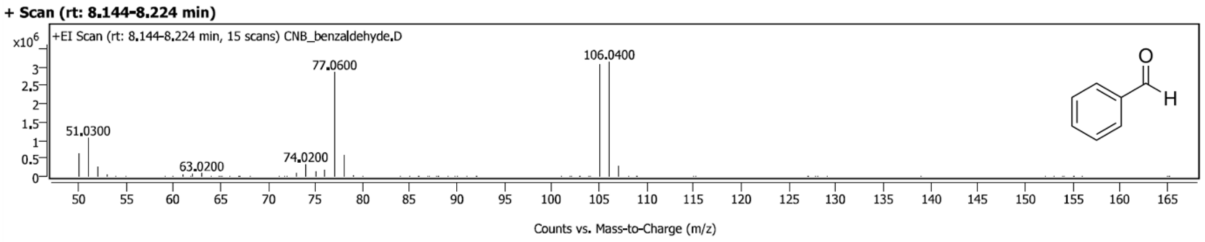

Figure S101 MS spectrum of benzaldehyde as observed within the product mixture of reaction with benzaldehyde and Pd/C in the presence of *in situ* generated hydrogen gas from the reaction of ethylene glycol and ethylene diamine catalysed by **1**.

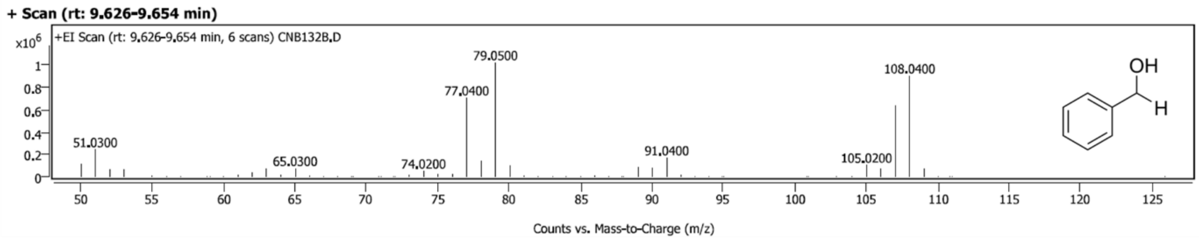

Figure S102 MS spectrum of benzylalcohol as observed within the product mixture of reaction with benzaldehyde and Pd/C in the presence of *in situ* generated hydrogen gas from the reaction of ethylene glycol and ethylene diamine catalysed by **1**.

2. Computational Details

DFT calculations were performed using Gaussian 16 series.[4] The generalised gradient approximation (GGA) functional BP86, that is composed of the Becke 1988 exchange functional[5] and the Perdew 86 correlation functional[6] and the hybrid Perdew–Burke–Ernzerhof exchange-correlation functional with 25% Hartree Fock exchange (PBE0)[7] were adopted for geometry optimisation calculations and single point calculations respectively. The def2-SVP and def2-TZVP of the Ahlrichs' family of basis set[8,9] were also employed as the basis set for geometry optimization calculations and single point calculations respectively. Geometry optimisation and single points calculations were performed with THF (ε=7.4257) as the solvent using the polarizable continuum model (PCM).[10] D3 dispersion corrections were included using Grimme's DFT-D3 correction[11] along with Becke-Johnson damping[12] only on the single-point calculations of our chosen level of theory. The effect of dispersion on the level of optimisation was not accounted for because transition state becomes very cumbersome to locate if included. The PBE0-D3[pcm,THF]/def2-TZVP//RI-BP86[pcm,THF]/def2-SVP level of theory adopted for this studies has already been reported from the benchmarking studies of the hydricity of three 3d metal complexes conducted in the Bühl group.[13] Minima and transition states were verified through evaluation of the harmonic vibrational frequencies (minima and transition states characterised through zero and one imaginary frequency, respectively). All stabilized adducts and intermediates were connected to their transition state via IRC.[14] The overestimation of the entropy contributions to the Gibbs free energy in solution have been corrected using the Martin, Hay and Pratt entropic correction[15] term. Based on the scheme proposed by Martin, Hay and Pratt, a correction term of 5.09 kcal/mol have been calculated for THF solvent at 423.15 K as shown below and have been applied to reaction terms with difference in molecularity, *i.e.* (n-m) * 5.09 kcal/mol.

$$\mathrm{p} = \frac{\rho RT}{M} = 427.40\, atm$$

$$S_{MHP} = RTIn\left(\frac{p}{p_0}\right) = 21312.7604\frac{J}{mol} = 5.09\; kcal/mol$$

ρ = 0.8876 g.cm^{-3} = 887.6 g.L^{-1}, R = 0.082057 L.atm.mol^{-1}.K^{-1} , R = 8.3145 J.mol^{-1}.K^{-1}, T = 423.15 K, M = 72.11 g.mol^{-1}, 1 Joule/Mol = 0.000238846 kcal/mol.

The Gibbs free energy values (ΔG) reported in the work at a temperature 423.15 K were recalculated from their corresponding ΔH and ΔS values obtained at 298.15 K.

The optimised Cartesian coordinates of all complexes of this study are available as separate Supporting Information file.

2.1 Thermodynamic driving forces for the various steps for the formation of G

Thermodynamic driving forces for the formation of dimethyl ethylene diamine (**G**) from ethylene glycol (**A**) and those of a number of conceivable intermediates on the way are summarised in the schematic off-metal profile in Scheme S1. Most of the possible intermediates have conformational or stereochemical flexibility. All possible gauche (g), anti (a), cis (c) and trans (t) conformers and isomers have been computed, but only the most stable one in each case is shown in the profile. The reaction sequence starts with the dehydrogenation of **A** to give the *cis*-conformer of glycol-aldehyde (**B**) with $\Delta G = 10.8$ kcal/mol. Methylamine is then added to **B** to afford the anti-conformer of *N*-methyl ethylene glycol (**C**), which is shown in the profile as a precursor for the formation of other intermediates along the profile. Interestingly, although the formation of ketene from **B** *via* dehydration is endothermic with $\Delta H = 8.9$ kcal/mol, this step is favoured by entropy to the extent that ketene formation could become competitive at the high temperatures employed here ($\Delta G = -1.8$ kcal/mol). Free ketene would be expected to rapidly add amine, affording ***cis*-NMA**, which is the most stable of all intermediates studied here.

Three intermediates namely: *trans*-methyl imine ethanol (**D1**), *cis*-methylamine ethenol (**D4**) and anti-methylamine ethenol (**aMAE**), were obtained from the dehydration of **C**. **aMAE** is indicated to be the lowest with a driving force for dehydration of just $\Delta G = -8.7$ kcal/mol (relative to **C**). This intermediate (**aMAE**) can rearrange *via* keto-enol tautomerism, affording another entry into ***cis*-NMA.** Starting from these three key intermediates, namely **D4**, **D1** and ***cis*-NMA**, three distinct pathways are conceivable, labelled **I**, **II** and **III** in Scheme S1**.** Pathway **I** proceeds *via* keto-enol tautomerism (expected to be rapid) from **D4** to *cis*-methylamine aldehyde (**D3**), which can add amine to give a diamine intermediate, dimethyl diamine ethanol (**E**). This can be transformed into the final product, **G**, through two consecutive dehydration/hydrogenation steps involving isomeric intermediates: *cis*-dimethyl ethene diamine (**F2**) and *trans-N*-methyl imine ethane (**F1**). Pathway B is seen to proceeded through a low-lying intermediate, anti-methylamine ethanol (**D2**), which can be dehydrogenated to **D3** on Pathway **I**. An alternate route is conceivable starting from **D1**, where hydrogenation and dehydrogenation steps are reversed (labelled **IIa** in Scheme S1), but this involves high energy intermediates, in particular *trans*-methyl imine aldehyde (***trans*-MIA**), which is obtained by dehydrogenation of **D1** and over 20 kcal/mol higher in free energy than the reactants (viz. **A**). Pathway **III** proceeds *via* hydrogenation of the carbonyl group of ***cis*-NMA**, followed by dehydration (*via* **NMEM**) and amine addition to yield **G**. The highest-lying intermediate on all pathways **I - III** is **B**, with a free energy of $\Delta G = 10.8$ kcal/mol above the entry point (**A**). The **cHMA** intermediate is accessible from the dehydrogenation of **C** but this does not connect to any other step leading to **G**.

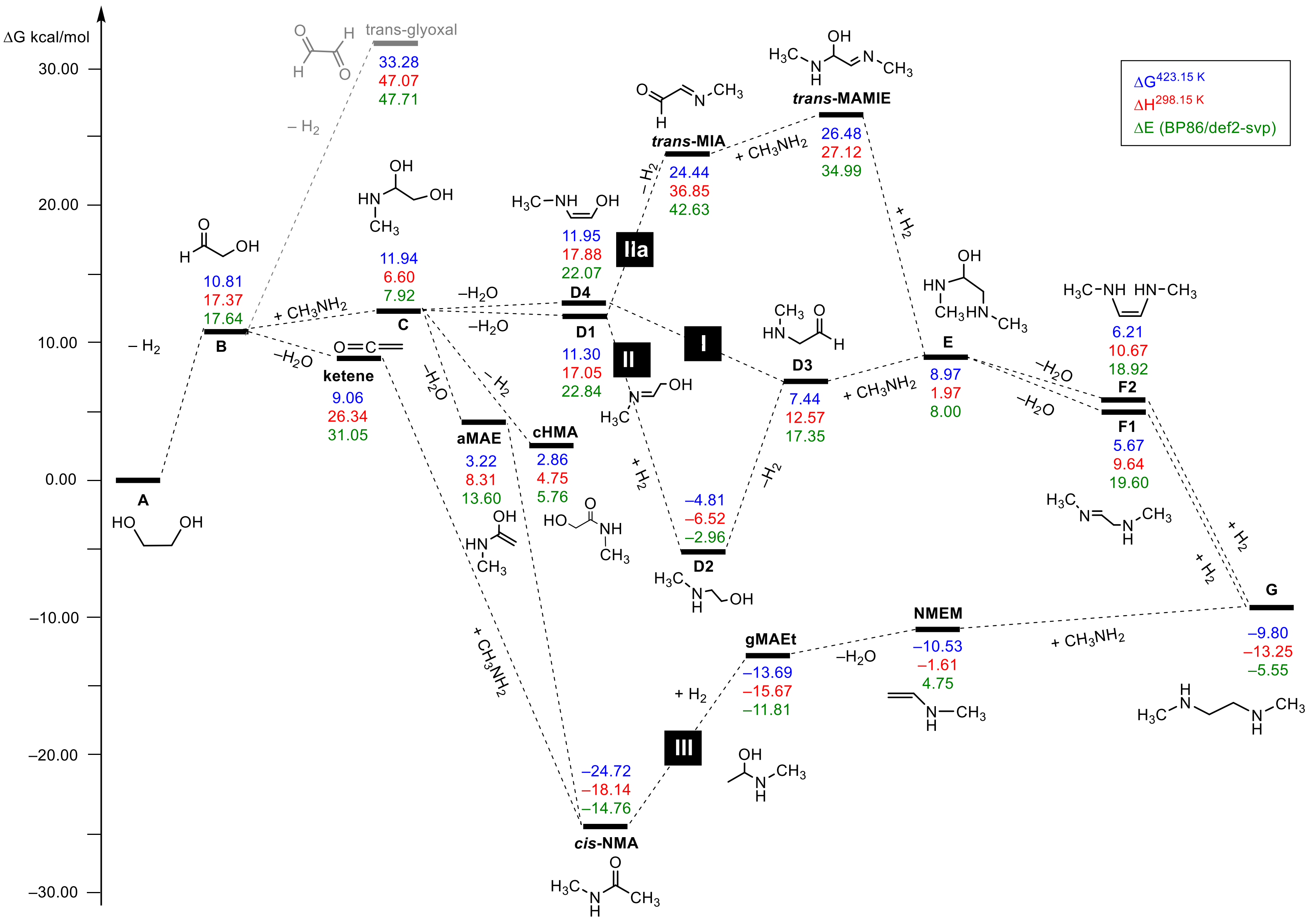

Scheme S1 Thermodynamic driving forces for the various steps for the formation of dimethyl ethylenediamine (G) from ethylene glycol (A) at the PBE0-D3[pcm,THF]/def2-TZVP//RI-BP86[pcm,THF]/def2-SVP level of theory.

2.2 Thermodynamics for the formation of a cyclic product and dehydration step in the absence of a catalyst

Considering the possibility of the formation of cyclic side products with piperazine motifs, selected intermediates along a possible route to our corresponding model, dimethylpiperazine (**DMPRZ**) have been explored in Scheme S2. This assumes formation of enamine, dimethylethylene diamine ethanol (**DMEDE)** from reaction between **G** and the aldehyde **B** obtained by dehydrogenation of **A**, keto-enol tautomerisation, another intramolecular enamine formation *via* dimethyl-tetrahydropyrazine (**DMTPZ)** and hydrogenation of the latter. A large thermodynamic driving force for formation of **DMPRZ** from **G** is predicted, ΔG = –21.7 kcal/mol. Apart from the last hydrogenation step (*vide infra*), no serious kinetic hindrance is expected for the steps involved in this process, and the highest intermediate is **DMEDE**, ΔG = 13.7 kcal/mol above **G**. In contrast, dehydrogenation affording the diimine, dimethylethane diimine (**DMEDI)** is predicted to be much less favourable. Similarly, formation of the diketone intermediate, ***trans*-glyoaxal**, formed from the dehydrogenation of **B** is highly unfavourable.

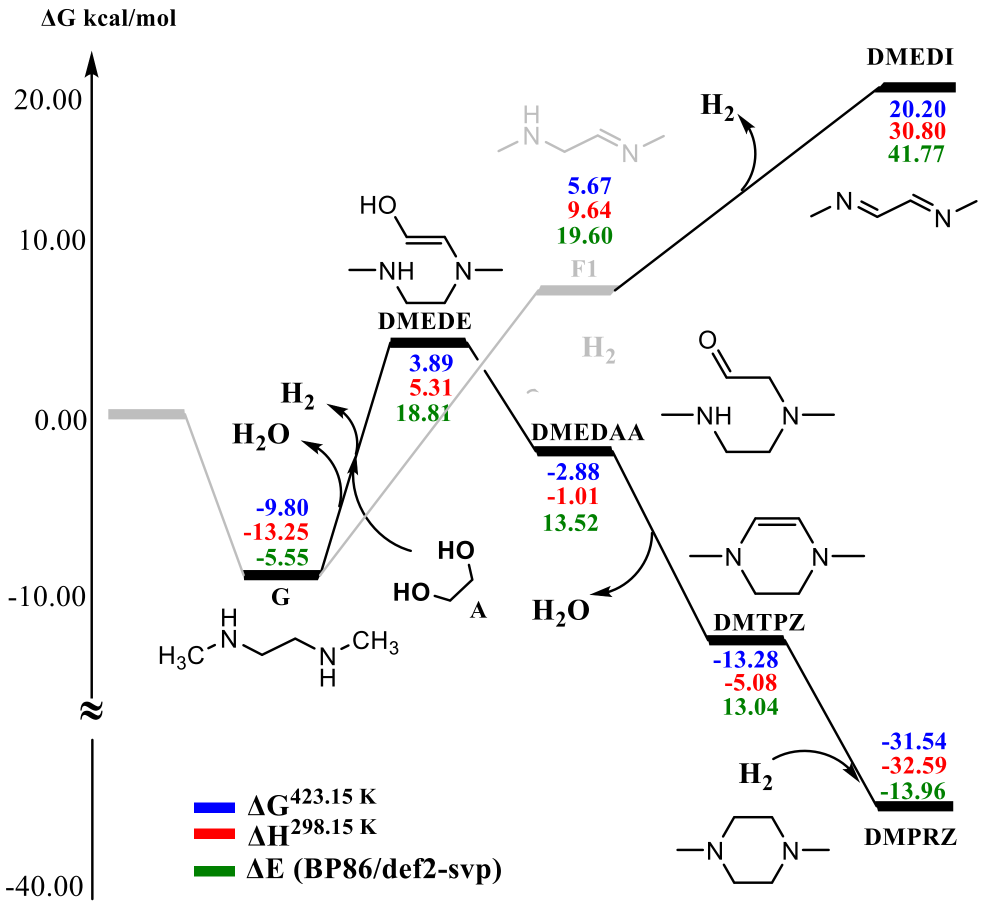

Scheme S2 Thermodynamic driving forces for the formation of cyclic primary amine (piperazine) and imine intermediates at the PBE0-D3[pcm,THF]/def2-TZVP//RI-BP86[pcm,THF]/def2-SVP level of theory.

Full appraisal of the feasibility of these pathways would require knowledge of the activation barriers of all steps involved.

2.3 Metal-free Transition States for the formation of D4 and D1

Addition of amines to carbonyl compounds (*e.g.* formation of **C** from the reaction of **B** and methylamine) can be catalysed by traces of acids or bases and this type of reaction can proceed without significant kinetic hinderance.[16,17] From our previous studies with MeOH as substrate,[16] (de)hydrogenation reactions involving carbonyl or imine groups are expected to be efficiently catalysed by the Mn complex **6**.

Hydration and dehydration reactions are expected to require catalysis as well. This is illustrated by selected barriers for uncatalysed H_2O elimination from **C** *via* the transition states **D4-TSb** and **D1-TSa** ($\Delta G^{\ddagger}$ = 59.4 kcal/mol and 58.4 kcal/mol, respectively, relative to **A**, as seen in Scheme S3). Established in literature,[18] the strain in such four-membered transition states can be alleviated by the involvement of protic substrates acting as proton relays. Here, the involvement of a single water molecule is indeed indicated to reduce the barriers by ~20 kcal/mol, with $\Delta G^{\ddagger}$ = 38.5 kcal/mol and 39.8 kcal/mol, relative to **A** (*via* **D4-TSb-H_2O** and **D1-TSb-H_2O,** respectively). This is consistent with previous reports by Poater, where the energy barrier to dehydration decreases from 54.2 kcal/mol to 39.5 or 34.1 kcal/mol when assisted by two water or benzyl alcohol molecules, respectively.[18] These steps, **D4-TSb-H_2O** and **D1-TSa-H_2O,** remain too high for efficient turnover. Hence, the potential involvement of Mn complex **6** in this kind of transformation is explored further below.

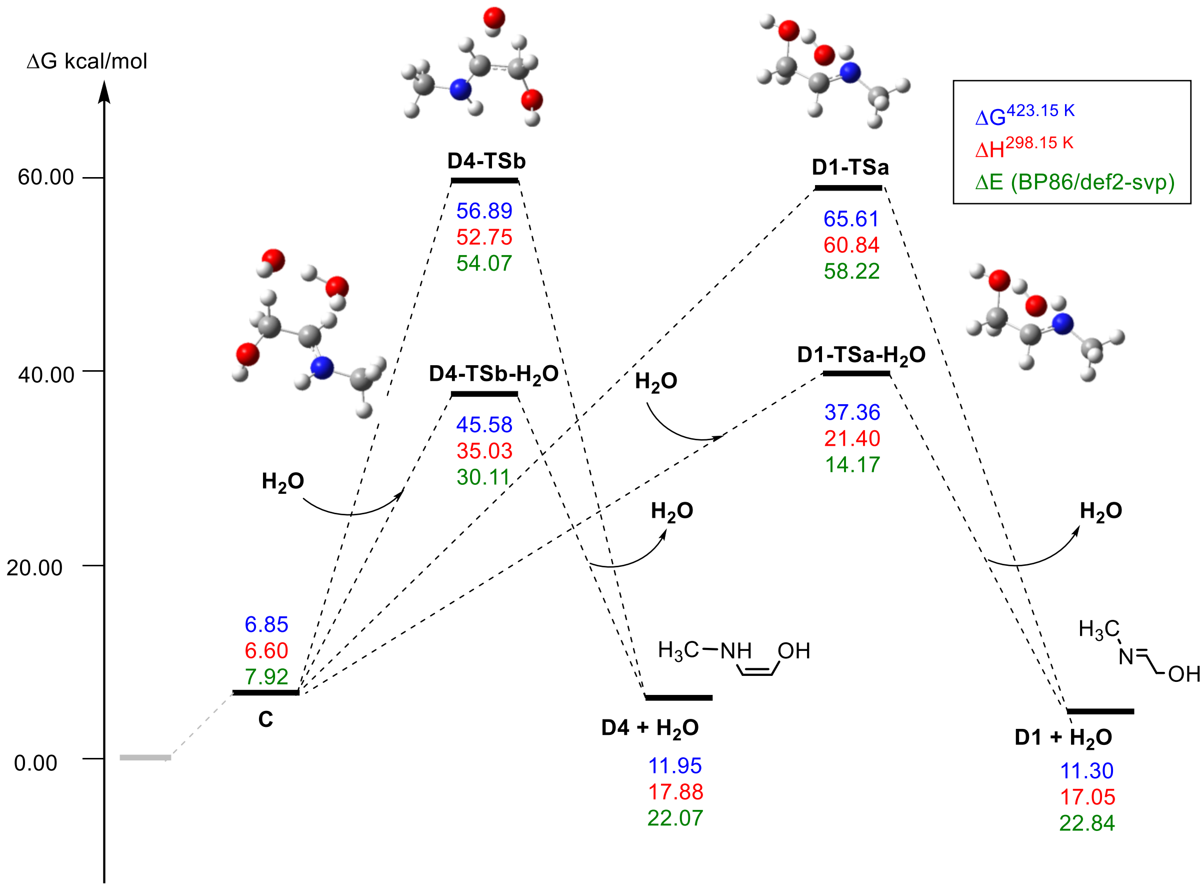

Scheme S3 Metal-free transition states for the formation of *cis*-methylamine ethenol (**D4**) and trans-methyl imine ethanol (**D1**) at the PBE0-D3[pcm,THF]/def2-TZVP//RI-BP86[pcm,THF]/def2-SVP level of theory.

2.4 Thermodynamic driving forces for Branched oligomer and Linear oligomer

Similar to the reaction pathway proposed for the formation of **G**, where methylamine is added to glycolaldehyde, **B,** to give a glycol intermediate, **C**, we propose a similar route for the formation of branched oligomer (**b-oligomer**) and linear oligomer (**l-oligomer**) through reaction of ethanediamine (**EDA**) with **B** to give the diaminoglycol product, 1-(2-aminoethylamino)ethane-1,2-diol (**AAEG**) similar to **C**. This is followed by the dehydration of **AAEG** to give the amino(imine) ethanol product, (*E*)-2-(2-aminoethylimino)ethan-1-ol (***trans*-AIE**). A similar step that involves addition of **EDA** and subsequent removal of water molecule leads to the formation of imine product, (*E*)-*N*-(2-((2-aminoethyl)imino)ethyl)ethane-1,2-diamine, **AIEED**. Low lying intermediates, amino(amino) ethanol (2-(2-aminoethylamino)ethanol (**AAE**)) and *N*,*N'*-(ethane-1,2-diyl)*bis*(ethane-1,2-diamine) **EDBED**, are obtained from the hydrogenation of the imine products, ***trans*-AIE** and **AIEED** (Scheme S4a). As with the dehydrogenation of ethylene glycol, dehydrogenation of **AAE**, affords the aldehyde, 2-(2-aminoethylamino)acetaldehyde (**AAAc**). A close look at the thermodynamic driving forces of the intermediates involved in the formation of **G** in Scheme S1 in comparison to those reported in Scheme S4a leading to the formation of **EDBED**, show that **G** is a more stable intermediate than **EDBED**.

Starting from **EDBED**, the addition of glycolaldehyde **B** would give rise to two isomers; the branched and the linear form of amino(ethane)diols, **b-4AED** and **l-4AED**, respectively. The dehydration of these isomers affords the olefin isomers, amino(ethane)ethenol; **b-4AEE** and **l-4AEE** for the branched and linear form respectively. Amino(imine)ethenol (**l-AIE**) isomer is also possible from **l-AED** and appears to be more stable than its olefin counterparts. Finally, the branched oligomer (**b-oligomer**) and linear oligomer (**l-oligomer**) can be obtained by hydrogenation of either the branched or linear olefin isomers or the imine isomer. From our computation, b-oligomer appears to be slightly more stable when compared to l-oligomer (Scheme S4b).

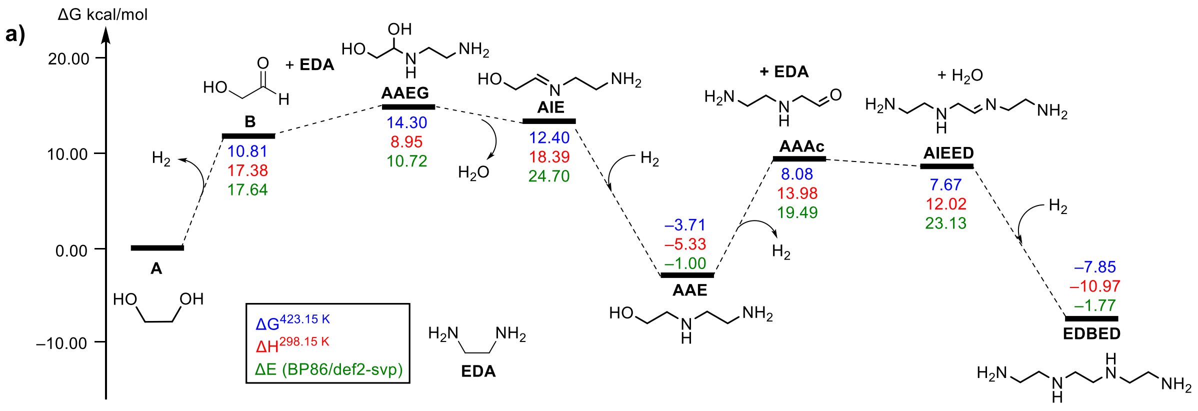

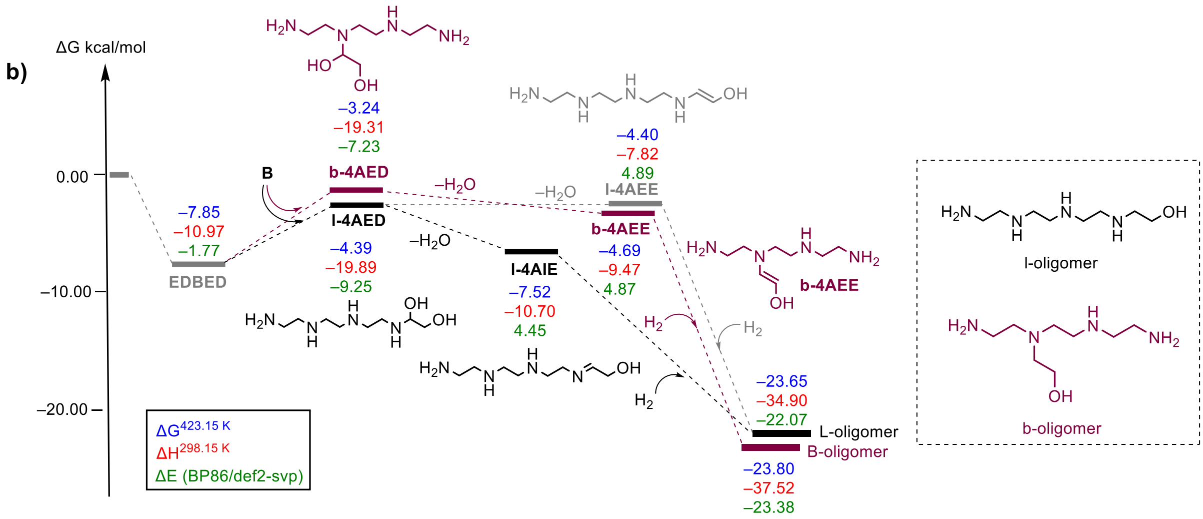

Scheme S4 **A**)Thermodynamic driving forces for the various steps leading to the formation of EDBED intermediate at the PBE0-D3[pcm,THF]/def2-TZVP//RI-BP86[pcm,THF]/def2-SVP level of theory; **B**) Thermodynamic driving forces for the various steps leading to the formation of branched and linear oligomer products at the PBE0-D3[pcm,THF]/def2-TZVP//RI-BP86[pcm,THF]/def2-SVP level of theory.

2.5 Dehydrogenation of ethylene glycol

The overall catalytic dehydrogenation of ethylene glycol is indicated to be more facile than that of methanol with ΔG = 10.8 kcal/mol and the sequence of elementary steps leading to the regeneration of the active catalyst **6** is shown in Scheme S5.

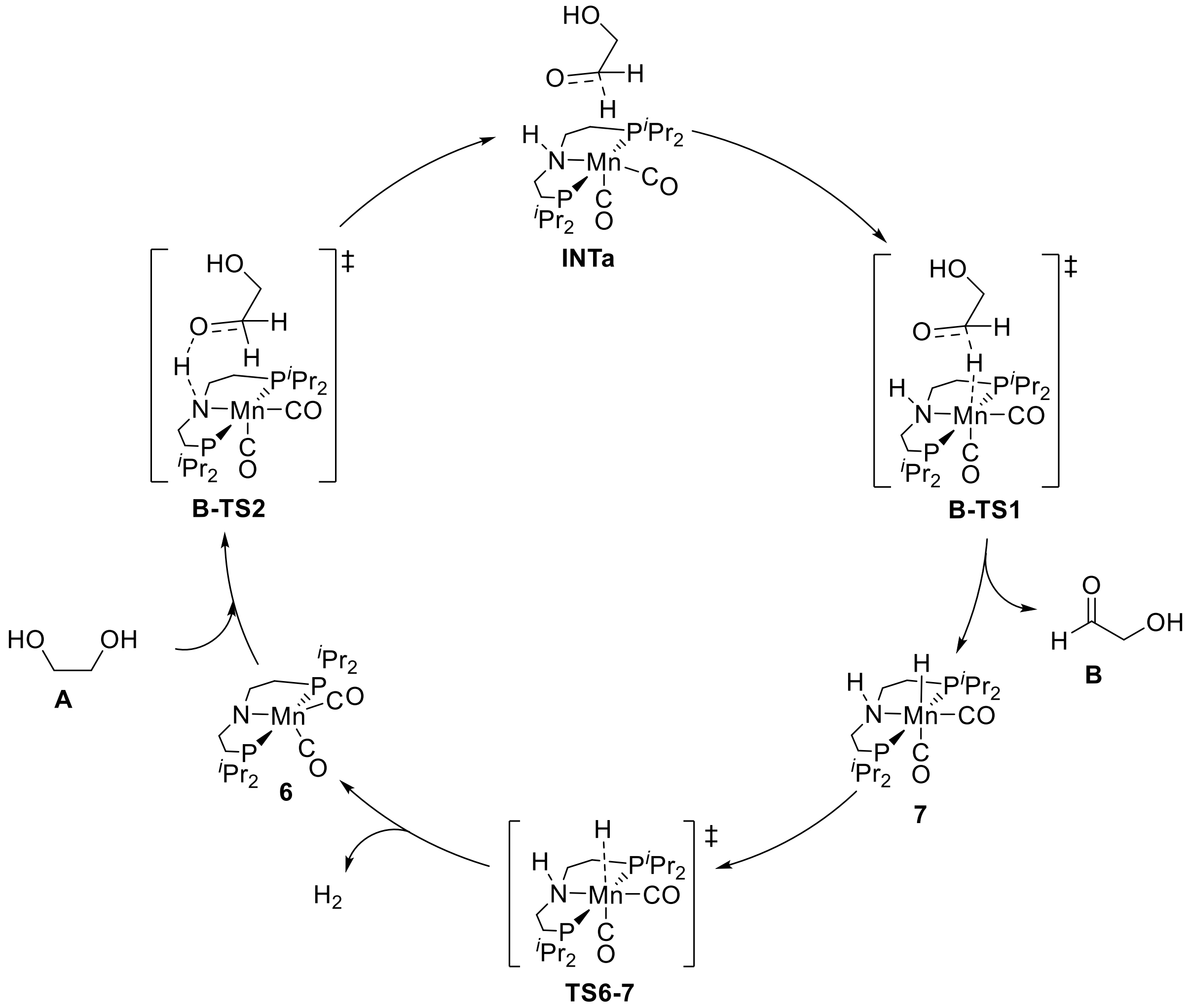

Scheme S5 Alternative pathways for the dehydrogenation of ethylene glycol (**A**) to form glycol aldehyde (**B**) catalysed by complex **6**, as emerging from our DFT calculations.

Another dehydrogenation step for which Mn-catalysis has been explored is that from **D2** to **D3** (Scheme S6). Again, this is computed to be a facile process with very similar energetics as that for the dehydrogenation of **A**, except that no zwitterionic intermediate akin to **INTa** is found and the H-transfer is indicated to be concerted rather than stepwise. When the cost for regenerating the catalyst is included, the overall barrier for dehydrogenation of **D2** is $\Delta G^{\ddagger}$ = 31.34 kcal/mol compared to that of **A** where $\Delta G^{\ddagger}$ = 30.31 kcal/mol has been found. As expected, the regeneration of the active catalyst **6** and the overall barrier for the process correspond to **TS6-7** with $\Delta G^{\ddagger}$ = 31.34 kcal/mol.

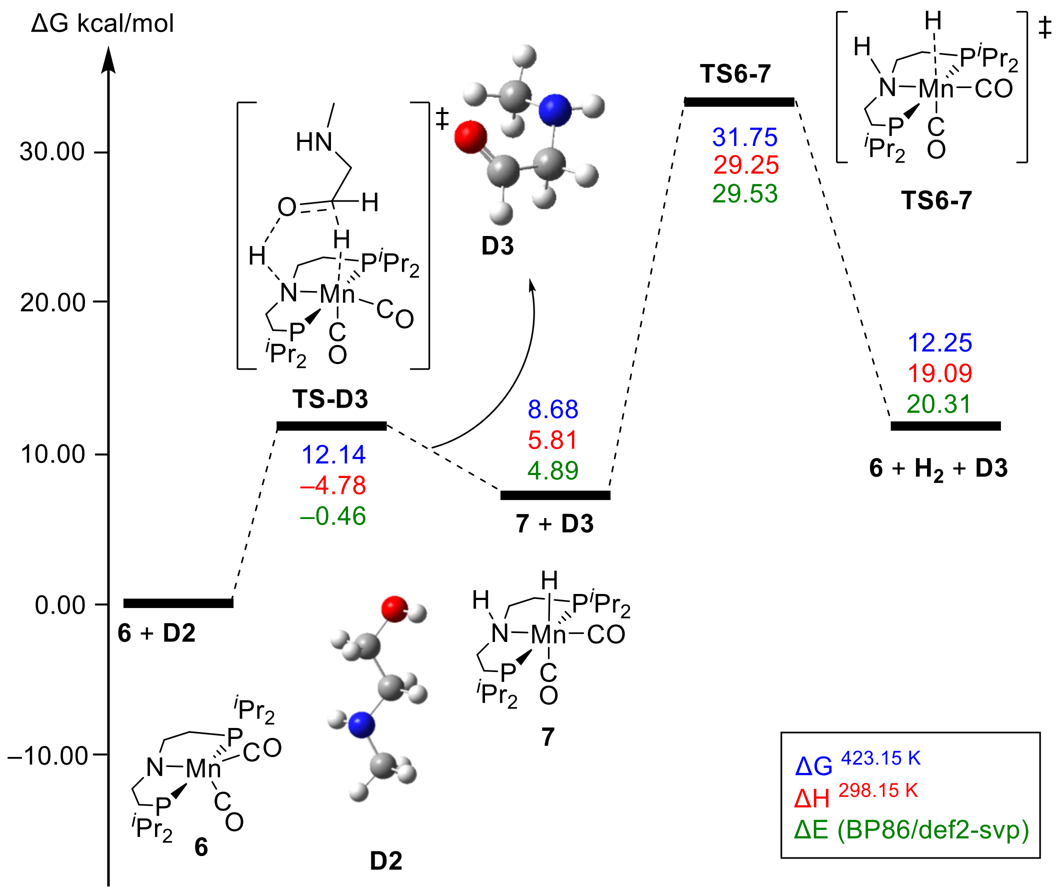

Scheme S6 Free energy profiles of the proposed pathways for dehydrogenation of **D2** to give methylamine aldehyde, **D3** at the PBE0-D3[pcm,THF]/def2-TZVP//RI-BP86[pcm,THF]/def2-SVP level of theory.

2.6 Steps involving dehydration

At our level of theory, the activation of water molecule on **6** to give **9** is computed to be exergonic by ΔG = –0.59 kcal/mol (see Scheme S7), in good agreement with recent experimental[19]and computational[20]results. Very low barriers have been inferred for this process in these previous studies (ΔG‡ = 13.1 – 7.9 kcal/mol at room temperature).

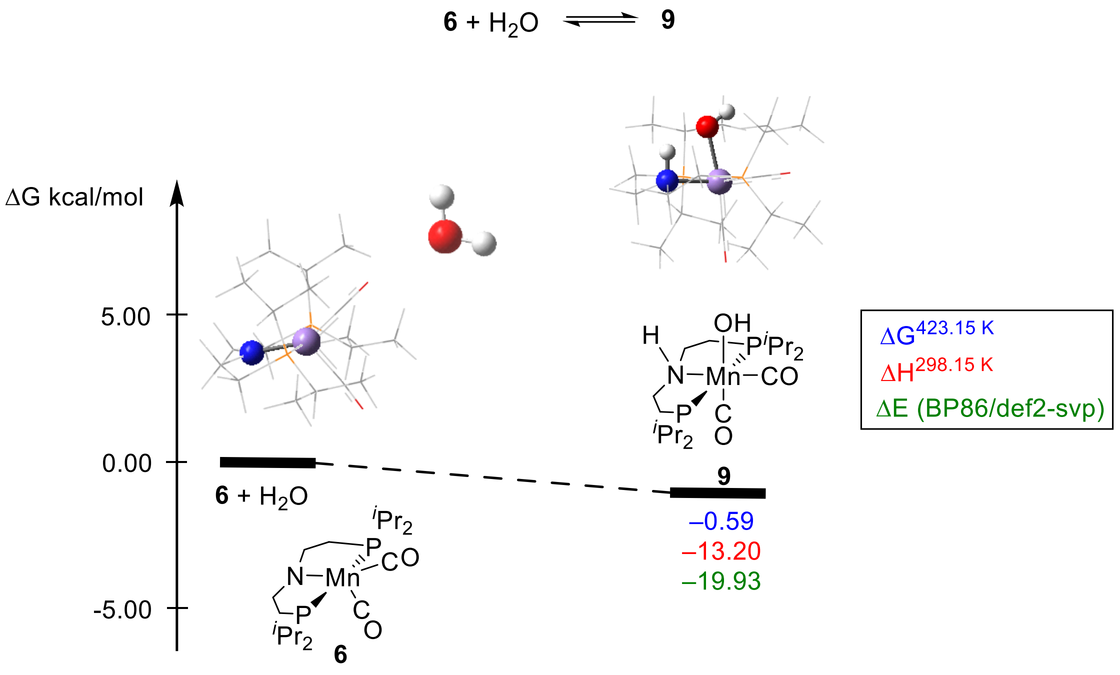

Scheme S7 Driving force for the catalytic activation of water molecule on active catalyst **6** to give the hydrated catalyst **9** at the PBE0-D3[pcm,THF]/def2-TZVP//RI-BP86[pcm,THF]/def2-SVP level of theory.

Traces of water may be bound by the active catalyst in the form of **9**, which then can readily release the active catalyst **6** *via* equilibrium. The same intermediate, **9**, could be formed though dehydration of a saturated alcohol.

We first consider a system where a polar double bond is formed, using **C**, as representative example. The dehydration of **C** across the CN bond affords **D1,** where the transfer of -OH group and N(H) proton to the Mn and N-atoms of the catalyst occur in a concerted fashion to give the separated products at ΔG = –1.23 kcal/mol. The regeneration of the active catalyst, **6**, occurs *via* a barrierless step that is slightly endergonic by ΔG = 0.59 kcal/mol relative to **9** + **D1** (Scheme S8). The overall barrier for the formation of the imine product, **D1** is computed to be ΔG‡ = 26.35 kcal/mol *via* **D1-TS**. While this barrier is significantly higher than, for example, that for dehydrogenation of ethylene glycol (ΔG‡ = 17.0 kcal/mol *via* **B-TS1**), Mn catalysis of this type of dehydration could still be feasible at the high temperature of the experiment. This is different from a ruthenium PNP complex where barrier of dehydration was slightly higher in the presence of catalyst.[21] However, looks consistent with that of Sola and Poater.[18,22]

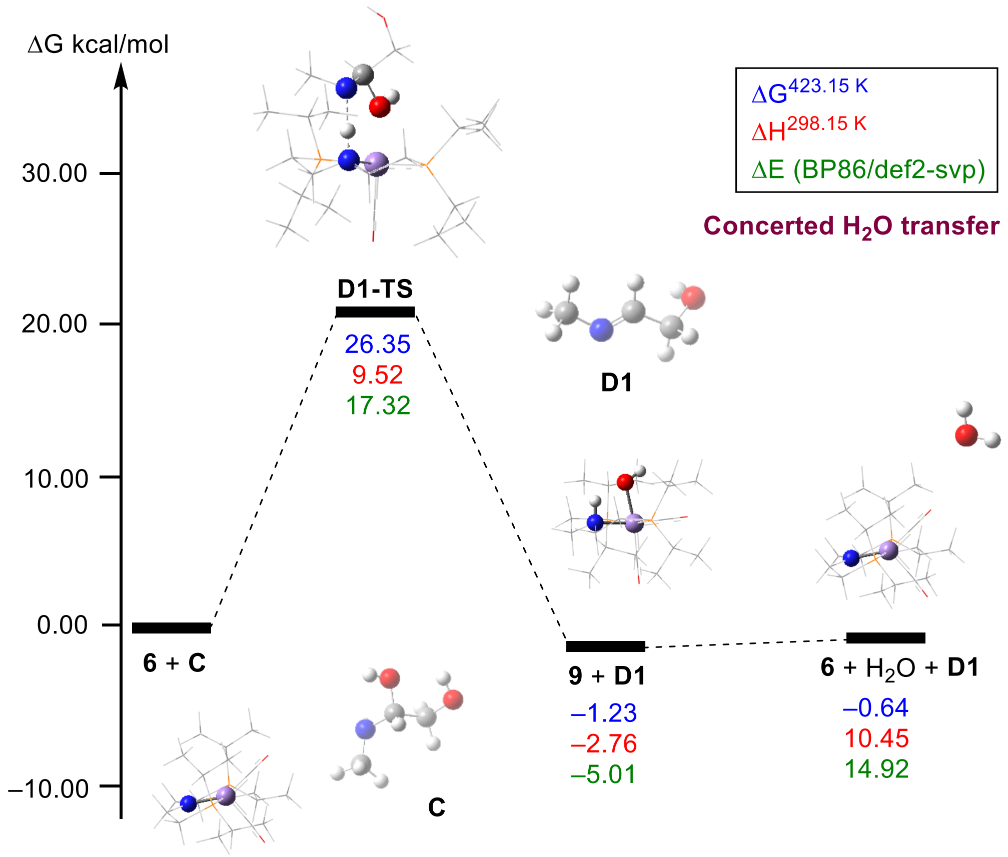

Scheme S8 Free energy profiles for the proposed pathways for dehydration of **C** to give trans imine product, **D1** at the PBE0-D3[pcm,THF]/def2-TZVP//RI-BP86[pcm,THF]/def2-SVP level of theory.

C can also be dehydrated to form an isomeric enol product, **D4** with a non-polar (C=C) double bond. Even though both dehydrated products are very similar in their thermodynamic stability (with predicted driving forces for formation from **C** of ΔG = –0.59 and 0.01 kcal/mol for **D1** and **D4**, respectively), the computed activation barriers are very different. The barrier for formation of **D4** via **D4-TS** is $\Delta G^{\ddagger}$ = 41.78 kcal/mol (Scheme S9), much higher than that of forming **D1** *via* **D1-TS** ($\Delta G^{\ddagger}$ = 26.35 kcal/mol in Scheme S8). Since it has been reported that catalytic hydrogenation with Mn(I) catalysts is more difficult for non-polar C-C multiple bonds than for polar C-X multiple bonds (where X = O, N),[23] it may be expected that the same will be the case for catalytic (de)hydration. This assumption can be rationalised from our DFT computations for the kinetic barriers leading to the formation of the two intermediates, **D1** and **D4** from **C.**

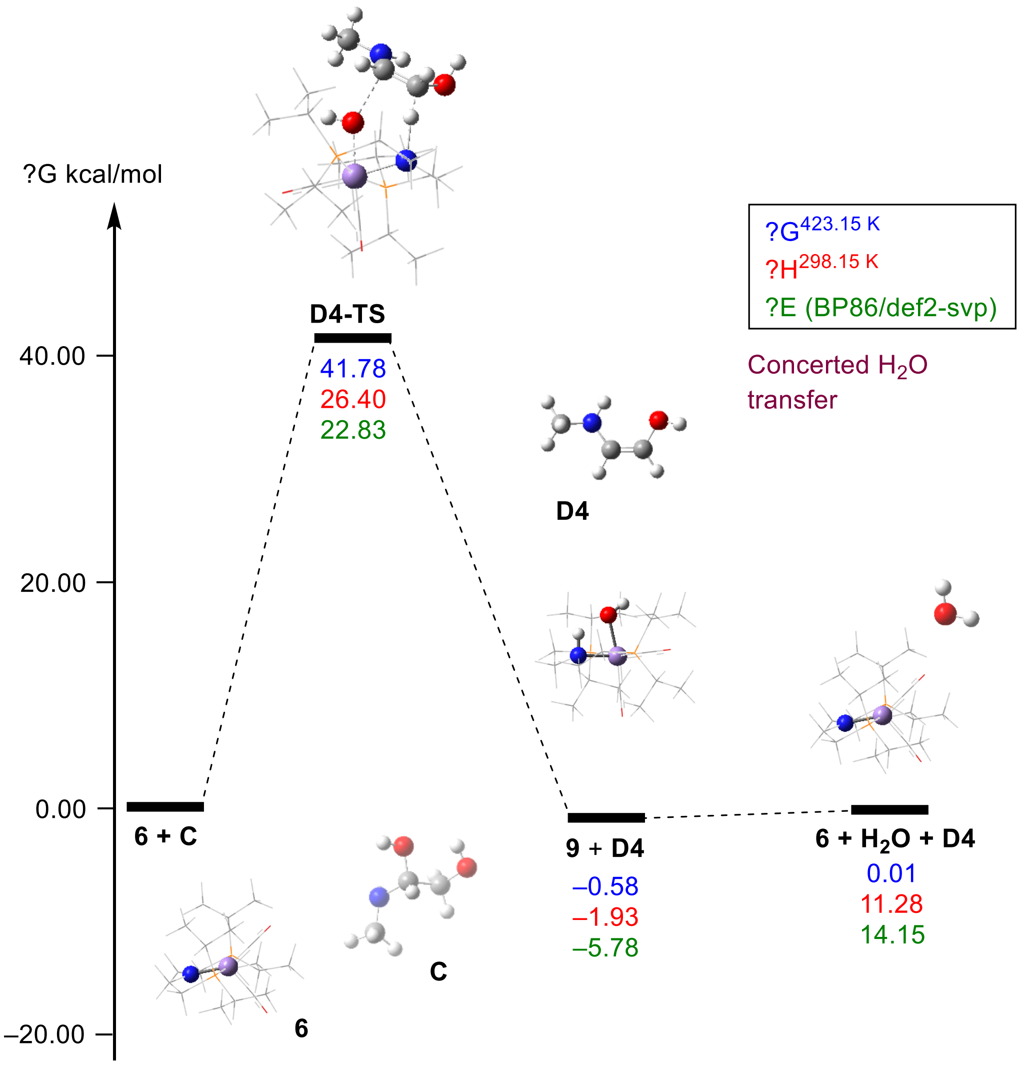

Scheme S9 Free energy profiles for the proposed pathways for dehydration of **C** to give *cis*-methylamine ethanol, **D4** at the PBE0-D3[pcm,THF]/def2-TZVP//RI-BP86[pcm,THF]/def2-SVP level of theory.

Very similar results are obtained for Mn-catalysed dehydration of another, later intermediate, namely **E**, which can give *N*-methyl imine ethane (**F1**) with a C=N double bond, or **F2** with a C=C double bond. The barrier for formation of the former polar bond ($\Delta G^{\ddagger}$ = 25.09 kcal/mol, Scheme S10) is much lower than that for formation of the latter, unpolar bond ($\Delta G^{\ddagger}$ = 36.78 kcal/mol, Scheme S11).

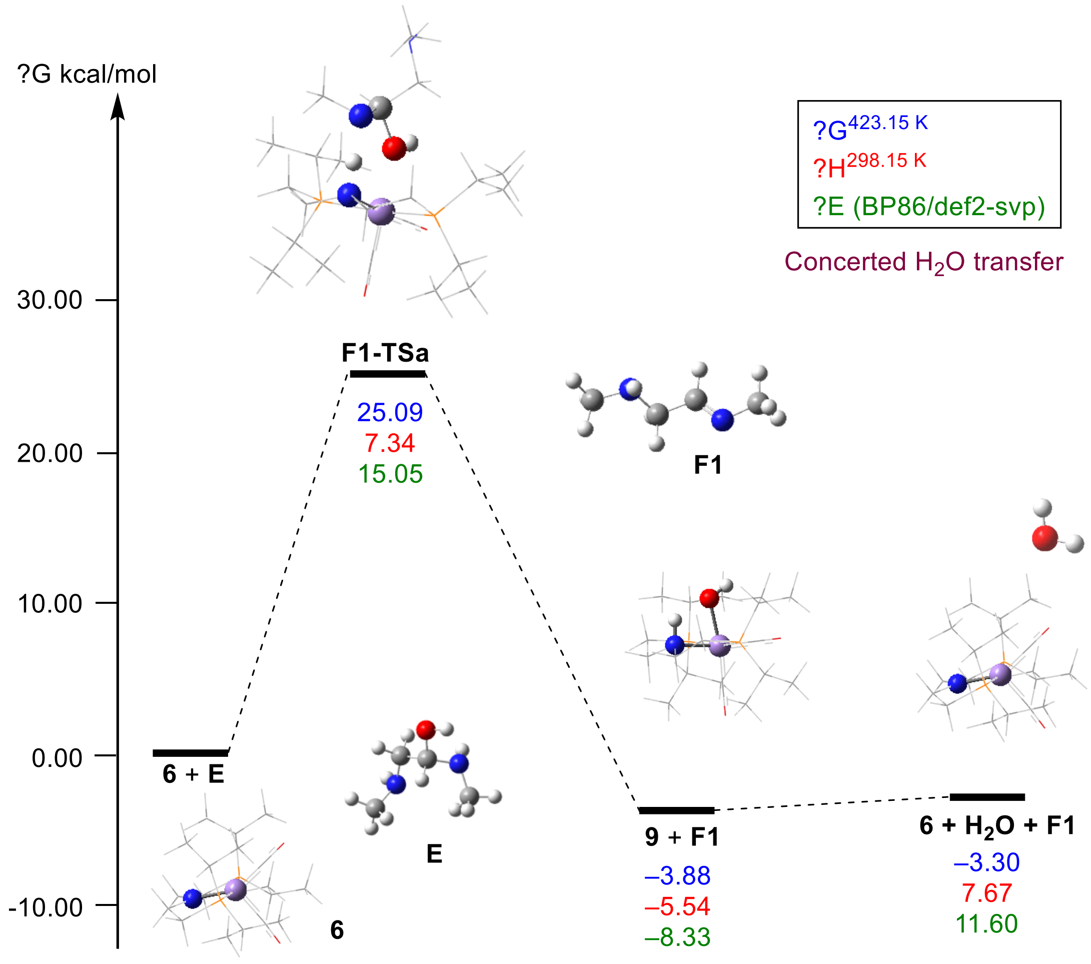

Scheme S10 Free energy profiles of the proposed pathways for dehydration of **E** to give *N*-methyl imine ethane (**F1**) at the PBE0-D3[pcm,THF]/def2-TZVP//RI-BP86[pcm,THF]/def2-SVP level of theory.

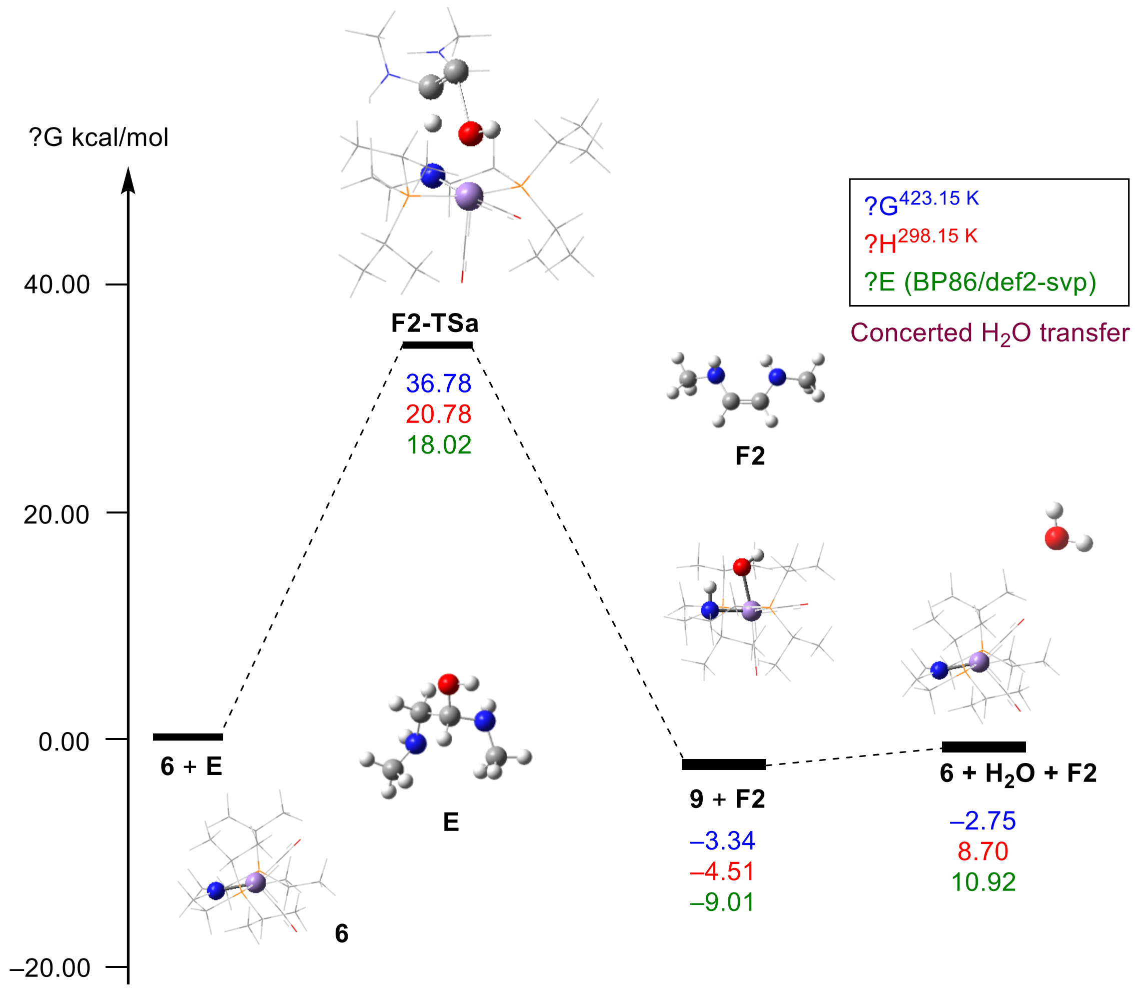

Scheme S11 Free energy profiles of the proposed pathways for dehydration of **E** to give dimethyl ethene diamine (**F2**) at the PBE0-D3[pcm,THF]/def2-TZVP//RI-BP86[pcm,THF]/def2-SVP level of theory.

2.7 Hydrogenation of imine and olefin intermediates

Hydrogenations of carbonyl groups affording alcohols (*e.g.* ***cis*-NMA** to **gMAE** in Scheme S1) are the reverse reactions of dehydrogenation steps discussed above and are known to be catalysed by complexes such as **6**. Again, our previous studies with MeOH as substrate,[16] (de)hydrogenation reactions involving carbonyl or imine groups are expected to be efficiently catalysed by the Mn complex **6.** At our level, the key steps leading to the formation of **G** by hydrogenation of the imine, **F1** is shown in Scheme S12 and **G** obtained by hydrogenation of alkene, **F2** is represented in Scheme S13. As show, the barrier is significantly higher for the dehydrogenation of alkene when compared to imine.

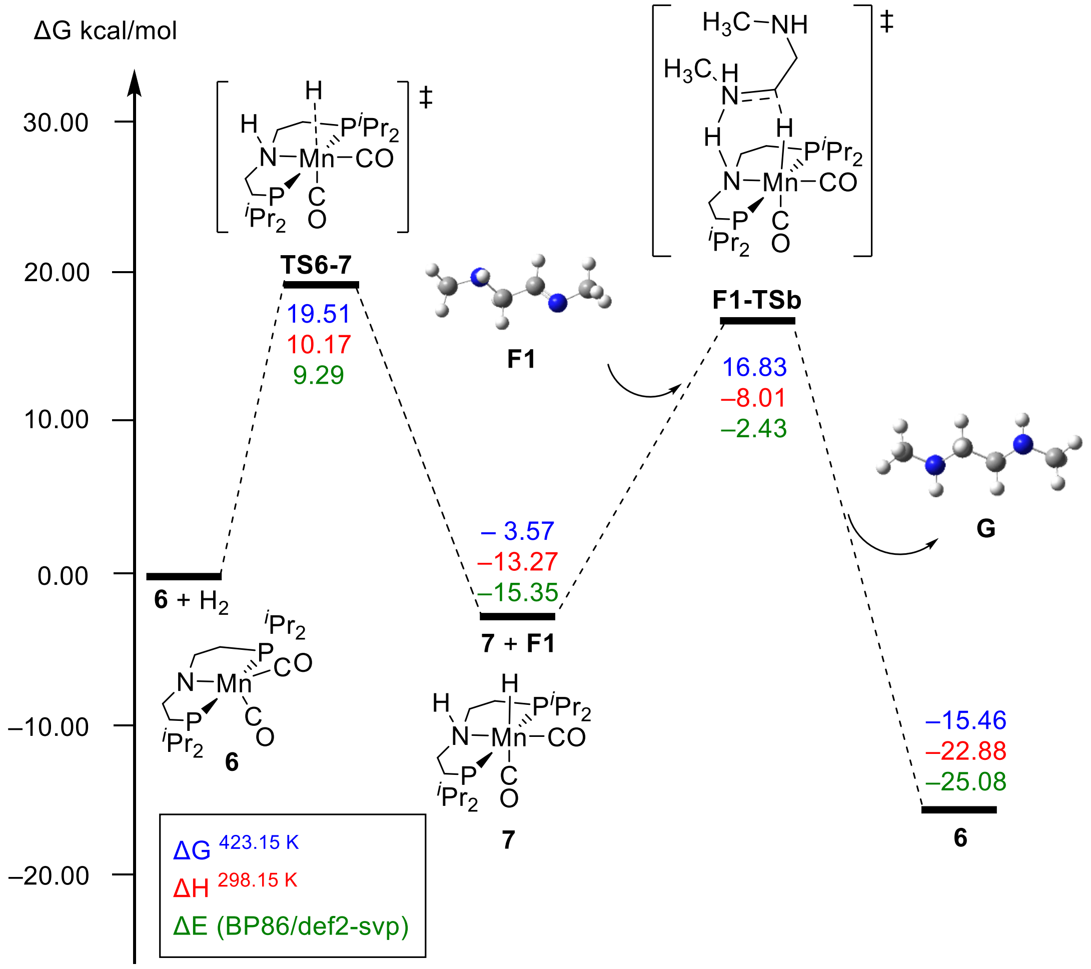

Scheme S12 Free energy profiles of the proposed pathways for dehydrogenation of **F1** to give G at the PBE0-D3[pcm,THF]/def2-TZVP//RI-BP86[pcm,THF]/def2-SVP level of theory.

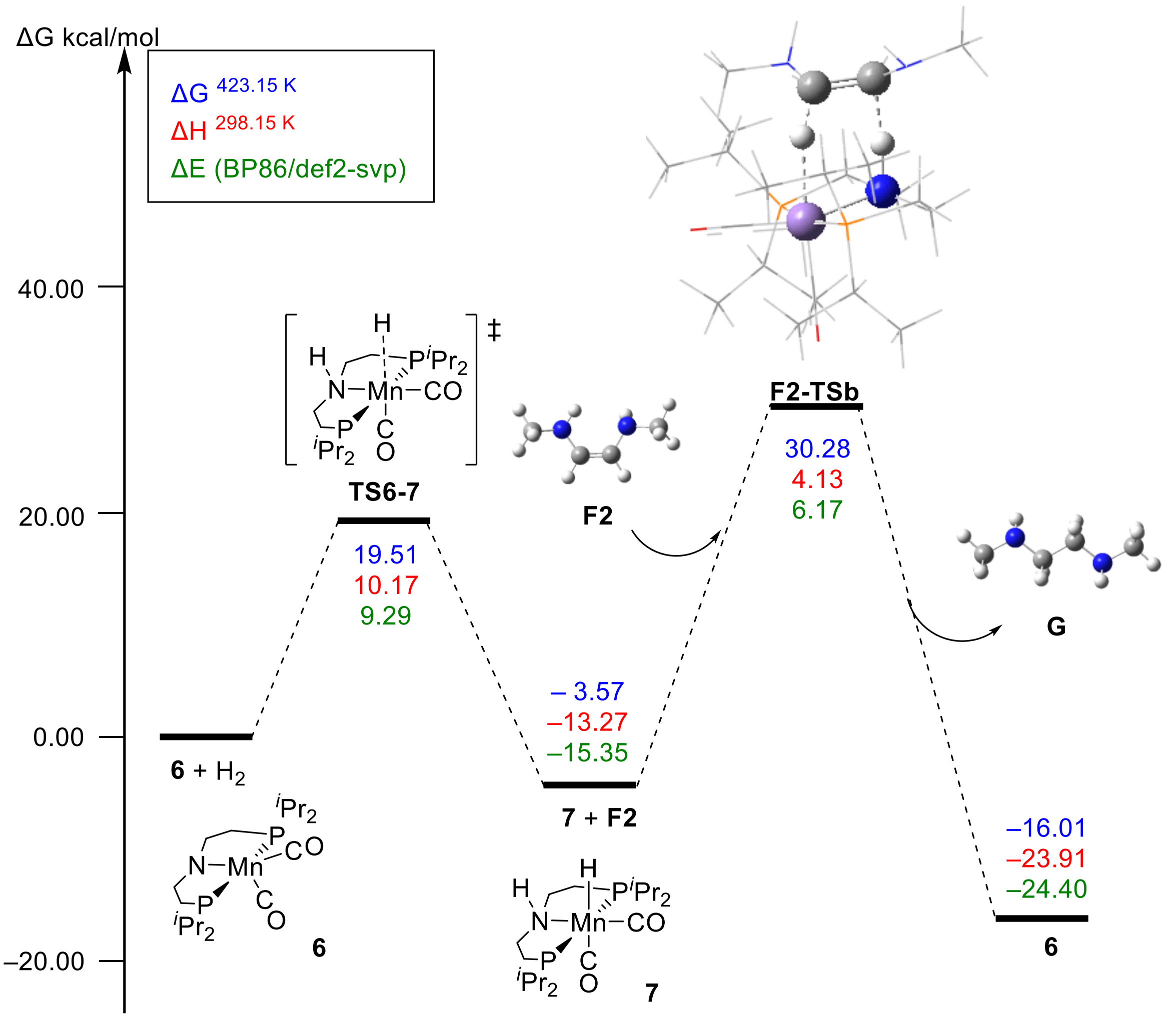

Scheme S13 Free energy profiles of the proposed pathways for dehydrogenation of **F2** to give **G** at the PBE0-D3[pcm,THF]/def2-TZVP//RI-BP86[pcm,THF]/def2-SVP level of theory.

2.8 Adduct formation of complex 6

The facile formation of the ethylene glycol adduct of **6**, compound **8** (see section 1.5), is corroborated by this reaction being computed to be exergonic, as ΔG of conformer **8** is –3.2 kcal/mol relative to **6** + ethylene glycol (see Figure 2B in the main paper). This conformer adopts a gauche conformation about the ethylene moiety, the corresponding anti minimum (**8a**, not shown), is higher than **8** by ΔG = 0.08 kcal/mol.

3. References

[1] M. Peña-López, P. Piehl, S. Elangovan, H. Neumann, M. Beller, *Angew. Chem. Int. Ed.,* **2016**, *55*, 14967–14971.

[2] J. Rana, V. Gupta, E. Balaraman, *Dalton Trans.* **2019**, *48*, 7094–7099.

[3] Y. Pocker, B. C. Davis, *J. Am. Chem. Soc.* **1973**, *95*, 6216–6223.

[4] M.J. Frisch, G.W. Trucks, H.B. Schlegel, G.E. Scuseria, M.A. Robb, J.R. Cheeseman, G. Scalmani, V. Barone, G.A. Petersson, H. Nakatsuji, X. Li, M. Caricato, A.V. Marenich, J. Bloino, B.G. Janesko, R. Gomperts, B. Mennucci, H.P. Hratchian, J.V. Ortiz, A.F. Izmaylov, J.L. Sonnenberg, D. Williams-Young, F. Ding, F. Lipparini, F. Egidi, J. Goings, B. Peng, A. Petrone, T. Henderson, D. Ranasinghe, V.G. Zakrzewski, J. Gao, N. Rega, G. Zheng, W. Liang, M. Hada, M. Ehara, K. Toyota, R. Fukuda, J. Hasegawa, M. Ishida, T. Nakajima, Y. Honda, O. Kitao, H. Nakai, T. Vreven, K. Throssell, J.A. Montgomery, Jr., J.E. Peralta, F. Ogliaro, M.J. Bearpark, J.J. Heyd, E.N. Brothers, K.N. Kudin, V.N. Staroverov, T.A. Keith, R. Kobayashi, J. Normand, K. Raghavachari, A.P. Rendell, J.C. Burant, S.S. Iyengar, J. Tomasi, M. Cossi, J.M. Millam, M. Klene, C. Adamo, R. Cammi, J.W. Ochterski, R.L. Martin, K. Morokuma, O. Farkas, J.B. Foresman, D.J. Fox, Gaussian 16, Revision C.01, Gaussian Inc., Wallingford CT, 2019

[5] A. D. Becke, *Phys. Rev. A* **1998**, *38*, 3098–3100.

[6] J. P. Perdew, *Phys. Rev. B* **1986**, *33*, 8822–8824.

[7] M. Ernzerhof, G. E. Scuseria, *J. Chem. Phys.* **1999**, *110*, 5029–5036.

[8] F. Weigend, F. Furche, R. Ahlrichs, *J. Chem. Phys.* **2003**, *119*, 12753–12762.

[9] F. Weigend, R. Ahlrichs, *Phys. Chem. Chem. Phys.* **2005**, *7*, 3297–3305.

[10] J. Tomasi, B. Mennucci, E. Cancès, *J. Mol. Struct. THEOCHEM* **1999**, *464*, 211–226.

[11] S. Grimme, J. Antony, S. Ehrlich, H. Krieg, *J. Chem. Phys.* **2010**, *132*, 1–19.

[12] S. Grimme, S. Ehrlich, L. Goerigk, *J. Comput. Chem.* **2011**, *32*, 1456–1465.

[13] A. S. Goodfellow, M. Bühl, *Molecules* **2021**, *26*, 4072.

[14] K. Fukui, *Acc. Chem. Res.* **1981**, *14*, 363–368.

[15] R. L. Martin, P. J. Hay, L. R. Pratt, *J. Phys. Chem. A* **1998**, *102*, 3565–3573.

[16] A. E. Owen, A. Preiss, A. McLuskie, C. Gao, G. Peters, M. Bühl, A. Kumar, *ACS Catal.* **2022**,

12, 6923–6933.

[17] M. Hatanaka, *Bull. Chem. Soc. Jpn.* **2009**, *82*, 1149–1151.

[18] J. Masdemont, J. A. Luque-Urrutia, M. Gimferrer, D. Milstein, A. Poater, *ACS Catal.* **2019**, *9*, 1662–1669.

[19] A. M. Tondreau, R. Michalczyk, J. M. Boncella, *Organometallics* **2017**, *36*, 4179–4183.

[20] Z. Wei, A. De Aguirre, K. Junge, M. Beller, H. Jiao, *Catal. Sci. Technol.* **2018**, *8*, 3649–3665.

[21] H. Li, M. B. Hall, *ACS Catal.* **2015**, *5*, 1895–1913.

[22] J. A. Luque-Urrutia, M. Solà, D. Milstein, A. Poater, *J. Am. Chem. Soc.* **2019**, *141*, 2398–2403.

[23] K. Das, S. Waiba, A. Jana, B. Maji, *Chem. Soc. Rev.* **2022**, *51*, 4386–4464.

4. DFT Coordinates

Optimised Cartesian Coordinates for all complexes of this study (RI-BP86[pcm,THF]/def2-SVP level)

H_2

H	0.00000000	0.00000000	0.38376237
H	0.00000000	0.00000000	-0.38376237

H_2O

O	0.00000000	0.00000000	0.12321100
H	0.00000000	0.75766997	-0.49284401
H	-0.00000000	-0.75766997	-0.49284401

CH_3NH_2

N	-0.75558640	-0.00000528	-0.13263986
H	-1.14504527	0.81367266	0.36541767
H	-1.14515801	-0.81364650	0.36539466
C	0.70309576	-0.00000336	0.01762954
H	1.13382141	-0.89057684	-0.48831415
H	1.13367166	0.89116997	-0.48735254
H	1.09324043	-0.00056217	1.06755616

Ketene

C	-0.10262970	-0.00003935	-0.00001940
O	-1.28082903	-0.00002957	0.00001763
C	1.22195574	-0.00005041	-0.00006509
H	1.76464474	0.95669048	0.00018286
H	1.76603127	-0.95591536	0.00018303

EDA

C	0.57246233	0.51974851	-0.00000391
C	-0.57245305	-0.51972864	0.00005370
H	0.45200918	1.18172278	-0.88913632
H	0.45192549	1.18194951	0.88898818
H	-0.45202883	-1.18179968	-0.88902203
H	-0.45181653	-1.18183631	0.88908945
N	-1.93784708	0.01722032	0.00013180
H	-2.05527450	0.63814642	0.81514024
N	1.93783340	-0.01723683	0.00008988
H	2.05591583	-0.63656907	-0.81603105
H	-2.05596443	0.63625449	-0.81619794
H	2.05527387	-0.63787180	0.81531902

A

C	0.70846685	0.55046509	-0.29146634
C	-0.70836308	0.55048969	0.29138239
H	0.64932585	0.56137319	-1.40373430
H	1.20743079	1.50893854	0.00586164
H	-0.64920980	0.56180758	1.40369891
H	-1.20732787	1.50889847	-0.00608497
O	1.47793885	-0.59356076	0.04847641
O	-1.47804774	-0.59343799	-0.04839889
H	1.53418917	-0.62514092	1.02351945
H	-1.53415961	-0.62561553	-1.02337715

B

C	0.82254978	0.50263137	0.00001390
O	1.34104022	-0.60601874	-0.00000383
H	1.43383689	1.44751853	0.00002634
C	-0.68146121	0.64714414	-0.00000518
H	-0.95534977	1.27288937	0.88943090

H	-0.95522705	1.27280891	-0.88952876
O	-1.31645055	-0.59996325	0.00000708
H	-0.56650881	-1.24401399	-0.00000681

C

C	-0.00865660	0.28910517	0.22447069
O	0.70125059	1.50348999	-0.02793158
C	0.91018534	-0.80289748	-0.34437559
H	0.48449241	-1.80587180	-0.12822197
H	0.93254965	-0.67963936	-1.45959971
O	2.20777488	-0.74630684	0.22696280
H	2.43183679	0.20853339	0.21447438
H	0.15334913	2.22688915	0.33690891
H	-0.12462495	0.11841084	1.32495174
N	-1.34112538	0.34793082	-0.34001319
C	-2.33321713	-0.55926161	0.23114061
H	-2.10748931	-1.65014306	0.12263163
H	-2.44622857	-0.35416442	1.31737400
H	-3.31912562	-0.37636511	-0.24331566
H	-1.29895532	0.25769294	-1.36477511

D1

C	0.07690744	-0.05903186	0.30574450
C	-1.29834713	0.53674143	0.10960549
H	-1.59480300	1.06818134	1.04226230
H	-1.24777914	1.29831871	-0.70467320
O	-2.29124217	-0.46125204	-0.10582216
H	-2.06807051	-0.90963094	-0.94493859
H	0.11668697	-0.94197110	0.99825840
N	1.11387592	0.40172827	-0.28532483
C	2.39460171	-0.22771867	-0.02241926
H	2.34448555	-1.10265963	0.67188941
H	2.84905683	-0.55753615	-0.98209515
H	3.09425710	0.52327082	0.40556349

D2

C	-0.00000763	-0.32836965	0.08236323
C	-1.28559179	0.49216564	-0.09300484
H	-1.31245998	1.30654649	0.66524787
H	-1.26605289	0.98830404	-1.09425871
O	-2.46321067	-0.27805241	0.11270762
H	-2.49565988	-0.94752726	-0.59788721
H	0.01787733	-1.13314438	-0.69049130
N	1.18791394	0.49842584	-0.09558438
C	2.43938964	-0.24688400	-0.01756504
H	2.50864536	-0.96116224	-0.86712891
H	3.30078331	0.44806786	-0.09822873
H	2.57063654	-0.84585488	0.92284309
H	-0.04821358	-0.85691312	1.07369653
H	1.19199031	1.23565002	0.62287697

D3

C	1.63916592	-0.68203547	0.26972317
H	0.94359546	-1.53642318	0.39512330
H	1.96346311	-0.35527855	1.29321983
H	2.53458809	-1.05687135	-0.26659609
N	0.99803068	0.36695473	-0.52018115
H	1.68843073	1.08343631	-0.77173520
C	-0.12704492	1.00449254	0.12768100
H	-0.39582436	1.94794013	-0.40231979
H	0.04893165	1.32874110	1.19697256

C	-1.39776897	0.17758174	0.17347661
H	-2.26412962	0.72371430	0.66190505
O	-1.52392275	-0.96302184	-0.23632329

D4

C	-1.36066218	0.56419060	0.06583848
C	-0.02331081	0.73700595	-0.09534177
H	-2.04885584	1.42032027	0.15975697
H	0.38600015	1.76071934	-0.10881856
O	-1.91106529	-0.70775883	0.01741956
H	-2.78711414	-0.68146431	0.44424097
N	0.89183199	-0.29281912	-0.34027119
H	0.47540869	-1.21708121	-0.18040984
C	2.24175012	-0.16652889	0.19092994
H	2.30564910	-0.18289468	1.30858716
H	2.87793080	-0.98698898	-0.19923669
H	2.69001684	0.79118808	-0.15013811

E

C	-0.57836512	-0.89464297	0.52064490
C	0.68983635	-0.50741828	-0.26842287
O	1.47757728	-1.69713678	-0.32068856
H	-0.24869096	-1.13973170	1.55427606
N	-1.57319442	0.17376695	0.57969442
C	-2.46267587	0.23282749	-0.57660106
H	-1.90098073	0.53017418	-1.48781088
H	-2.97557221	-0.73755094	-0.81128985
H	-3.24467672	1.00236067	-0.41227915
H	-0.98221925	-1.84694437	0.08275969
H	-2.13554281	0.05986640	1.43030066
H	0.39983012	-0.17683666	-1.29937924
H	2.37603537	-1.37389605	-0.53802921
N	1.47942004	0.57309397	0.29689307
H	1.48756877	0.50240957	1.32394263
C	1.15234310	1.93564787	-0.12057460
H	0.13020512	2.27292380	0.17114004
H	1.89169627	2.64243674	0.31179370
H	1.23131878	2.01537147	-1.22630659

F1

C	0.74677677	0.06480223	-0.30846657
C	-0.63188803	-0.52505889	-0.13968638
H	0.80538385	0.93906854	-1.00982784
H	-0.88394224	-1.06963801	-1.08213645
H	-0.59517239	-1.30065214	0.66950984
N	-1.63315166	0.52173457	0.04562435
H	-1.43956594	1.01637593	0.92682415
N	1.77331904	-0.39584011	0.30083313
C	-3.00464379	0.02369713	0.06278996
H	-3.20420016	-0.77663420	0.82332215
H	-3.70691866	0.85976575	0.25955231
H	-3.26374797	-0.40195363	-0.93131895
C	3.05956926	0.22457484	0.04318222
H	3.76362603	-0.53534062	-0.36157477
H	3.02153945	1.08681306	-0.66813090
H	3.50294109	0.57284219	1.00166273

F2

C	-0.68090286	0.84064063	-0.03749395
C	0.68247945	0.83333293	-0.04527543
N	-1.41677751	-0.35899265	-0.26694016

C	-2.76318221	-0.39155054	0.29056495
H	-2.71822488	-0.35480934	1.40020376
H	-3.41783748	0.45692275	-0.03992225
H	-3.26803369	-1.33771274	0.00661706
H	-1.23027108	1.79160580	0.11021042
H	-1.43538441	-0.60588019	-1.26705373
H	1.24413761	1.77360985	0.08999098
N	1.45053780	-0.29750626	-0.30304341
H	0.88253485	-1.15208457	-0.21706016
C	2.79272047	-0.39988814	0.25026887
H	2.82663283	-0.50317846	1.36362439
H	3.31581810	-1.27383376	-0.18860970
H	3.37761705	0.50564378	-0.01650239

G

C	-0.66449678	-0.37314193	-0.08176977
C	0.66449994	0.37315349	0.08177886
H	-0.66141965	-0.89716806	-1.07599077
H	-0.72425060	-1.18227930	0.68440157
H	0.72425184	1.18231173	-0.68436861
H	0.66144541	0.89713841	1.07601783
N	1.80460796	-0.51697495	-0.11397853
H	1.76159516	-1.26907982	0.58821307
N	-1.80460726	0.51696788	0.11393287
H	-1.76162457	1.26907163	-0.58825395
C	3.09380355	0.15640284	-0.00753747
H	3.25071260	0.72318289	0.94937641
H	3.91753869	-0.58146101	-0.10193002
H	3.20886149	0.88892104	-0.83684672
C	-3.09380262	-0.15640746	0.00756312
H	-3.91751900	0.58151601	0.10161649
H	-3.25061402	-0.72347178	-0.94920363
H	-3.20900689	-0.88867390	0.83707945

trans-glyoxal

C	0.32941177	0.69285703	0.00034674
O	-0.32941177	1.71497208	-0.00035292
H	1.45838032	0.66964472	0.00074288
C	-0.32941177	-0.69285703	0.00034674
O	0.32941177	-1.71497208	-0.00035292
H	-1.45838032	-0.66964472	0.00074288

a-MAE

C	0.52861179	0.03826007	-0.01504862
O	1.57981616	-0.84347615	-0.00086946
C	0.67461214	1.39648826	0.00847829
H	-0.19353723	2.06656649	0.03869365
H	1.67846265	1.84347401	-0.03712296
H	2.39876793	-0.32918087	0.13628585
N	-0.66291544	-0.65717079	-0.09287807
C	-1.93604376	0.02350468	0.02305684
H	-2.06544096	0.56579807	0.99183580
H	-2.05150781	0.76855940	-0.79328473
H	-2.75503138	-0.71455987	-0.07525316
H	-0.61291541	-1.60217054	0.29702872

cis-NMA

C	0.48578888	0.16723294	-0.00151228
O	0.42835879	1.40382704	-0.00007586
C	1.81772825	-0.57571256	0.00044734
H	1.71998302	-1.67851202	-0.03999993

H	2.41870048	-0.23425259	-0.86652152
N	-0.62745085	-0.63212295	-0.00147690
C	-1.98035376	-0.10635218	0.00094190
H	-2.54349843	-0.42848044	0.90314898
H	-1.90560001	0.99784900	-0.00010931
H	-2.54687078	-0.43009689	-0.89854407
H	-0.49828279	-1.64545825	-0.00044543
H	2.38187387	-0.29781377	0.91415476

gMAET

C	-0.43671735	0.02531287	0.31549502
O	-0.49068409	1.36655069	-0.22674638
C	-1.73139034	-0.73100444	0.00330786
H	-1.70380573	-1.75588513	0.42557450
H	-2.61096318	-0.20303323	0.42588936
N	0.70107156	-0.67628131	-0.21192264
C	2.00306262	-0.08744422	0.09822122
H	2.13222101	0.96459126	-0.25212891
H	2.15932530	-0.09164864	1.19900108
H	2.80752368	-0.70478705	-0.35171014
H	0.59069944	-0.78461667	-1.22997619
H	-1.87388790	-0.80220174	-1.09612519
H	-0.29858692	0.08858265	1.42488320
H	-1.19428342	1.84937687	0.24987714

NMEM

C	1.93891249	0.06632683	-0.09016017
C	0.65885228	-0.36237446	0.07703344
H	2.76592950	-0.65768487	-0.08333707
H	2.18789280	1.13446134	-0.20779622
H	0.45647236	-1.44533330	0.18258339
N	-0.47910289	0.41252317	0.18920971
H	-0.35618556	1.40869376	-0.01539227
C	-1.79811381	-0.12142531	-0.10340715
H	-1.89696888	-1.13439994	0.33937740
H	-2.57873836	0.51983125	0.35422704
H	-2.02258738	-0.20839285	-1.19492704

trans-MIA

C	0.02686678	0.28229568	-0.00014821
C	1.37415741	-0.35930495	0.00013337
H	1.33529651	-1.48846155	0.00036493
O	2.42206382	0.26941239	0.00003064
H	0.00230894	1.40033782	-0.00021325
N	-1.01387100	-0.47006783	-0.00035170
C	-2.32189980	0.14413146	0.00011921
H	-2.31459369	1.25974658	-0.00116115
H	-2.88962014	-0.22076945	-0.88383693
H	-2.88755150	-0.21841090	0.88643690

trans-MAMIE

C	-0.71519544	-0.03158624	-0.14651115
C	0.56032369	0.55567617	0.42926926
O	1.02103214	1.62305050	-0.42727048
H	-0.62251882	-0.57663288	-1.12420345
N	-1.84379950	0.11912347	0.43607109
C	-3.02342926	-0.44687283	-0.19063257
H	-2.82553579	-0.97361272	-1.15715446
H	-3.50730557	-1.16193982	0.51005923
H	-3.76889630	0.35827478	-0.37091726
H	1.06466223	1.27352721	-1.34031412

H	0.29326472	1.04710292	1.38643701
N	1.53002909	-0.47817461	0.72782315
H	2.23739222	-0.10011393	1.37000571
C	2.18673756	-1.13244644	-0.40480235
H	2.73643226	-0.43839941	-1.08823613
H	2.91907244	-1.87186996	-0.02319817
H	1.45094911	-1.69600616	-1.01551342

DMEDI

C	0.68157286	-0.27776657	0.00001982
C	-0.68157278	0.27776665	-0.00002127
H	0.75116377	-1.39477521	0.00013924
H	-0.75116369	1.39477529	-0.00013358
N	-1.70717361	-0.49825218	-0.00005846
N	1.70717365	0.49825226	0.00005603
C	-3.02267223	0.10471446	0.00003477
H	-3.02172519	1.22204729	0.00023683
H	-3.59110510	-0.25338294	-0.88663504
H	-3.59121676	-0.25374831	0.88648335
C	3.02267214	-0.10471458	-0.00003290
H	3.59110352	0.25338565	0.88663672
H	3.02172496	-1.22204741	-0.00023135
H	3.59121819	0.25374526	-0.88648170

DMEDE

C	0.22775141	-0.28311482	0.90328660
C	1.75900852	-0.27872164	0.85499135
H	-0.11597012	-1.31201217	0.66441135
H	-0.11570906	-0.05409996	1.94679843
H	2.15585724	0.72482065	1.17415527
H	2.12543298	-0.99544793	1.62525146
N	2.23260215	-0.69297188	-0.45923985
N	-0.38350837	0.61142168	-0.07867908
C	3.66753411	-0.53894199	-0.64854392
H	4.21924311	-1.22922956	0.02751216
H	3.94580224	-0.80523213	-1.68927096
H	4.05901269	0.49402385	-0.44331115
C	-0.01647499	2.02260501	0.02763923
H	-0.48315941	2.58667361	-0.80520384
H	-0.35273001	2.49146285	0.98774435
H	1.08110244	2.15276885	-0.05329923
C	-1.73968783	0.38905245	-0.38618626
C	-2.55449030	-0.58368007	0.10005008
O	-3.81554310	-0.76581307	-0.43856490
H	-4.31594588	-1.34734595	0.16216659
H	1.71576166	-0.15194117	-1.16695691
H	-2.15194440	1.07510039	-1.14803152
H	-2.24791071	-1.29537894	0.88456323

DMEDAA

C	0.38164623	0.59455855	1.01418372
C	1.59329334	-0.33490042	0.86224166
H	-0.22486137	0.27133247	1.89858831
H	0.75788405	1.61324390	1.24482617
H	2.14388692	-0.35643384	1.83076091
H	1.24457014	-1.39449300	0.69780912
N	2.46890923	0.14651629	-0.19643949
H	1.86911538	0.33951591	-1.01245065
N	-0.39798885	0.69381411	-0.22742636

C	3.53276833	-0.77937899	-0.55548832
H	3.18268792	-1.81270242	-0.82523895
H	4.10919904	-0.38287520	-1.41706974
H	4.24472571	-0.89547425	0.29166199
C	-1.07862922	1.96857736	-0.43641539
H	-1.50812029	1.99814266	-1.46043384
H	-1.91187013	2.18121380	0.28285165
H	-0.34897177	2.80045105	-0.35329314
C	-1.21553243	-0.46738028	-0.55300057
H	-0.61529853	-1.39597416	-0.40522342
H	-1.50082707	-0.46407397	-1.62939829
C	-2.49612901	-0.65114641	0.26217256
H	-2.49082406	-0.17122721	1.28901499
O	-3.46903025	-1.27436718	-0.12843827

DMTPZ

C	-0.67168279	1.26394863	-0.13587568
C	0.67178646	1.26406427	0.13617820
N	1.39876772	0.09148197	0.41236389
C	0.55978975	-1.09425169	0.54000571
C	-0.55961861	-1.09449388	-0.53940159
N	-1.39848312	0.09136082	-0.41206656
H	-1.23859950	2.20838952	-0.18308659
H	1.23856313	2.20858053	0.18350578
H	1.18727913	-2.00510431	0.46499374
H	-0.07468397	-1.10194043	-1.54405652
H	0.07473013	-1.10117316	1.54460899
H	-1.18713429	-2.00528040	-0.46385503
C	2.68551123	-0.07250857	-0.25106160
H	2.60192742	-0.34300390	-1.33555334
H	3.25565126	0.87806247	-0.19131797
H	3.28182237	-0.86000280	0.25611152
C	-2.68587396	-0.07241601	0.25010092
H	-2.60335679	-0.34318894	1.33460800
H	-3.25569770	0.87832752	0.19006314
H	-3.28196585	-0.85962208	-0.25777883

DMPRZ

C	-0.27142644	-0.71559418	1.20095257
C	0.27142644	0.71559418	1.20095257
N	-0.15517693	1.43207794	0.00000000
C	0.27142644	0.71559418	-1.20095257
C	-0.27142644	-0.71559418	-1.20095257
N	0.15517693	-1.43207794	-0.00000000
H	0.10444713	-1.25450422	2.09735658
H	-0.10444713	1.25450422	2.09735658
H	-0.10444713	1.25450422	-2.09735658
H	-1.39415350	-0.67847175	-1.28289292
H	1.39415350	0.67847175	-1.28289292
H	0.10444713	-1.25450422	-2.09735658
H	-1.39415350	-0.67847175	1.28289292
H	1.39415350	0.67847175	1.28289292
C	0.27142644	2.82232493	0.00000000
H	-0.12239156	3.34291580	0.89829589
H	1.38957622	2.94531875	0.00000000
H	-0.12239156	3.34291580	-0.89829589
C	-0.27142644	-2.82232493	0.00000000
H	0.12239156	-3.34291580	-0.89829589
H	0.12239156	-3.34291580	0.89829589

H	-1.38957622	-2.94531875	-0.00000000

AAEG

C	-1.08231048	0.09053652	0.26454220
H	-1.09772745	0.28983794	1.37051794
C	-2.27123055	-0.82069635	-0.08790087
H	-2.15655930	-1.12111351	-1.16113203
H	-2.24232305	-1.74643624	0.52652707
O	-3.49340928	-0.15631503	0.16314605
O	-1.32781386	1.34215283	-0.40563250
H	-3.32864138	0.74953467	-0.18448369
H	-1.16943464	2.06171499	0.23304317
N	0.15901953	-0.49412534	-0.18945945
H	0.15545220	-1.50623999	-0.00266238
C	1.37276378	0.11798192	0.34785085
H	1.31117570	1.21143899	0.14895039
H	1.48033514	0.00507570	1.45880803
C	2.63017198	-0.43852928	-0.33042241
H	2.50202079	-0.32941181	-1.43772082
H	2.68674039	-1.53377176	-0.13459600
N	3.83709568	0.18596968	0.22377099
H	4.66885656	-0.26815213	-0.17809157
H	3.89071538	1.16215354	-0.10386785

AIE

C	-1.15168837	-0.35841251	0.32114522
H	-1.09652768	-0.17557135	1.42731610
C	-2.54386680	-0.31602214	-0.26580188
H	-2.46693272	-0.36555114	-1.37794660
H	-3.10133346	-1.22004851	0.06876573
O	-3.28958151	0.80287271	0.20338892
H	-2.83766910	1.60470264	-0.12518576
N	-0.11274690	-0.57451103	-0.39395650
C	1.18686196	-0.60036366	0.25685099
H	1.11999310	-0.41112251	1.36031614
H	1.62837089	-1.61426953	0.12482386
C	2.14819477	0.40938012	-0.39308593
H	1.70470959	1.43393339	-0.29001624
H	2.18390432	0.19734576	-1.48384607
N	3.49785115	0.26291436	0.16162421
H	4.14174613	0.88282319	-0.34899126
H	3.50765192	0.61846223	1.12932828

AAE

N	0.01649758	0.39688399	0.01943580
H	0.00672829	0.97905772	0.86976473
C	-1.20880640	-0.39628751	-0.02213843
H	-1.21113085	-1.00564137	-0.95356034
H	-1.28519779	-1.13506382	0.82463959
C	-2.45527212	0.49618283	-0.01849460
H	-2.40896720	1.16816822	0.88045836
H	-2.40507492	1.16764431	-0.90324641
C	1.24144548	-0.39341284	-0.00038571
H	1.24772573	-1.02422369	-0.91744635
H	1.32726798	-1.11018697	0.86535573
C	2.48749177	0.50245422	-0.02163107
H	2.47996340	1.17448320	0.87469149
H	2.44383188	1.16267366	-0.91376676
N	-3.67524879	-0.31409665	-0.10748085
H	-3.81414094	-0.81510197	0.78287722

H	-4.49113259	0.30867572	-0.18503538
O	3.69598521	-0.24014011	-0.11580925
H	3.76435148	-0.78249580	0.69395636

AAAc

N	0.16246693	0.30793448	0.10365711
H	0.17335136	0.78654950	1.01503288
C	-1.09851574	-0.41692381	-0.04541043
H	-1.11388967	-0.90377479	-1.04602864
H	-1.22143737	-1.24734306	0.70261092
C	-2.29974817	0.52978705	0.05777215
H	-2.23813768	1.07855709	1.03572465
H	-2.20040673	1.30444410	-0.73337326
C	1.35496643	-0.51135408	-0.02531668
H	1.27717889	-1.10288873	-0.97070937
H	1.53729559	-1.26321424	0.78621802
C	2.60435118	0.33938865	-0.19407252
H	2.39527430	1.34810185	-0.66794590
N	-3.55360984	-0.20238807	-0.14992304
H	-3.73899834	-0.79759580	0.67120374
H	-4.33679094	0.46510808	-0.16945499
O	3.72977985	-0.00651947	0.11784356

AIEED

N	-1.49878682	-0.23762491	0.18525961
H	-1.52028856	-1.25581240	0.33924192
C	-0.33105382	0.09198576	-0.62843107
H	-0.34896503	1.19271418	-0.81651256
H	-0.29750779	-0.39646444	-1.63667605
C	-2.77229617	0.18452615	-0.39392372
H	-2.72517441	1.28662754	-0.55863007
H	-2.98635464	-0.26548154	-1.39979213
C	0.95015057	-0.22026440	0.10426458
H	0.97114501	0.10195089	1.17790103
N	1.93717924	-0.81150247	-0.45567719
C	3.15747997	-0.97481480	0.31616216
H	3.55713379	-1.99788819	0.13941140
H	2.98671473	-0.86651746	1.41920448
C	4.23206285	0.04214391	-0.11977631
H	5.21329704	-0.30473555	0.29395859
H	4.32243089	-0.00517819	-1.22708907
N	3.85687025	1.40681666	0.25715292
H	4.00315599	1.53570679	1.26887056
H	4.49176782	2.07810100	-0.19549546
C	-3.94533624	-0.13300142	0.54184557
H	-3.99029662	-1.23547204	0.69597548
H	-3.71675877	0.30960824	1.54527834
N	-5.21645044	0.29443013	-0.05452857
H	-5.99314730	-0.00973150	0.54859349
H	-5.26488072	1.32428563	-0.04053457

EDBED

C	0.62965853	0.43640400	-0.02663702
C	-0.62965886	-0.43650260	0.02665893
H	0.55620361	1.11647948	-0.91776574
H	0.64649709	1.10701700	0.86438316
H	-0.64648609	-1.10714874	-0.86433659
H	-0.55622853	-1.11654057	0.91781755
N	-1.84180459	0.37662752	0.00189653
H	-1.84113559	0.99708502	0.82474454

N	1.84182012	-0.37669555	-0.00192692
H	1.84127330	-0.99697454	-0.82490952
C	-3.07896053	-0.39850967	-0.00119585
H	-3.16569610	-1.09690117	0.87832899
H	-3.09306620	-1.05030137	-0.90370348
C	3.07894118	0.39849206	0.00147306
H	3.16554418	1.09737164	-0.87768009
H	3.09313424	1.04978291	0.90434460
C	-4.31149084	0.51231576	-0.03700800
H	-4.25467803	1.22153994	0.83217823
H	-4.25071536	1.14427503	-0.94980906
C	4.31151859	-0.51228915	0.03664862
H	4.25470907	-1.22095515	-0.83299781
H	4.25078028	-1.14485163	0.94903830
N	-5.54444129	-0.28146798	-0.09155678
H	-5.68760183	-0.74537369	0.81808177
H	-6.35064315	0.35099695	-0.18976784
N	5.54441991	0.28150843	0.09168830
H	6.35067844	-0.35097372	0.18927483
H	5.68742334	0.74620324	-0.81756821

I-4AED

C	-1.57918286	-0.48707217	-0.18813334
C	-2.81300679	0.33299557	0.20666976
H	-1.70461444	-1.53240028	0.20348701
H	-1.54401606	-0.58231338	-1.29870708
H	-2.84608392	0.42933475	1.31738672
H	-2.68931122	1.37788532	-0.18615007
N	-4.04537612	-0.31997580	-0.22537758
H	-4.03175182	-0.40924044	-1.25188786
N	-0.34689132	0.16322466	0.24782311
H	-0.36570666	0.25645525	1.27387321
C	-5.26601549	0.37644032	0.17012383
H	-5.31999648	1.43568709	-0.20911559
H	-5.27868152	0.45478515	1.28259664
C	0.86797217	-0.54674069	-0.13869988
H	0.90347496	-1.60477604	0.24666981
H	0.89815009	-0.63072792	-1.24805342
C	-6.52882993	-0.37647424	-0.28785847
H	-6.50528001	-0.48044567	-1.39761019
H	-6.48557297	-1.41281225	0.11463080
C	2.12108107	0.19861209	0.32996875
H	2.06757839	0.35779097	1.44201064
H	2.13234730	1.20938530	-0.12736006
N	-7.80672721	0.23260346	0.09071420
H	-7.84218865	0.33370458	1.11618045
H	-7.84123290	1.19873954	-0.26788513
N	3.33578719	-0.50633600	-0.08191360
H	3.36274488	-1.43528137	0.36095616
C	4.56351224	0.19627172	0.22277810
H	4.58087703	0.57348198	1.28889558
C	5.77358155	-0.72828518	0.03160644
H	5.75665063	-1.55516742	0.78322415
H	5.70401660	-1.17687430	-0.98607455
O	4.68796607	1.30596104	-0.65651905
O	6.93014351	0.09537776	0.17573121
H	5.64501915	1.52771838	-0.60430611
H	7.66648175	-0.32673839	-0.30391257

I-4AIE

C	1.00513464	0.07132059	0.41150923
C	2.29748669	-0.38020527	-0.27915039
H	1.13589631	-0.03702665	1.52136029
H	0.85525257	1.16110384	0.22862387
H	2.44768836	-1.46976384	-0.09426344
H	2.16512321	-0.27504685	-1.38955081
N	3.45875041	0.32985548	0.24861699
H	3.33901943	1.33860259	0.07521789
N	-0.15558639	-0.63856797	-0.11960426
H	-0.03077298	-1.64850766	0.04282031
C	4.73364909	-0.09800100	-0.31984604
H	4.78104482	0.00495982	-1.44058051
H	4.86031605	-1.18652000	-0.11255730
C	-1.42684529	-0.22073596	0.46238378
H	-1.49694551	-0.38022132	1.57157420
H	-1.54257050	0.87535304	0.30377107
C	5.92145500	0.66463407	0.29576162
H	5.78584116	1.75566318	0.11122554
H	5.88675753	0.53729177	1.40049549
C	-2.60231590	-0.94628777	-0.21322809
H	-2.45619356	-2.04467356	-0.08133766
H	-2.58459176	-0.74347320	-1.31361347
N	7.24809966	0.27655213	-0.19088456
H	7.38786654	-0.73123446	-0.02403078
H	7.27581791	0.37747773	-1.21665060
N	-3.86298400	-0.59100360	0.41764353
C	-4.80629989	-0.14571154	-0.32196933
H	-4.69575434	-0.00580516	-1.42958960
C	-6.16766630	0.19513803	0.22862506
H	-6.83511063	-0.68500426	0.03695390
H	-6.08782642	0.30546122	1.33716313
O	-6.64332865	1.36478196	-0.42443366
H	-7.58977475	1.45434892	-0.20647919

I-4AEE

C	1.10822298	0.51638965	-0.03863821
C	2.33637184	-0.39774979	0.04205057
H	1.21810512	1.33158805	0.72589387
H	1.09934770	1.02906760	-1.02891288
H	2.34388097	-0.91162598	1.03195414
H	2.22648635	-1.21250646	-0.72312861
N	3.57517061	0.36510868	-0.07908548
H	3.58242163	0.84429471	-0.99135638
N	-0.13048941	-0.24682121	0.08495359
H	-0.13724199	-0.72415758	0.99810808
C	4.78912727	-0.43728814	0.04007881
H	4.85195607	-1.26861411	-0.71734159
H	4.78021306	-0.93865283	1.03631685
C	-1.34064276	0.55913413	-0.03561539
H	-1.40294250	1.38429624	0.72813631
H	-1.33845913	1.06574369	-1.02652450
C	6.05963662	0.42607015	-0.06922186
H	6.05569340	0.95209032	-1.05218476
H	6.00932694	1.22578441	0.70268158
C	-2.59597369	-0.31417217	0.06579952
H	-2.57100515	-0.89157693	1.02854227
H	-2.57364086	-1.07289918	-0.74646836

N	7.33069799	-0.29074805	0.06434924
H	7.34795033	-0.78256119	0.97031991
H	7.37209335	-1.04169577	-0.64099168
N	-3.80987644	0.48476559	-0.08160594
H	-3.88287347	1.21580617	0.63682745
C	-5.02193418	-0.19340510	-0.28980398
H	-4.98280299	-1.00371626	-1.04081271
C	-6.21390634	0.12310698	0.27499724
H	-6.30164276	0.91333261	1.04536713
O	-7.39136440	-0.45233385	-0.16771761
H	-8.04988077	-0.37397618	0.54715457

L-oligomer

C	1.21407107	0.47434780	0.07450162
C	2.45998761	-0.41264076	-0.03342821
H	1.32156120	1.13366664	0.97742996
H	1.18034591	1.16395241	-0.80125579
H	2.49167344	-1.10451161	0.84081087
H	2.35387021	-1.06976709	-0.93808911
N	3.68286923	0.38488731	-0.02125965
H	3.66948797	1.02599375	-0.82794332
N	-0.00881313	-0.32329832	0.06700616
H	0.00364034	-0.95813838	0.87857456
C	4.91304330	-0.40026913	-0.06508178
H	4.98229156	-1.07367441	-0.96536705
H	4.92578966	-1.07929466	0.81959031
C	-1.23452825	0.46802989	0.10644123
H	-1.29410019	1.14711876	1.00303241
H	-1.25723567	1.13918347	-0.78119650
C	6.16570163	0.49438365	-0.02108053
H	6.14073203	1.19400806	-0.88872189
H	6.10870241	1.13511056	0.88673541
C	-2.47723330	-0.42799596	0.07421681
H	-2.43023396	-1.15286562	0.93475677
H	-2.44270418	-1.05123781	-0.84704144
N	7.45158832	-0.20811244	-0.03272619
H	7.48789183	-0.85985286	0.76536188
H	7.49963571	-0.81330779	-0.86615190
N	-3.70231410	0.36574125	0.06003813
H	-3.72465381	0.95644520	0.90407579
C	-4.92458725	-0.42867699	0.00538769
H	-5.06193200	-1.11564260	0.88399575
C	-6.15788520	0.46712033	-0.10702761
H	-6.05168208	1.10955385	-1.01539325
O	-7.30699016	-0.36890707	-0.16380216
H	-8.08641072	0.21085266	-0.24909609
H	-4.88360779	-1.08530944	-0.89264258
H	-6.19187034	1.15865588	0.77396807

b-4AED

N	1.75066065	-0.13986224	0.61795426
H	1.19544188	0.14747016	-0.20224505
N	-1.18187200	0.26079557	0.63055789
N	-0.34833668	2.65861979	-1.15173112
H	-0.13493289	1.68585246	-1.42250420
C	-2.25367423	-0.65598993	0.23752519
H	-3.14552855	-0.54187207	0.92567119
C	-0.61064294	2.67604084	0.29135862
H	0.35158953	2.49788537	0.82185093

H	-0.94683019	3.69577174	0.58296687
C	0.87413072	-0.97032466	1.44099698
H	0.60081950	-1.93865839	0.95247377
H	1.40382337	-1.22708475	2.38754139
C	-0.40727684	-0.20755578	1.80285509
H	-1.03622122	-0.83813391	2.47576284
H	-0.11174018	0.68967749	2.38623792
C	-2.72757413	-0.41270005	-1.20998295
H	-3.25224949	0.56375780	-1.31904789
C	-1.64824092	1.65184552	0.79188914
H	-2.58435041	1.79568882	0.21105067
H	-1.91766909	1.86748770	1.85795523
C	2.96770629	-0.80644911	0.16953295
H	2.77000635	-1.75592728	-0.40435220
H	3.55266795	-1.11721119	1.06733412
C	3.84298996	0.11962820	-0.69485863
H	4.06794525	1.03888048	-0.11005450
H	3.24922769	0.45934728	-1.57501540
N	5.09939105	-0.45930138	-1.17959709
H	5.65838326	-0.77458680	-0.37258022
H	4.89340027	-1.32191998	-1.70569473
H	-1.22216984	2.87874109	-1.65377915
H	-1.82440797	-0.40343860	-1.86287859
O	-1.77750171	-1.98653004	0.30804464
O	-3.59762514	-1.50617951	-1.51455117
H	-2.38451470	-2.46929256	-0.30079881
H	-3.64508431	-1.59949074	-2.48402794
**b-4AEE**			
N	1.60643559	-0.37672553	0.55723160
H	1.03241250	0.17528834	-0.09660591
N	-1.38224818	-0.25168917	0.81973156
N	-1.02461738	2.46399003	-0.77093117
H	-0.82498138	1.58000738	-1.26230264
C	-2.11538161	-0.95249601	-0.14252052
H	-1.96562224	-2.04741451	-0.12860892
C	-0.95825835	2.24565515	0.67733260
H	0.10575254	2.07716311	0.95969210
H	-1.26914487	3.18399919	1.19006542
C	0.80054287	-1.50404489	1.01101495
H	0.55135913	-2.22701787	0.18645985
H	1.38412369	-2.08864822	1.75814071
C	-0.49816247	-1.03657082	1.68480052
H	-1.03175359	-1.93527104	2.08189263
H	-0.23185002	-0.40219764	2.55450664
C	-2.99885631	-0.46144281	-1.05404850
C	-1.79356499	1.08264268	1.25752302
H	-2.86763584	1.22282436	0.99180716
H	-1.73259317	1.12792861	2.37010033
C	2.85468432	-0.75233621	-0.10112741
H	2.70086638	-1.41910187	-0.99531458
H	3.46512129	-1.34829186	0.61715846
C	3.66639215	0.48145653	-0.53741906
H	3.85095791	1.11573655	0.35770991
H	3.04326541	1.09971998	-1.22433051
N	4.94408298	0.20168012	-1.19783177
H	5.53027236	-0.37102392	-0.57225783
H	4.77731148	-0.39698450	-2.02053073

H	-1.98817241	2.70998173	-1.04109289
H	-3.19103750	0.61714140	-1.18789188
O	-3.76193127	-1.33389078	-1.81523478
H	-3.99314624	-0.88068267	-2.64745425

B-oligomer

N	1.41360076	0.23095704	0.58263445
H	0.83537994	-0.07824526	-0.21500904
N	-1.43093661	0.28293586	0.40448259
N	-0.31106192	2.46066781	-1.38680840
H	0.38289238	1.93382225	-0.83014212
C	-2.09969500	-1.01643214	0.47989621
H	-3.16240361	-0.92467222	0.82934712
C	-1.52422170	2.61917004	-0.58416855
H	-1.25752726	3.20542844	0.32330128
H	-2.25345585	3.25691802	-1.13347545
C	0.61794557	-0.01434013	1.78254335
H	0.48843905	-1.11084586	1.99518507
H	1.14114191	0.40498043	2.67121677
C	-0.75393901	0.66443323	1.64981362
H	-1.37450236	0.44852472	2.55786663
H	-0.59026928	1.76035592	1.63482106
C	-2.08432143	-1.78543646	-0.84530682
C	-2.27723128	1.32968109	-0.17713422
H	-2.74702040	0.90254755	-1.09001653
H	-3.12957273	1.60968957	0.50369711
C	2.71125518	-0.43552654	0.56406968
H	2.63247557	-1.55011099	0.70186996
H	3.31211630	-0.06774396	1.42875992
C	3.48889084	-0.15418015	-0.73475540
H	3.60891834	0.94627703	-0.84598000
H	2.87190939	-0.48297536	-1.60295065
N	4.80568086	-0.78896338	-0.83605156
H	5.38837999	-0.48763486	-0.04069600
H	4.70219520	-1.80744565	-0.71320183
H	-0.51530956	1.85821282	-2.19843274
H	-2.56577912	-1.17740973	-1.65036304
O	-2.77378774	-3.01235860	-0.63501191
H	-2.78755425	-3.49110316	-1.48444906
H	-1.58199050	-1.64726559	1.23310179
H	-1.02124192	-1.94583024	-1.15390185

TS6-7

P	2.26475419	0.03407430	-0.31874845
C	2.45006319	-1.12611336	-1.77888361
C	1.20499015	-2.01856911	-1.82090055
H	3.39273689	-1.70998771	-1.75293871
H	2.47693685	-0.48442488	-2.68611960
H	1.19767972	-2.58473923	-2.78953327
H	1.28403923	-2.80443732	-1.01706249
N	-0.00000017	-1.21998466	-1.69410994
H	0.00000032	0.42895573	-1.68530279
C	-1.20499055	-2.01856897	-1.82090066
C	-2.45006369	-1.12611333	-1.77888368
H	-1.28403973	-2.80443718	-1.01706259
H	-1.19768006	-2.58473913	-2.78953336
H	-2.47693797	-0.48442510	-2.68611983
H	-3.39273715	-1.70998806	-1.75293820
P	-2.26475455	0.03407455	-0.31874863

Mn	-0.00000007	0.08512608	-0.00991915
C	-0.00000067	-1.17659311	1.22132021
O	-0.00000050	-2.02389921	2.04831181
C	0.00000079	1.37640919	1.19981510
O	0.00000238	2.22448144	2.02671830
C	3.09260604	1.60856835	-0.98359918
C	4.51808209	1.39108559	-1.52000917
C	3.03745858	2.79987066	-0.01254423
H	2.42548279	1.84121898	-1.84541558
H	4.57867363	0.56937503	-2.26175825
H	4.87628089	2.31619799	-2.02116193
H	5.23663073	1.16951162	-0.70256278
H	2.02312620	2.96345689	0.40067270
H	3.73673318	2.66507313	0.83927328
H	3.34539747	3.72870349	-0.53901407
C	3.44104997	-0.59521531	1.02396761
C	3.44863532	-2.12919112	1.14900701
C	3.13580962	0.06387798	2.38182479
H	4.45187997	-0.27072081	0.68895189
H	3.74941279	-2.63508412	0.20989017
H	4.17722932	-2.43111195	1.93217982
H	2.45623164	-2.51925661	1.45229919
H	3.10519683	1.16964443	2.32705770
H	2.15899511	-0.27895301	2.77908038
H	3.91721297	-0.21607323	3.12078196
C	-3.09260585	1.60856843	-0.98360028
C	-3.03745141	2.79987395	-0.01254971
C	-4.51808463	1.39108732	-1.52000363
H	-2.42548569	1.84121463	-1.84542029
H	-2.02311702	2.96345874	0.40066273
H	-3.34538991	3.72870580	-0.53902151
H	-3.73672295	2.66508124	0.83927107
H	-4.57868221	0.56937138	-2.26174628
H	-5.23663110	1.16952248	-0.70255287
H	-4.87628159	2.31619736	-2.02116203
C	-3.44105128	-0.59521550	1.02396631
C	-3.13581432	0.06387989	2.38182322
C	-3.44863443	-2.12919119	1.14900748
H	-4.45188122	-0.27072317	0.68894835
H	-3.10520478	1.16964638	2.32705519
H	-3.91721751	-0.21607303	3.12077991
H	-2.15899911	-0.27894777	2.77908010
H	-3.74940887	-2.63508577	0.20989052
H	-2.45623091	-2.51925480	1.45230252
H	-4.17722990	-2.43111220	1.93217884
H	0.00000085	1.24838779	-1.30810821

D1-TSa

C	0.05149973	-0.22881278	1.05993967
O	0.44555546	1.82364868	-0.11856401
C	0.99506055	-1.14350148	0.31761569
H	1.90579813	-1.31053149	0.94134149
H	0.54367480	-2.14683836	0.12724281
O	1.26432153	-0.48552753	-0.90722649
H	1.04676336	0.53586723	-0.71964484
H	0.86200869	2.64899921	-0.44122805
H	0.42252743	0.37725093	1.90181324
N	-1.10572781	0.15462912	0.56017655

C	-1.72165308	-0.48751401	-0.60162459
H	-1.00792128	-0.50532262	-1.45082813
H	-2.00365039	-1.52928305	-0.35029667
H	-2.63571606	0.07019297	-0.87468638
H	-1.02184919	1.23126180	0.49579001

D4-TSb

C	0.00822201	-0.34872289	-0.25295500
O	-0.08355629	1.95525410	-0.05735710
C	-1.27127220	-0.26904182	0.44153199
H	-1.24871402	-0.72778208	1.45988392
H	-1.05358747	0.96559518	0.46466081
O	-2.38386938	-0.67449819	-0.34088494
H	-3.04342817	0.04155595	-0.28169733
H	0.47550489	2.14254719	0.72735113
H	0.02921429	-0.17094240	-1.33947454
N	1.15023238	-0.72213249	0.32217131
C	2.44390495	-0.34749281	-0.23424207
H	2.50241697	-0.65367594	-1.29770101
H	2.56493120	0.75922187	-0.18519732
H	3.25626998	-0.84493132	0.32621169
H	1.12004250	-0.91116307	1.33069023

D1-TSa-H_2O

C	-0.73393831	0.92253632	-0.06519780
O	2.27986419	-0.81622383	-0.97596854
C	0.48718761	0.95577859	0.70040426
H	0.34036177	1.23231449	1.77010416
H	0.62882460	-0.33947834	0.84062212
O	1.57141900	1.59147128	0.07650992
H	1.99502657	0.83493585	-0.43138552
H	1.80588207	-0.90046411	-1.82640387
H	-0.89083053	1.58154737	-0.94535398
N	-1.62181237	-0.03924585	0.15896168
C	-2.88492034	-0.21485452	-0.53505995
H	-3.74079788	-0.10633040	0.16410302
H	-2.98650650	0.54559463	-1.33393229
H	-2.93566705	-1.22726659	-0.98595184
H	-1.21459137	-0.83833985	0.71150449
H	1.65693426	-1.29875278	-0.32450793
O	0.47569931	-1.67936209	0.77676477
H	0.86821684	-1.95688451	1.62914162

D4-TSb-H_2O

C	-0.73393831	0.92253632	-0.06519780
O	2.27986419	-0.81622383	-0.97596854
C	0.48718761	0.95577859	0.70040426
H	0.34036177	1.23231449	1.77010416
H	0.62882460	-0.33947834	0.84062212
O	1.57141900	1.59147128	0.07650992
H	1.99502657	0.83493585	-0.43138552
H	1.80588207	-0.90046411	-1.82640387
H	-0.89083053	1.58154737	-0.94535398
N	-1.62181237	-0.03924585	0.15896168
C	-2.88492034	-0.21485452	-0.53505995
H	-3.74079788	-0.10633040	0.16410302
H	-2.98650650	0.54559463	-1.33393229
H	-2.93566705	-1.22726659	-0.98595184
H	-1.21459137	-0.83833985	0.71150449
H	1.65693426	-1.29875278	-0.32450793

O	0.47569931	-1.67936209	0.77676477
H	0.86821684	-1.95688451	1.62914162

6

P	2.25429754	-0.12281291	-0.23003811
C	2.46811988	-1.21039064	-1.72966656
C	1.19433804	-2.04652770	-1.88873614
H	3.38115531	-1.84081975	-1.68633946
H	2.57700422	-0.51913858	-2.59254538
H	1.04527181	-2.29117771	-2.96966086
H	1.31353883	-3.03840555	-1.38500815
N	-0.00047472	-1.35874643	-1.36426548
C	-1.19539417	-2.04285104	-1.89319827
C	-2.46860227	-1.20600264	-1.73275811
H	-1.31657774	-3.03667199	-1.39375177
H	-1.04476213	-2.28334926	-2.97484918
H	-2.57706074	-0.51291425	-2.59419594
H	-3.38216693	-1.83576474	-1.69073956
P	-2.25433308	-0.12198248	-0.23065598
Mn	-0.00012509	0.12948105	-0.18070629
C	-0.00022144	0.73426719	1.48151094
O	-0.00015924	1.12650608	2.60295820
C	-0.00015515	1.73917244	-0.85542249
O	-0.00071065	2.78916070	-1.41028361
C	3.37806678	1.34615700	-0.62800972
C	4.86114627	0.97387893	-0.79404420
C	3.18488388	2.55036531	0.31027429
H	2.97590040	1.63974534	-1.62496418
H	5.01267639	0.13185196	-1.50033457
H	5.42848670	1.84370114	-1.19004264
H	5.32699918	0.69640590	0.17483373
H	2.11603838	2.81296162	0.43141375
H	3.60906046	2.36730987	1.31820718
H	3.70621985	3.43767044	-0.10880575
C	3.12868233	-1.09855130	1.14053881
C	2.40239963	-2.43328546	1.38512053
C	3.28288343	-0.30699626	2.45055549
H	4.14212324	-1.31851514	0.73462326
H	2.40470242	-3.08810951	0.49087159
H	2.90157182	-2.99256712	2.20544639
H	1.34578905	-2.26483741	1.68145296
H	3.92092387	0.59082635	2.33151623
H	2.30254460	0.02178786	2.85084425
H	3.76243442	-0.95003978	3.21995579
C	-3.37789400	1.34822900	-0.62447925
C	-3.18357408	2.55010926	0.31657827
C	-4.86120859	0.97690924	-0.79040327
H	-2.97627549	1.64409669	-1.62097186
H	-2.11459259	2.81257233	0.43677949
H	-3.70562804	3.43837572	-0.09958621
H	-3.60626978	2.36450222	1.32466457
H	-5.01354019	0.13647003	-1.49842184
H	-5.32656933	0.69749803	0.17815119
H	-5.42846585	1.84781139	-1.18414875
C	-3.12834768	-1.10108350	1.13783699
C	-3.28143214	-0.31285129	2.44995314
C	-2.40234309	-2.43671412	1.37836395
H	-4.14210993	-1.31954067	0.73189743

H	-3.91881122	0.58578261	2.33350812
H	-3.76113698	-0.95749764	3.21789464
H	-2.30067498	0.01422833	2.85063172
H	-2.40702404	-3.08976498	0.48284797
H	-1.34493427	-2.26940552	1.67252954
H	-2.89998400	-2.99728930	2.19874028

7

P	2.22613805	0.06530368	-0.31169259
C	2.44740759	-1.08912883	-1.78824639
C	1.24232528	-2.02763293	-1.85061175
H	3.40069209	-1.65477659	-1.76188017
H	2.47427335	-0.45385329	-2.70090571
H	1.23628075	-2.60293447	-2.80681799
H	1.26469236	-2.76231828	-1.01876523
N	0.00006244	-1.23022951	-1.71103380
C	-1.24268676	-2.02666226	-1.85174152
C	-2.44716509	-1.08738076	-1.78937427
H	-1.26594672	-2.76189859	-1.02041011
H	-1.23657592	-2.60132583	-2.80833084
H	-2.47292696	-0.45125618	-2.70146804
H	-3.40086962	-1.65237024	-1.76411678
P	-2.22607851	0.06570836	-0.31179722
Mn	0.00005308	0.03563956	0.05051323
C	-0.00000573	-1.28843226	1.23715815
O	-0.00014885	-2.11681243	2.09292445
C	0.00024790	1.29035601	1.27010053
O	0.00066392	2.16477277	2.07480257
C	3.04125738	1.64529777	-0.98178531
C	4.46864588	1.46198331	-1.52395361
C	2.96245741	2.83087978	-0.00572725
H	2.35897915	1.86608255	-1.83431483
H	4.54794221	0.64200281	-2.26655701
H	4.80470441	2.39480116	-2.02679095
H	5.19643799	1.25676478	-0.71022291
H	1.94780776	2.95579829	0.42016497
H	3.67686813	2.71438935	0.83646134
H	3.23318642	3.77243396	-0.53057653
C	3.45970230	-0.57447572	0.98372570
C	3.45917072	-2.10837988	1.10547364
C	3.18740310	0.07365008	2.35371013
H	4.46374293	-0.25299562	0.62606237
H	3.74100107	-2.61695139	0.16128966
H	4.19845841	-2.42053921	1.87482198
H	2.46825553	-2.48839933	1.42723741
H	3.17847028	1.18054234	2.31228118
H	2.20622311	-0.25359294	2.75423514
H	3.97083120	-0.23052591	3.08133201
C	-3.04116190	1.64629445	-0.98044541
C	-2.96277817	2.83085351	-0.00311606
C	-4.46842279	1.46331784	-1.52306727
H	-2.35873100	1.86805357	-1.83259578
H	-1.94819822	2.95568841	0.42295781
H	-3.23378380	3.77286832	-0.52699769
H	-3.67718644	2.71325773	0.83891996
H	-4.54745713	0.64418557	-2.26663307
H	-5.19629589	1.25703498	-0.70967589
H	-4.80456814	2.39665728	-2.02488207

C	-3.45983893	-0.57528138	0.98287846
C	-3.18771287	0.07142451	2.35356943
C	-3.45950428	-2.10933121	1.10302745
H	-4.46382797	-0.25337564	0.62544568
H	-3.17863311	1.17835949	2.31326450
H	-3.97133763	-0.23338803	3.08071334
H	-2.20668095	-0.25637630	2.75398781
H	-3.74148932	-2.61687919	0.15834133
H	-2.46865370	-2.48984835	1.42439006
H	-4.19879060	-2.42218307	1.87209745
H	-0.00039649	1.24049231	-1.03186017
H	0.00067850	-0.52598252	-2.46471339
**9**			
P	2.27008358	0.05475253	-0.23739363
C	2.44765689	-0.75695840	-1.92663426
C	1.24093699	-1.66350330	-2.17643982
H	3.40219725	-1.30818206	-2.04652104
H	2.44940536	0.06727285	-2.67160274
H	1.22034002	-1.99811258	-3.24101193
H	1.28756890	-2.57784648	-1.54774710
N	0.00001428	-0.93381923	-1.83538723
C	-1.24100673	-1.66340465	-2.17649231
C	-2.44756781	-0.75664186	-1.92672526
H	-1.28773137	-2.57772243	-1.54777521
H	-1.22036473	-1.99802657	-3.24105708
H	-2.44870845	0.06787946	-2.67139340
H	-3.40223309	-1.30751128	-2.04715875
P	-2.27003963	0.05476601	-0.23739738
Mn	-0.00002299	0.03166769	0.07809722
C	-0.00008043	-1.44713380	1.02197728
O	-0.00014892	-2.44152233	1.67815904
C	0.00003217	1.00814771	1.53553350
O	0.00019486	1.66532001	2.52407410
C	3.07056807	1.73796020	-0.55816227
C	4.45915807	1.67730715	-1.21322146
C	3.07175354	2.66104137	0.67184239
H	2.32302897	2.13495517	-1.28207522
H	4.47500473	1.07081627	-2.14128704
H	4.79245513	2.70233374	-1.48640695
H	5.22565592	1.26381224	-0.52262214
H	2.08210938	2.70305105	1.16776486
H	3.82172557	2.34440449	1.42743418
H	3.34126740	3.69481942	0.36465362
C	3.47574806	-0.87308878	0.88970596
C	3.44806451	-2.39744819	0.67771388
C	3.21374877	-0.52721791	2.36789894
H	4.48628927	-0.49933914	0.60707941
H	3.72252756	-2.69405981	-0.35471632
H	4.18038656	-2.88071598	1.36017776
H	2.45005971	-2.82093635	0.91228391
H	3.21690568	0.56325549	2.56165641
H	2.23209801	-0.92385379	2.69824855
H	3.99715243	-0.98574462	3.00930338
C	-3.07059997	1.73802678	-0.55797555
C	-3.07199155	2.66087347	0.67216482
C	-4.45910932	1.67745038	-1.21324849
H	-2.32308753	2.13517144	-1.28186692

H	-2.08246601	2.70279364	1.16833642
H	-3.34141617	3.69473257	0.36516368
H	-3.82214669	2.34406700	1.42750777
H	-4.47480575	1.07118805	-2.14146343
H	-5.22566848	1.26375279	-0.52283779
H	-4.79239874	2.70253511	-1.48621798
C	-3.47571691	-0.87306125	0.88969664
C	-3.21355213	-0.52747723	2.36792381
C	-3.44831408	-2.39738025	0.67740912
H	-4.48620684	-0.49907336	0.60720513
H	-3.21636022	0.56297170	2.56182182
H	-3.99708534	-0.98585444	3.00927539
H	-2.23201512	-0.92445659	2.69818670
H	-3.72289529	-2.69372489	-0.35506822
H	-2.45033861	-2.82106458	0.91178866
H	-4.18062458	-2.88068304	1.35985616
H	0.00002500	-0.00828644	-2.31540195
O	0.00014911	1.56499601	-1.27949465
H	0.00023649	2.42234803	-0.81607683

B-TS1

P	2.35422497	0.09951803	-0.09016363
C	2.48117286	0.59276239	-1.90106712
C	1.33972613	-0.08008061	-2.66519404
H	3.46613139	0.35678706	-2.35303768
H	2.35811478	1.69704835	-1.94533653
H	1.28087713	0.31527913	-3.70684776
H	1.50191467	-1.17576704	-2.73849425
N	0.05709465	0.14826553	-1.95252756
C	-1.10217941	-0.45033089	-2.66235369
C	-2.38922389	-0.10830311	-1.91150957
H	-0.94698094	-1.54864055	-2.71153878
H	-1.14990106	-0.07526793	-3.71218365
H	-2.57515531	0.98556300	-1.97617820
H	-3.26694143	-0.62104099	-2.35463963
P	-2.13776054	-0.49645509	-0.08913355
Mn	0.12774606	-0.33661342	0.13534874
C	0.35916115	-2.07564942	-0.05947291
O	0.51330722	-3.25360009	-0.12844309
C	0.14380036	-0.42257705	1.88742366
O	0.15516486	-0.45830629	3.07319095
C	3.17347578	1.58611035	0.76681881
C	4.54725953	1.98306089	0.20051721
C	3.21036867	1.45917619	2.29941997
H	2.45571257	2.40005176	0.51944411
H	4.53235044	2.14019456	-0.89688934
H	4.88399037	2.93480749	0.66563723
H	5.32477982	1.22310076	0.42704536
H	2.23402337	1.14909200	2.72104627
H	3.97624906	0.72670611	2.63010525
H	3.48312858	2.43681098	2.75190580
C	3.65297579	-1.27068339	0.13918545
C	3.72703002	-2.25562958	-1.04089576
C	3.44231770	-2.02280459	1.46730205
H	4.62419695	-0.72969263	0.19094577
H	3.98111918	-1.76011905	-1.99956946
H	4.52289322	-3.00631150	-0.84390217
H	2.77727610	-2.81212074	-1.17047056

H	3.36917300	-1.34423099	2.33939935
H	2.51820184	-2.63339444	1.43751238
H	4.29596756	-2.71139119	1.64808326
C	-3.29792328	0.76314720	0.71881427
C	-3.24326101	0.74778092	2.25591456
C	-4.75007294	0.71720694	0.21182650
H	-2.85532702	1.72377378	0.37489462
H	-2.20462449	0.75022729	2.64128150
H	-3.75834883	1.64681104	2.65801753
H	-3.76268949	-0.13783390	2.67981901
H	-4.82195707	0.75710793	-0.89435566
H	-5.28901051	-0.19057746	0.55762299
H	-5.30864398	1.59301069	0.60746122
C	-3.03149141	-2.15052270	0.19125681
C	-2.63901799	-2.76921077	1.54624070
C	-2.84169637	-3.16356993	-0.95142124
H	-4.11013919	-1.87973121	0.22803937
H	-2.76051848	-2.06292416	2.39103933
H	-3.27649143	-3.65558867	1.75521956
H	-1.58365676	-3.10791600	1.53945071
H	-3.20463155	-2.78272580	-1.92741115
H	-1.78080945	-3.46422384	-1.06185820
H	-3.42302317	-4.08444051	-0.72758549
H	-0.13757561	1.30805049	0.36061476
C	-0.16312388	3.09484460	-0.41783365
H	-0.10311587	1.18178804	-1.94298793
O	-0.50830486	2.93400257	-1.60924894
H	0.90902748	3.07123419	-0.10920816
C	-1.12120711	3.82059669	0.51647792
H	-1.15555475	3.34200912	1.51882437
H	-0.67292151	4.83995128	0.66702699
O	-2.40352531	3.89101164	-0.06102239
H	-2.22906952	3.65925887	-1.00902175

B-TS2

P	2.38450243	0.06496235	-0.09516714
C	2.47763257	0.71662156	-1.84559339
C	1.27461786	0.16963068	-2.61547068
H	3.43942469	0.48818993	-2.34813532
H	2.38352295	1.82087101	-1.76774121
H	1.16501978	0.72374013	-3.57967508
H	1.42802508	-0.90033443	-2.88862078
N	0.04118235	0.31753228	-1.80663249
C	-1.13554897	-0.07472740	-2.61655542
C	-2.42994932	0.17591448	-1.84230686
H	-1.05352958	-1.14776917	-2.90899435
H	-1.15007884	0.50577244	-3.57134543
H	-2.61016437	1.26777472	-1.75382238
H	-3.31063010	-0.27263285	-2.34555971
P	-2.19241686	-0.43522139	-0.09025336
Mn	0.10734242	-0.28965777	0.12884112
C	0.29418658	-2.00195226	-0.01686644
O	0.42331103	-3.17718778	-0.13019511
C	0.13932859	-0.40335448	1.89707167
O	0.17517990	-0.50583889	3.07573224
C	3.25898123	1.43840262	0.88429577
C	4.62129138	1.85229217	0.30124845
C	3.35287418	1.16618960	2.39540931

H	2.55375133	2.28680586	0.73994736
H	4.56456604	2.12839766	-0.77065808
H	5.00594000	2.73879303	0.85010458
H	5.38175681	1.05040524	0.41153389
H	2.39019386	0.83138309	2.82880450
H	4.12119480	0.39926658	2.62650721
H	3.65730436	2.09558337	2.92262656
C	3.60973097	-1.38020428	-0.02072371
C	3.56818324	-2.28076625	-1.26840664
C	3.45109710	-2.21038738	1.26766770
H	4.60416623	-0.88050457	0.00379257
H	3.78457695	-1.72704711	-2.20371547
H	4.34161662	-3.07313012	-1.17280869
H	2.58933989	-2.78887486	-1.37718017
H	3.46161396	-1.59040484	2.18473913
H	2.50729020	-2.78994842	1.25958086
H	4.28864523	-2.93658928	1.34588822
C	-3.33622223	0.73467843	0.87051911
C	-3.20114841	0.64271423	2.39906896
C	-4.80955225	0.66252387	0.43097522
H	-2.94427353	1.72283516	0.53931265
H	-2.14768467	0.69088301	2.73618258
H	-3.74764818	1.48694894	2.87182049
H	-3.64429598	-0.29263905	2.80127861
H	-4.93480850	0.73908541	-0.66847037
H	-5.30476548	-0.27153964	0.77114394
H	-5.37086009	1.50827761	0.88346385
C	-3.07192598	-2.11174462	-0.01801377
C	-2.78110960	-2.85202653	1.30056978
C	-2.79823242	-3.00611484	-1.24044840
H	-4.15073167	-1.84111727	-0.03584460
H	-2.98815719	-2.22984904	2.19353790
H	-3.42081803	-3.75810962	1.36986433
H	-1.72526286	-3.18391277	1.35386137
H	-3.09422744	-2.52537623	-2.19451793
H	-1.73271879	-3.30077246	-1.30946268
H	-3.39379648	-3.94019937	-1.14940948
H	-0.29260828	1.75846223	0.42402249
C	-0.08827163	2.82308051	-0.01218186
H	-0.04246439	1.65271263	-1.64102579
O	-0.07454693	2.79421484	-1.38918871
H	0.88384321	3.12592527	0.45118330
C	-1.20842272	3.77114290	0.47898741
H	-1.53161209	3.52632710	1.51423429
H	-0.80267573	4.81458284	0.49397414
O	-2.31100874	3.66898884	-0.40726289
H	-1.83982346	3.42743754	-1.25187162
**TS-D1**			
P	2.27108370	-0.36745261	-0.00256088
C	2.53160909	0.14684174	-1.79017375
C	1.28634199	-0.22043086	-2.59785761
H	3.44240734	-0.29591450	-2.24220116
H	2.67535156	1.24679554	-1.78904915
H	1.32195632	0.25494399	-3.60657927
H	1.23627579	-1.31796903	-2.76013279
N	0.04450723	0.18135340	-1.88197574
C	-1.16712646	-0.17405765	-2.66894512

C	-2.41775510	0.28248244	-1.92054644
H	-1.16819383	-1.27340338	-2.82307948
H	-1.12152054	0.30022148	-3.67771365
H	-2.43300658	1.39370308	-1.88198329
H	-3.34334965	-0.05162578	-2.43228229
P	-2.29122319	-0.30440386	-0.14312608
Mn	-0.00767630	-0.40161793	0.15597068
C	-0.02468960	-2.14686771	-0.05366445
O	-0.01751417	-3.33346648	-0.14846669
C	-0.06813546	-0.47228395	1.91156198
O	-0.11396662	-0.50133925	3.09701563
C	3.31174886	0.93017088	0.91708759
C	4.74998967	1.09283223	0.39648032
C	3.28346389	0.76130968	2.44572474
H	2.75153252	1.85944209	0.67347985
H	4.79613353	1.27715227	-0.69599022
H	5.23218319	1.96056012	0.89668339
H	5.37804378	0.20467780	0.62072133
H	2.25691902	0.61501831	2.83552328
H	3.90245874	-0.09971385	2.77442631
H	3.70763100	1.66624723	2.93181294
C	3.30822703	-1.94694545	0.21236937
C	3.25104199	-2.89982701	-0.99501750
C	2.95076690	-2.68602757	1.51650556
H	4.35332718	-1.57417821	0.29793948
H	3.60682173	-2.42692772	-1.93254332
H	3.91060400	-3.77405814	-0.80404728
H	2.22806376	-3.29122024	-1.16221582
H	2.96533364	-2.02370804	2.40393029
H	1.94481968	-3.14530699	1.45314108
H	3.68295085	-3.50284874	1.69612499
C	-3.41315644	0.94842007	0.73889269
C	-3.49470537	0.73511245	2.26019243
C	-4.81086758	1.12004104	0.11937080
H	-2.84895897	1.88670104	0.56304257
H	-2.50006460	0.56671852	2.71809331
H	-3.94497709	1.62949881	2.74213250
H	-4.14193912	-0.12927275	2.51815019
H	-4.77730823	1.33901727	-0.96697613
H	-5.44927247	0.22378124	0.27001868
H	-5.33242823	1.97049029	0.60963662
C	-3.34592819	-1.88804788	-0.07605927
C	-3.11682627	-2.67239230	1.23018318
C	-3.18822553	-2.80087805	-1.30488660
H	-4.39204550	-1.50907813	-0.07479577
H	-3.21681814	-2.04010259	2.13382903
H	-3.86469501	-3.49092583	1.31068059
H	-2.11096315	-3.13540337	1.24953578
H	-3.44877437	-2.29020904	-2.25388809
H	-2.16012477	-3.20500292	-1.38997992
H	-3.87510426	-3.66953409	-1.20653993
C	0.26018617	2.87299893	-0.11142936
H	0.02606540	1.23650530	-1.79531677
C	-0.75321470	3.36911553	0.91976639
O	-0.50424192	4.74199346	1.21841692
H	1.30793075	3.00013390	0.27851975
H	-1.76631739	3.33387862	0.47220098

H	-0.73744244	2.73188623	1.83442149
H	0.32097791	4.77801187	1.74062551
N	0.04558231	3.06933789	-1.40605578
C	1.18440692	3.44759654	-2.22568477
H	1.07946544	4.51260046	-2.54362101
H	2.17382318	3.37170713	-1.70381837
H	1.24256889	2.86004125	-3.17015545
H	0.13590304	1.33764530	0.31154936

TS-D3

P	-2.37808898	-0.02555232	0.08450987
C	-2.43398443	0.64884940	1.82888376
C	-1.27928010	0.02497928	2.61482296
H	-3.41279568	0.50111097	2.32867002
H	-2.25213413	1.74161972	1.74121829
H	-1.14992457	0.56588481	3.58355458
H	-1.49443622	-1.03657505	2.87373236
N	-0.03022047	0.11083237	1.82068914
C	1.12645191	-0.35754438	2.61952907
C	2.41867084	-0.12932549	1.83444789
H	0.99904316	-1.43241893	2.88207634
H	1.17027141	0.19991504	3.58652539
H	2.58557563	0.96524631	1.73772787
H	3.30266081	-0.57182779	2.33688293
P	2.15557103	-0.75847088	0.09289108
Mn	-0.12659024	-0.49884771	-0.13209634
C	-0.39912528	-2.19654983	0.08225139
O	-0.58510609	-3.35842590	0.24620518
C	-0.15371244	-0.64014327	-1.89648824
O	-0.17757519	-0.75178150	-3.07524860
C	-3.17706492	1.37479447	-0.91711490
C	-4.49642640	1.89827714	-0.32393352
C	-3.31937799	1.06102753	-2.41629694
H	-2.41068282	2.17482949	-0.81194182
H	-4.39590826	2.21692269	0.73283993
H	-4.83837166	2.78303053	-0.90314098
H	-5.30848624	1.14243901	-0.38091461
H	-2.38922987	0.64975476	-2.85540800
H	-4.14146705	0.34027133	-2.60856959
H	-3.57119905	1.99175750	-2.96839606
C	-3.67943010	-1.40447436	0.01969833
C	-3.71563734	-2.27554683	1.28820445
C	-3.53659353	-2.27515873	-1.24368094
H	-2.41068282	2.17482949	-0.81194182
H	-4.39590826	2.21692269	0.73283993
H	-4.83837166	2.78303053	-0.90314098
H	-5.30848624	1.14243901	-0.38091461
H	-2.38922987	0.64975476	-2.85540800
H	-4.14146705	0.34027133	-2.60856959
H	-3.57119905	1.99175750	-2.96839606
C	-3.67943010	-1.40447436	0.01969833
C	-3.71563734	-2.27554683	1.28820445
C	-3.53659353	-2.27515873	-1.24368094
H	-4.64467981	-0.85300223	-0.04141347
H	-3.91989643	-1.68850886	2.20582246
H	-4.52998313	-3.02604137	1.19375241
H	-2.76905442	-2.83465150	1.42940126
H	-3.48707315	-1.67947773	-2.17541360

H	-2.62815699	-2.90702544	-1.19542621
H	-4.41239203	-2.95423541	-1.32710965
C	3.35604477	0.32157796	-0.90215624
C	3.36586746	0.01540313	-2.40953576
C	4.78311940	0.36979794	-0.32895998
H	2.89793651	1.32509080	-0.76419084
H	2.34611494	-0.05922868	-2.83532550
H	3.90091998	0.82486639	-2.95090104
H	3.90321633	-0.92998751	-2.63269835
H	4.80777453	0.66589295	0.73894784
H	5.30622453	-0.60489177	-0.42886806
H	5.38436015	1.11627532	-0.89160250
C	2.96013921	-2.47533046	0.03460694
C	2.56336405	-3.25573174	-1.23334868
C	2.71276285	-3.31442335	1.30102627
H	4.04973147	-2.25345289	-0.01707820
H	2.71296504	-2.67297036	-2.16273301
H	3.18210597	-4.17553097	-1.31234007
H	1.50185958	-3.56996293	-1.19616617
H	3.08446618	-2.82076266	2.22116429
H	1.63801237	-3.54939288	1.43534370
H	3.25229733	-4.28190794	1.21016092
H	0.26833833	1.46834380	-0.39123245
C	0.19754860	2.57034630	0.07129152
H	0.15126672	1.34733301	1.68343350
O	0.31862419	2.54621053	1.41913569
H	-0.80560903	2.92605750	-0.29854453
C	1.24961234	3.40847797	-0.68534950
H	1.19642262	3.15353872	-1.76626388
H	2.26984172	3.11295472	-0.31704295
N	0.99380191	4.83942615	-0.54178581
H	1.43200440	5.34095967	-1.32347234
C	1.43769896	5.41700922	0.72407098
H	1.21139663	6.50358215	0.74380638
H	0.89787708	4.92925038	1.56018878
H	2.53482164	5.28876009	0.92746430

D1-TS

P	-2.30514164	-0.37498762	0.00801659
C	-2.53323544	0.25694119	1.75812217
C	-1.29122303	-0.10664585	2.57686738
H	-3.46033572	-0.11785607	2.23665747
H	-2.62251202	1.36014536	1.68513185
H	-1.31633867	0.42197294	3.56024640
H	-1.27567100	-1.19722097	2.80152058
N	-0.06472751	0.24893343	1.83259775
C	1.15720577	-0.00132414	2.62352895
C	2.38422762	0.39556292	1.79950885
H	1.19679949	-1.07483549	2.91485943
H	1.13061515	0.58889737	3.57121681
H	2.31956630	1.48631919	1.59139778
H	3.33372294	0.19796730	2.33775326
P	2.27478085	-0.45226949	0.13549883
Mn	-0.01636650	-0.55995659	-0.15312604
C	-0.06715507	-2.21046862	0.39420618
O	-0.10257051	-3.34503825	0.74645317
C	0.03192364	-1.11565687	-1.82625689
O	0.07217492	-1.50477020	-2.94388019

C	-3.17790244	0.95914715	-1.01364125
C	-4.60744307	1.28312983	-0.54896523
C	-3.12423958	0.68469085	-2.52592944
H	-2.52916973	1.83799862	-0.80414850
H	-4.65955013	1.54587238	0.52688088
H	-4.99885262	2.15314064	-1.11944566
H	-5.30589131	0.43857901	-0.73037227
H	-2.10234228	0.43017278	-2.87097283
H	-3.79935526	-0.14699847	-2.81807083
H	-3.45838455	1.58454455	-3.08568625
C	-3.42833828	-1.89473004	-0.16120370
C	-3.50591044	-2.75683431	1.11157670
C	-3.02766657	-2.74977311	-1.37829193
H	-4.43985120	-1.46889159	-0.34762929
H	-3.88700487	-2.19644912	1.98845078
H	-4.20468424	-3.60365583	0.93876773
H	-2.52016501	-3.19134200	1.37267998
H	-2.93673675	-2.15786079	-2.30983934
H	-2.05678335	-3.25683064	-1.20732318
H	-3.79236709	-3.53715360	-1.55213385
C	3.27852253	0.69347038	-0.98486821
C	3.43199394	0.16121551	-2.42015035
C	4.63071316	1.12995118	-0.39757072
H	2.60268237	1.57334381	-1.02180252
H	2.46966490	-0.18076956	-2.85074860
H	3.82941806	0.96533740	-3.07613880
H	4.15179140	-0.68244940	-2.47314583
H	4.53131426	1.60836587	0.59723154
H	5.33725919	0.27784433	-0.30063701
H	5.11025459	1.87130266	-1.07293512
C	3.34369244	-2.01275425	0.26074174
C	3.03761483	-3.00062019	-0.88184134
C	3.25962097	-2.71531078	1.62738275
H	4.38455100	-1.63730272	0.13363798
H	3.06811640	-2.52707327	-1.88201826
H	3.78598437	-3.82221650	-0.87629714
H	2.03654682	-3.45882859	-0.75577109
H	3.56236381	-2.05864741	2.46736333
H	2.23822884	-3.09751600	1.82777337
H	3.94521636	-3.59006008	1.63365426
C	-0.10274170	2.98701344	-0.16807886
H	-0.08227705	1.33371231	1.60473325
C	1.05129614	3.78535610	-0.81830255
O	0.92600121	5.16885239	-0.50921397
H	-1.06969140	3.37232529	-0.62370096
H	2.01277892	3.44942934	-0.37733317
H	1.09868808	3.61955473	-1.92654205
H	0.06816805	5.46502629	-0.87360592
N	-0.12508519	2.89940557	1.19337226
C	-1.21033397	3.58417015	1.85129631
H	-0.98172035	4.65296666	2.11072823
H	-2.16128703	3.63251750	1.24258533
H	-1.47572723	3.09724881	2.82159063
O	0.08286920	1.51532374	-0.79520407
H	0.10470354	1.59464670	-1.76895552

D4-TS

P	2.36164948	-0.01287079	-0.09982562

C	2.38855032	0.72053538	-1.82481408
C	1.22096474	0.10594639	-2.60954609
H	3.36314374	0.59703730	-2.34123166
H	2.21931649	1.81134739	-1.70112318
H	1.09581371	0.66747261	-3.57602494
H	1.48493566	-0.94426018	-2.91534465
N	0.00089037	0.15854330	-1.81806003
C	-1.13410380	-0.35144203	-2.57553707
C	-2.42401600	-0.17841347	-1.76322590
H	-1.00226644	-1.43245175	-2.85914212
H	-1.24114406	0.19715173	-3.55020378
H	-2.64230909	0.90626396	-1.66874679
H	-3.30105556	-0.65896115	-2.24462573
P	-2.09266514	-0.81762378	-0.03426601
Mn	0.17819841	-0.60689133	0.15516286
C	0.46512317	-2.24009845	-0.39355910
O	0.66354524	-3.36637647	-0.73627255
C	0.29608981	-1.12023236	1.83585386
O	0.38265836	-1.48685027	2.96535177
C	3.00375621	1.42307000	0.95851056
C	4.25750787	2.12504368	0.41578319
C	3.16487913	1.05592668	2.44331347
H	2.12105616	2.09598312	0.87649584
H	4.13934286	2.46845856	-0.63179226
H	4.49362998	3.02001715	1.03281000
H	5.14964377	1.46323276	0.45687445
H	2.27585112	0.52771198	2.84240883
H	4.05194994	0.40962282	2.61378898
H	3.31658537	1.97606427	3.04906410
C	3.77658737	-1.27874607	-0.05992891
C	3.91229965	-2.08421769	-1.36483778
C	3.63281513	-2.22889061	1.14429202
H	4.70323223	-0.67393653	0.07137348
H	4.10344143	-1.44311186	-2.24863314
H	4.76814169	-2.78904867	-1.27973645
H	3.00336913	-2.68809486	-1.56193195
H	3.53046437	-1.69214344	2.10728476
H	2.74190497	-2.87841045	1.02796553
H	4.52611151	-2.88697353	1.21728640
C	-3.15720365	0.32382973	1.03450175
C	-3.13496100	-0.05075752	2.52575241
C	-4.59285203	0.53129450	0.52944552
H	-2.56232622	1.25759766	0.91597783
H	-2.10430805	-0.21984204	2.89662488
H	-3.58480285	0.76737297	3.13042911
H	-3.72926566	-0.96623567	2.73343332
H	-4.63077146	0.87590050	-0.52366160
H	-5.19804771	-0.39860790	0.60434711
H	-5.10923036	1.29919044	1.14738664
C	-2.96614912	-2.50110709	0.06589727
C	-2.45789050	-3.31736301	1.26960031
C	-2.85172378	-3.32901814	-1.22679572
H	-4.04174802	-2.25841677	0.22805548
H	-2.50087630	-2.75397113	2.22180973
H	-3.07025798	-4.23748415	1.39199420
H	-1.40514610	-3.62964353	1.11546933
H	-3.28737515	-2.81532918	-2.10705046

H	-1.79450942	-3.57485596	-1.45457216
H	-3.39776211	-4.28999491	-1.10383691
C	-0.26263663	3.46996489	-0.32676600
H	-0.28992516	1.79829328	-1.66227378
H	0.76149685	3.54229098	0.06534038
O	-0.22565236	1.38680551	0.68459489
H	-0.04634380	1.49030130	1.63853631
C	-0.50318933	2.98363562	-1.68742888
H	0.24673022	3.39908608	-2.39857651
O	-1.85251892	3.17616204	-2.11625188
H	-1.92460689	4.06180958	-2.52379099
N	-1.23592648	4.04817098	0.35423704
H	-2.15900893	3.97711377	-0.09971525
C	-1.16940446	4.49068344	1.73684136
H	-1.70022152	5.45624221	1.85338097
H	-1.63594216	3.74616243	2.41595728
H	-0.11230014	4.62696081	2.03349246

F1-TSa

P	-2.39164368	0.33384746	0.02188007
C	-2.33076247	1.07462481	1.74128918
C	-1.29749816	0.30454677	2.56880200
H	-3.32070642	1.09559442	2.24009501
H	-2.00115694	2.12698538	1.61916705
H	-1.10986957	0.83762638	3.53219379
H	-1.68569060	-0.70508175	2.83582254
N	-0.03905113	0.15538548	1.81000189
C	1.00913983	-0.51397417	2.60515730
C	2.29180766	-0.60698339	1.77504503
H	0.65571483	-1.52331519	2.91619482
H	1.20700992	0.05964255	3.54303265
H	2.63100386	0.42869276	1.55245260
H	3.10416906	-1.13713135	2.31280493
P	1.86503416	-1.36849087	0.12123997
Mn	-0.31883323	-0.65693001	-0.14868052
C	-0.94300757	-2.17024839	0.43754570
O	-1.37419848	-3.21009950	0.81854293
C	-0.50017907	-1.22824773	-1.80818140
O	-0.62315121	-1.63144157	-2.91481819
C	-2.76312873	1.84595056	-1.05591047
C	-3.95412226	2.69156860	-0.57491392
C	-2.88377736	1.50392327	-2.55089994
H	-1.83114843	2.43710523	-0.91585597
H	-3.85459180	3.01083982	0.48206636
H	-4.03124571	3.61368980	-1.19073790
H	-4.91879384	2.15106030	-0.68151979
H	-2.04913938	0.86860612	-2.90888836
H	-3.83421104	0.97627440	-2.77698756
H	-2.88178706	2.43812113	-3.15249856
C	-3.98584930	-0.69089268	-0.05811140
C	-4.32228759	-1.42261716	1.25373819
C	-3.95798666	-1.67508616	-1.24278836
H	-4.78510741	0.06176469	-0.24339444
H	-4.44202275	-0.73288409	2.11293914
H	-5.28595490	-1.96341520	1.13471674
H	-3.55166362	-2.17634011	1.51204163
H	-3.70445872	-1.18635041	-2.20355701
H	-3.21893415	-2.48345352	-1.07219534

H	-4.95572304	-2.15036477	-1.35954443
C	3.19988070	-0.66411678	-1.01665212
C	3.12108175	-1.20356332	-2.45510817
C	4.62776657	-0.76239580	-0.45526637
H	2.89730519	0.40529447	-1.03788916
H	2.09248290	-1.16685719	-2.86626558
H	3.77312292	-0.59625472	-3.11921002
H	3.48019743	-2.25204529	-2.52284077
H	4.72733107	-0.29559391	0.54500110
H	4.97478830	-1.81536319	-0.38013257
H	5.33408196	-0.23814084	-1.13511243
C	2.30389408	-3.20813017	0.25154869
C	1.65028709	-4.02674787	-0.87852092
C	1.99180864	-3.82589145	1.62621674
H	3.40835424	-3.23144583	0.11044751
H	1.84178330	-3.60371598	-1.88350401
H	2.04967399	-5.06384648	-0.86892790
H	0.55226152	-4.08946010	-0.74262646
H	2.51905642	-3.31454468	2.45627074
H	0.90410948	-3.81783943	1.84076558
H	2.32111459	-4.88741101	1.63527721
C	0.82643063	2.67309115	-0.27572144
H	0.35992636	1.17223274	1.53679621
C	2.08402593	3.09729598	-1.08065900
H	-0.01576246	3.34464826	-0.62300395
H	2.91018829	2.36894573	-0.86767086
H	1.86833437	3.04283813	-2.17223147
N	0.91765895	2.58472976	1.10035624
C	0.18633701	3.59636577	1.82766926
H	0.76747532	4.53785336	2.02896158
H	-0.75227497	3.94843822	1.30688670
H	-0.12370670	3.22443763	2.83492799
O	0.49769357	1.24512018	-0.84471940
H	0.51241928	1.29232901	-1.82008975
N	2.45462173	4.47668322	-0.76278357
H	2.87151061	4.91674154	-1.59068454
C	3.36538755	4.60427094	0.37209805
H	4.36010427	4.10522555	0.21988665
H	3.55166403	5.67606117	0.59546384
H	2.89789249	4.13002901	1.25804410
**F2-TSa**			
P	2.34153206	0.61752581	-0.07309677
C	2.03001204	1.59163002	-1.64420533
C	1.03041692	0.79473799	-2.49479830
H	2.95876706	1.84185003	-2.19745806
H	1.56755504	2.55029487	-1.32852154
H	0.67560808	1.44451790	-3.34181634
H	1.56077021	-0.06540063	-2.99093578
N	-0.08793077	0.35588014	-1.67318910
C	-1.08632883	-0.33191658	-2.47858463
C	-2.29231854	-0.71735626	-1.61040040
H	-0.67388215	-1.25433193	-2.97566171
H	-1.43391348	0.31746626	-3.32945909
H	-2.81093243	0.20222521	-1.26379641
H	-3.03559579	-1.33336569	-2.15816209
P	-1.63154569	-1.55026244	-0.06712580
Mn	0.46830881	-0.66455426	0.10553896

C	1.20148088	-1.99658374	-0.75512722
O	1.71020941	-2.92233890	-1.31166536
C	0.87265386	-1.40200028	1.65296719
O	1.15631619	-1.91504865	2.68936247
C	2.58186189	1.98404574	1.21796835
C	3.50895694	3.13210305	0.79137100
C	2.96668590	1.44044666	2.60439492
H	1.52967522	2.34150849	1.28026417
H	3.20687487	3.59538461	-0.16952700
H	3.50469503	3.93495846	1.56137329
H	4.56255302	2.79295530	0.68779865
H	2.31816347	0.59837495	2.91893460
H	4.02015926	1.08931401	2.63101981
H	2.87644076	2.24450037	3.36727113
C	4.07462944	-0.13454188	-0.26989221
C	4.37571503	-0.60932960	-1.70295911
C	4.29541536	-1.28376276	0.73233692
H	4.77872873	0.69338635	-0.02367992
H	4.32867355	0.21059990	-2.44723430
H	5.40271540	-1.03342176	-1.74690404
H	3.67321218	-1.40738870	-2.01827380
H	4.06097480	-0.99760085	1.77589762
H	3.65965554	-2.15527282	0.47626835
H	5.35600762	-1.61628169	0.70140938
C	-2.89721219	-1.04475064	1.24510713
C	-2.64501711	-1.70320077	2.61184600
C	-4.36634919	-1.18696087	0.81988632
H	-2.61628942	0.02970346	1.33160912
H	-1.58438453	-1.62494630	2.92359856
H	-3.26601390	-1.20953164	3.39149115
H	-2.92519154	-2.77836350	2.61315576
H	-4.59568117	-0.65062571	-0.12306430
H	-4.65801159	-2.25138918	0.68381535
H	-5.03420751	-0.77071080	1.60647885
C	-1.93800457	-3.40791874	-0.31778847
C	-1.09673917	-4.24220085	0.66729178
C	-1.69569085	-3.88144770	-1.76214292
H	-3.01673563	-3.55714331	-0.08065008
H	-1.22418754	-3.92332316	1.71989949
H	-1.38458384	-5.31422246	0.60059445
H	-0.01758406	-4.16551585	0.42464391
H	-2.34784012	-3.36994224	-2.49819000
H	-0.64004422	-3.72635207	-2.06457331
H	-1.90662667	-4.97069507	-1.83790266
C	-1.28737388	3.08322793	0.47124940
H	-0.79912969	1.84762608	-1.18997119
H	-0.37615882	3.38178604	1.00677584
N	-2.43511756	3.10614756	1.11876715
H	-3.23357300	2.87047682	0.49817447
C	-2.61848264	3.18481007	2.55621433
H	-3.42839866	3.90099116	2.80052053
H	-2.88826059	2.19262632	2.97546575
H	-1.68176758	3.52770601	3.03508122
C	-1.29218552	2.91695801	-0.99073146
H	-0.58944103	3.65027063	-1.45984701
O	-0.49952273	0.96319897	1.01462933
H	-0.30103658	0.94184896	1.97044946

N	-2.64312451	2.94361211	-1.54447016
H	-2.72148843	2.25415720	-2.30018921
C	-3.10041912	4.25342009	-2.01419607
H	-2.43024907	4.71597683	-2.78178617
H	-4.11577537	4.16476010	-2.45259321
H	-3.16481405	4.96304192	-1.16213952

F1-TSb

P	-2.32698118	-0.30021957	-0.02237022
C	-2.63858443	-0.24591319	1.83142654
C	-1.43869032	-0.87226377	2.54438805
H	-3.58805849	-0.73391260	2.13056493
H	-2.72181215	0.82502694	2.10932940
H	-1.48650393	-0.65731196	3.63879324
H	-1.44359300	-1.97760679	2.43036931
N	-0.17245110	-0.34687764	1.97183852
C	1.00991901	-0.87699107	2.70014248
C	2.29971060	-0.34863012	2.06778935
H	0.97660150	-1.98664114	2.66812299
H	0.96049946	-0.58055767	3.77514324
H	2.36412896	0.74846383	2.23306670
H	3.19822930	-0.80537932	2.53131434
P	2.19972999	-0.61835632	0.21184046
Mn	-0.05964641	-0.50782416	-0.13751101
C	-0.18973305	-2.25553902	-0.23697502
O	-0.27890369	-3.43914733	-0.33221527
C	0.05511611	-0.28005158	-1.87980689
O	0.13434686	-0.12834533	-3.05404998
C	-3.20211550	1.28370166	-0.60082466
C	-4.64508396	1.44952248	-0.09553901
C	-3.10840244	1.49960922	-2.12030372
H	-2.57114540	2.05641845	-0.10845532
H	-4.72918981	1.35929340	1.00648326
H	-5.02775791	2.45542239	-0.37377997
H	-5.33305038	0.70705829	-0.55279807
H	-2.07757169	1.35966075	-2.50158736
H	-3.77576637	0.80511168	-2.67275310
H	-3.43268434	2.53109640	-2.37739360
C	-3.46814422	-1.66520157	-0.69732212
C	-3.60676038	-2.88241316	0.23431002
C	-3.04033267	-2.10103292	-2.11189755
H	-4.46635689	-1.17821041	-0.76860676
H	-4.01435946	-2.61716601	1.23044736
H	-4.31089226	-3.61363101	-0.21893552
H	-2.63864769	-3.40264441	0.37620789
H	-2.90130371	-1.24615718	-2.80223172
H	-2.08818160	-2.66703217	-2.08370880
H	-3.81574857	-2.76595971	-2.55032602
C	3.38126569	0.70368151	-0.47320102
C	3.61371179	0.57897350	-1.98910245
C	4.70987152	0.83057250	0.29084693
H	2.79280803	1.63392479	-0.31963828
H	2.66513474	0.47275975	-2.55153615
H	4.12467590	1.49270336	-2.36161905
H	4.26954011	-0.28121925	-2.23841690
H	4.56778734	1.03023339	1.37185367
H	5.33697179	-0.08134238	0.19083063
H	5.30195502	1.67472658	-0.12477190

C	3.17534716	-2.22142416	-0.10429088
C	2.89982485	-2.79652611	-1.50780981
C	2.96341936	-3.30059728	0.97234952
H	4.24102123	-1.90117699	-0.05845702
H	3.00815264	-2.04534224	-2.31320802
H	3.61542453	-3.62057512	-1.71921218
H	1.87717361	-3.21630839	-1.57259290
H	3.25452801	-2.96093967	1.98659852
H	1.91026871	-3.64589897	1.00253287
H	3.59177928	-4.18613505	0.73408246
H	0.04161430	1.26876194	0.05816548
C	0.39356412	2.48427975	0.82751735
H	-0.16093275	0.72644326	2.14689030
H	1.47442508	2.25156722	0.73539634
H	-0.04610230	4.54865275	0.38273385
C	-1.28536527	3.14482125	2.40173755
H	-1.62783245	2.89257495	3.42837150
H	-1.14233396	4.25454194	2.37694896
H	-2.14661732	2.94433316	1.71326698
N	-0.05980451	2.42852866	2.09224713
C	-0.01131231	3.57658241	-0.17932269
N	0.89917979	3.64106862	-1.31960725
C	2.03674800	4.53625786	-1.13375460
H	2.64999406	4.56402728	-2.05764374
H	1.75572621	5.59093353	-0.87256159
H	2.69386256	4.16537820	-0.31803983
H	-1.04467718	3.40624730	-0.54419607
H	0.38288904	3.91242402	-2.16269362
**F2-TSb**			
P	1.99010834	-0.90728510	-0.01868926
C	2.45086864	-0.13621588	-1.66252564
C	1.19158698	-0.07415358	-2.52554395
H	3.28303792	-0.65800798	-2.17706047
H	2.76938404	0.90455553	-1.43079813
H	1.36303649	0.58943013	-3.40775210
H	0.91235801	-1.07440037	-2.92142207
N	0.05450395	0.44996380	-1.71434195
C	-1.14108802	0.64709034	-2.58085794
C	-2.30872694	1.18700554	-1.75553563
H	-1.40246674	-0.32272710	-3.05494452
H	-0.89335067	1.35667165	-3.40734828
H	-2.09738018	2.23555796	-1.45198758
H	-3.25194598	1.20180761	-2.33966043
P	-2.42326632	0.18354180	-0.17300740
Mn	-0.26819021	-0.53558108	0.07235433
C	-0.64187552	-2.15133422	-0.46476023
O	-0.90205639	-3.26492749	-0.80473870
C	-0.40595223	-0.90132007	1.79037146
O	-0.50136667	-1.17117294	2.94708147
C	3.16236855	-0.00977567	1.17421495
C	4.61704843	0.08182926	0.68220249
C	3.10082804	-0.55421638	2.61222890
H	2.72243221	1.01546471	1.17917827
H	4.70363540	0.56233644	-0.31300333
H	5.21548649	0.69168150	1.39412039
H	5.10470872	-0.91548567	0.62569362
H	2.06163907	-0.68625173	2.97307775

H	3.62425509	-1.52957106	2.70606054
H	3.61134496	0.15191800	3.30249822
C	2.69109000	-2.67365778	-0.07557784
C	2.51873423	-3.35455946	-1.44505942
C	2.10817022	-3.55410739	1.04606758
H	3.78194692	-2.54231744	0.10818816
H	3.01308998	-2.79870102	-2.26683280
H	2.97643590	-4.36738104	-1.41538395
H	1.44818237	-3.48462836	-1.70169443
H	2.19773447	-3.09069959	2.04735463
H	1.03565269	-3.76608086	0.86578329
H	2.64427370	-4.52766729	1.07682866
C	-3.19834073	1.44768963	1.02072293
C	-3.45971333	0.88379242	2.42810147
C	-4.43782886	2.17536201	0.47230079
H	-2.37307367	2.18958951	1.10663683
H	-2.59674896	0.31196562	2.82192208
H	-3.66716620	1.71667848	3.13422649
H	-4.35024604	0.22102872	2.44321918
H	-4.25773131	2.65652097	-0.51013153
H	-5.30617209	1.49110070	0.36396804
H	-4.74660634	2.97609851	1.17912632
C	-3.84815215	-1.03819613	-0.47395295
C	-3.89969690	-2.13187682	0.60995009
C	-3.82112473	-1.66437825	-1.87949610
H	-4.76846471	-0.41664131	-0.39394965
H	-3.90916936	-1.72004126	1.63751706
H	-4.82203637	-2.73966547	0.48432380
H	-3.03272744	-2.81623987	0.52577424
H	-3.89094925	-0.90864005	-2.68764076
H	-2.90528512	-2.26844693	-2.03838029
H	-4.69095696	-2.34666119	-1.99674947
H	0.15443526	1.41649902	0.54894144
C	0.35528132	2.62289309	0.45078272
H	0.37655234	1.53861225	-1.45105982
C	0.84401427	2.98634594	-0.88186036
H	0.21628709	3.70340208	-1.45378717
H	-0.68081492	3.02385231	0.59193390
N	2.25110516	3.11109398	-1.08095548
H	2.76333854	3.41626767	-0.24356859
N	1.24746606	2.91178764	1.56548838
H	1.49361418	3.91151929	1.53550206
C	2.68914244	3.77060522	-2.29491986
H	3.79648464	3.82090458	-2.32533903
H	2.35267447	3.19545203	-3.18637700
H	2.29378320	4.81388962	-2.41520165
C	0.70158178	2.58298138	2.87846998
H	1.43224198	2.84520561	3.67068927
H	-0.26361160	3.10347807	3.11214194
H	0.50754823	1.49250996	2.93993917

INTa

P	2.38152395	0.04529646	-0.10332252
C	2.49797265	0.54948189	-1.90304729
C	1.29292255	-0.02720118	-2.64567719
H	3.45561967	0.26102525	-2.38156012
H	2.42456432	1.65818545	-1.91062116
H	1.22479199	0.41882384	-3.66596837

H	1.38522048	-1.12643851	-2.77902930
N	0.04940824	0.26328513	-1.88326266
C	-1.15376101	-0.16644325	-2.64250423
C	-2.42156461	0.17814513	-1.86231790
H	-1.08428310	-1.25967685	-2.82913411
H	-1.16919507	0.33086849	-3.64096317
H	-2.54134563	1.28125026	-1.80937480
H	-3.32742448	-0.23968692	-2.34702112
P	-2.20506139	-0.38413654	-0.08909183
Mn	0.09509315	-0.25410066	0.14332241
C	0.25576882	-1.97920412	-0.00577834
O	0.36017511	-3.15880704	-0.08700707
C	0.13162743	-0.36288245	1.90590667
O	0.16828677	-0.44838008	3.08488396
C	3.30077883	1.47146940	0.75045592
C	4.71348827	1.73223201	0.19931291
C	3.29834031	1.37470484	2.28527501
H	2.66582693	2.33936238	0.46605418
H	4.72877627	1.85696933	-0.90219651
H	5.11607461	2.66868411	0.64197813
H	5.42309841	0.92041939	0.46486525
H	2.28973441	1.16957150	2.69522035
H	3.98381437	0.58060713	2.64817549
H	3.65419082	2.33256505	2.72140346
C	3.56736960	-1.42030847	0.10798217
C	3.55266642	-2.40707705	-1.07312065
C	3.34123374	-2.14693858	1.44752024
H	4.57237902	-0.94386715	0.13484275
H	3.81154990	-1.92448198	-2.03683600
H	4.31010643	-3.20049759	-0.89426289
H	2.57120851	-2.90967074	-1.18097007
H	3.33474114	-1.45759643	2.31424824
H	2.38226154	-2.70150998	1.44906752
H	4.15535207	-2.88497252	1.61286350
C	-3.34950435	0.81994528	0.82354164
C	-3.20822978	0.78144383	2.35396527
C	-4.82414832	0.73396147	0.39136673
H	-2.95645147	1.79328084	0.45348840
H	-2.15261675	0.84041250	2.68358698
H	-3.75165536	1.64199017	2.80003089
H	-3.64923426	-0.13935619	2.79089831
H	-4.95232346	0.76586872	-0.70997517
H	-5.32257304	-0.18289773	0.77122424
H	-5.38079704	1.59999463	0.80992668
C	-3.10566806	-2.04850412	0.02250303
C	-2.81709652	-2.76067803	1.35707862
C	-2.85761676	-2.97618577	-1.18009794
H	-4.18037504	-1.76288661	0.00601843
H	-3.00928063	-2.11336796	2.23554450
H	-3.46990620	-3.65488450	1.45298907
H	-1.76603005	-3.10700045	1.41250773
H	-3.16037188	-2.51580818	-2.14214710
H	-1.79748019	-3.28826375	-1.25463922
H	-3.46452982	-3.89943051	-1.05877659
H	-0.24789083	1.60078934	0.32284726
C	-0.00505909	2.73845381	-0.06341173
H	0.01172472	1.36194220	-1.79294039

O	0.04705767	2.82623287	-1.39753480
H	0.94299387	2.95501454	0.49320414
C	-1.15936158	3.61871579	0.50088086
H	-1.55651426	3.23847406	1.46756479
H	-0.74124550	4.64091269	0.68999610
O	-2.17777956	3.65441593	-0.48091364
H	-1.61550121	3.44092837	-1.29056696

8

P	-2.34646269	-0.04499037	0.17387091
C	-2.48518982	0.08152784	2.04374185
C	-1.30297539	-0.64381741	2.68550853
H	-3.45303428	-0.29185322	2.43492624
H	-2.42878926	1.16483234	2.28523904
H	-1.26118782	-0.42875687	3.77934126
H	-1.39594673	-1.74448586	2.57288209
N	-0.04613125	-0.21995532	2.02545119
C	1.16714548	-0.78153866	2.66359046
C	2.41787114	-0.21648991	1.99063918
H	1.12401046	-1.88645462	2.56321927
H	1.17550171	-0.55317903	3.75542957
H	2.51730106	0.86390806	2.23227240
H	3.33604818	-0.71408984	2.36329478
P	2.22777020	-0.32898187	0.12333172
Mn	-0.06913271	-0.26503586	-0.11914093
C	-0.18290828	-2.00685161	-0.24214478
O	-0.26543055	-3.19012973	-0.34319225
C	-0.07855530	-0.11358853	-1.87177947
O	-0.08771481	-0.02840296	-3.05306163
C	-3.19821661	1.55493468	-0.36831823
C	-4.55156588	1.82539273	0.31066580
C	-3.30780873	1.68727772	-1.89724689
H	-2.45718660	2.29983854	-0.00376599
H	-4.48961767	1.82170732	1.41727696
H	-4.92830634	2.82616107	0.00694812
H	-5.32363695	1.08595300	0.00794404
H	-2.35625116	1.45019586	-2.41318700
H	-4.09721300	1.02362605	-2.30938136
H	-3.58851491	2.72839234	-2.16579127
C	-3.56221432	-1.40953936	-0.33092254
C	-3.58271272	-2.60810896	0.63465167
C	-3.30039198	-1.87746707	-1.77574073
H	-4.56175756	-0.91991089	-0.29216354
H	-3.86307365	-2.32342250	1.66873705
H	-4.33498065	-3.34884546	0.28702297
H	-2.60226664	-3.12525876	0.66317745
H	-3.25314705	-1.03984788	-2.49851827
H	-2.34602433	-2.43674684	-1.84485566
H	-4.11479333	-2.55902205	-2.10347340
C	3.29821745	1.12592049	-0.44723947
C	3.37791556	1.24296000	-1.97935247
C	4.70052296	1.17214306	0.18487755
H	2.71839583	1.99307294	-0.06008888
H	2.38536172	1.15390737	-2.46435581
H	3.80338499	2.23070140	-2.25868409
H	4.04372934	0.46859515	-2.41601398
H	4.67620844	1.16788844	1.29316279
H	5.33921143	0.32602015	-0.14788798

H	5.21636826	2.10643088	-0.12597349
C	3.23071960	-1.85508162	-0.39171782
C	2.87955430	-2.28889977	-1.82792285
C	3.10541857	-3.03979872	0.58278103
H	4.28903614	-1.50971950	-0.37567538
H	2.94766402	-1.45831479	-2.55704822
H	3.57849541	-3.08631112	-2.16090638
H	1.85157052	-2.69962619	-1.87958406
H	3.44350982	-2.79105117	1.60890109
H	2.06473576	-3.41863768	0.63452215
H	3.74252128	-3.87813305	0.22663236
C	0.12327148	2.78337622	-0.59803364
H	0.00499375	0.81981252	2.03792634
H	-0.82336377	2.94032555	-1.17940677
O	0.04277341	1.74508723	0.33621300
H	0.92318220	2.60136923	-1.36360266
C	0.46276935	4.07623138	0.16975848
H	-0.45411247	4.45986788	0.68906409
H	0.81205818	4.87468937	-0.52207434
O	1.49150606	3.78452375	1.11261996
H	1.25726547	2.84141776	1.32143730
**8a**			
P	-2.29542811	-0.24063477	0.17071480
C	-2.45891325	-0.29857531	2.04192157
C	-1.25373731	-1.03120632	2.63166557
H	-3.41448308	-0.74599321	2.38266555
H	-2.44867743	0.75931911	2.38153668
H	-1.23155640	-0.90985222	3.74081640
H	-1.30467414	-2.12138018	2.42580997
N	-0.01043691	-0.50497785	2.02735476
C	1.21976487	-1.06549832	2.62750722
C	2.44243741	-0.36254452	2.03787361
H	1.24189386	-2.15584578	2.41757826
H	1.20287666	-0.94752128	3.73710556
H	2.46108329	0.69395563	2.38160601
H	3.38651501	-0.83627606	2.37500649
P	2.27739929	-0.29272458	0.16721365
Mn	-0.01109914	-0.32585473	-0.11288675
C	-0.03073081	-2.04554033	-0.44068084
O	-0.04642564	-3.21132586	-0.68377427
C	-0.00794178	0.04033497	-1.83210505
O	-0.00600272	0.27587770	-2.99359830
C	-3.21532370	1.36434874	-0.22692088
C	-4.59294279	1.50053692	0.44378427
C	-3.30122711	1.65083263	-1.73585749
H	-2.51821751	2.10123317	0.22930903
H	-4.55255202	1.38238644	1.54520783
H	-5.01167704	2.50956059	0.23764521
H	-5.32291727	0.76294934	0.04666776
H	-2.32744390	1.51902725	-2.24752091
H	-4.04503000	0.99443411	-2.23537992
H	-3.63271347	2.69850534	-1.90202411
C	-3.44218164	-1.60590339	-0.47559264
C	-3.39872554	-2.89404074	0.36569851
C	-3.15546683	-1.91328859	-1.95801144
H	-4.46613441	-1.17571377	-0.39348247
H	-3.69368786	-2.72858964	1.42144075

H	-4.10978927	-3.63620616	-0.05755641
H	-2.39169859	-3.35786104	0.34823150
H	-3.16325935	-1.00749073	-2.59494487
H	-2.16763153	-2.40140750	-2.07752129
H	-3.92609506	-2.60944169	-2.35408598
C	3.24540784	1.28577055	-0.22517155
C	3.33294696	1.57925921	-1.73273563
C	4.63011155	1.37438954	0.43922789
H	2.57781215	2.04459832	0.23854935
H	2.35344021	1.47899293	-2.24073344
H	3.69281936	2.61824356	-1.89322925
H	4.05518393	0.90473443	-2.23967561
H	4.59168635	1.24990177	1.54005705
H	5.33521297	0.61780320	0.03311118
H	5.07779201	2.37177904	0.23780014
C	3.38549536	-1.68663461	-0.48618909
C	3.08807103	-1.98065345	-1.96910534
C	3.31057938	-2.97612864	0.35078636
H	4.42053079	-1.28392859	-0.40480176
H	3.12007902	-1.07297998	-2.60262591
H	3.83856229	-2.69636715	-2.36896746
H	2.08691742	-2.44071924	-2.08880637
H	3.61149719	-2.82169146	1.40651220
H	2.29229500	-3.41463226	0.33363752
H	4.00224674	-3.73434977	-0.07616841
C	0.04689491	2.77765806	-0.13895059
H	0.00398071	0.53808094	2.10484972
H	-0.85665257	2.92366808	-0.80178849
O	-0.00040184	1.60586916	0.59932476
H	0.92370836	2.83941336	-0.84002187
C	0.12493587	4.00579953	0.78866080
H	1.03617680	3.93071338	1.42225942
H	-0.75122796	3.99245069	1.48665479
O	0.22618716	5.23521614	0.07512900
H	-0.55880444	5.28950775	-0.50444599
